# Impaired beta-oxidation increases vulnerability to influenza A infection

**DOI:** 10.1016/j.jbc.2021.101298

**Published:** 2021-10-09

**Authors:** Sebastiaan van Liempd, Diana Cabrera, Carolin Pilzner, Heike Kollmus, Klaus Schughart, Juan M. Falcón-Pérez

**Affiliations:** 1 Metabolomics Platform CIC bioGUNE-BRTA, Derio, Spain; 2 Department of Infection Genetics, Helmholtz Centre for Infection Research, Braunschweig, Germany; 3 University of Veterinary Medicine Hannover, Hannover, Germany; 4 Department of Microbiology, Immunology and Biochemistry, University of Tennessee Health Science Center, Memphis, Tennessee, USA; 5 IKERBASQUE, Basque Foundation for Science, Bilbao, Spain

**Keywords:** influenza A virus, mouse model, diabetes, beta-oxidation, metabolomics, MS, ABCD, ATP-binding cassette D, ACar, acylcarnitine, ACC, acetyl-CoA carboxylase, ACN, acetonitrile, AcCoA, acetyl coenzyme A, ACoA, acyl-CoA, Ahr, aryl-hydrocarbon receptor, C5, complement component 5, CPT, carnitine palmitoyltransferase, DC, dendritic cell, dpi, days postinfection, FA, fatty acid, Gln, glutamine, 5HIU, 5-hydroxyisourate, hrLCMS, high-resolution liquid chromatography–coupled mass spectrometry, IAV, influenza A virus, Ile/Leu, isoleucine/leucine, LCFA, long-chain fatty acid, LME, linear mixed-effect, LPC, (lyso)phosphatidylcholine, LPE, lysophosphatidylethanolamine, LPI, lysophosphatidylinositol, MCar, malonylcarnitine, MCFA, medium-chain fatty acid, MCoA, malonyl-coenzyme A, MNA, *N*^1^-methylnicotinamide, NAM, nicotinamide, nPY, *N*^1^-methyl-*n*-pyridone-5-carboxamide, PAMP, pathogen-associated molecular pattern, PP2A, protein phosphatase 2A, Phe, phenylalanine, TAG, triacylglycerol, TCA, tricarboxylic acid, TLR, Toll-like receptor, TMAO, trimethylamine N-oxide, Val, valine, VLCFA, very long–chain fatty acid

## Abstract

Influenza A virus (IAV) infection casts a significant burden on society. It has particularly high morbidity and mortality rates in patients suffering from metabolic disorders. The aim of this study was to relate metabolic changes with IAV susceptibility using well-characterized inbred mouse models. We compared the highly susceptible DBA/2J (D2) mouse strain for which IAV infection is lethal with the C57BL/6J (B6) strain, which exhibits a moderate course of disease and survives IAV infection. Previous studies showed that D2 has higher insulin and glucose levels and is predisposed to develop diet-induced type 2 diabetes. Using high-resolution liquid chromatography–coupled MS, the plasma metabolomes of individual animals were repeatedly measured up to 30 days postinfection. The biggest metabolic difference between these strains in healthy and infected states was in the levels of malonylcarnitine, which was consistently increased 5-fold in D2. Other interstrain and intrastrain differences in healthy and infected animals were observed for acylcarnitines, glucose, branched-chain amino acids, and oxidized fatty acids. By mapping metabolic changes to canonical pathways, we found that mitochondrial beta-oxidation is likely disturbed in D2 animals. In noninfected D2 mice, this leads to increased glycerolipid production and reduced acylcarnitine production, whereas in infected D2 animals, peroxisomal beta-oxidation becomes strongly increased. From these studies, we conclude that metabolic changes caused by a distortion of mitochondrial and peroxisomal metabolism might impact the innate immune response in D2, leading to high viral titers and mortality.

Every year, the influenza epidemic costs the lives of hundreds of thousands of people worldwide. The severity of disease is determined by many environmental and intrinsic factors. Besides age, diabetes, obesity, and metabolic syndrome are major risk factors for severe outcome (1, 2). However, the complexity of factors that impact susceptibility to influenza makes it difficult to assess the underlying mechanism in humans and necessitate studies in well-controlled experimental models. In this work, we compared two well-characterized mouse strains with different metabolic phenotypes and different susceptibilities to influenza A virus (IAV) infections. We aimed to find metabolic pathways correlated with adverse outcome of IAV infection.

This study makes use of the C57BL/6J (B6) and DBA/2J (D2) mouse strains. B6 mice recover readily from infection with a low virulent IAV strain, whereas such an infection is lethal in D2 mice. Genetic mapping has identified several genomic regions that are associated with the increased susceptibility of D2 mice to infections. However, the detailed mechanisms are still elusive (3, 4, 5).

Furthermore, both mouse strains were previously studied in different metabolic contexts, including diabetes, energy metabolism (6, 7, 8, 9, 10, 11, 12, 13), and hepatic steatosis (14). These studies revealed that under normal and nonchallenge conditions, levels of insulin, triacylglycerides, and fat mass are about twice as high in D2 compared with B6. In addition, D2 is more predisposed to the development of diet-induced type 2 diabetes. Therefore, these interstrain metabolic differences might further explain the difference in susceptibility to IAV infections.

By repeated sampling of individual animals early after infection and up to 30 days postinfection (dpi), we compared the plasma metabolomes of healthy and infected B6 and D2 animals. We used an untargeted and high-resolution liquid chromatography–coupled mass spectrometric (hrLCMS) approach to detect and quantify plasma metabolites. After multistep data analysis, changes in several metabolite families were found between strains and within strains upon infection. Observed differentially expressed metabolites were mapped to canonical pathways. Taking in account prior knowledge and the newly observed interstrain metabolic differences in healthy and infected animals, we propose a hypothesis for the high susceptibility of D2 mice to IAV infection. This may shed light on the cause for severe disease in human patients and aid in development of novel therapeutic approaches in patients with metabolic comorbidities.

## Results

Metabolic responses were measured by an untargeted hrLCMS approach. Infection with IAV invoked physiological and metabolic changes in both mouse strains. Some effects were similar between strains but many differed. Moreover, animals in the control arm of the experiment (mock infection and 0 dpi) also showed metabolic interstrain differences. The results with respect to hrLCMS data analysis, metabolic effects, and physiological behavior are described later.

### Physiological changes upon infection

**Figure 1:**
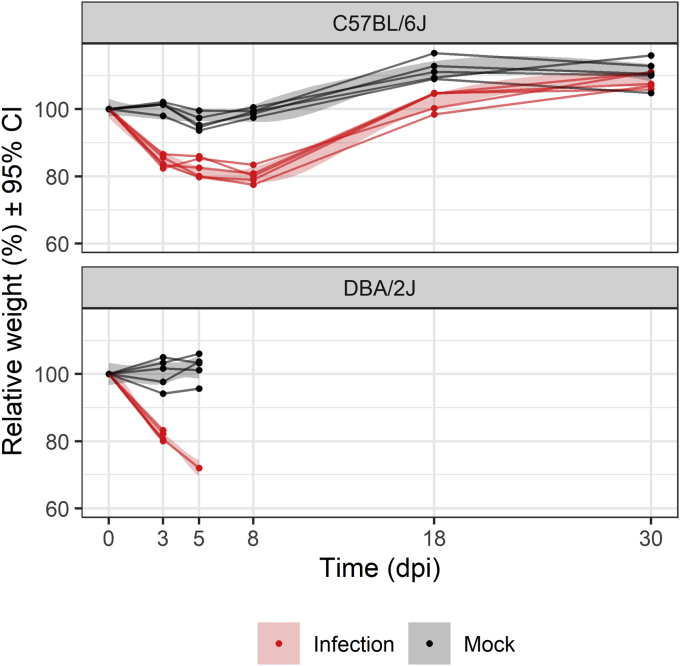
**Changes in body weights after IAV infection for D2 and B6 mouse strains.** CI, 95% confidence interval for the LOESS fit; IAV, influenza A virus.

Mice were either infected with influenza H1N1 virus or mock treated. While B6 mice recovered from this infection, D2 mice in the test group were all dead or had to be euthanized after 5 dpi. The infected B6 animals lost around 20% of their initial weight at 3 dpi. For infected D2 animals, weight loss was about 25% (*p* = 0.04 between strains at 3 dpi; Fig. 1). The infected B6 strain recovered its initial weight after 14 dpi.

### Analysis of metabolomics data

To track the plasma metabolomes of the animals, raw hrLCMS data were cleaned up, adjusted, and modeled. After automatic peak extraction, 10,350 features in positive electrospray ionization mode and 1461 features in negative electrospray ionization mode were collected over all the samples. Noise reduction and other cleaning steps reduced these numbers by about 63%, leaving the total number of features at 4318. After subsequent data adjustment, 1641 features showed a *p* value of less than 0.001 in a linear mixed-effect (LME) model. Subsequent post hoc analysis resulted in a selection of 706 features that showed differences between either mock and infected groups or between strains. This feature set was then manually integrated. The reintegrated signals were again subjected to adjustments and statistical filtering. Finally, 106 selected features were identified, and 52 more features were added by pathway enrichment, resulting in a final set containing 158 identified metabolites (Fig. S1). Most of these metabolites were related to specific canonical metabolic pathways, that is, beta-oxidation, phospholipid, nucleotide, and nicotinamide (NAM) metabolism. The final set was used to form a hypothesis explaining the increased vulnerability of D2 mice to IAV infection. The entire set, including chemical details, is included in Table S1.

### Metabolic responses

**Figure 2:**
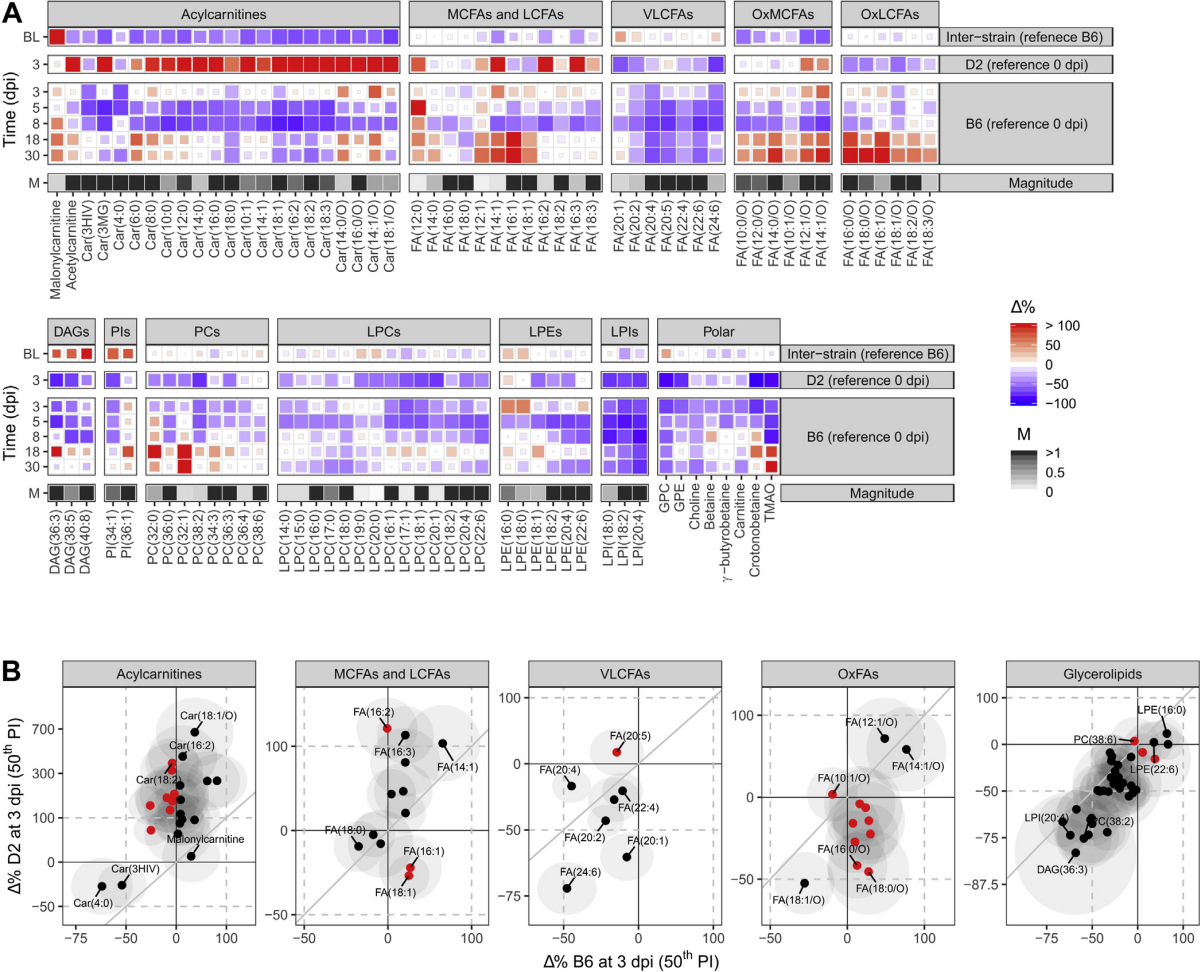
**Responses in lipid metabolism in healthy and infected animals for D2 and B6 strains.** Heatmaps of percent change from baseline (Δ%) for the lipid-associated metabolites in serum (*A*) and correlations between Δ% for these metabolites within each lipid class for both strains at 3 dpi (*B*). The colors of the heatmap tiles define the direction of the effects, whereas their sizes define the credibility of this direction. For clarity, color scales for positive effects (*red*) are cut off at 100% so that extreme values do not dominate the scale. The *top row* shows the differences between strains (interstrain) for baselines (BL, *i.e.*, pooled 0 dpi and mock 3 and 5 dpi results). Since the reference strain was taken to be B6, a positive Δ% means an increase in D2 levels (*e.g.*, MCar). The *second row* shows the Δ% for D2 between 0 dpi (reference) and infected 3 dpi. Rows 3 to 7 show the Δ% for B6 baseline and infected day 3 to day 30. The relative magnitudes for the raw signals with respect to the median within these finer classifications are shown in the *bottom rows*. The magnitude *gray* scale starts at 0 and is cut off at 1 where 1 would be the metabolite with the median value per class. Note that these magnitudes are only informative for metabolites that have similar ionization efficiencies; hence, no magnitude is displayed for the “polar” group. Interstrain correlations for Δ% per lipid class at 3 dpi (log scales) are displayed in panel *B*. The diagonal *gray line* indicates the identity line (*i.e.*, equal effects in both strains). The *ellipses* indicate the 50% posterior percentile intervals per metabolite. *Red dots* indicate metabolites with opposite effects between strains. The panel “Glycerolipids” in (*B*) include all detected DAGs, (L)PIs, (L)PCs, and LPEs. DAGs, diacylglycerols; LCFAs, long-chain fatty acids; (L)PCs, (lyso)phosphatidylcholines; LPE, lysophosphatidylethanolamines; (L)PIs, (lyso)phosphatidylinositols; MCFAs, medium-chain fatty acids; OxFAs, oxidized fatty acids; VLCFAs, very long–chain fatty acids.

**Figure 3:**
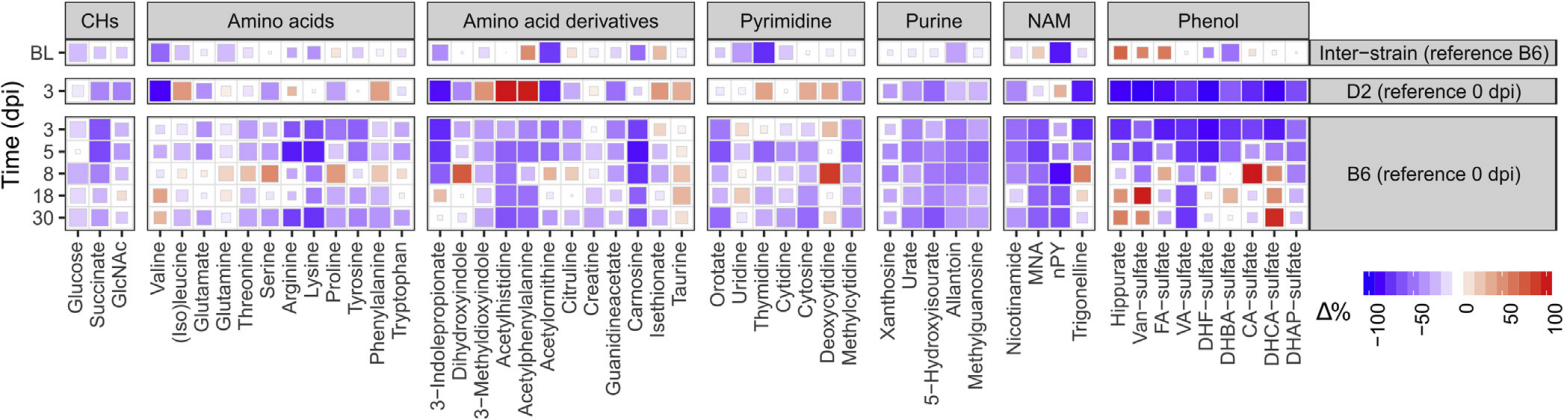
**Heatmaps of percent change from baseline (Δ%) for metabolites in serum.** The colors of the heatmap tiles define the direction of the effects, whereas their sizes define the credibility of this direction. For clarity, color scales for positive effects (*red*) are cut off at 100% so that extreme values do not dominate the scale. Interstrain correlations for Δ% at 3 dpi (log scales) are displayed in panel *B*. For abbreviations of metabolites, see Table S1. CHs, carbohydrates and related; NAM, nicotinamide.

We determined differences in metabolite levels *between strains* (interstrain) for baseline and infection and *within strains* (intrastrain) for infection (Figs. 2 and 3 and Table S2). Results for each metabolite were calculated as percentage change (%Δ) from relevant baselines (*i.e.*, B6 for interstrain, 0 dpi for intrastrain). The 50% and 90% percentile intervals roughly represent the shape of the posterior joint distributions of the simulated %Δ values. The effects for IAV infection in B6 were analyzed for 3, 5, 8, 18, and 30 dpi. Since infections were lethal for D2, no reliable metabolic data were available beyond 3 dpi for this strain, and hence, only analysis of 3 dpi was included. Combined raw and model data for each metabolite are included in Figure S2.

#### Acylcarnitines

The most important differences between and within strains were observed for acylcarnitines (ACars; Fig. 2*A*). Levels in healthy animals for most ACar species were approximately −50% lower in D2 compared with B6. Conversely, malonylcarnitine (MCar) was +505% higher in D2.

During infection at 3 dpi, B6 ACar levels with six or more acyl-carbons stayed more or less stable, whereas D2 levels showed a strong increase, ranging from +56% for Car(6:0) to +670% for Car(18:1/O) (Fig. 2, *A* and *B*). Note from Figure 2*B* that there is a positive correlation between relative effect sizes in both strains with a positive offset for D2. This means that ACars that are upregulated in B6 are stronger upregulated in D2 (*e.g.*, Car(18:1/O)); ACars that were downregulated in B6 are moderately upregulated in D2 (*e.g.*, Car(18:2), red points, opposite effects); and ACars that were strongly downregulated in B6 were moderately downregulated in D2 (*e.g.*, Car(4:0)).

During 5 dpi to 8 dpi, the medium-chain and long-chain ACar levels in B6 decreased between −38% and −83%. During recovery, a divergence of ACar levels with respect to oxidation state and chain length was observed in B6 mice. Most oxidized short-chain and medium-chain ACars were increased, whereas the remaining species stayed equal or showed a decrease, compared with healthy baseline.

#### Fatty acids

In healthy animals, the most abundant long-chain fatty acids (LCFAs) and very long–chain fatty acids (VLCFAs), that is, FA(16:0), FA(18:0), FA(18:1), FA(20:4), and FA(22:6), showed no important differences between strains (Fig. 2*A*).

After infection, most unsaturated medium-chain fatty acids (MCFAs) and LCFAs showed no change or an increase in both strains. Increases were slightly higher in D2, equivalent to ACars (Fig. 2, *A* and *B*). Exceptions were FA(18:1) and FA(16:1), which showed opposite interstrain effects, namely an increase of about +20% in B6 and a decrease of about −30% in D2 (red points in the panel of MCFAs and LCFAs; Fig. 2*B*). VLCFAs showed decreases upon infection in both strains. Overall, these decreases were stronger in D2 compared with B6.

FAs showed a strong decease in B6 at 5 and 8 dpi, similar to ACars. Most saturated and monounsaturated MCFAs and LCFAs (*e.g.*, FA(12:0) and FA(16:1)) showed a strong upregulation (from +36% up to +153%) during recovery. Conversely, all the polyunsaturated VLCFAs showed a decrease, ranging from −31% to −65%, after the virus was eliminated at 30 dpi.

#### Oxidized FAs

In healthy animals, oxidized MCFAs were all decreased in D2 compared with B6, ranging from −60% for FA(12:1/O) to −18% for FA(10:1/O). Oxidized LCFAs showed no interstrain differences in healthy animals. Upon infection, saturated oxidized species showed opposite effects between species (red points, OxFAs panel; Fig. 2*B*). In B6, the saturated and oxidized FA levels were higher (up to +70%), whereas in D2, these metabolites showed a decrease (down to −65%). Moreover, all oxidized LCFAs were strongly decreased in D2 at 3 dpi. During disease progression in B6, levels of most oxidized FAs dropped between 5 dpi and 8 dpi. Analogous to MCFAs and LCFAs, all OxFA species showed a strong increase in B6 upon recovery (up to +132%).

#### Glycerolipids

In healthy animals, the biggest interstrain differences for lipids were seen for diacylglycerols (DAGs) and phosphatidylinositols, which were around +80% higher in D2 compared with B6 (Fig. 2*A*). Other detected glycerolipids did not show important differences between strains. Variations for (lyso)phosphatidylcholines (LPCs, PCs), lysophosphatidylethanolamines (LPEs), and lysophosphatidylinositols (LPIs) were approximately between +20% and −20%. The same was observed for polar, lipid, and carnitine-associated metabolites like glycerophosphorylcholine, carnitine, and γ-butyrobetaine. In general, the most abundant members of the glycerolipid families showed the lowest effects.

In general, all lipid species, including DAGs, PCs, LPCs, LPEs, and LPIs, became downregulated upon infection at 3 dpi (Fig. 2*A*). These downregulations were stronger for D2 (Fig. 2*B*). The biggest drops were observed in D2, especially for the DAGs. Plasma levels of DAGs in D2 decreased by −44% to −80% compared with healthy D2 animals, whereas in B6, these drops lay between −27% and −62%. Moreover, strong drops in polar lipid and carnitine-associated metabolites were seen in infected animals of both strains; for example, glycerophosphorylcholine, glycerophosphorylethanolamine, crotonobetaine, and trimethylamine N-oxide (TMAO). During the recovery period in B6, betaine, crotonobetaine, and TMAO were upregulated between 8 and 30 dpi.

#### Carbohydrates

Baseline glucose and succinate levels in healthy animals were more than −20% lower in D2 compared with B6 (Fig. 3) and were not clearly affected by infection in either strain. Succinate was about −50% lower during infection in both species at 3 dpi. In B6, succinate stayed decreased at 5 dpi to recover slightly under baseline after 18 dpi.

#### Amino acids and derivatives

Interstrain differences in baseline plasma levels in healthy animals were detected for amino acids and their derivatives (Fig. 3). Decreases in D2 compared with B6 were found for the essential AA lysine (Lys; −38%) and branched chain AAs valine (Val; −55%) and isoleucine/leucine (Ile/Leu; −21%). Glutamine (Gln) levels were −23% lower in D2. The most important interstrain difference for AA derivatives was observed for acetylornithine and was −71% lower in D2.

Upon infection, Val levels at 3 dpi dropped by −88% in D2, whereas virtually, no change was observed in B6. Other differences between infected strains were seen for Ile/Leu and phenylalanine (Phe). These species were about +40% upregulated in D2, whereas a decrease of about −15% was observed in B6. A similar but less pronounced effect was observed for Gln (+9% in D2 *versus* −9% in B6). Furthermore, in infected animals, Lys, arginine, and tyrosine were decreased by about −50% in B6, whereas no change was observed in D2. Effects for AA derivates tracked in equal directions between strains, with the bigger effect sizes observed in D2. Upregulations were seen for sulfur-containing derivatives, isethionate and taurine, whereas most other derivatives showed downregulations. Especially, 3-indolepropionate was downregulated by about −80% in both species. Differential effects were seen for acetylated histidine and Phe and 3-methyldioxyindole, which were strongly upregulated in D2 (between +50% and +100%) and downregulated in B6 (all around −35%).

For B6, the amino acid profile on 5 dpi was the same as on 3 dpi. Levels of most AAs tended to normal between 8 dpi and 18 dpi, yet stayed somewhat below initial levels. However, some increase with respect to the healthy B6 baseline was seen for proline, Phe, serine, and Val. At 30 dpi, all detected species except Val were lower than they were initially.

#### Nucleobases

In healthy animals, the pyrimidine nucleobases thymidine, uridine, and cytidine were all lower in D2 (respectively −76%, −35%, and −19%) with respect to B6 (Fig. 3). The purine-derived metabolite allantoin was also lower in D2 by −32%. After infection, at 3 dpi, the changes in both strains moved in the same direction except for cytosine, which was +36% higher in D2 and −16% lower in B6. In both strains, the biggest observed changes were the decreases of the pyrimidine base 5-methylcytidine (by about −40%), and all detected purine-related metabolites, that is, allantoin, urate, methylguanosine, and 5-hydroxyisourate (5HIU) (between −21% and −50%). Deoxycytidine was increased by about +35% in both strains. Moreover, compared with 0 dpi, this metabolite generated a substantial spike (+88%) in B6 at 8 dpi and returned to normal at 18 dpi. After recovery, most detected nucleobase-related metabolites were still lower after 8 dpi in B6.

#### NAM

Interstrain differences in healthy animals were detected for metabolites in the NAM pathway (Fig. 3). *N*^1^-methyl-*n*-pyridone-5-carboxamide (nPY, n = 2 or 4) was −83% lower in D2. Conversely, levels of *N*^1^-methylnicotinamide (MNA) were +23% elevated in D2.

During infection, different responses between strains were seen for nPY. Levels for this metabolite went up in D2 by +36% and down in B6 by −30% at 3 dpi. Other detected metabolites of the NAM pathway were downregulated in both strains during infection with the strongest effect seen for trigonelline, which was decreased in both strains by about −80%. In B6, except for trigonelline, all NAM-related metabolites stayed downregulated after recovery. The strongest downregulation during the recovery phase in B6 was seen for nPY (−86%). Trigonelline saw an increase of around +57% at 8 and 18 dpi to return to normal at 30 dpi.

#### Phenols

Sulfated phenolic compounds showed interstrain differences in baseline concentrations, ranging from −49% decrease (dihydroxybenzoic acid sulfate) to +59% increase (ferrulic acid sulfate) in D2 with respect to B6 (Fig. 3). Moreover, hippuric acid was +71% decreased in D2. During infection, in both species, the plasma levels of all phenolsulfates dropped by around −80% at 3 dpi with stronger negative effects observed in D2 In B6, these levels stayed reduced by the same amount at 5 dpi. In the recovery phase, some of the phenols returned to normal, whereas most of them exceeded the initial levels up to +100%. Only vanillic acid sulfate kept on being reduced.

#### Nutritional markers

**Figure 4:**
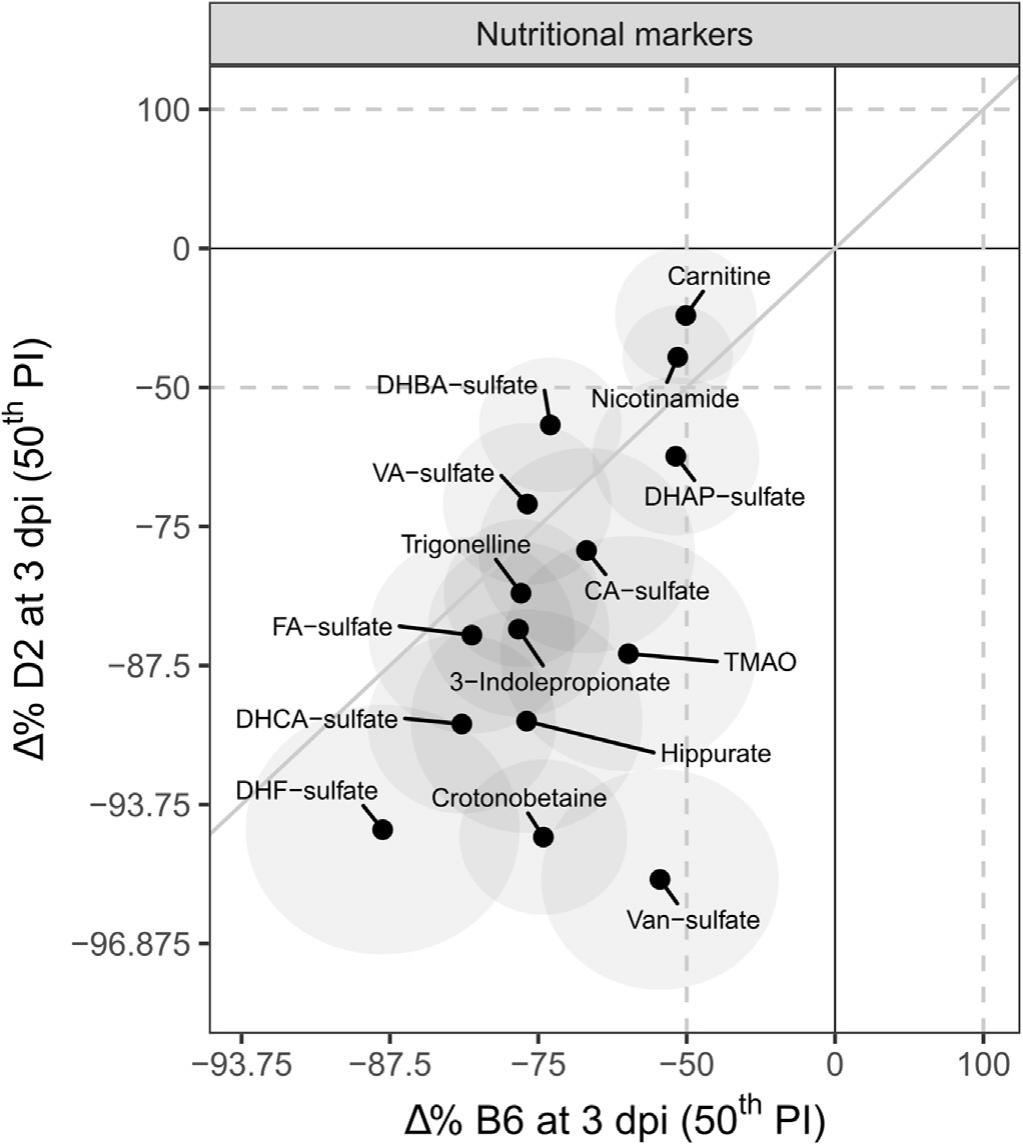
**Correlations between Δ% for nutritional markers for both strains at 3 dpi.** The diagonal *gray line* indicates the identity line (*i.e.*, equal effects in both strains). The *ellipses* indicate the 50% posterior percentile intervals per metabolite.

Some of the differentially expressed metabolites are partially or entirely extracted from the diet or are produced by the microbiome. These nutritional markers include carnitine, crotonobetaine, NAM, trigonelline, 3-indolepropionate, and the polyphenols including hippurate. In both strains, these markers decrease during infection at 3 dpi (Fig. 4). However, the decrease is stronger in D2 compared with B6 for most markers, for example, TMAO, hippurate, crotonobetaine, and vanillin 4-sulfate.

#### Omitted metabolites

To keep the article concise, some metabolite classes were omitted from the Results section. Omission was based on whether metabolites were used in the mechanistic discussion. Metabolite classes that were omitted were a miscellaneous group including vitamins. These omitted markers are included in the supporting information (Tables S1 and S2, and Figs. S2 and S3).

## Discussion

Influenza can be a life-threatening disease in immunocompromised individuals, which is clearly observed in patients suffering from metabolic disorders. Thus, new insights into pathophysiological metabolic mechanisms involved in IAV infection could help to ameliorate symptoms in these patients through tailored therapeutic or dietary interventions. Therefore, in this work, plasma from mouse models of high and moderate influenza disease was used to study differential metabolic responses to IAV infection. These responses were subsequently used to infer a mechanism as to why the D2 strain is highly vulnerable to IAV, whereas B6 is not.

However, some limitations are associated to plasma metabolic studies given that an important characteristic of metabolism is the compartmentalization of metabolic processes on organ, cell, or organelle level. Hence, information on the origin or destination of detected plasma metabolites is obscured since all organs and cell types contribute to or extract from the plasma metabolome. What is more, the plasma metabolome contains mainly metabolites that can, either actively or passively, traverse cell membranes depending on, for example, chemical nature or concentration. This means that in plasma, not all members of a certain metabolic pathway can be detected. This incompleteness problem is aggravated by the fact that the chemical diversity of metabolites is vast, that is, they vary in molecular weight, polarity, stability, or concentration. This is a challenge from an analytical chemical perspective. Despite hrLCMS being one of the more sensitive and selective methods available, it is simply not possible for a single technique to detect or to identify the entire metabolite set.

Nevertheless, a big corpus of information is available on most metabolic pathways, including their members, interactions, and locations. Moreover, genomic, transcriptomic, proteomic, and metabolomic information is available for the B6 and D2 strains. As such, it is known that both B6 and D2 mice have a predisposition for diet-induced type II diabetes, but D2 has the worst outcome. It is the D2 strain that shows a diabetic phenotype from the outset, including insulin resistance and lipid accumulation (6, 12). We used this prior information, together with the observed changes in the plasma metabolome from this untargeted metabolomics study, to deduce a hypothesis for the heightened vulnerability of D2 toward the IAV.

### Mitochondrial beta-oxidation is decreased in noninfected D2 mice

High baseline plasma levels of MCar in D2 suggest decreased mitochondrial beta-oxidation, which is offset by other energy-generating processes including increased tricarboxylic acid (TCA) cycle activity and peroxisomal beta-oxidation.

The main observed metabolic differences between strains at baseline were the vastly increased plasma levels of MCar in D2. MCar is a proxy for the production of malonyl-coenzyme A (MCoA), and differences in plasma levels of MCar reflect intracellular concentrations of MCoA (15, 16). MCoA plays a crucial role in lipid metabolism. It is a strong inhibitor of carnitine palmitoyltransferase I (CPT1) and an essential substrate for the fatty acid synthase complex and elongation of very long–chain fatty acid enzymes.

**Figure 5:**
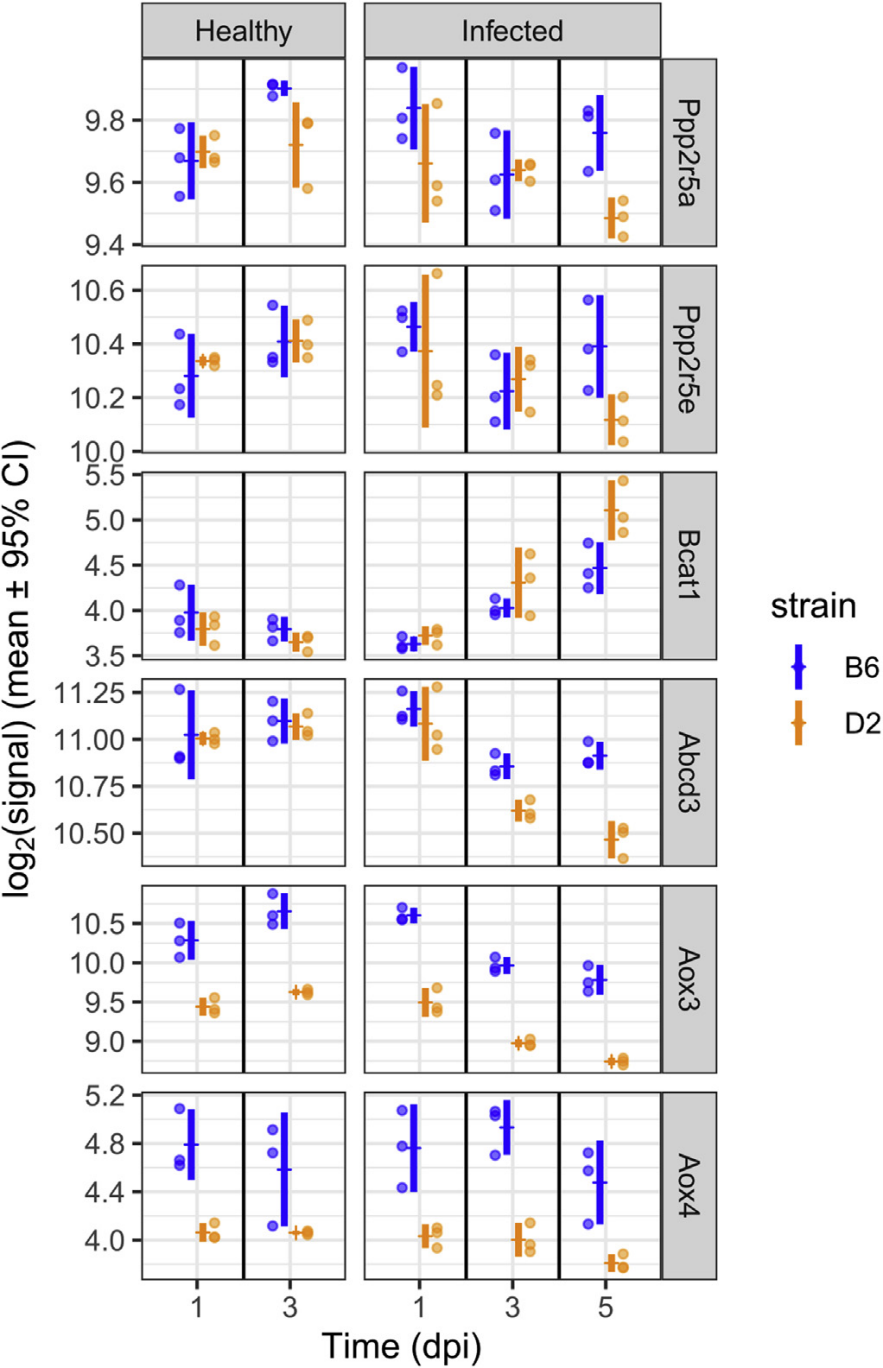
**Transcription levels of various genes from previously published data** (20)**.***Dots* represent the measured log2-transformed signals for both strains where B6 is represented in *blue* and D2 in *orange*. The *vertical bars* represent the 95% confidence intervals around the mean for a normal distribution.

**Figure 6:**
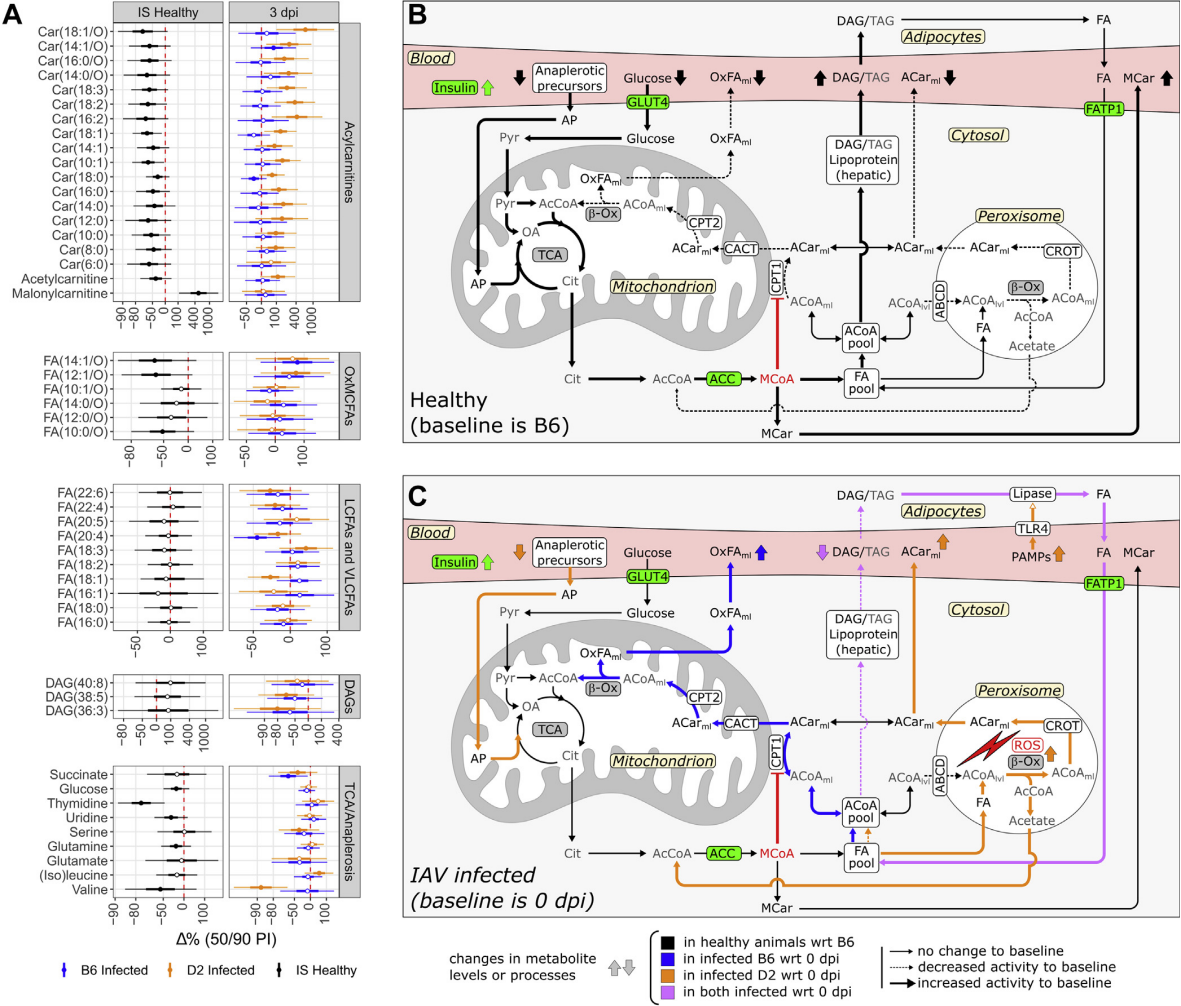
**Proposed changes in energy metabolism between strains in healthy and infected state.** Proposed changes in energy metabolism between strains in a healthy state (*A*) and for D2 in infected state (*B*), which could explain the heightened vulnerability of D2 toward influenza A virus (IAV). Percentage changes (%Δ) within percentile intervals for relevant metabolic classes are added for comparison (*C*). Metabolites for which 80% of the posterior density was either above or below 0 are indicated with *filled dots*. Comparisons of healthy D2 and B6 energy metabolomes suggest that mitochondrial beta-oxidation is inhibited in D2 by malonyl-CoA (MCoA). Compensatory processes (anaplerosis) are increased in D2 to make up for decreased oxidative phosphorylation. This is likely driven by increased insulin levels in D2. During infection, D2 virus titers are extremely high compared with B6 (43), possibly leading to increased TLR4 signaling and fatty acid (FA) recruitment from adipose tissue. The surplus of FA cannot be efficiently utilized by D2 because of the continuous blockade of mitochondrial beta-oxidation by MCoA. Fatty acids in D2 are routed to peroxisomal beta-oxidation, thereby increasing the production of reactive oxygen species and ACars. Moreover, amino acids like valine and serine are used to fuel the TCA cycle in D2 during infection, thereby depleting energy deposits more than in B6. Thus, possible driving factors for the increased vulnerability of D2 are impaired mitochondrial beta-oxidation, depletion of energy reserves, and increased production of reactive oxygen species. Metabolites depicted in *gray* were not directly observed. Enzymes in *green* are upregulated by insulin. ABCD, ATP-binding cassette D; ACar, acylcarnitine; ACC, acetyl-CoA carboxylase; ACoA, acyl-CoA; AcCoA, acetyl coenzyme A; AP, anaplerotic precursor; β-Ox, beta-oxidation; CACT, carnitine-acylcarnitine translocase; Cit, citrate; CPT1/2, carnitine palmitoyl transferase ½; DAG, diacylglycerol; FATP1, fatty acid transporter protein 1; GLUT4, glucose transporter 4; MCar, malonylcarnitine; OA, oxaloacetate; OxFA, oxidized fatty acids (ml: medium/long, lvl: long/very long); PAMPs, pathogen-associated patterns; Pyr, pyruvate; TAG, triacylglycerol; TCA, tricarboxylic acid cycle.

MCoA is synthesized by acetyl-CoA carboxylase (ACC). This enzyme has two isoforms, ACC1 and ACC2, which are expressed at different intracellular locations. ACC1 is located in the cytosol and associated with lipid synthesis. The ACC1 isoform shows overall the highest expression, specifically in adipose and liver tissues (17). ACC2 is located at the mitochondrial outer membrane and therefore associated with the inhibition of CPT1. Insulin activates ACC *via* dephosphorylation by protein phosphatase 2A (PP2A) (18). Earlier findings showed that under basal glucose levels, islet cells in D2 hypersecrete insulin in comparison to B6 (8, 9, 13). In addition, many other studies report increased basal and fasted insulin levels in D2 when compared with B6 (6, 7, 12). Therefore, elevated plasma insulin levels and subsequently altered downstream processes in D2 could explain the increased intracellular levels of MCoA and hence MCar plasma levels. Moreover, a transcriptomics study in healthy lung tissue showed that RNA levels of two isoforms of the regulatory subunit of PP2A (*i.e.*, *Ppp2r5a* and *Ppp2r5e*) were about 2-fold higher in D2 compared with B6 (19). However, these results were not reproduced in unpublished data from a previous transcriptomics study (20) (Fig. 5). Nevertheless, a 5-fold increase in MCoA is likely to inhibit CPT1, leading to a decrease in the formation of ACars, an effect clearly observed in this study. Since CPT1 is part of the mitochondrial acyl-CoA (ACoA) shuttle, inhibition of this enzyme decreases the transport of acyl esters into the mitochondria. Consequently, mitochondrial beta-oxidation would be decreased in D2. This is reflected in the lower baseline levels of oxidized MCFAs, which are intermediates and biomarkers of mitochondrial beta-oxidation (21) (Fig. 6, *A* and *B*).

In the context of beta-oxidation, it should also be mentioned that the D2 strain expresses an aryl-hydrocarbon receptor (Ahr) allele with a 10 to 100 times lower ligand affinity compared with B6. It was previously shown that stimulation of this receptor increased the transcription of *Cpt1b*, coding for the mitochondrial form of CPT1 in liver (22). On the other hand, under high-fat diet, liver-specific knockout of Ahr caused increased expression of lipogenesis-related genes coding for proteins ACC1 (*Acaca*) and glycerol-3-phosphate acyltransferase 1 (*Gpam*) (23). Moreover, indirect inhibition of AHR in CD4^+^ T cells led to the decrease of CPT1 protein expression and activity, whereas ACC2 was upregulated and its phosphorylated inactive form downregulated (24). Therefore, the D2 deficiency in AHR response might work in concert with increased insulin levels causing increased levels of MCar and decreased levels of ACars.

Decreased mitochondrial beta-oxidation and a subsequent deficit in ATP production might be offset by increased glucose utilization driven by increased D2 insulin levels. Evidence for this is found in decreased baseline plasma glucose levels in D2 as observed in this study and others (6, 7, 12). Furthermore, decreased beta-oxidation could in part be compensated by increased catabolism of amino acids (25). Evidence that this is the case in D2 can be found in lower plasma levels of the (branched-chain) amino acids Val, Ile/Leu, and Glu, which can be used as alternative energy sources. Increased mitochondrial utilization of glucose and amino acids would lead to increased TCA cycle activity and increased levels of TCA intermediates, including citrate. Excess citrate is transported from the mitochondria to the cytosol where it serves as a source of cytosolic acetyl coenzyme A (AcCoA) for subsequent production of MCoA and hence FAs (26).

**Figure 7:**
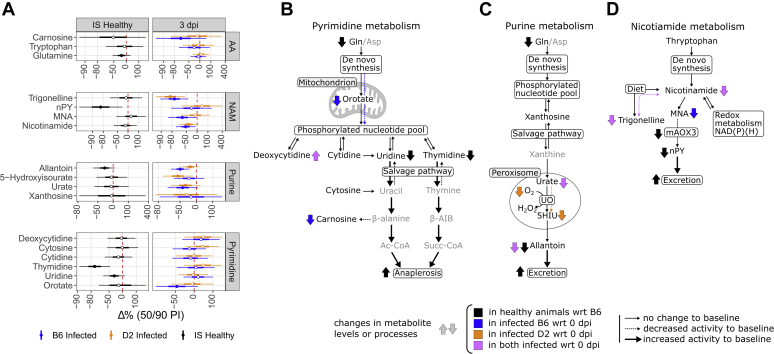
**Proposed changes in miscellaneous pathways showing interstrain differences in healthy and infected states.** Pyrimidine metabolism (*A*), purine metabolism (*B*), and nicotinamide metabolism (NAM) (*C*). Percentage changes (%Δ) within percentile intervals for relevant metabolic classes are added for comparison (*D*). Pyrimidine metabolism flux toward anaplerotic substrates might be increased in healthy D2 animals. For purine metabolism, increased renal excretion could lead to a higher clearance of allantoin. Upon infection, 5-hydroxyisourate (5HIU) levels in D2 might be increased because of lower oxygen availability in peroxisomes. As for NAM metabolism, low baseline levels of *N*^1^-methyl-*n*-pyridone-5-carboxamide (nPY, n = 2 or 4) is likely because of impaired function of aldehyde oxidase homologs 1 (mAOX3) enzyme in D2 and an increased renal excretion.

The pyrimidine nucleosides uridine and thymidine are also implicated in energy metabolism. Both nucleosides are strongly decreased in D2 compared with B6 at baseline. Orotate shows no difference between strains. Therefore, *de novo* pyrimidine synthesis is likely the same in both strains despite lower Gln levels in D2 (Fig. 7, *A* and *B*). Assuming stable phosphorylated pyrimidine nucleotide pools (UTP, CTP, etc) decreased levels of dephosphorylated nucleotides would indicate increased catabolism of pyrimidine nucleosides. In mice, thymidine and uridine are catabolized *via* the same set of enzymes, which would explain decreases in both nucleosides. Full catabolism of uridine results in the production of β-alanine, which in turn can be converted to acetate. Acetate is excreted or converted to AcCoA and subsequently MCoA. On the other hand, thymidine catabolism results in the production of beta-aminoisobutyrate, which is converted to succinyl-CoA, an anaplerotic substrate for the TCA cycle. Therefore, increased pyrimidine breakdown could also compensate for decreased D2 beta-oxidation through anaplerosis.

### Glycerolipid synthesis is increased in noninfected D2 mice

Baseline metabolic signatures observed in D2, including high MCar and DAG levels, together with previously observed gene and protein expression profiles suggest that glycerolipid synthesis is upregulated in D2.

As suggested, increased levels of MCoA could lead to increased FA synthesis. Strain-specific increases in D2 healthy plasma levels of saturated FAs and VLCFAs were observed, which might suggest increased FA synthesis. However, FA levels are determined by a myriad of processes including chain elongation and shortening, desaturation, diffusion, and lipase activity. Moreover, with our analytical methods, the desaturation and oxidation positions, which play crucial roles in inferring their origins, could not be determined.

The best evidence of increased FA synthesis in D2 is found in increased baseline levels of DAGs and higher amounts of adipose tissue with respect to B6. Both DAGs and triacylglycerols (TAGs) are produced in hepatocytes and enterocytes from FAs and glycerol-3-phosophate. DAGs, together with TAGs and various other phospholipids, are transported to adipose and muscle tissue in low-density lipoprotein-containing vesicles. Although our analytical method did not allow for the detection of TAGs, lipoprotein-associated DAGs are correlated with the TAGs and reflect TAG synthesis (27). Excess TAGs are stored in adipose tissue as energy reserve. Assuming that dietary FA uptake is equal between noninfected strains, the 2-fold increase of adipose tissue mass in D2 with respect to B6 as observed in various studies (7, 10, 11) can be explained by increased FA production in D2 livers (Fig. 6, *A* and *B*).

Furthermore, the low responsiveness of the D2 AHR might play an additional role in the observed changes in lipid metabolism. The expression of many enzymes involved in TAG synthesis is modulated by AHR. As mentioned, glycerol-3-phosphate-O-acyltransferase is upregulated in *Ahr*-deficient mice under a high-fat diet (23). In addition, diacylglycerol O-acyltransferases and acylglycerol-3-phosphate-O-acyltransferases are downregulated after stimulation of AHR (28). Therefore, reduced AHR signaling in D2 might increase the metabolic flux through the DAG synthesis pathway, thereby increasing the formation of the associated lipids. This, together with increased FA synthesis, would further explain the increased levels of both DAGs and phosphatidylinositols as was observed in D2.

An indirect effect of increased FA synthesis in D2 might be the downregulation of a peroxisomal transporter protein. Transcriptomics results show that in D2 after 1 dpi, transcription levels of the ATP-binding cassette D3 gene (*Abcd3*) in lung tissue are decreased with respect to B6 (Fig. 5, (20)). Moreover, the same study that found higher PP2A transcription also found that RNA levels of Abcd3 in healthy lung tissue were about 40 times lower in D2 (19) although this effect did not show up in data from Ref. (20). ABCD3 is one of the three known peroxisomal transporters of medium-chain and long-chain ACoA esters and is essential for peroxisomal beta-oxidation (29). The physiological reason for the observed downregulation might be that peroxisomal beta-oxidation produces higher amounts of reactive oxygen species than mitochondrial beta-oxidation. Hence, a decrease of peroxisomal ACoA transport in the presence of elevated FA levels would reduce production of reactive intermediates and subsequent cell damage.

### Interstrain difference in NAM and purine pathways for noninfected animals

Finally, a basal interstrain difference was observed for nucleotide metabolism. Changes in purine metabolism are likely driven by changes in renal clearance, whereas changes in pyrimidine metabolism can be explained by difference in aldehyde oxidase function.

Since allantoin is the irreversible end product of purine catabolism, decreases in D2 with respect to B6 at baseline are either caused by decreased levels of upstream precursors or increased excretion. The direct precursors urate and 5-hydroxyisourate neither show interstrain differences nor does the purine nucleoside xanthosine. Therefore, an explanation for low D2 allantoin levels might be found in the higher renal clearance of allantoin (Fig. 7, *A* and *C*). This is supported by the fact that under control conditions, D2 mice have about 20% to 50% higher glomerular filtration rate as B6 (30).

Similarly, interstrain differences were found for nPY and MNA. Both metabolites are the end products of the NAM metabolic pathway (Fig. 7, *A* and *D*). The strong decrease in D2 for nPY can be explained by increased renal excretion in D2. However, D2 has a selective deficit in expression of *Aox3* and *Aox4* coding for aldehyde oxidase 3 and 4 (31, 32). Especially AOX3 is of interest here since it oxidizes MNA to form nPY (33), thus driving the nPY levels in D2 even further down. Transcriptomics data from Wilk *et al.* (20) clearly show that transcription of both *Aox3* and *Aox4* genes is reduced in D2 compared with B6 in both healthy and infected animals (Fig. 5).

### Similarities in metabolic effects during IVA infection in both strains and recovery in B6

Similarities in metabolic responses between strains during infection were seen for phospholipids, carnitine-related metabolites, amino acid derivatives, and phenolic compounds. These changes seem to be driven by nutritional changes during infection and recovery.

Most detected phospholipid species, including PC, LPCs, LPEs, and LPIs, show the same trends (mainly a decrease) between species upon infection. This overall reduction in phospholipid levels is observed in infections featuring strong inflammation, for example, community-acquired pneumonia and correlates with levels of C-reactive protein (34). Furthermore, polar metabolites associated with lipid degradation and carnitine metabolism are similarly decreased in infected animals of both strains. Carnitine-associated metabolites are probably downregulated because of lower food intake and gut metabolism. Crotonobetaine, γ-butyrobetaine, and TMA, the direct precursor of TMAO, are produced in the gut from carnitine (35) as well as the AA derivative 3-indolepropoinate. The strong decreases in sulfated phenolic compounds and their degradation product hippurate are also likely driven by reduced food intake. A similar explanation can be given for the decreases of trigonelline and NAM. Looking at Figure 2*B* (glycerolipid panel) and Figure 4, it seems that glycerolipids and nutritional markers are more decreased in D2, suggesting lower food intake in this strain.

Purine metabolites were downregulated at 3 dpi, which could also be caused by lower nutritional intake or lower *de novo* synthesis. Interesting is the increase of deoxycytidine in both strains, an effect previously observed in lung tissue of B6 under similar conditions (36). Moreover, an 88% spike for this metabolite was seen in B6 at 8 dpi. It was shown that an enzyme involved in the phosphorylation of deoxycytidine, deoxycytidine kinase, is necessary in the development of T and B lymphocytes (37). Therefore, deoxycytidine might play a role in immune response in both strains.

Since all D2 animals died between 5 and 8 dpi, metabolic disease progression could only be described for B6. In general, all detected metabolite classes were maximally decreased between 5 dpi and 8 dpi. Drops in the levels of ACars, FAs, and other lipids are probably because of the exhaustion of lipid reserves. However, upon recovery (from 18 dpi on), most metabolites did not return to their preinfection levels. MCFAs, LCFAs, and their oxidized forms were in general higher at recovery, whereas VLCFAs stayed down with respect to 0 dpi. Differential effects were also observed for ACars. These changes might be caused by altered metabolism because of renewed buildup of energy reserves or to age-related changes in energy expenditure. Increased levels of diet-dependent markers (*e.g.*, trigonelline, crotonobetaine, TMAO, and phenols) indicated a higher food intake after recovery. Increased food intake just prior to recovery might also be the reason for the increase of most AAs, the tyrosine derivative dihydroxyindole and taurine at 8 dpi. Increased AA metabolism would lead to activation of the urea cycle and hence increased acetylornithine and citrulline as observed in B6.

### Interstrain differences in energy metabolism after infection

We argue that most metabolic and interstrain differences during infection are due to lower mitochondrial beta-oxidation and subsequent infection-induced increase of peroxisomal beta-oxidation in D2.

After IAV infection, both strains showed significant weight loss, which was about 5% higher in D2 compared with B6 (Fig. 1). Weight loss could be explained by a decrease in nutritional intake and increased energy expenditure needed for the activation of the immune response (38) and higher breathing effort. To source the extra energy needed, high-energy substrates are released from adipose tissue in the form of FAs, from myocytes as AAs and from autophagic processes. In IAV infection, these catabolic processes are mediated by Toll-like receptors (TLRs), in particular TLR4, after interaction with pathogen-associated molecular patterns (PAMPs) (39). TLR4 is known to trigger lipase activity (40) and insulin resistance (41) in adipocytes and breakdown of myofiber proteins in muscle (42). This leads to increased availability of plasma glucose, production of FAs, and release of amino acids. Since virus titers were about 10 to 100 times higher in D2 (43, 44), it is likely that circulating PAMPs were also higher. This would lead to higher TLR activity and subsequent loss of adipose and muscle tissues, causing higher weight loss in D2 and increased release of FAs and AAs. Moreover, food intake is likely more reduced in D2 compared with B6 during IAV infection, which might lead to activation of pathways for releasing stored energy deposits.

In muscle cells, FAs serve as a direct energy source, mainly through mitochondrial beta-oxidation. In the liver, FAs are transformed to ketogenic bodies and glucose by mitochondrial processes to serve as energy sources elsewhere. However, high levels of MCar in D2 at 3 dpi suggest that MCoA levels are still highly upregulated in this strain during infection. Therefore, in D2 during IAV infection, mitochondrial oxidation of FAs would still be reduced by continuing inhibition of CPT1. This would lead to a reduction in ACar *via* this pathway. However, a strong increase in circulating ACars was observed in D2 plasma. Thus, an alternative pathway is likely responsible for the increased production of ACars.

Interestingly, highly increased ACar levels were detected in patients with influenza-associated encephalopathy (45). This was caused by a thermolabile variant of CPT2 that was rendered nonfunctional upon fever. Through the CPT2 defect, ACars in the mitochondrial matrix were not converted to their CoA esters, which led to impaired mitochondrial beta-oxidation with severe consequences. A thermolabile variant of CPT2 would increase plasma ACar levels, but it would not explain the interstrain decrease in ACar levels in healthy D2 animals.

Another candidate pathway leading to increased ACar levels upon infection in D2 is peroxisome metabolism. These organelles are also capable of producing ACars *via* carnitine O-octanoyltransferase and can compensate for a defective mitochondrial carnitine shuttle (46). Peroxisomes actively take up long- to very long–chain polyunsaturated ACoA *via* the aforementioned ABCD transporters. Moreover, passive transport of nonesterified FAs over the peroxisomal membrane is possible (47). Once in the peroxisomes, the acyl chains are shortened under the formation of AcCoA but not until completion. The shortened ACoA esters are then converted into ACars. The resulting ACars are excreted back into the cytosol where they can serve as fuel for mitochondrial beta-oxidation. In the liver, AcCoA leaves the peroxisome as acetate after hydrolyzation by ACOT12 to add to the cytosolic pool (48). Acetate in the cytosol is either excreted or converted to AcCoA and thus MCoA/MCar (Fig. 6, *A* and *C*). Thus, excessive peroxisomal beta-oxidation could lead to increased cytosolic levels of carnitine esters.

Compatible with increased peroxisomal beta-oxidation, it was found that the transcription of mediator complex subunit 1 (*Med1*, *Pparbp*) was upregulated in lungs of D2 (19) and at the same time identified as a quantitative trait locus for influenza virus resistance (5). This mediator enhances peroxisome proliferation and FA oxidation *via* peroxisome proliferator–activated receptor alpha signaling (49).

Because of the spatial separation of peroxisomes and mitochondria, peroxisomal ACar formation is less efficiently coupled to mitochondria as is the CPT1/CACT/CPT2 shuttle. Therefore, we hypothesize that in D2 during infection, peroxisomal ACars will leak away to the extracellular space instead of entering the mitochondria. This would explain the strongly increased plasma ACar levels in D2. Higher release of FAs from adipose tissue, because of increased TLR4 signaling, could augment this process in D2. On the contrary, mitochondrial beta-oxidation in B6 seems to increase in activity during IAV infection as increased OxFA levels suggest.

Since D2 cannot efficiently utilize FAs, Val might be used to compensate for energy shortages. This AA exhibited a −90% drop in D2 at 3 dpi, whereas B6 levels were stable. Serine and glutamate might be other AAs used in both strains to replenish the TCA cycle. This is supported by transcriptomics data, which clearly show that the transcription of the gene coding for the branched-chain amino acid transporter 1 (*Bcat1*) is higher in D2 compared with B6 at 5 dpi (Fig. 4). Conversely, Ile/Leu was increased in D2 and apparently cannot be efficiently used in anaplerotic reactions during infection.

Moreover, increased peroxisomal hepatic beta-oxidation in D2 might affect purine metabolism. The catalysis of urate to 5HIU by urate oxidase is a peroxisomal process using oxygen. In both species, urate and 5HIU levels were downregulated. However, relative to B6, 5HIU D2 levels were lower, whereas its precursor urate was higher. Since D2 oxygen levels are probably decreased with respect to B6 because of impaired lung function and increased peroxisomal beta-oxidation, less oxygen is available for urate catalysis. The downstream metabolite allantoin was reduced in both species, an effect previously observed in IAV-infected B6 lung tissue (50).

The proposed switch from mitochondrial to peroxisomal FA metabolism might in part explain the heighted vulnerability of D2 animals for IAV infection. Since peroxisomal beta-oxidation produces high amounts of ROS (51), oxidative stress and subsequent cell damage would increase in D2, at least in metabolic tissues like liver and muscles (52).

In lung tissue, another mechanism explaining a poor outcome in D2 might be involved. Higher virus titers in D2 very early (1 dpi) after infection (43) are either related to an impaired initial cellular host defense, an increased replication cycle, or both. While we discuss possible differences in immune response later, a distortion of peroxisomal beta-oxidation in healthy lungs of D2 animals might explain this increase in virus replication. Deficient FA oxidation in alveolar epithelial cells is specifically associated with pathogen-induced acute lung injury (53). Furthermore, it was found that in lung epithelial cells, IAV decreases peroxisomal beta-oxidation and increases peroxisomal ether lipid (plasmalogen) synthesis for viral envelope fabrication. Conversely, induction of peroxisomal beta-oxidation reduced viral replication in a lung-epithelial cell line (54). Moreover, IAV led to a downregulation of carnitine O-octanoyltransferase in airway epithelium (55). Since peroxisomal beta-oxidation of FAs is shut down by IAV in lung epithelial cells, these metabolites can now be used in the synthesis of ether lipids in these organelles. The increased levels of plasma FAs might be a cause of the high initial viral titers observed in D2 since they provide a source for the increased synthesis of ether lipids for viral assembly and thus.

In addition, ACars are pneumotoxic in high concentrations. Accumulation at the epithelial air–fluid interface reduces the effect of pulmonary surfactant, thereby reducing pulmonary compliance. This might lead to increased susceptibility to infections and hypoxia (56). Increased D2 plasma levels of ACars therefore might compromise lung function further.

### Interstrain differences in immune response

Both mitochondria and peroxisomes play an important role in innate antiviral signaling *via* retinoic acid–inducible gene I–like receptor signaling (57, 58). It might be that the compromised peroxisomal–mitochondrial axis in D2 would affect these signaling pathways. It is known that mitochondria are specifically affected by the pathogenic influenza A subtype used in this study through the viral protein PB1-F2. This viral protein translocates to the mitochondrial inner membrane, thereby impairing innate immunity (59). Therefore, we hypothesize that initially compromised mitochondrial and peroxisomal functions, together with specific targeting of these organelles by IAV in lung epithelial cells, work in concert to increase viral replication and spreading in the lung. This would further aggravate the severe disease outcome in D2.

High lethality in D2 compared with B6 was previously observed for tuberculosis, which was contributed to reduced levels of CD103^+^ dendritic cells (DCs) in D2 (60). Specifically, this DC subtype is not affected by IAV and therefore able to induce virus-specific CD8+ T cells (61). Therefore, reduced levels of these DCs in D2 lungs would also impair an antiviral response. In addition, it was found that a ketogenic diet increased γδ T cells in lung tissue and protected against IAV infection. However, as a prerequisite for this protective effect, there should occur a metabolic switch favoring FA oxidation. Increasing the plasma levels of the ketogenic substrate betahydroxybutyric acid alone did not increase expansion of protective T cells (62). Therefore, a reduced T-cell response because of a decreased mitochondrial beta-oxidation might weaken the bridge toward an adaptive immune response in D2.

Finally, it is known that D2 is deficient in complement component 5 (C5, hemolytic complement), a protein involved in both innate and adaptive immune responses. Upon activation of the complement system *via* PAMPs, C5 is cleaved into C5a and C5b. C5b is part of the pore forming membrane attack complex, which causes targeted cells to lyse. C5a interacts with C5a receptors and is involved in chemotaxis, immune cell activation, and reactive oxygen species production. It was found that after crossing B6 with the C5-deficient A/J mouse strain, C5 was associated with higher IAV susceptibility in females than in males (63). However, this sex-dependent effect was not observed for D2 since both genders were equally susceptible (43).

In IAV infection, C5 is implicated in CD8+ T-cell response, and blocking C5a receptor results in the impairment of this response (64). However, in primary infection, this response has an onset at 5 to 7 dpi, which makes it less relevant to our observations. This is corroborated by a study in C5-deficient and sufficient B10.D2 mice where the beneficial effect of C5 on mortality only showed after 7 dpi (65). Moreover, a chimeric D2 model, which had its bone marrow replaced with that of B6, excluded the involvement of C5 in IAV susceptibility (5).

Conversely, C5aR^−/−^ mice on a BALBc background recovered better than the WT mice, and blocking of the C5aR1 receptor had little to no effect on mortality during H1N1 infection in mice (66). Moreover, excessive C5 activation was shown to cause lung injury and thus can have an adverse effect. Targeting C5 with a specific inhibitor reduced excessive inflammatory reactions associated with severe IAV infections. Also, inhibition of C5 had no effect on viral load (67). Therefore, although important in later stages of IAV infection, C5 has a limited or even adverse effect at the onset of infection and thus would be less relevant in explaining the observed phenotype in D2.

## Conclusion and future perspectives

By using an untargeted high-resolution LC–MS approach, we identified metabolic markers that differentiate the susceptible (D2) and resistant (B6) inbred mouse strains at baseline and during H1N1 influenza A viral infection. By combining the observed metabolic responses with prior metabolic, genetic, and transcriptional information, we derived a hypothesis for the increased susceptibility of the D2 strain. In brief, high insulin levels in the insulin-resistant prediabetic D2 strain leads to increased production of MCoA. MCoA inhibits mitochondrial beta-oxidation in healthy and infected D2 animals. In healthy D2 animals, we suggest that this leads to increased FA synthesis and adipose lipid storage. In infected animals, it leads to increased peroxisomal beta-oxidation resulting in increased levels of ROS. Increased FA mobilization through increased TLR4 signaling in D2 aggravates this process. Furthermore, since both mitochondria and peroxisomes are implicated in the antiviral innate immune response, the increased metabolic stress on these organelles by IAV leads to high uncontrolled virus replication and spreading in D2 mice.

It is well known that metabolic disorders like diabetes, insulin resistance, and obesity are risk factors for severe outcomes of influenza A infection. For example, it has been shown that in *bona fide* diabetic mouse models, hyperglycemia in the lung increases the severity of IAV infection by damaging the pulmonary epithelial–endothelial barrier and possibly compromises host defense (68, 69). However, in D2, although showing high insulin levels and some insulin resistance, plasma glucose levels are relatively low. Therefore, the D2 strain could be used as an alternative prediabetic *in vivo* model. This model could be used to get a better understanding in the role of the mitochondrial–peroxisomal axis in host defense. Furthermore, the model could be used to study infection progression under pharmacological therapies. As such, it would be interesting to test the effects of ACC inhibitors or insulin secretion blockers on disease outcome in D2.

Moreover, investigating dietary measures to ameliorate symptoms could be of interest. In this respect, one could think of administration of glucose, amino acids, or short-chain FAs in combination with TLR4 inhibitors. Furthermore, it would be interesting to investigate if some of the observed changes in D2 metabolic profiles during IAV infection are also observed in diabetic or obese mouse models or mouse models with severe outcomes for IAV or other viral infections. In this respect, it would be important in a follow-up study to investigate the difference in metabolomic changes during weight loss in both strains that were either induced by dietary restriction or by IAV infection. At the same time, it would then be important to follow gene transcription in metabolic tissues, such as liver, muscle, and adipose tissue rather than lung as used in this report.

From a translational perspective, it would be interesting to determine if correlations between plasma levels of MCar, ACars, oxidized FAs, and amino acids and disease outcome can also be found in human patients. As such, plasma levels of these metabolites could serve as predictor for high risk or biomarkers for the progression toward severe disease outcome. We expect that our metabolite study represents a stepping stone to protect an ever-growing population with insulin resistance, diabetes, and related syndromes from severe consequences of IAV infection.

## Experimental procedures

### Chemicals

HPLC-grade acetonitrile (ACN), water, and acetic acid were purchased from Fisher Scientific. Ammonium acetate was purchased from Sigma–Aldrich. Chemical standards were purchased from different suppliers. Supplier information can be found in Table S1.

### Mouse husbandry and infections

All animal experiments were approved by an external committee according to the German regulations on animal welfare (permit numbers: 33.9.42502-04-051/09 and 3392 42502-04-13/1234). The protocol and all methods used in these experiments have been reviewed and approved by the Niedersächsisches Landesamt für Verbraucherschutz und Lebensmittelsicherheit (permit numbers: 33.42502-108/06; 33.19-42502-04-18/2922; 33.9.42502-04-051/09; and 33.19–42502-04-13/1234) after consultation with its ethics committee. All methods were performed in accordance with the relevant guidelines and regulations. The study is reported in accordance with the ARRIVE guidelines (70).

C57BL/6J and DBA/2J mice were obtained from Janvier. All mice were maintained under specific pathogen-free conditions and according to the German animal welfare law (BGBl. I S. 1206, 1313 and BGBl. I S. 1934) at the animal facility of the Helmholtz Centre for Infection Research. Breeding and maintenance colonies were kept in individually ventilated cages (Sealsafe Plus GM500; Tecniplast) at a temperature of 22 ± 1 °C, humidity of 55 ± 5%, 75 air exchanges per hour in the cages, and a 14/10-h light/dark cycle with the lights on at 6:00 AM. The maximum caging density was five mice from the same litter and sex starting from weaning. As bedding, aspen wood bedding (Tapvei) was provided. Mice were fed a standardized mouse diet (V1534-300; Ssniff) and provided drinking water ad libitum. All materials, including individually ventilated cages, lids, feeders, bottles, bedding, and water were autoclaved before use. Health status was controlled quarterly by exhaust air dust PCR testing (Environmental Mouse Complete PRIA; Charles River Laboratories) and biannually by serum samples of stock animals (Federation of European Laboratory Animal Science Associations Annual Serology; Charles River Laboratories). Mice were negative for at least all Federation of European Laboratory Animal Science Associations–relevant murine infectious agents.

Mice were allocated to groups by chance; a specific randomization protocol was not used. Mice exhibiting abnormal behavior, posture, or injuries were excluded from the experiments. These criteria were established *a priori*. Treatment was not blinded because infected and noninfected groups had to be kept in separated cages to avoid crossinfection.

The mouse-adapted virus strain influenza A/Puerto Rico/8/34 (H1N1;PR8;PR8_Mun2_INFG_0312) was produced and titrated as described previously (4, 71). Female 10-week-old DBA/2J (D2) or C57BL/6J (B6) mice were anesthetized by intraperitoneal injection with ketamine/xylazine (85% isotonic NaCl solution, 10% ketamine, and 5% xylazine) with doses adjusted to the individual body weight. Mice were then intranasally infected with 20 μl virus solution (2 × 10^3^ focus-forming unit PR8) or mock infected with PBS. Body weight changes were followed for each day after infection. In addition to mice that were found dead, animals with a body weight loss of more than 30% of the starting body weight were euthanized and recorded as dead. For each individual, animal eye blood (75 μl blood to 25 μl heparin) was taken by final bleeding on 0, 1, 3, 5, 8, 18, and 30 dpi for metabolite analysis. Per strain and challenge, five animals were used.

### Sample preparation

Heparin-blood samples were centrifuged 10 min/4 °C/1300*g*, and the supernatant was stored at −70 °C. All collected samples were processed and analyzed at the same time. Stored samples were thawed, and 150 μl ACN/water (50% v/v) was added to 50 μl of supernatant. Samples were shaken at 4 °C at 1400 rpm in a thermomixer and then centrifuged for 15 min at 4 °C and 13,000 rpm. Finally, 175 μl of the supernatant was transferred into a new tube and left 20 min at −70 °C, then placed in a speed vac to evaporate fluid to dryness in approximately 2 h. Pellets were shipped from Germany to Spain on dry ice and resuspended in 100 μl ACN/water (60%/40% v/v) prior to analysis.

### Metabolite analysis

Samples were analyzed on an ultraperformance liquid chromatographic system (Acquity; Waters, Inc) coupled to a time-of-flight mass spectrometer (SYNAPT G2; Waters, Inc). A 2.1 × 100 mm, 1.7 μm BEH HILIC (hydrophilic interaction) column (Waters, Inc), thermostated at 40 °C, was used for sample separation. Mobile phase solvent A (aqueous phase) contained 98.5% water, 1% ACN, 0.5% acetic acid, and 5 mM ammonium acetate, whereas solvent B (organic phase) contained 1% water, 98.5% ACN, 0.5% acetic acid, and 5 mM ammonium acetate.

Separation of the analytes was performed with the following gradient: from 5% A to 15% A in 2 min in a linear gradient, from 15% A to 99.9% A in 4.5 min in a curved gradient (#9; defined by Waters MassLynx software), constant at 99.9% A for 1.5 min, back to 5% A in 0.2 min, and constant at 5% A for 1.8 min. The flow rate was 0.5 ml. All samples were injected randomly with an injection volume of 2 μl. After every five injections, a quality control sample was injected.

The MS was operated in positive and negative ESI mode as described previously (72). The sampling cone voltage was set to 25 V, extraction cone voltage to 5 V, and capillary voltage to 500 V for both polarities. Source temperature was set to 120 °C and capillary temperature to 450 °C. The flow of the cone and desolvation gas (both nitrogen) were set to 5 and 800 l/h, respectively. Scan time was set to 0.2 scans/s covering a range from 50 to 1200 Da. A 2 ng/ml Leu-enkephalin solution was infused at 10 μl/min and used for a lock mass, which was measured each 15 s for 0.5 s, and peaks were automatically corrected for deviations. The MS was tuned to a resolution of 20.000 full-width half-maximum at the *m/z* of the lock mass (556.1227) with an intensity of 1 × 10^5^ counts.

### Data treatment and analysis

For clarity, we will start this section with some definitions that will be used throughout the article. We make a difference between features and metabolites. An *LCMS feature* is defined as a unique mass-to-charge/retention time pair, that is, a unique code based on raw data. An LCMS feature is not chemically identified yet and can be endogenous or exogenous of nature. If the LCMS feature is exogenous, it is referred to as *noise*, if it is endogenous it is referred to simply as *feature*. Features are metabolites or related to metabolites, that is, LCMS adducts or fragments of metabolites. Features are raised to metabolite status and assigned proper names after the identification process.

**Figure 8:**
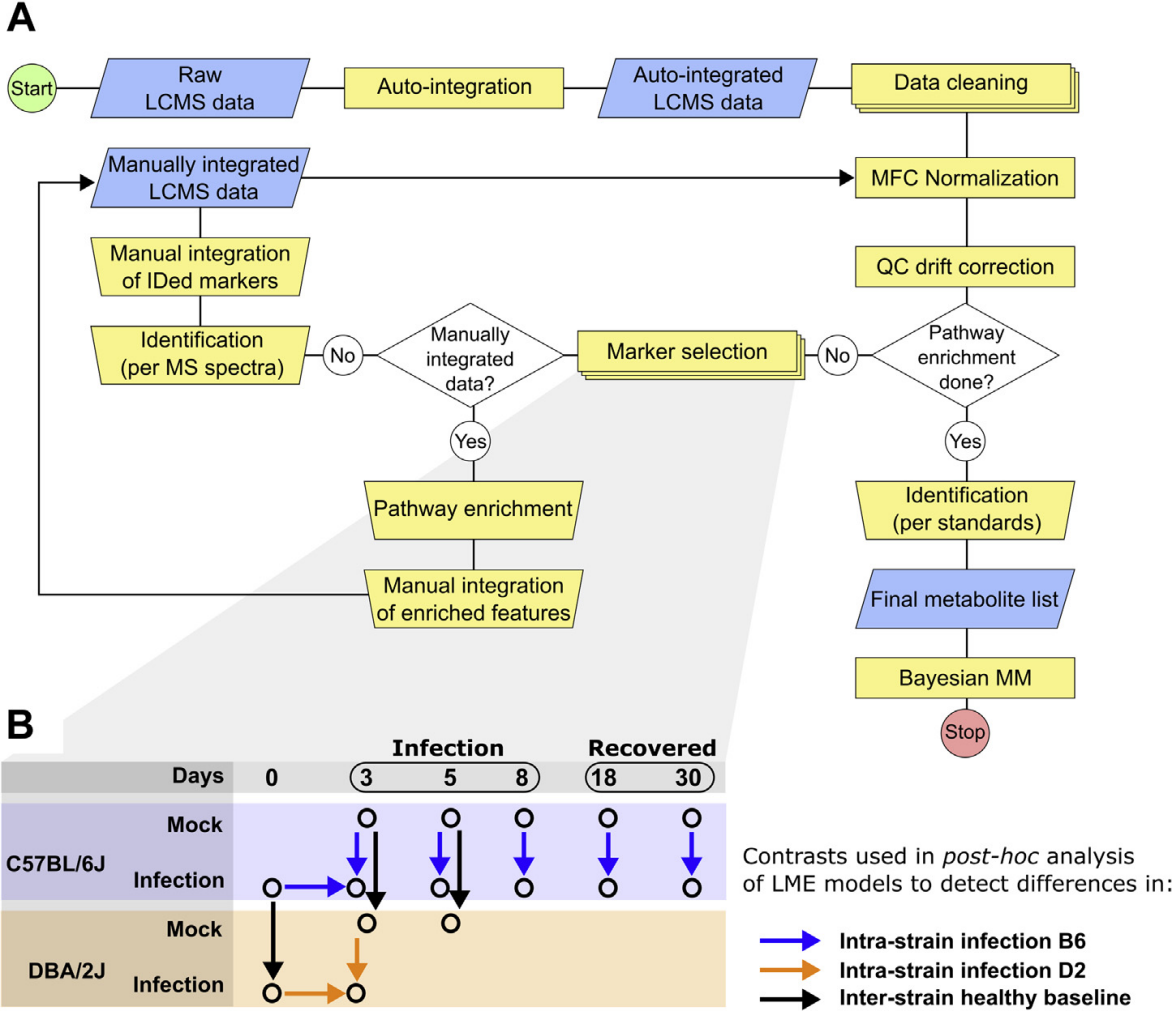
**Data processing pipeline.** (*A*) and contrasts used in the post hoc test to select relevant markers with a linear mixed-effects (LME) model (*B*). MFC, median fold change; MM, multilevel model; QC, quality control.

The data analysis pipeline is depicted in Figure 8. In short, peaks in extracted ion chromatograms were autointegrated with MarkerLynx software (Waters, Inc) in order to obtain LCMS features. Custom and publicly available R scripts were used for further analysis (https://github.com/smvanliempd/IAVmetabolism). Noise was cleaned from the datasets, and remaining features were adjusted for differences in sampled material (median fold change normalization (73, 74)) and drift during the LCMS run (quality control correction). Per feature, the adjusted signals were log standardized and compared between sample groups with a LME model and subsequent post hoc analysis. The sample groups (*i.e.*, strain × time × challenge) were taken as fixed effects, whereas animals were taken as nested random effects.

LME models of features with *p* values smaller than 0.001 were selected and subsequently subjected to *post hoc* analysis on relevant contrasts (Fig. 8*B*). Next, features that showed interstrain or intrastrain differences in the *post hoc* test (at least one adjusted *p* value < 0.05) were selected. If possible, these features were identified by accurate mass, isotope patterns, and in-source fragmentation ions (75) with the aid of HMDB and METLIN metabolite databases. Because of possible autointegration errors, selected features were manually reintegrated (QuanLynx software; Waters, Inc) and again subjected to data adjustment and statistical filtering (post hoc–adjusted *p* value < 0.01). The resulting metabolite set was enriched in an iterative process by mapping the previously identified members to canonical pathways (Kyoto Encyclopedia of Genes and Genomes) and looking for their nearest neighbors in the raw or autointegrated data. For initially selected acyl-containing metabolites like ACars, FAs, or phospholipids, data were mined for members of the same family with different acyl chains and desaturations. The raw data were subsequently mined in order to find, reintegrate, and include these neighbors. Where possible, after pathway enrichment, the putatively identified features were confirmed with chemical standards.

Metabolites of the final set were modeled with a Bayesian multilevel model in the probabilistic modeling language Stan. Posterior distributions of the percent change (%Δ) between relevant (pooled) sample groups were obtained with samples from the Markov-chain Monte Carlo process. The healthy interstrain %Δ is calculated by calculating the relative difference between strains using the pooled posterior samples of 0 dpi, mock-infected 3 dpi, and mock-infected 5 dpi, taking B6 as baseline. As such, for healthy interstrain comparisons, negative %Δ values represent lower levels of a particular metabolite in D2 with respect to B6. For the intrastrain %Δ, for a particular strain, the sample groups in the infected arm were individually compared with 0 dpi. Therefore, negative values for intrastrain %Δ reflect a decrease in metabolite levels during infection compared with 0 dpi.

The %Δ distributions are represented as the 50% percentile in the 50% (25% to 75%) and 90% (5% to 95%) percentile intervals. The 50% percentile of the %Δ distributions represents the most likely value for %Δ based on data, prior probability, and likelihood. Note that Bayesian models do not return *p* values, but the posterior distributions contain (unlike classical confidence intervals) the most likely value of the calculated metric. The posterior distributions were used to infer metabolic differences between strains in healthy and infected states.

## Data availability

Data that were used in this study are available at the following github repository: https://github.com/smvanliempd/IAVmetabolism.

## Supporting information

This article contains supporting information.

## Conflict of interest

The authors declare that they have no conflicts of interest with the contents of this article.
