# Supplementary material for: Impaired beta-oxidation increases vulnerability to influenza A infection
Source: J Biol Chem. 2021 Oct 9;297(5):101298. doi: 10.1016/j.jbc.2021.101298 (PMC8564733; doi:10.1016/j.jbc.2021.101298)
Supplement: Figure S1 [file mmc1.pdf]

Impaired beta-oxidation and increased vulnerability to Influenza A infection in a diabetic mouse model.

Sebastiaan van Liempd[1], Diana Cabrera[1], Carolin Pilzner[2], Heike Kollmus[2], Klaus Schughart[2,3,4], Juan M. Falcón-Pérez[1,3]

1. Metabolomics Platform CIC bioGUNE-BRTA, Derio, Spain

2. Department of Infection Genetics, Helmholtz Centre for Infection Research, Braunschweig, Germany.

3. University of Veterinary Medicine Hannover, Hannover, Germany.

4. Department of Microbiology, Immunology and Biochemistry, University of Tennessee Health Science Center, Memphis, TN, USA.

5. IKERBASQUE, Basque Foundation for Science, Bilbao, Spain

Corresponding author:

Sebastiaan van Liempd

CIC bioGUNE, Metabolomics Platform

Parque Tecnológico de Vizcaya Ed. 800

48160, Derio, Bizkaia, Spain

Email: smvanliempd@cicbiogune.es

Figure S1) Analysis pipeline with amount of features and metabolites remaining after each step.

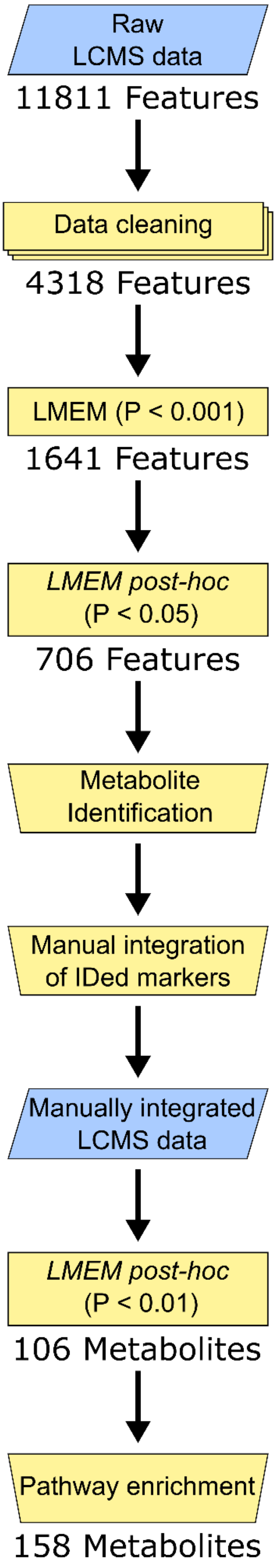

Raw LCMS data
11811 Features
Data cleaning
4318 Features
LMEM (P < 0.001)
1641 Features
LMEM post-hoc (P < 0.05)
706 Features
Metabolite Identification
Manual integration of IDed markers
Manually integrated LCMS data
LMEM post-hoc (P < 0.01)
106 Metabolites
Pathway enrichment
158 Metabolites
