# Supplementary material for: Impaired beta-oxidation increases vulnerability to influenza A infection
Source: J Biol Chem. 2021 Oct 9;297(5):101298. doi: 10.1016/j.jbc.2021.101298 (PMC8564733; doi:10.1016/j.jbc.2021.101298)
Supplement: Figure S2 [file mmc2.pdf]

Impaired beta-oxidation and increased vulnerability to Influenza A infection in a diabetic mouse model.

Sebastiaan van Liempd[1], Diana Cabrera[1], Carolin Pilzner[2], Heike Kollmus[2], Klaus Schughart[2,3,4], Juan M. Falcón-Pérez[1,3]

1. Metabolomics Platform CIC bioGUNE-BRTA, Derio, Spain

2. Department of Infection Genetics, Helmholtz Centre for Infection Research, Braunschweig, Germany.

3. University of Veterinary Medicine Hannover, Hannover, Germany.

4. Department of Microbiology, Immunology and Biochemistry, University of Tennessee Health Science Center, Memphis, TN, USA.

5. IKERBASQUE, Basque Foundation for Science, Bilbao, Spain

Corresponding author:

Sebastiaan van Liempd

CIC bioGUNE, Metabolomics Platform

Parque Tecnológico de Vizcaya Ed. 800

48160, Derio, Bizkaia, Spain

Email: smvanliempd@cicbiogune.es

Figure S2, Raw data (open circles) per challenge and strain for each metabolite. Triangles indicate the median value of the modelled posterior distribution for the mean value of the response. The vertical bars represent the 50% and 90% posterior density intervals.

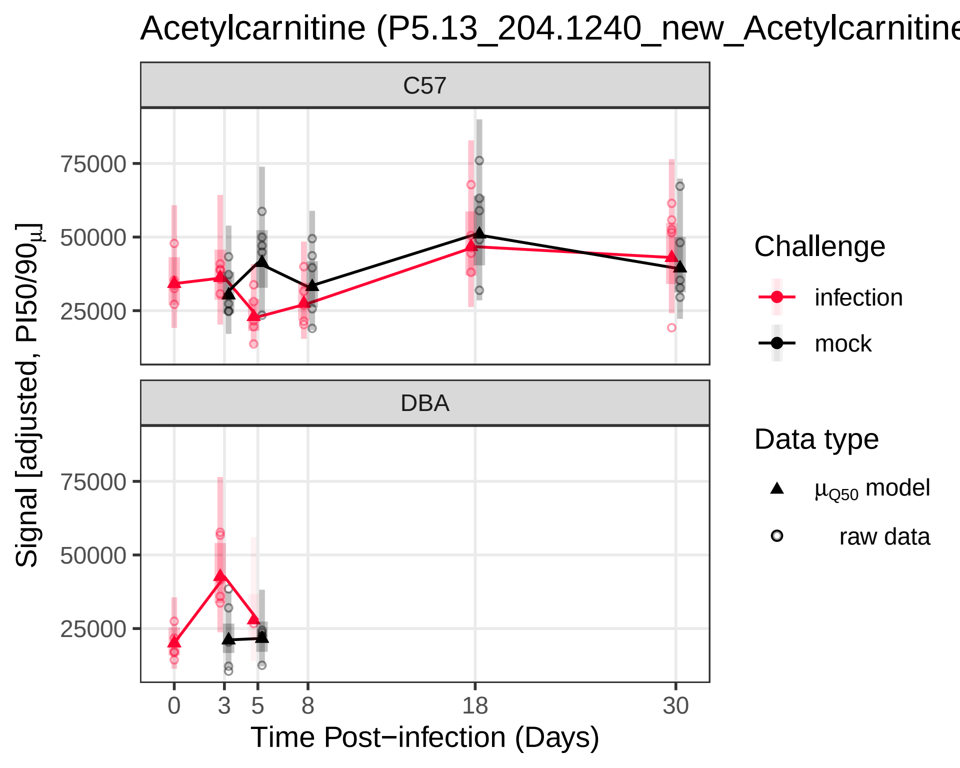

Acetylcarnitine (P5.13_204.1240_new_Acetylcarnitine
C57
DBA
75000
50000
25000
Signal [adjusted, PI50/90μ]
0
3
5
8
18
30
Time Post−infection (Days)
Challenge
infection
mock
Data type
μQ50 model
raw data

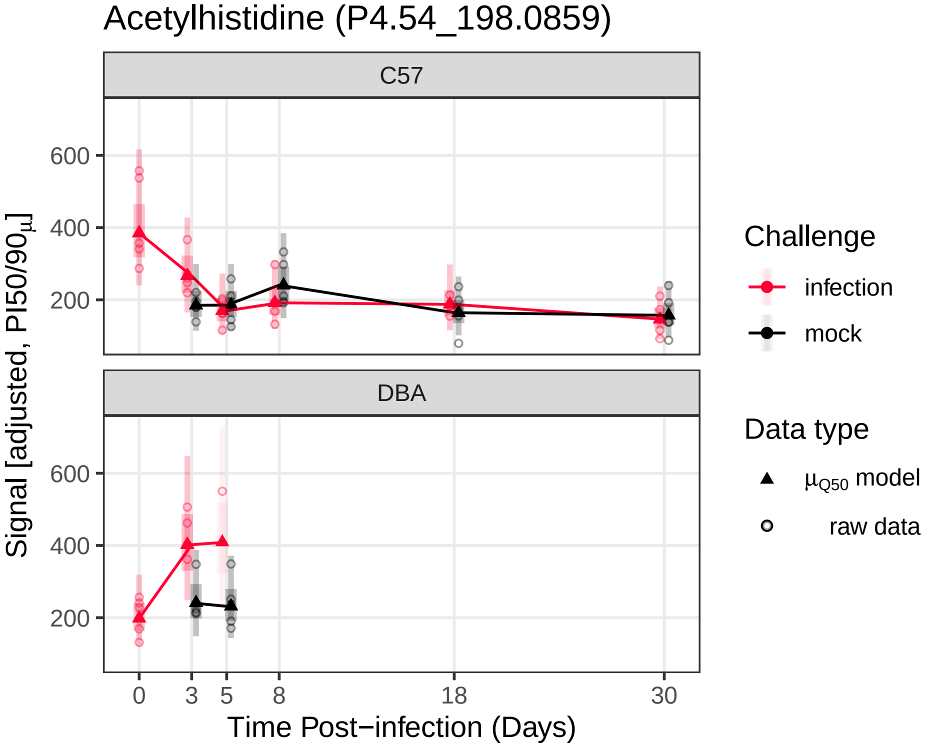
Acetylhistidine (P4.54_198.0859)
C57
DBA
600
400
200
Signal [adjusted, PI50/90μ]
0
3
5
8
18
30
Time Post−infection (Days)
Challenge
infection
mock
Data type
μQ50 model
raw data

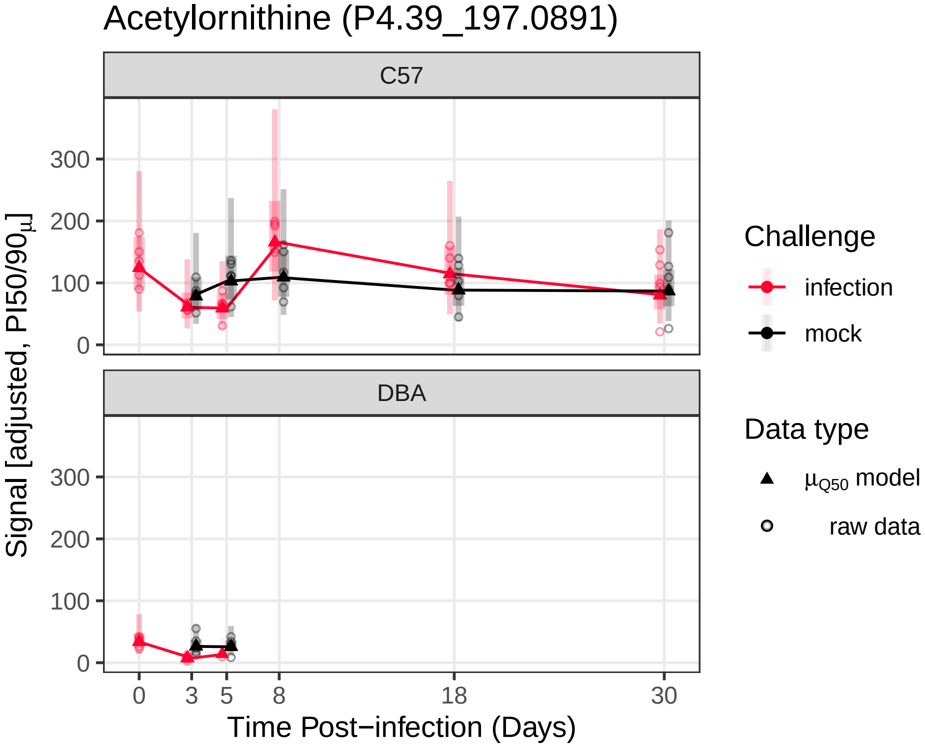
Acetylornithine (P4.39_197.0891)
C57
DBA
300
200
100
0
Signal [adjusted, PI50/90$_{\mu}$]
0
3
5
8
18
30
Time Post−infection (Days)
Challenge
infection
mock
Data type
μ_{Q50} model
raw data

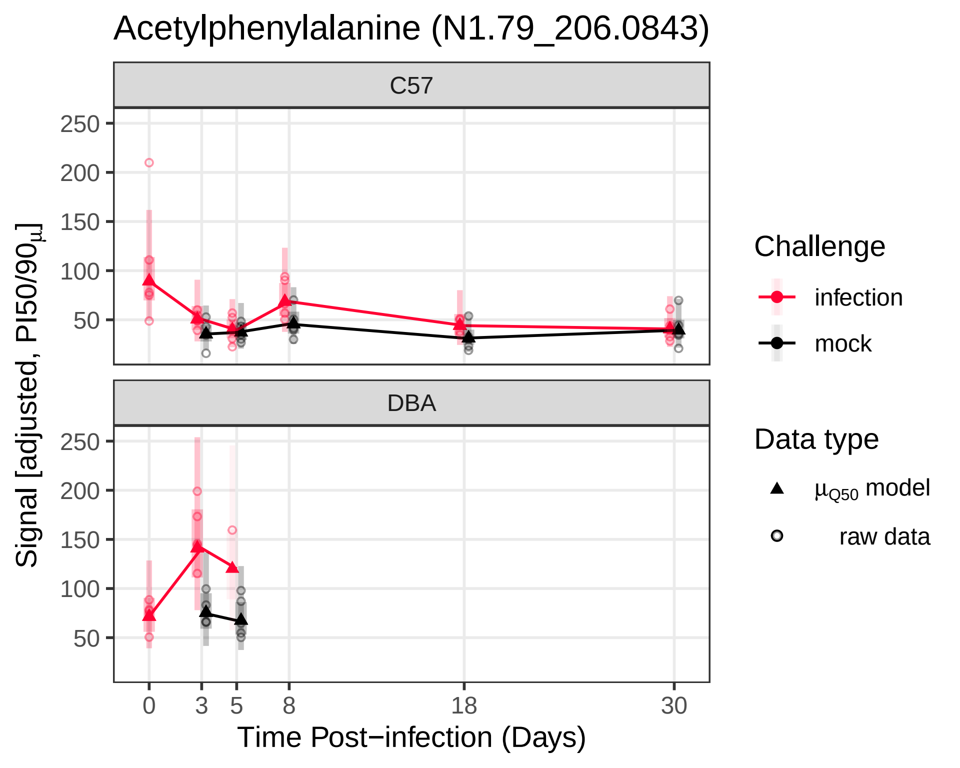
Acetylphenylalanine (N1.79_206.0843)
C57
DBA
250
200
150
100
50
Signal [adjusted, PI50/90$_{\mu}$]
0
3
5
8
18
30
Time Post−infection (Days)
Challenge
infection
mock
Data type
μ_{Q50} model
raw data

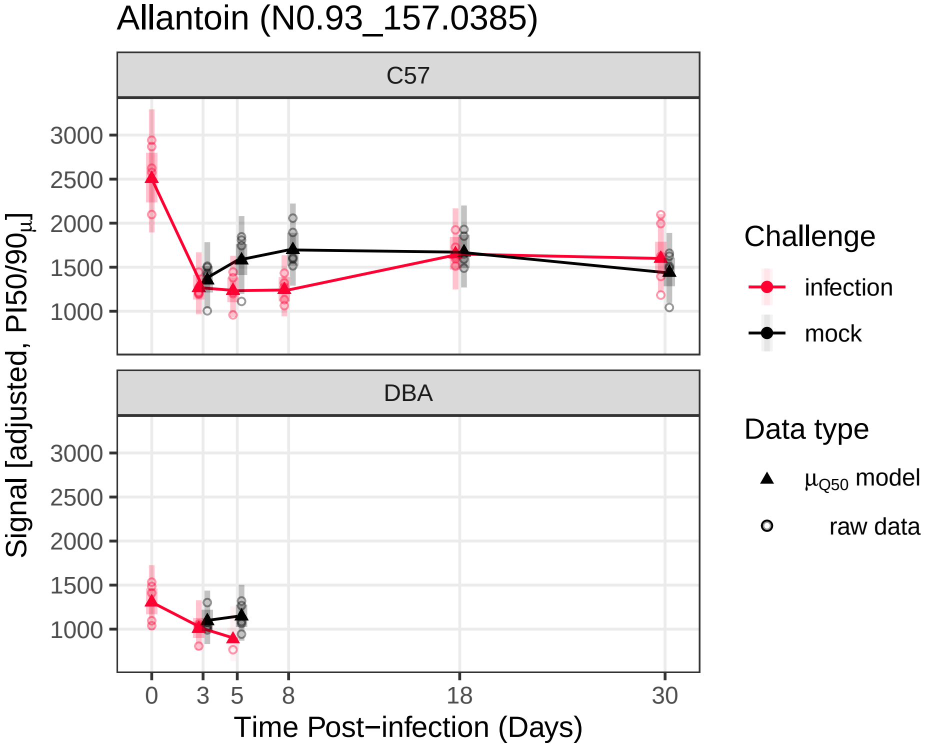

Allantoin (N0.93_157.0385)
C57
DBA
3000
2500
2000
1500
1000
Signal [adjusted, PI50/90μ]
0
3
5
8
18
30
Time Post−infection (Days)
Challenge
infection
mock
Data type
μQ50 model
raw data

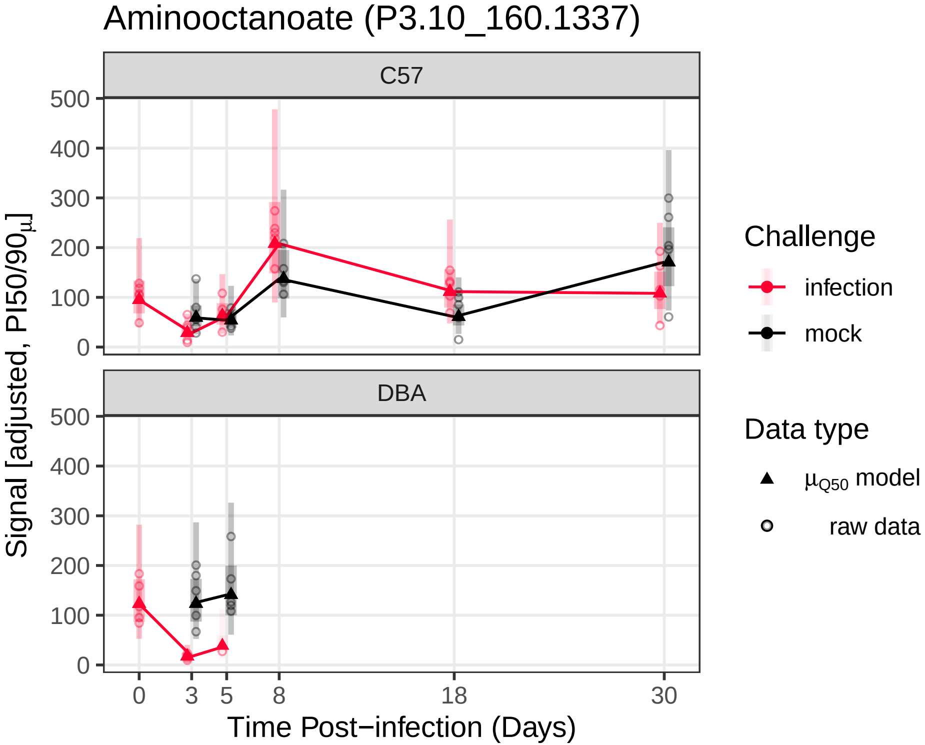

Aminooctanoate (P3.10_160.1337)
C57
DBA
500
400
300
200
100
0
0
3
5
8
18
30
Time Post−infection (Days)
Signal [adjusted, PI50/90μ]
Challenge
infection
mock
Data type
μQ50 model
raw data

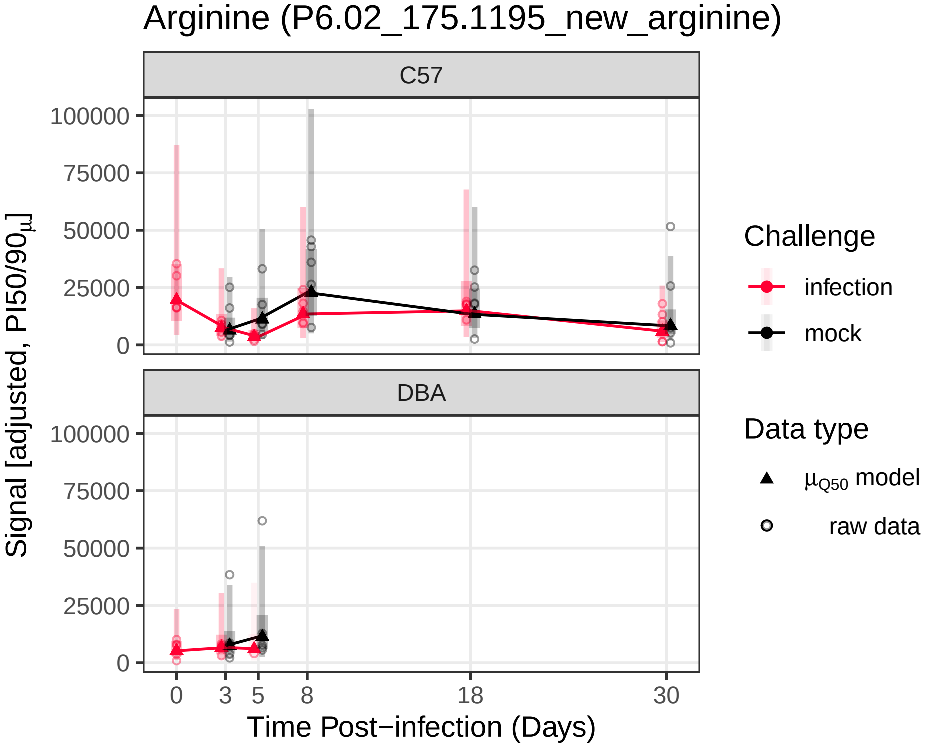

Arginine (P6.02_175.1195_new_arginine)
C57
DBA
100000
75000
50000
25000
0
Signal [adjusted, PI50/90$_{\mu}$]
0
3
5
8
18
30
Time Post−infection (Days)
Challenge
infection
mock
Data type
μ_{Q50} model
raw data

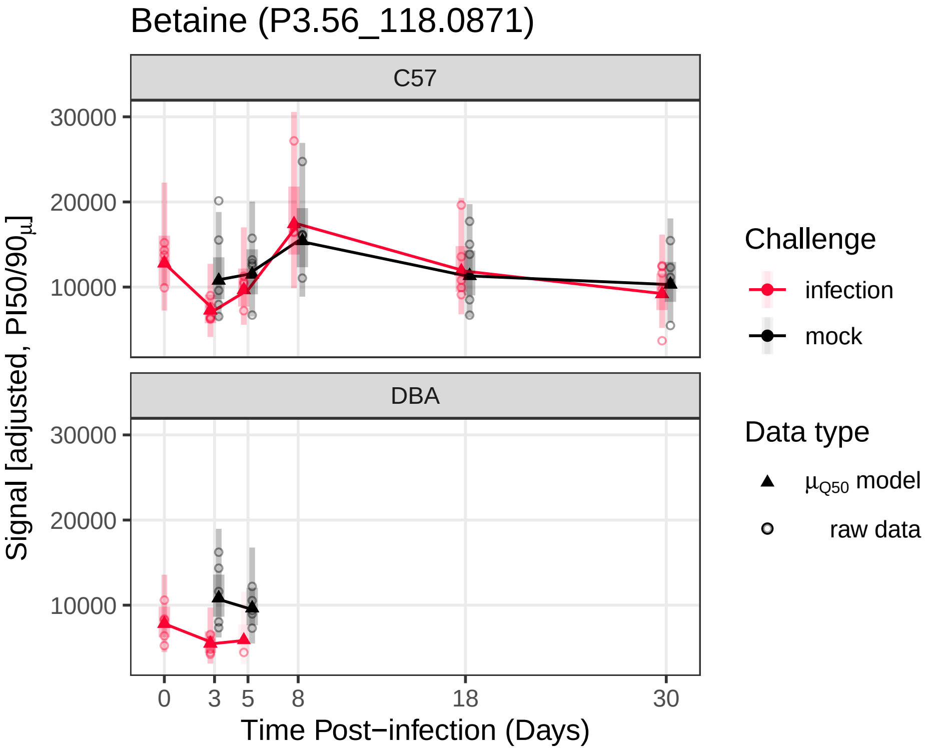

Betaine (P3.56_118.0871)
C57
DBA
30000
20000
10000
0
3
5
8
18
30
Signal [adjusted, PI50/90μ]
Time Post−infection (Days)
Challenge
infection
mock
Data type
μQ50 model
raw data

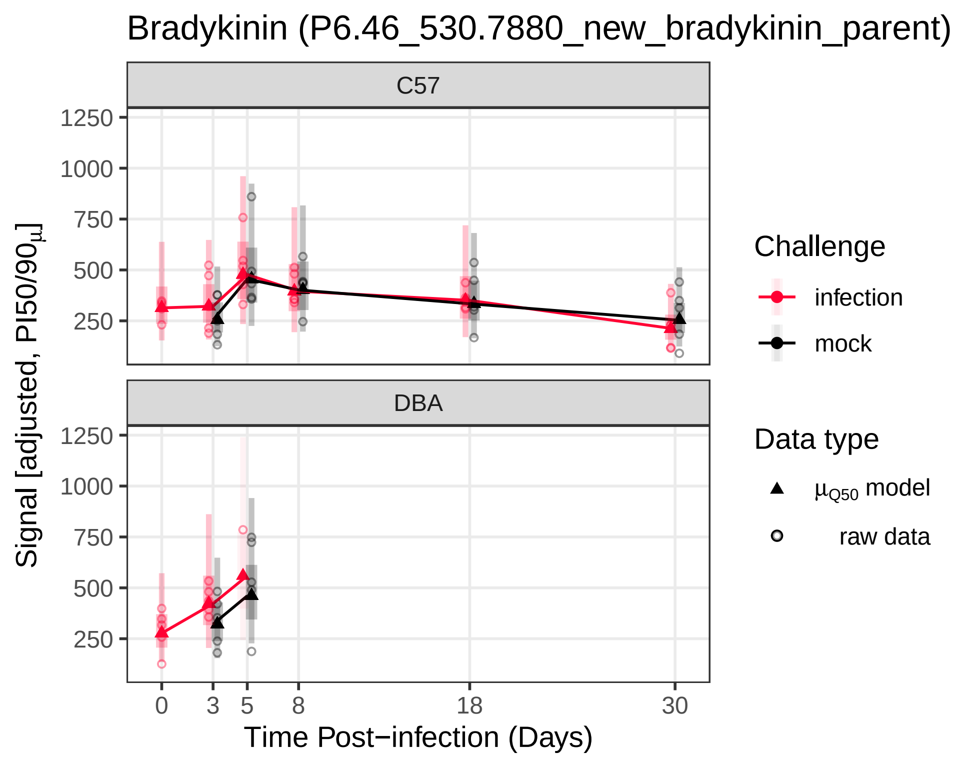

Bradykinin (P6.46_530.7880_new_bradykinin_parent)
C57
DBA
1250
1000
750
500
250
Signal [adjusted, PI50/90$_{\mu}$]
0
3
5
8
18
30
Time Post−infection (Days)
Challenge
infection
mock
Data type
μ_{Q50} model
raw data

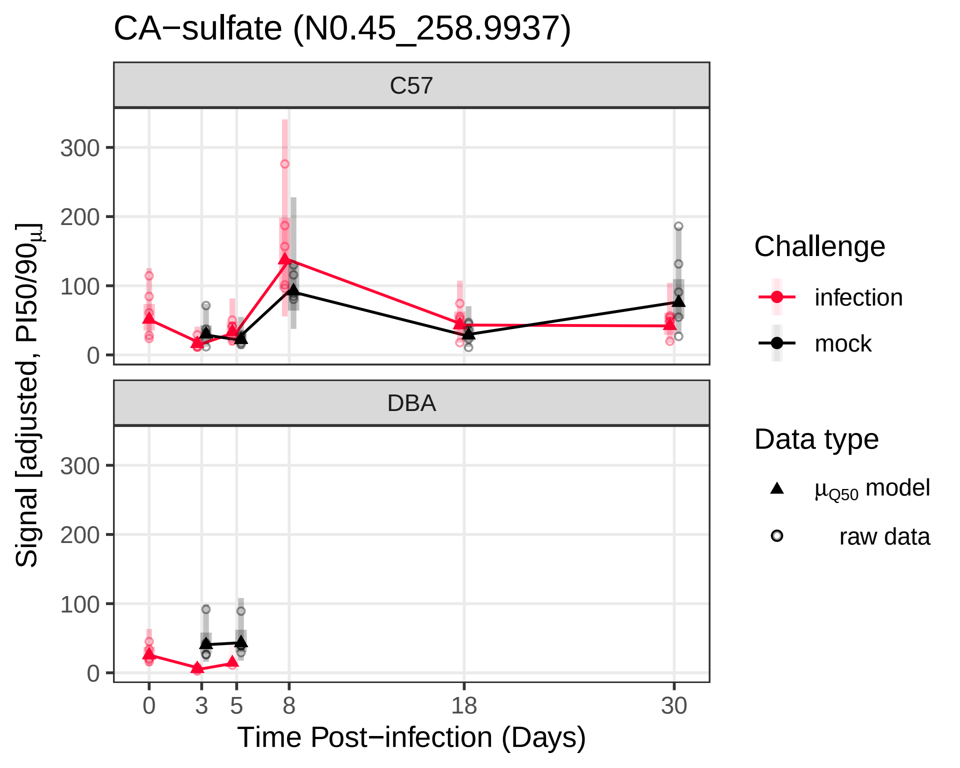

CA−sulfate (N0.45_258.9937)
C57
DBA
Signal [adjusted, PI50/90μ]
300
200
100
0
Time Post−infection (Days)
0
3
5
8
18
30
Challenge
infection
mock
Data type
μQ50 model
raw data

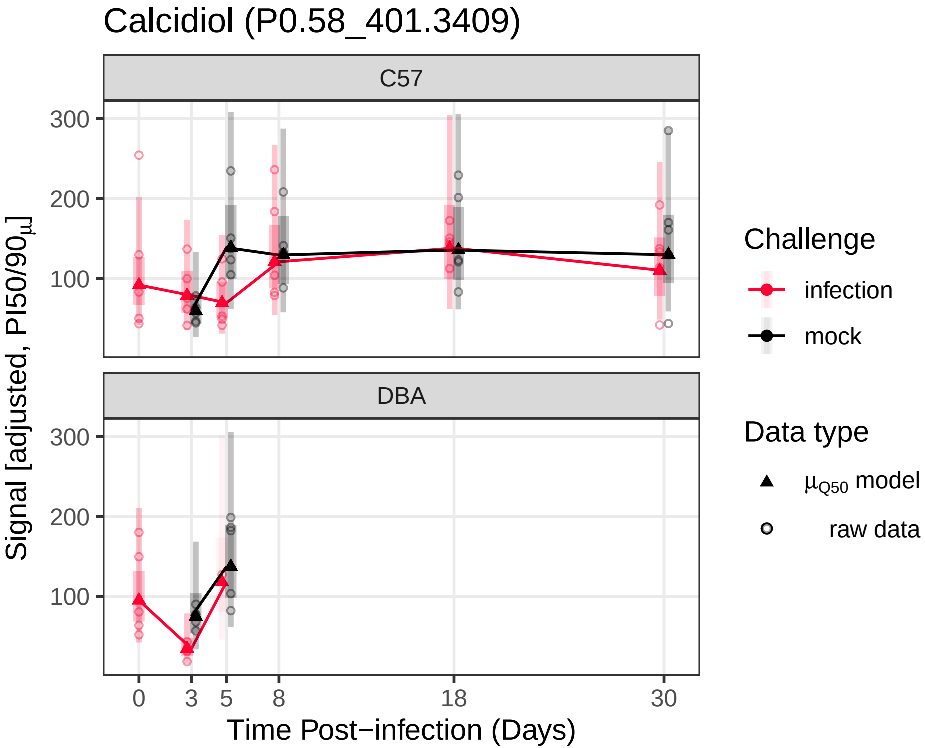
Calcidiol (P0.58_401.3409)
C57
DBA
300
200
100
Signal [adjusted, PI50/90μ]
0
3
5
8
18
30
Time Post−infection (Days)
Challenge
infection
mock
Data type
μQ50 model
raw data

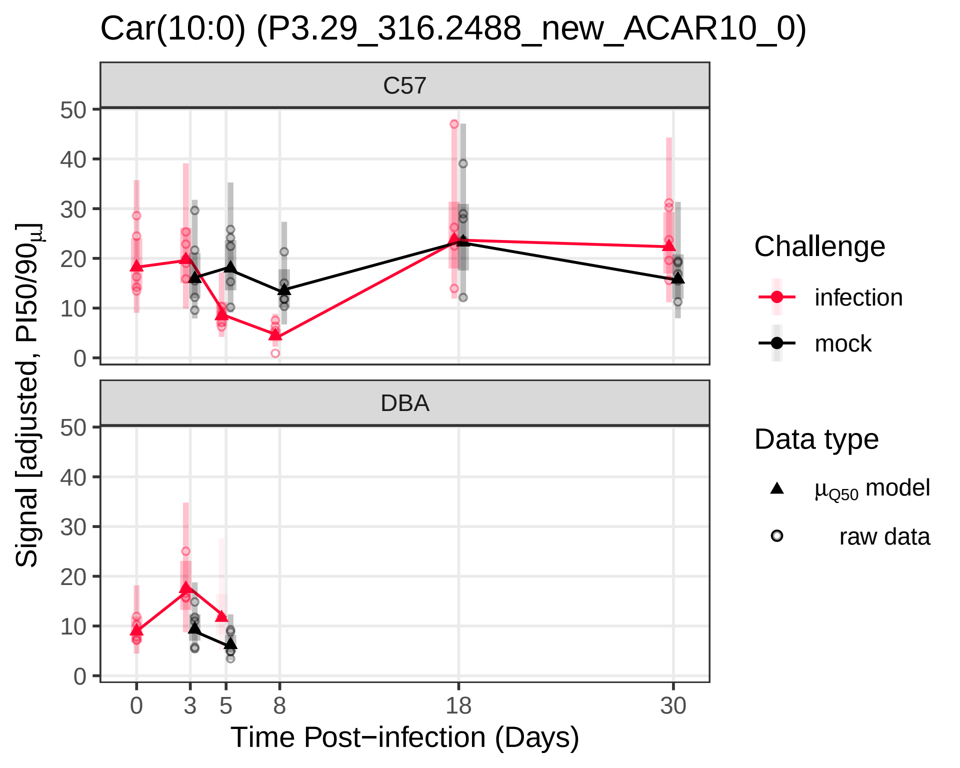

Car(10:0) (P3.29_316.2488_new_ACAR10_0)
C57
DBA
Signal [adjusted, PI50/90μ]
Time Post−infection (Days)
0
3
5
8
18
30
0
10
20
30
40
50
Challenge
infection
mock
Data type
μQ50 model
raw data

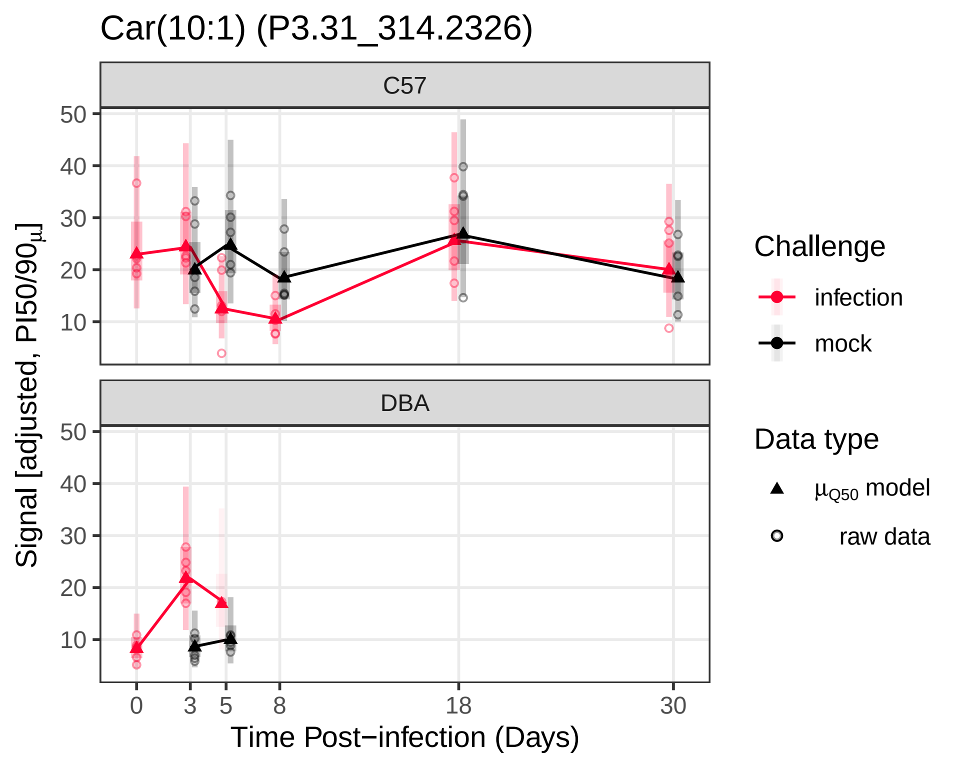
Car(10:1) (P3.31_314.2326)
C57
DBA
50
40
30
20
10
0
3
5
8
18
30
Signal [adjusted, PI50/90$_\mu$]
Time Post−infection (Days)
Challenge
infection
mock
Data type
μ_{Q50} model
raw data

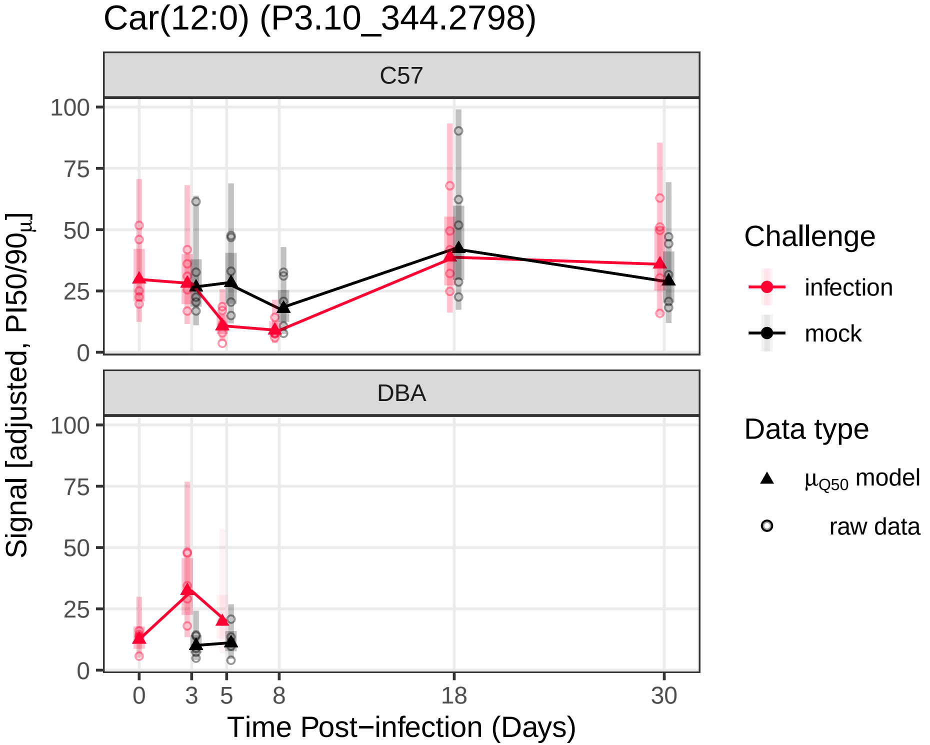
Car(12:0) (P3.10_344.2798)
C57
DBA
Signal [adjusted, PI50/90μ]
Time Post−infection (Days)
0
3
5
8
18
30
0
25
50
75
100
Challenge
infection
mock
Data type
μQ50 model
raw data

Car(14:0/O) (P3.58_388.3040)

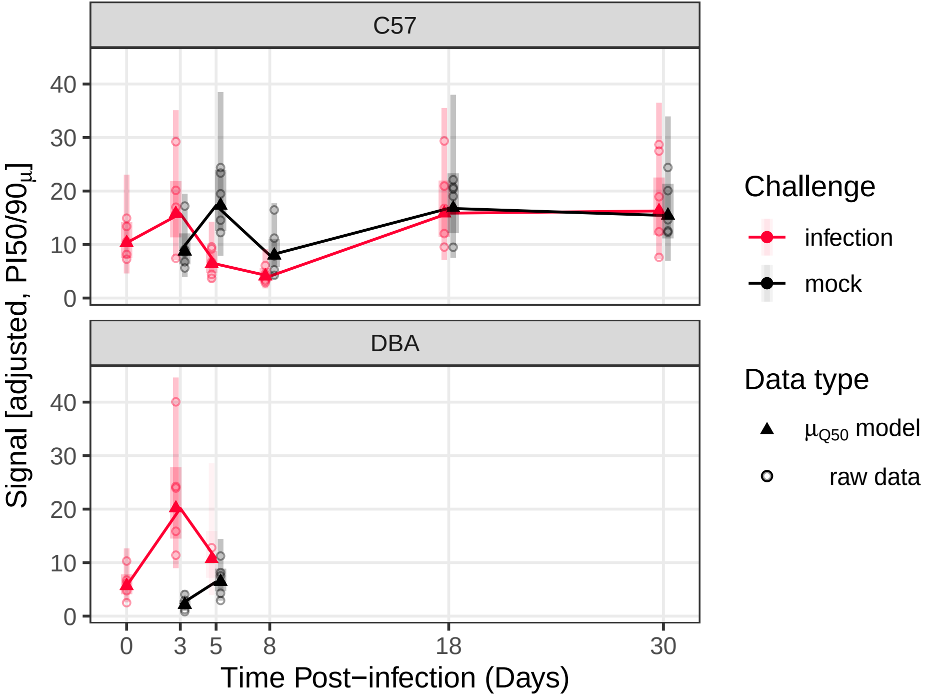

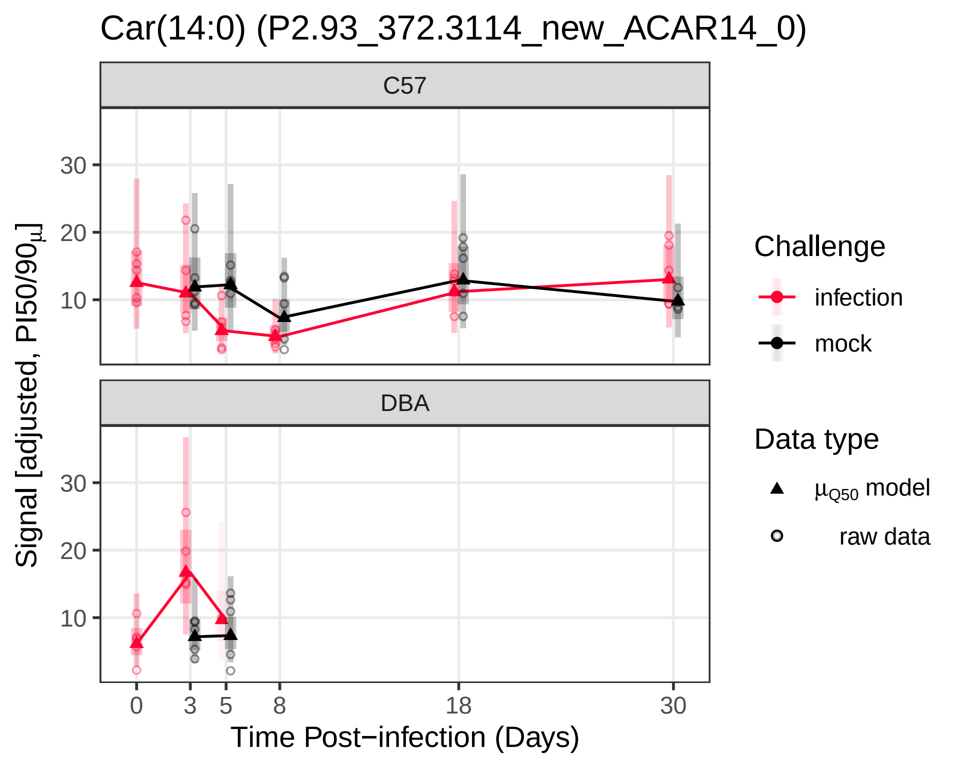
Car(14:0) (P2.93_372.3114_new_ACAR14_0)
C57
DBA
Signal [adjusted, PI50/90μ]
Time Post−infection (Days)
0
3
5
8
18
30
10
20
30
Challenge
infection
mock
Data type
μQ50 model
raw data

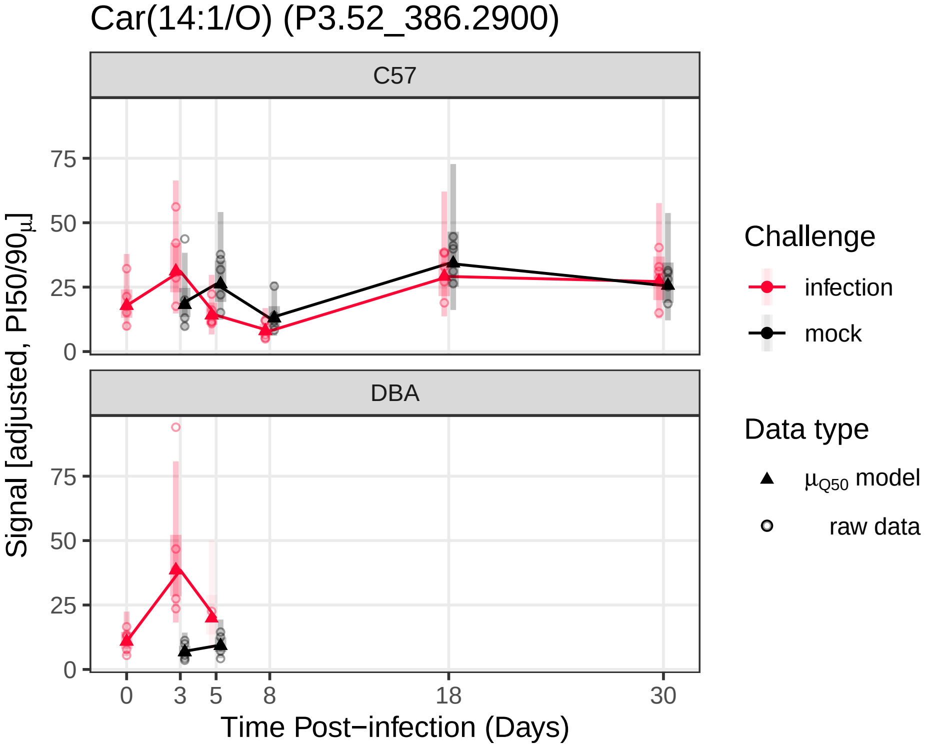
Car(14:1/O) (P3.52_386.2900)
C57
DBA
Signal [adjusted, PI50/90μ]
0
25
50
75
0
3
5
8
18
30
Time Post−infection (Days)
Challenge
infection
mock
Data type
μQ50 model
raw data

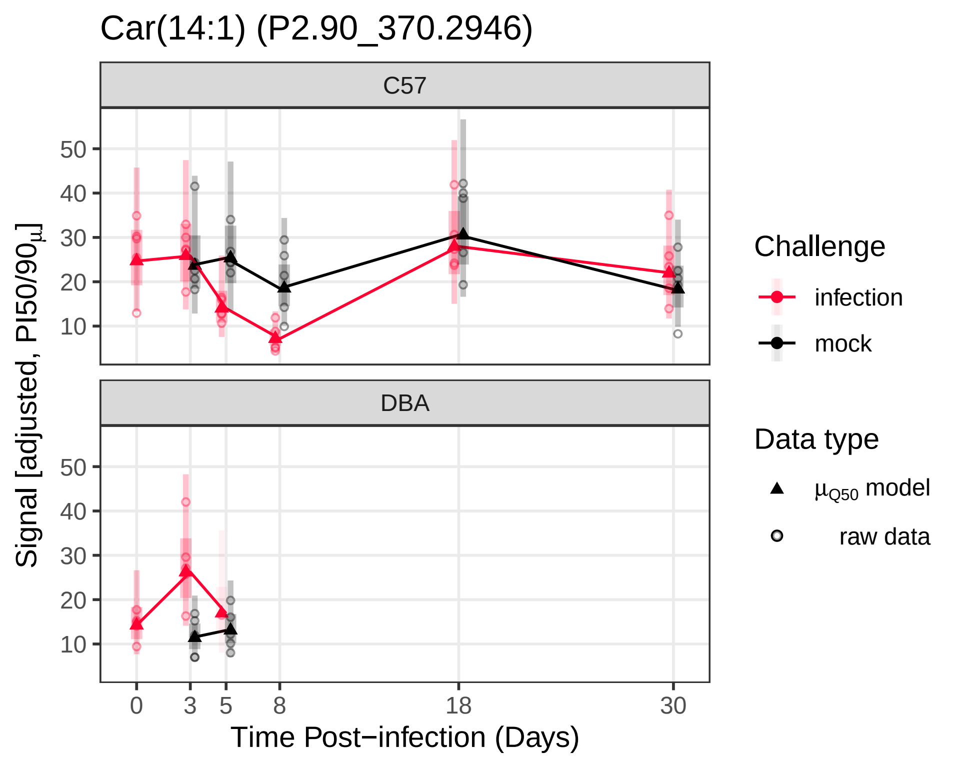
Car(14:1) (P2.90_370.2946)
C57
DBA
50
40
30
20
10
Signal [adjusted, PI50/90μ]
0
3
5
8
18
30
Time Post−infection (Days)
Challenge
infection
mock
Data type
μQ50 model
raw data

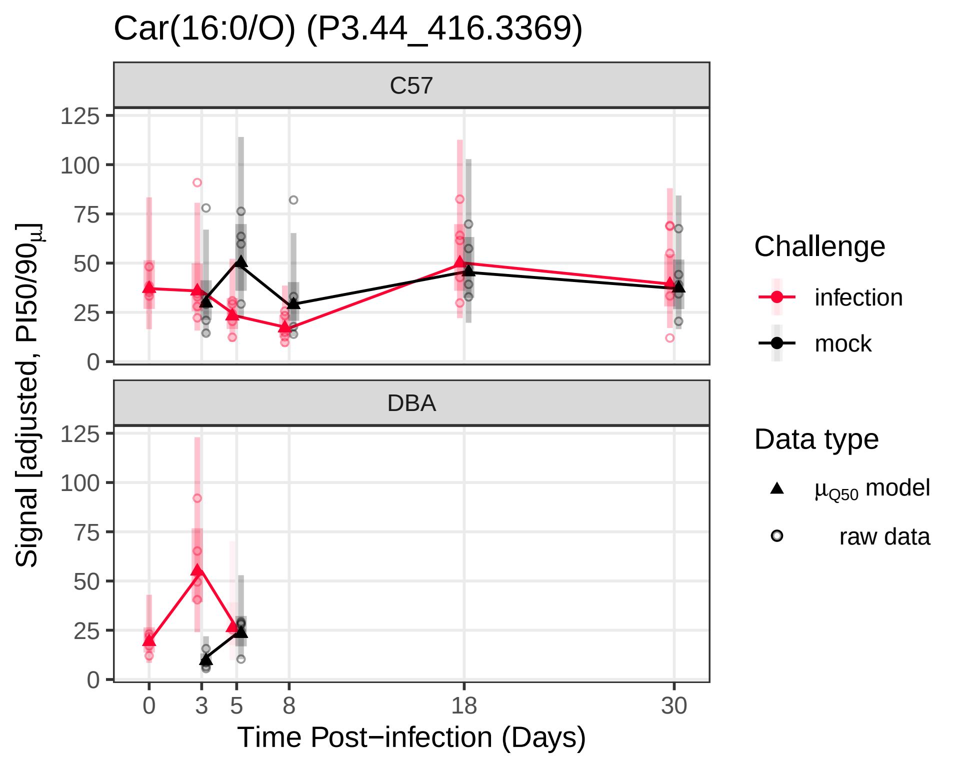
Car(16:0/O) (P3.44_416.3369)
C57
DBA
Signal [adjusted, PI50/90μ]
125
100
75
50
25
0
0
3
5
8
18
30
Time Post−infection (Days)
Challenge
infection
mock
Data type
μQ50 model
raw data

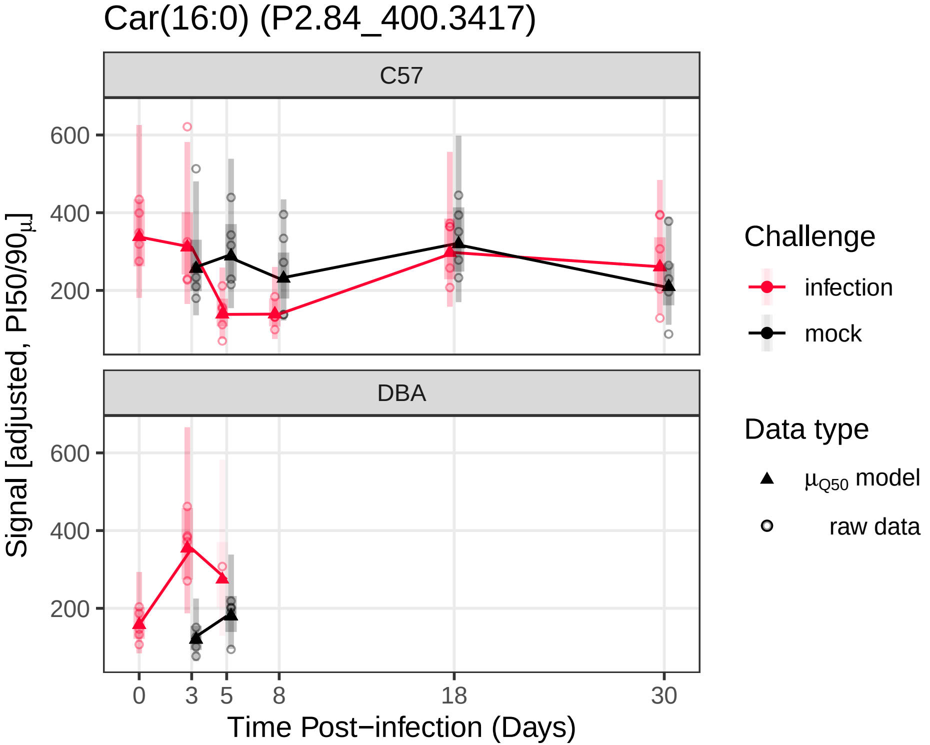
Car(16:0) (P2.84_400.3417)
C57
DBA
600
400
200
Signal [adjusted, PI50/90μ]
0
3
5
8
18
30
Time Post−infection (Days)
Challenge
infection
mock
Data type
μQ50 model
raw data

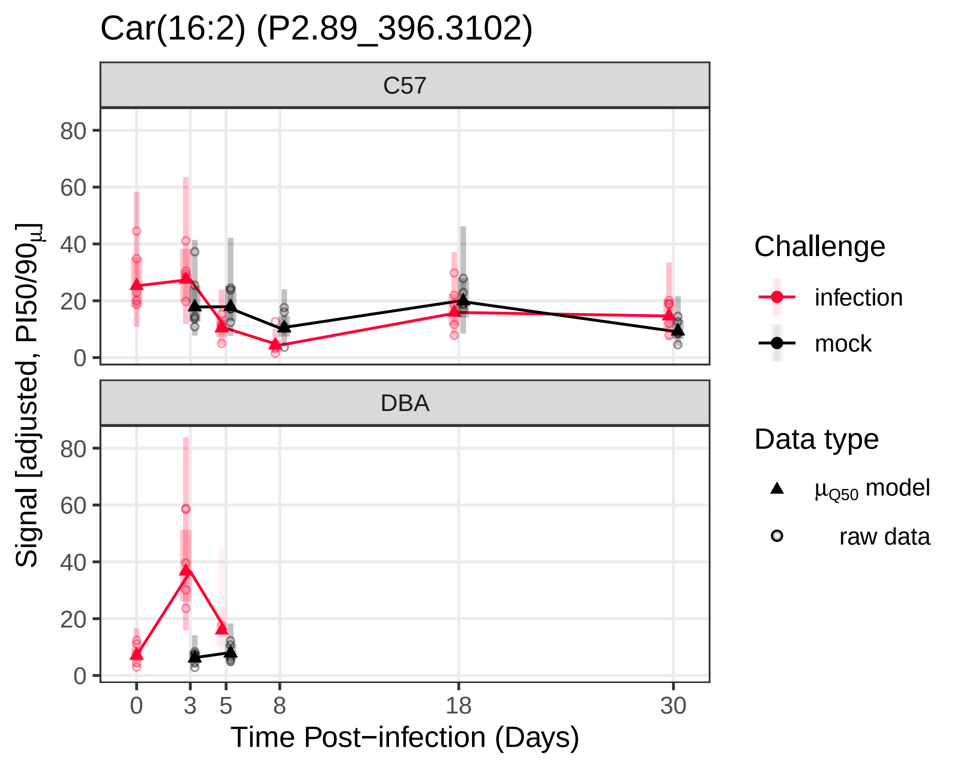

Car(16:2) (P2.89_396.3102)
C57
DBA
Signal [adjusted, PI50/90μ]
80
60
40
20
0
0
3
5
8
18
30
Time Post−infection (Days)
Challenge
infection
mock
Data type
μQ50 model
raw data

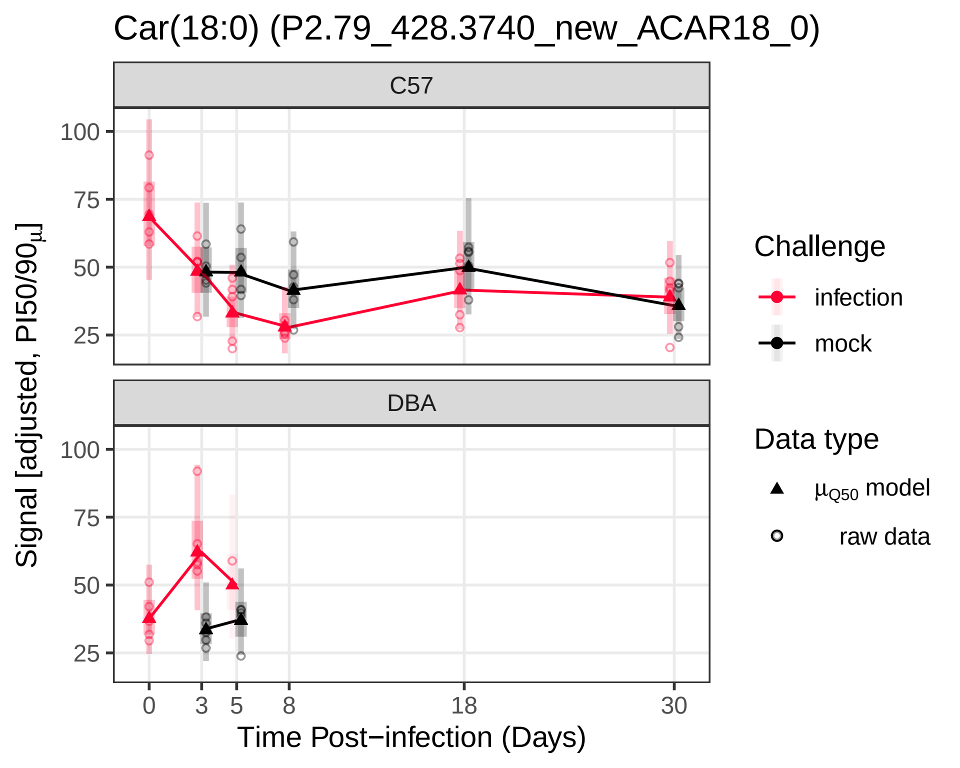

Car(18:0) (P2.79_428.3740_new_ACAR18_0)
C57
DBA
100
75
50
25
0
3
5
8
18
30
Signal [adjusted, PI50/90$_{\mu}$]
Time Post−infection (Days)
Challenge
infection
mock
Data type
μ_{Q50} model
raw data

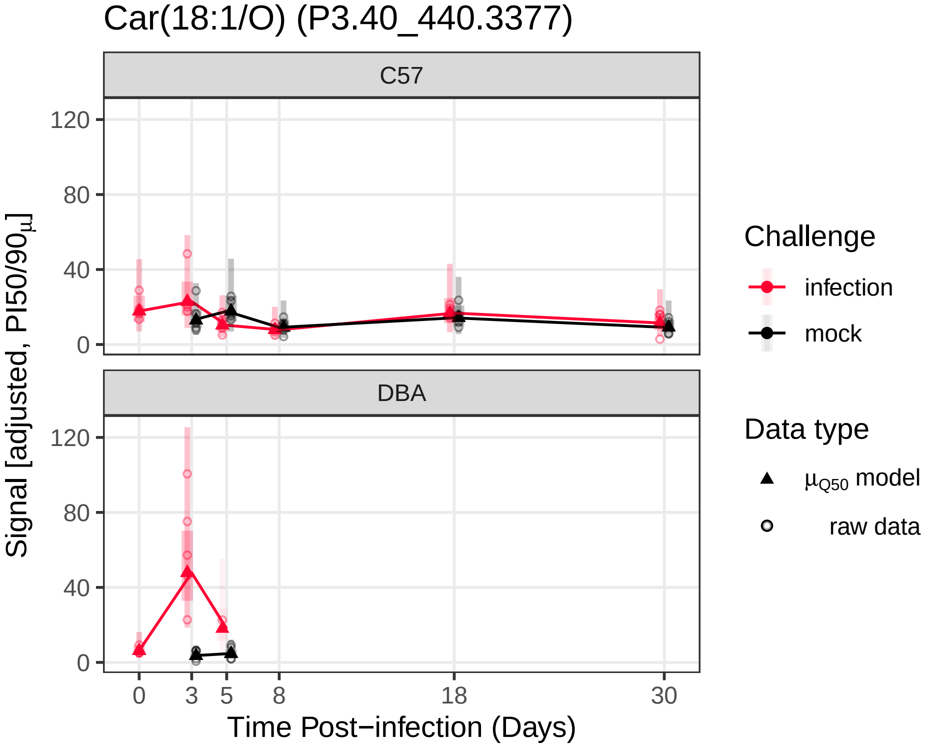
Car(18:1/O) (P3.40_440.3377)
C57
DBA
Signal [adjusted, PI50/90$_{\mu}$]
Time Post−infection (Days)
0
3
5
8
18
30
0
40
80
120
Challenge
infection
mock
Data type
μ_{Q50} model
raw data

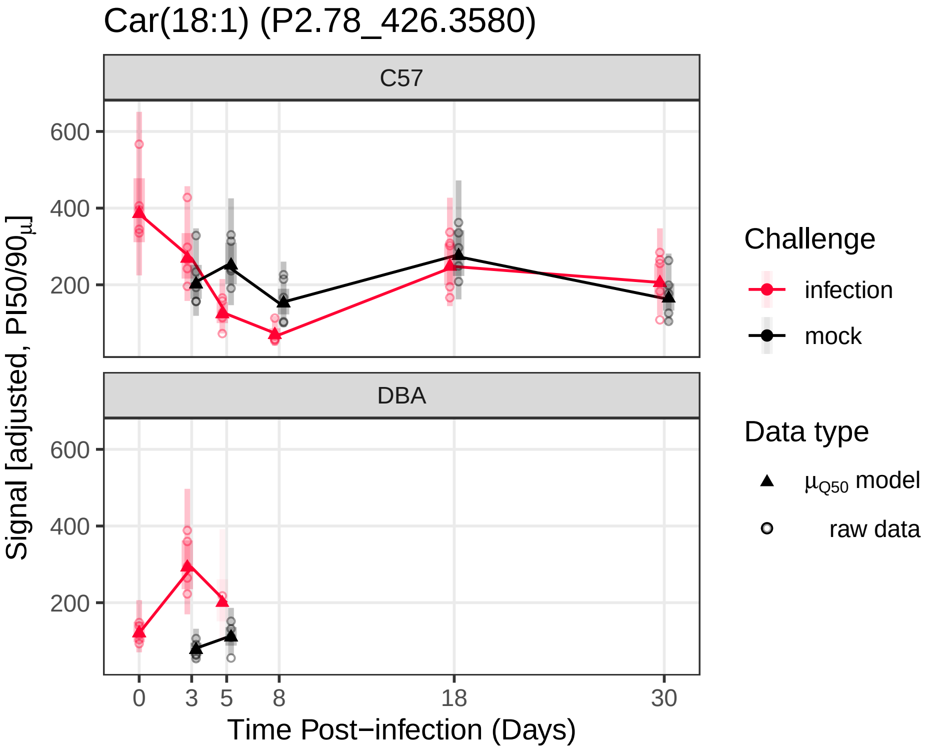

Car(18:1) (P2.78_426.3580)
C57
DBA
600
400
200
0
3
5
8
18
30
Time Post−infection (Days)
Signal [adjusted, PI50/90μ]
Challenge
infection
mock
Data type
μQ50 model
raw data

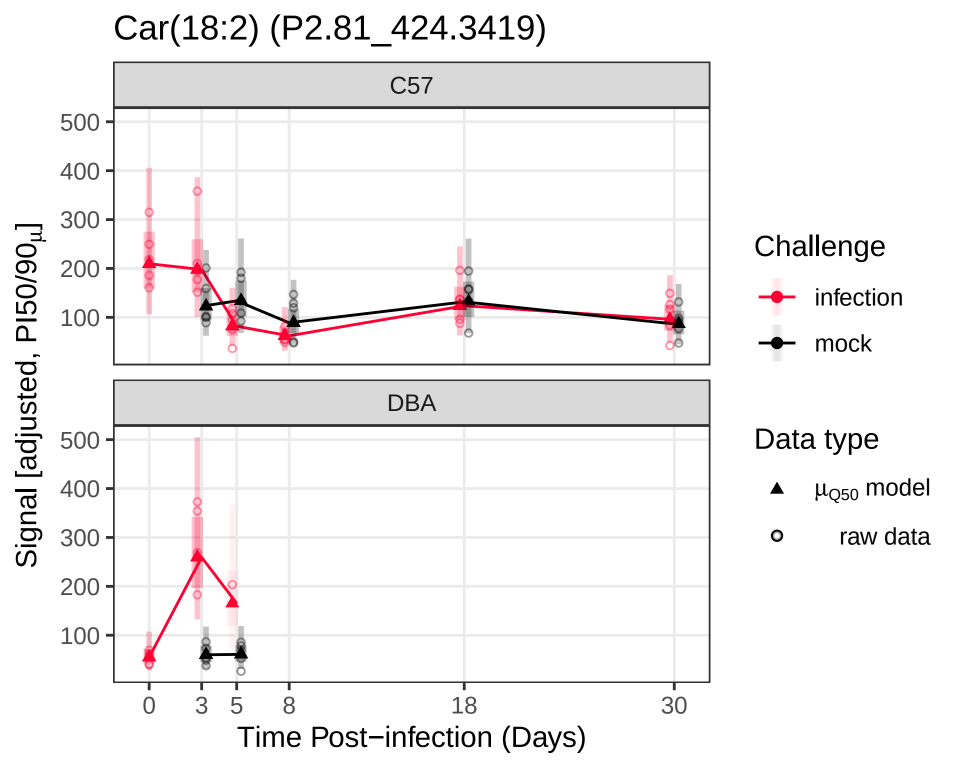

Car(18:2) (P2.81_424.3419)
C57
DBA
Signal [adjusted, PI50/90μ]
500
400
300
200
100
0
3
5
8
18
30
Time Post−infection (Days)
Challenge
infection
mock
Data type
μQ50 model
raw data

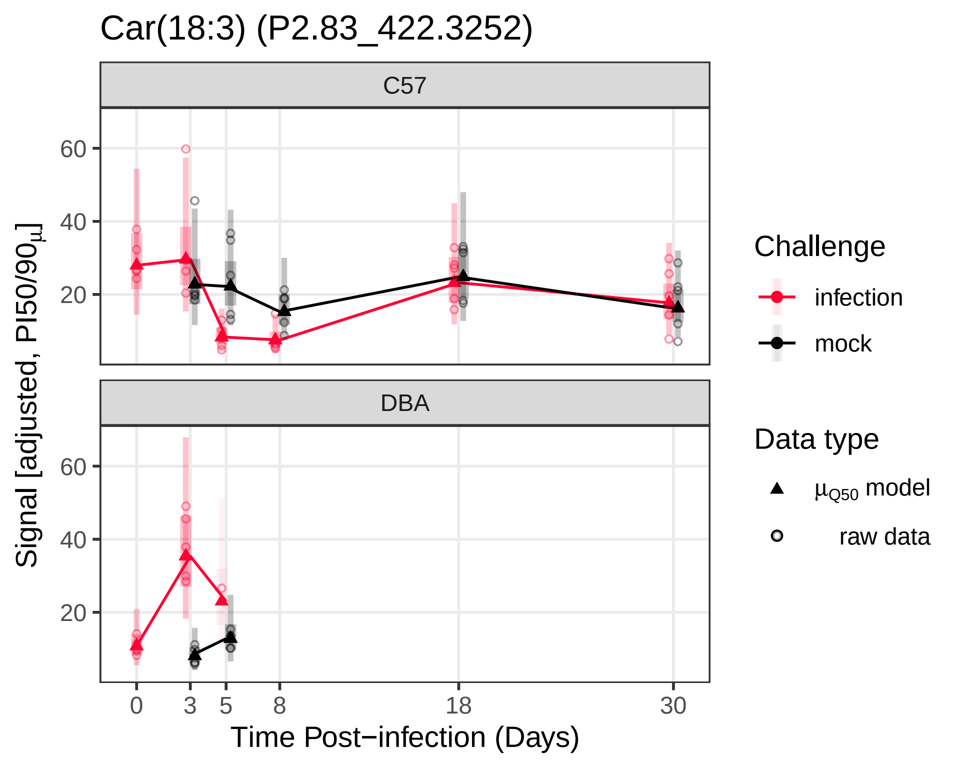

Car(18:3) (P2.83_422.3252)
C57
DBA
Signal [adjusted, PI50/90$_{\mu}$]
60
40
20
0
3
5
8
18
30
Time Post−infection (Days)
Challenge
infection
mock
Data type
μ_{Q50} model
raw data

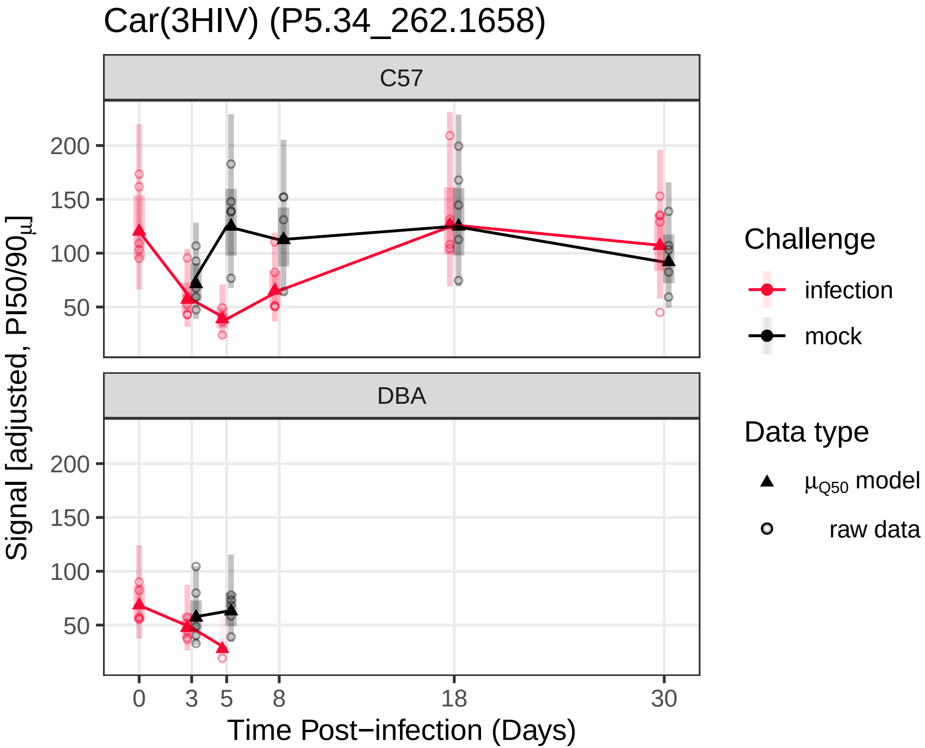

Car(3HIV) (P5.34_262.1658)
C57
DBA
200
150
100
50
Signal [adjusted, PI50/90μ]
0
3
5
8
18
30
Time Post−infection (Days)
Challenge
infection
mock
Data type
μQ50 model
raw data

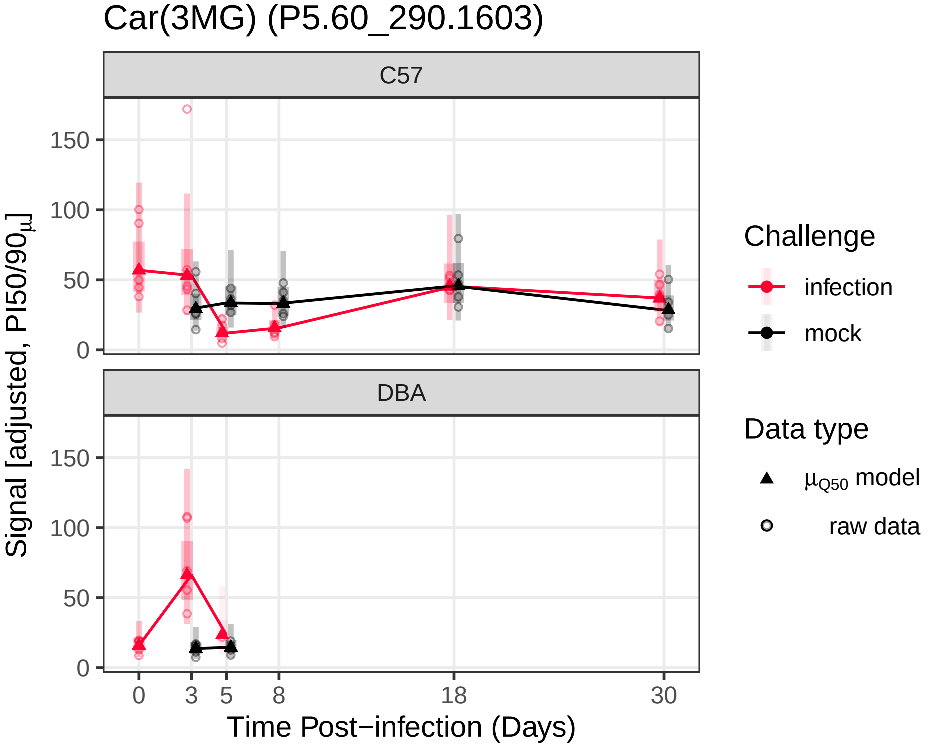
Car(3MG) (P5.60_290.1603)
C57
DBA
150
100
50
0
Signal [adjusted, PI50/90μ]
0
3
5
8
18
30
Time Post−infection (Days)
Challenge
infection
mock
Data type
μQ50 model
raw data

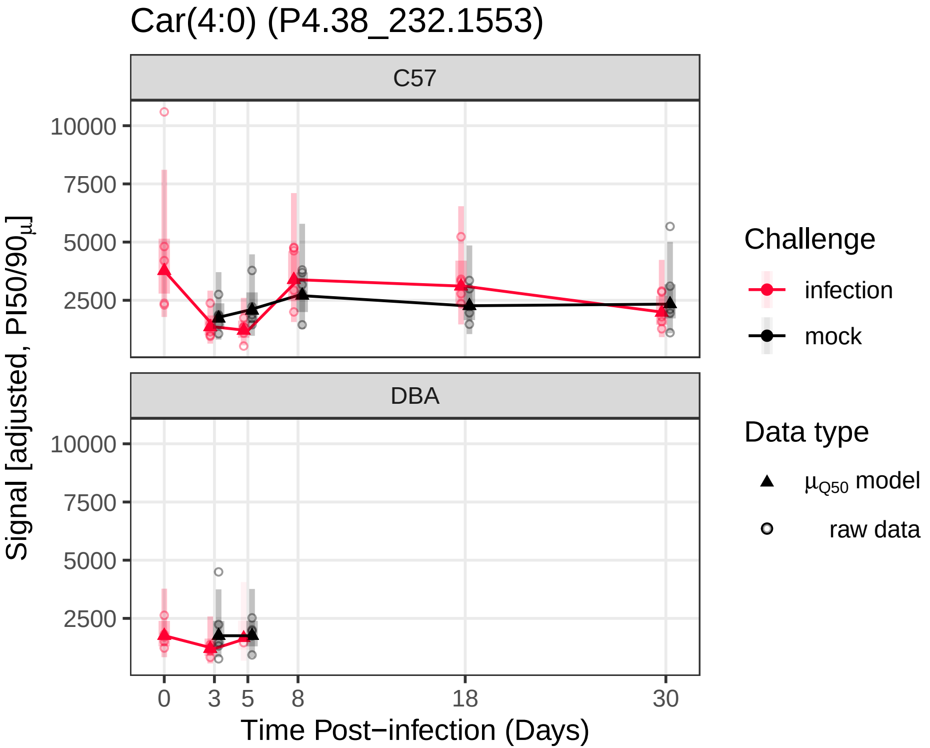

Car(4:0) (P4.38_232.1553)
C57
DBA
10000
7500
5000
2500
0
3
5
8
18
30
Time Post−infection (Days)
Signal [adjusted, PI50/90μ]
Challenge
infection
mock
Data type
μQ50 model
raw data

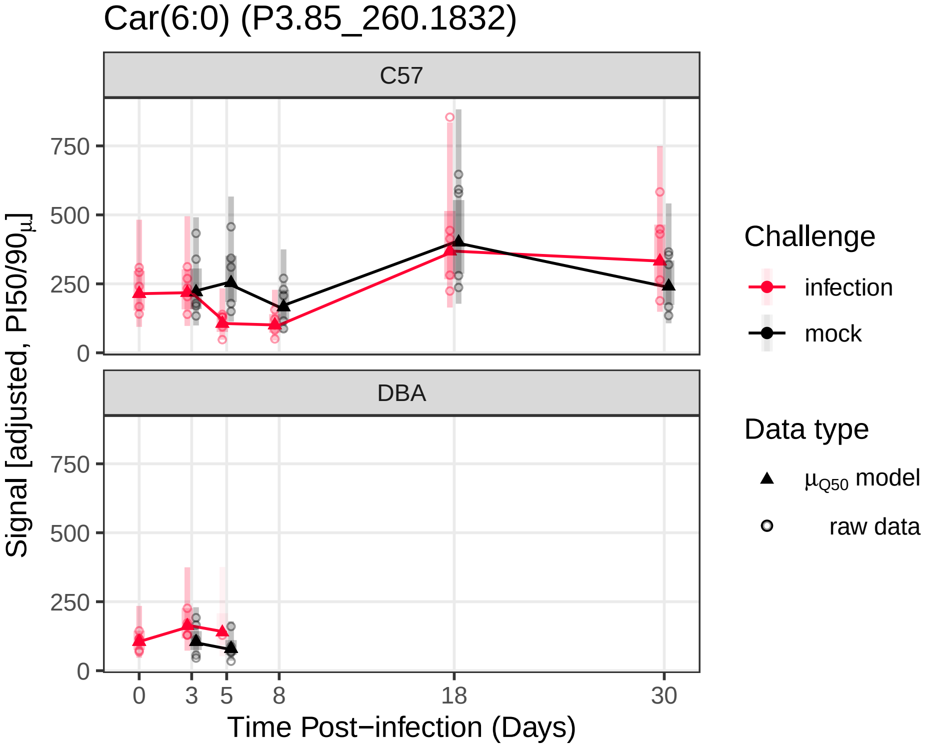

Car(6:0) (P3.85_260.1832)
C57
DBA
750
500
250
0
Signal [adjusted, PI50/90$_\mu$]
0
3
5
8
18
30
Time Post−infection (Days)
Challenge
infection
mock
Data type
μ_{Q50} model
raw data

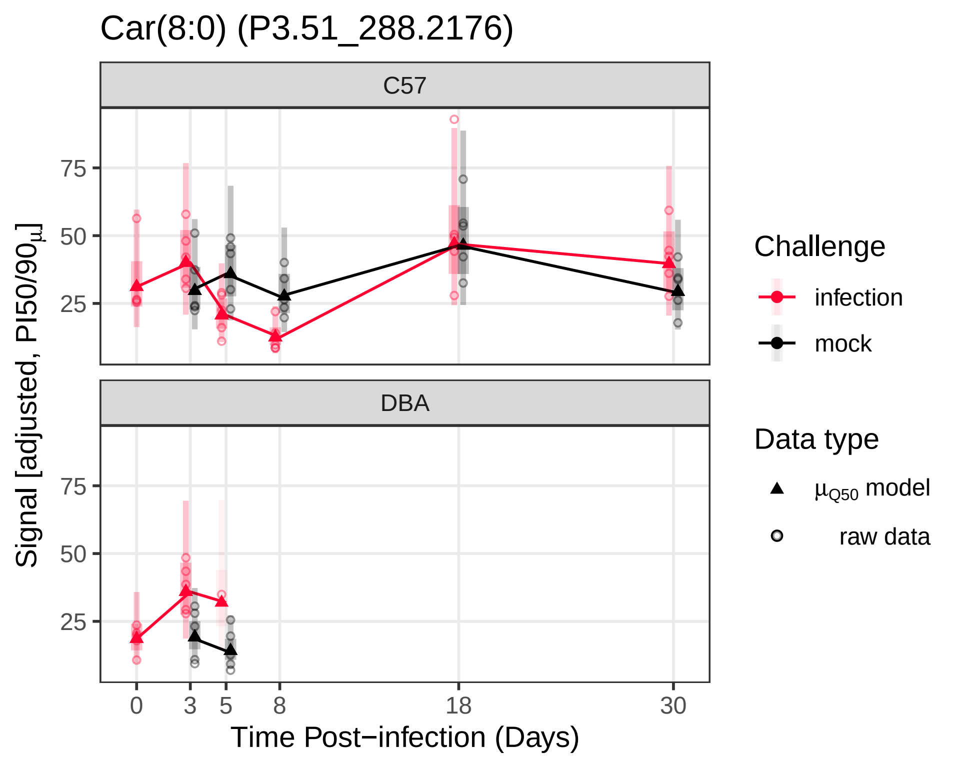
Car(8:0) (P3.51_288.2176)
C57
DBA
75
50
25
Signal [adjusted, PI50/90μ]
0
3
5
8
18
30
Time Post−infection (Days)
Challenge
infection
mock
Data type
μQ50 model
raw data

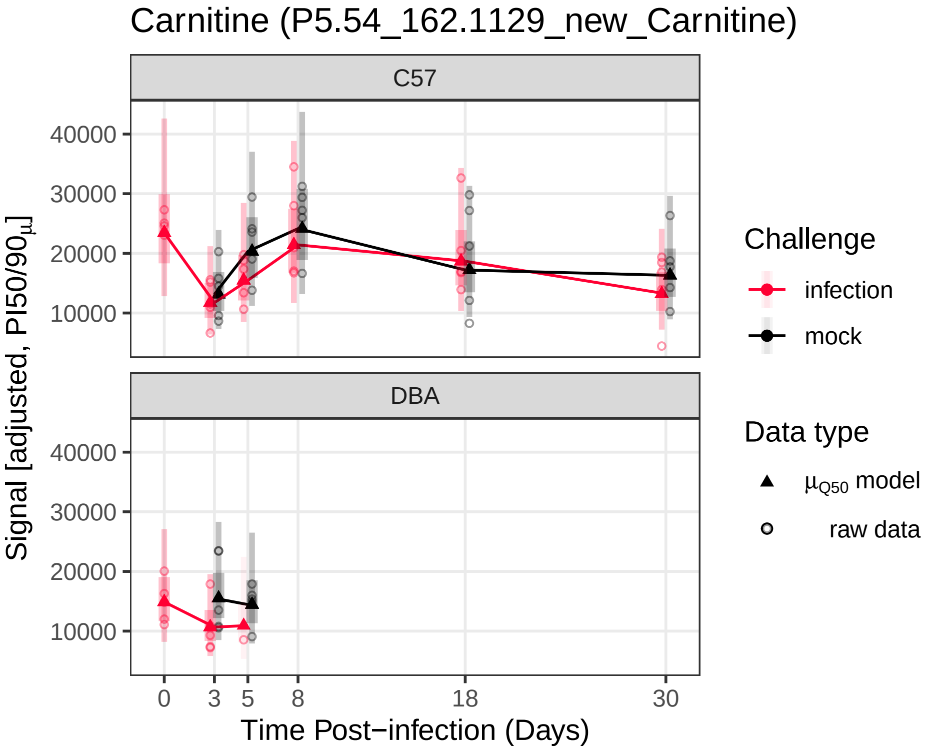

Carnitine (P5.54_162.1129_new_Carnitine)
C57
DBA
Signal [adjusted, PI50/90μ]
40000
30000
20000
10000
0
3
5
8
18
30
Time Post−infection (Days)
Challenge
infection
mock
Data type
μQ50 model
raw data

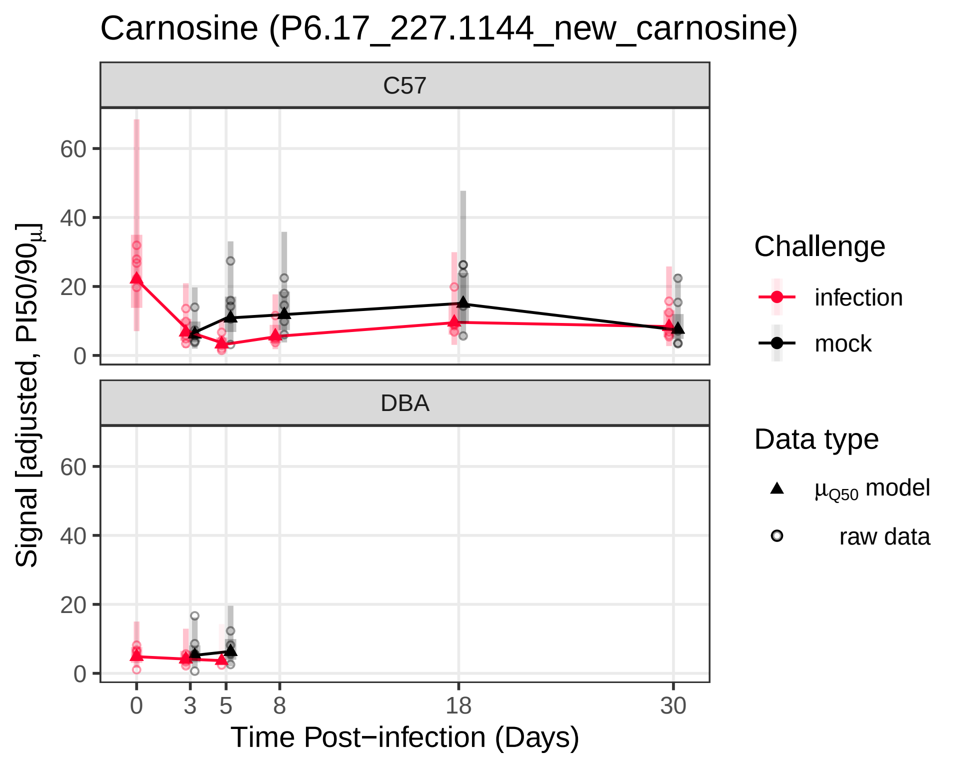
Carnosine (P6.17_227.1144_new_carnosine)
C57
DBA
Signal [adjusted, PI50/90μ]
Time Post−infection (Days)
0
3
5
8
18
30
60
40
20
0
Challenge
infection
mock
Data type
μQ50 model
raw data

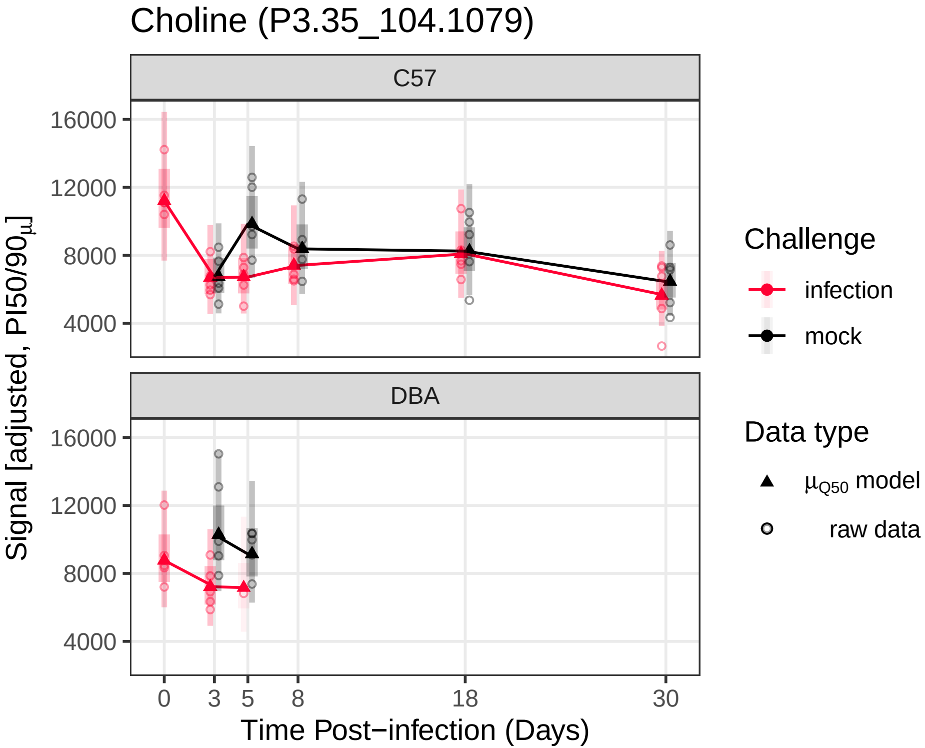

Choline (P3.35_104.1079)
C57
DBA
16000
12000
8000
4000
Signal [adjusted, PI50/90μ]
0
3
5
8
18
30
Time Post−infection (Days)
Challenge
infection
mock
Data type
μQ50 model
raw data

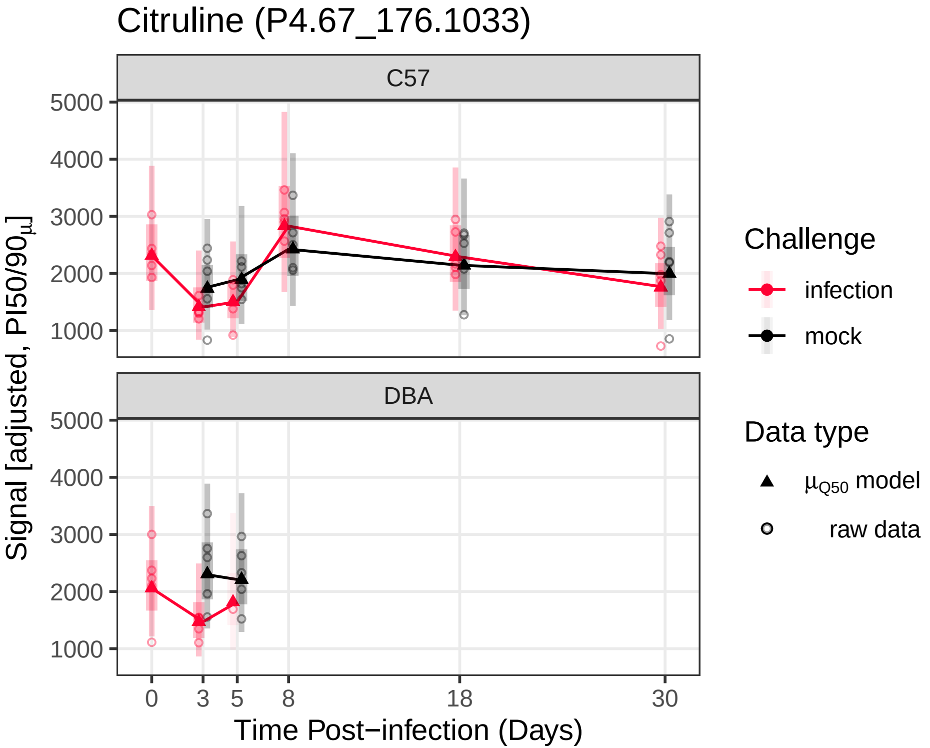

Citruline (P4.67_176.1033)
C57
DBA
5000
4000
3000
2000
1000
Signal [adjusted, PI50/90μ]
0
3
5
8
18
30
Time Post−infection (Days)
Challenge
infection
mock
Data type
μQ50 model
raw data

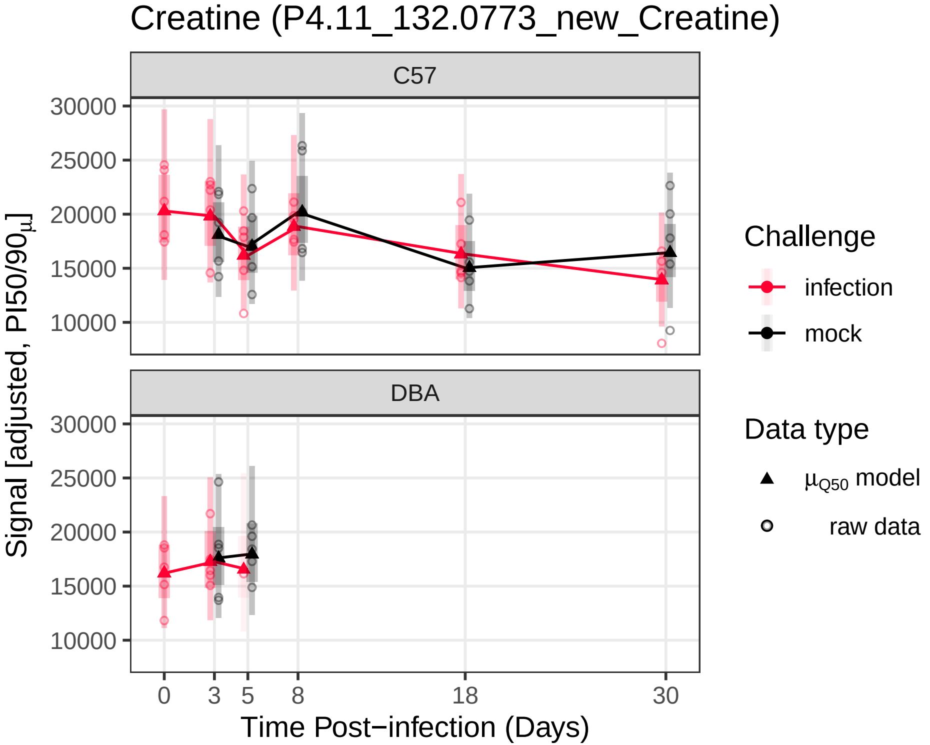

Creatine (P4.11_132.0773_new_Creatine)
C57
DBA
Signal [adjusted, PI50/90μ]
30000
25000
20000
15000
10000
0
3
5
8
18
30
Time Post−infection (Days)
Challenge
infection
mock
Data type
μQ50 model
raw data

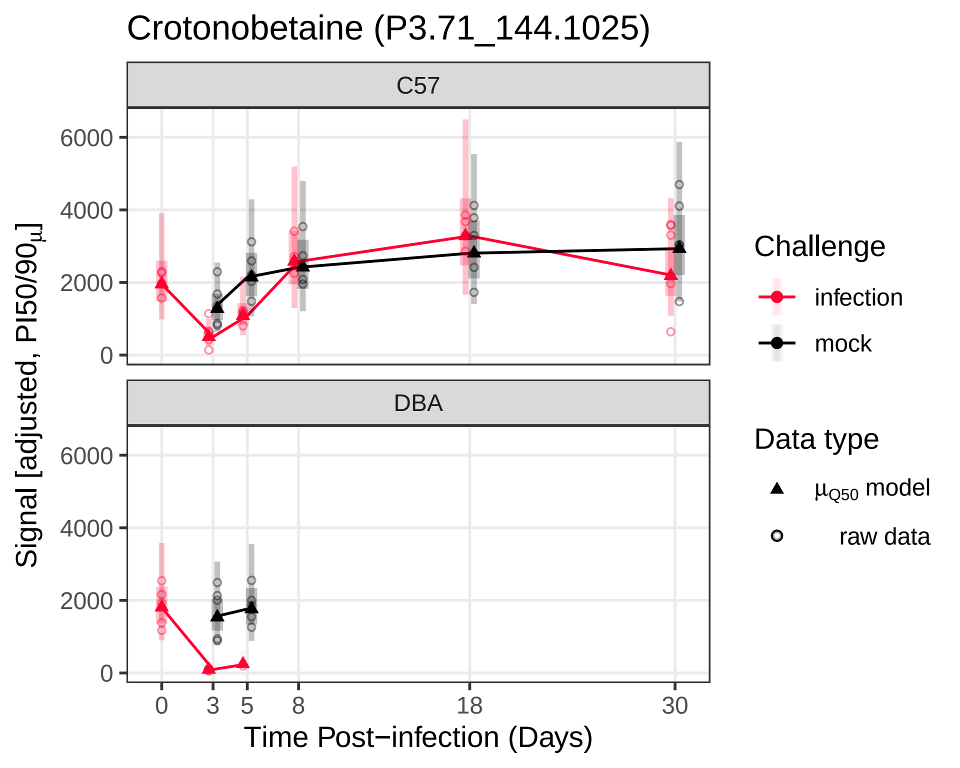
Crotonobetaine (P3.71_144.1025)
C57
DBA
6000
4000
2000
0
Signal [adjusted, PI50/90μ]
0
3
5
8
18
30
Time Post−infection (Days)
Challenge
infection
mock
Data type
μQ50 model
raw data

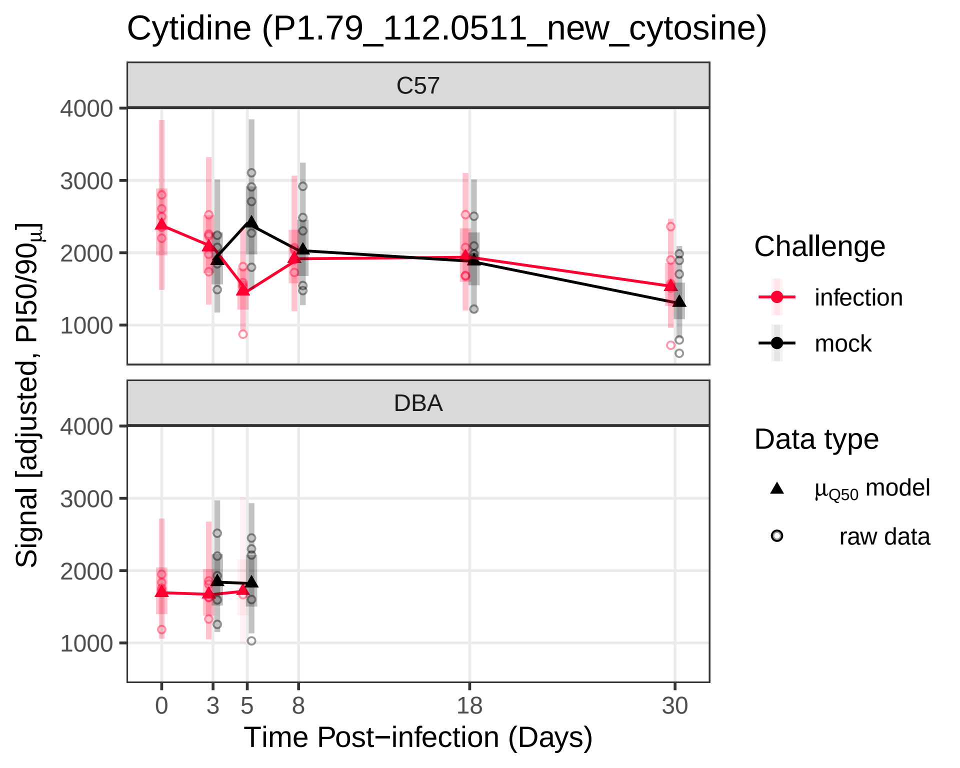

Cytidine (P1.79_112.0511_new_cytosine)
C57
DBA
4000
3000
2000
1000
Signal [adjusted, PI50/90μ]
0
3
5
8
18
30
Time Post−infection (Days)
Challenge
infection
mock
Data type
μQ50 model
raw data

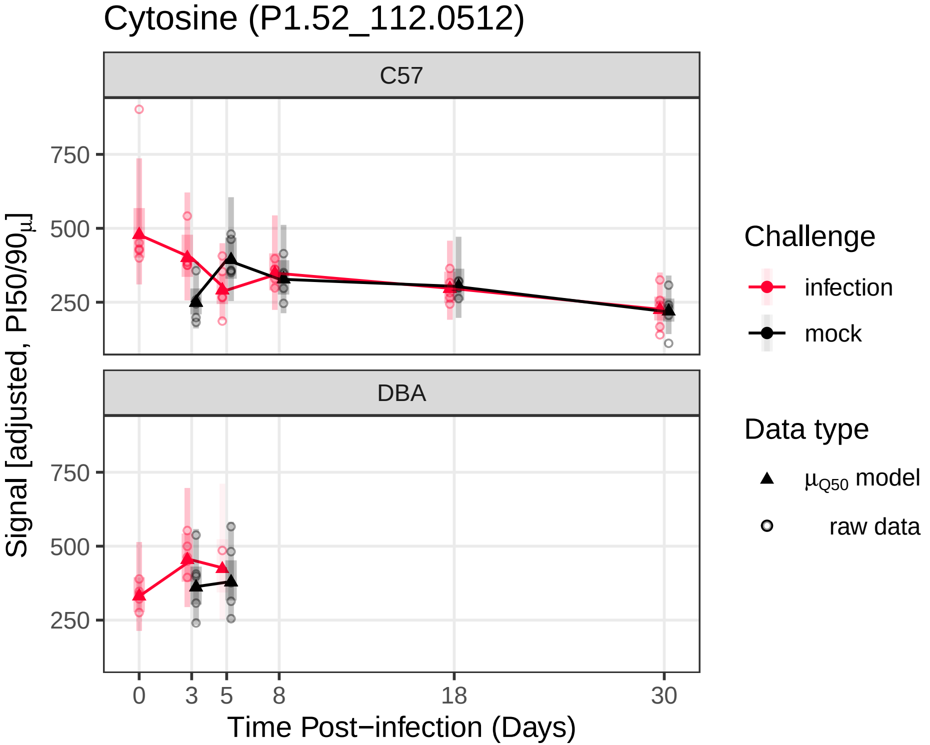

Cytosine (P1.52_112.0512)
C57
DBA
Signal [adjusted, PI50/90μ]
750
500
250
0
3
5
8
18
30
Time Post−infection (Days)
Challenge
infection
mock
Data type
μQ50 model
raw data

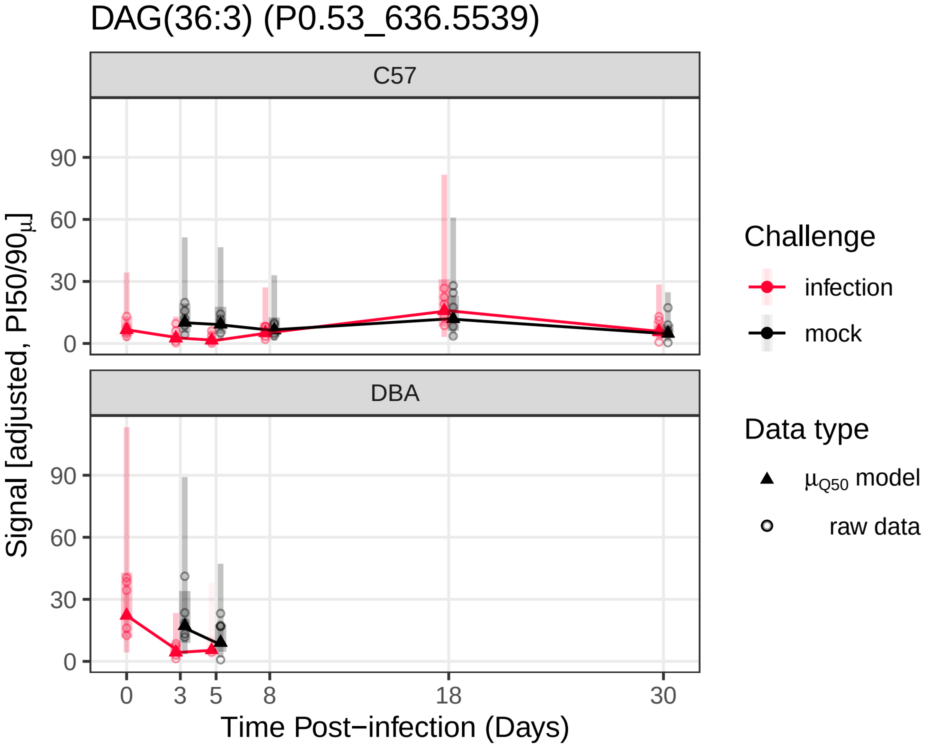
DAG(36:3) (P0.53_636.5539)
C57
DBA
Signal [adjusted, PI50/90$_{\mu}$]
90
60
30
0
0
3
5
8
18
30
Time Post−infection (Days)
Challenge
infection
mock
Data type
μ_{Q50} model
raw data

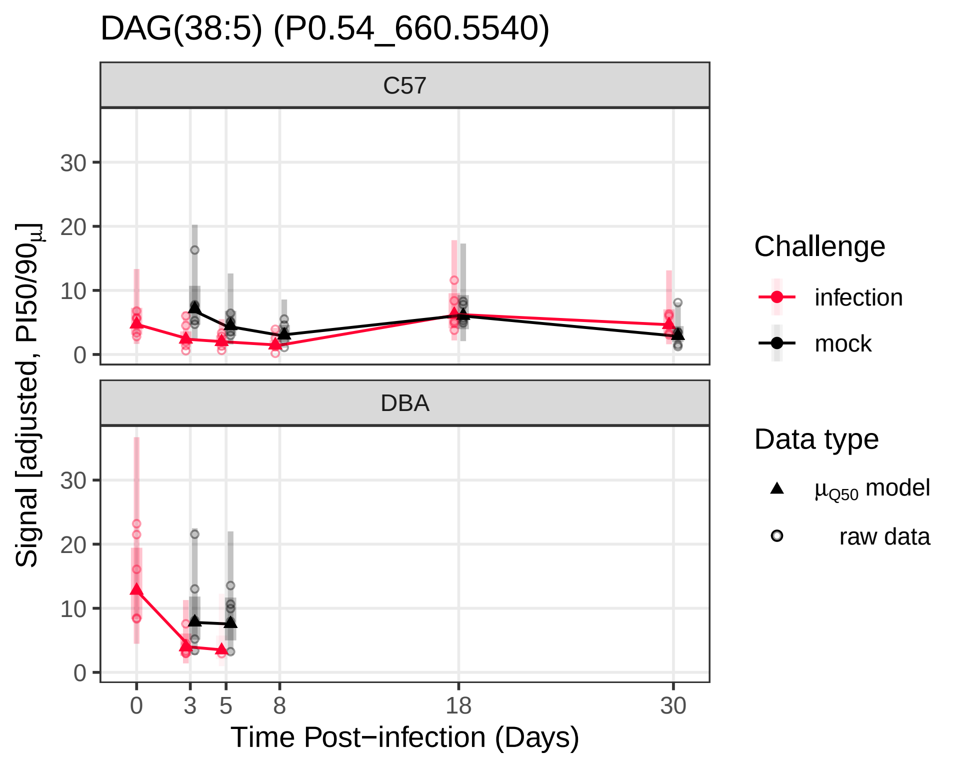

DAG(38:5) (P0.54_660.5540)
C57
DBA
Signal [adjusted, PI50/90μ]
Time Post−infection (Days)
0
3
5
8
18
30
0
10
20
30
Challenge
infection
mock
Data type
μQ50 model
raw data

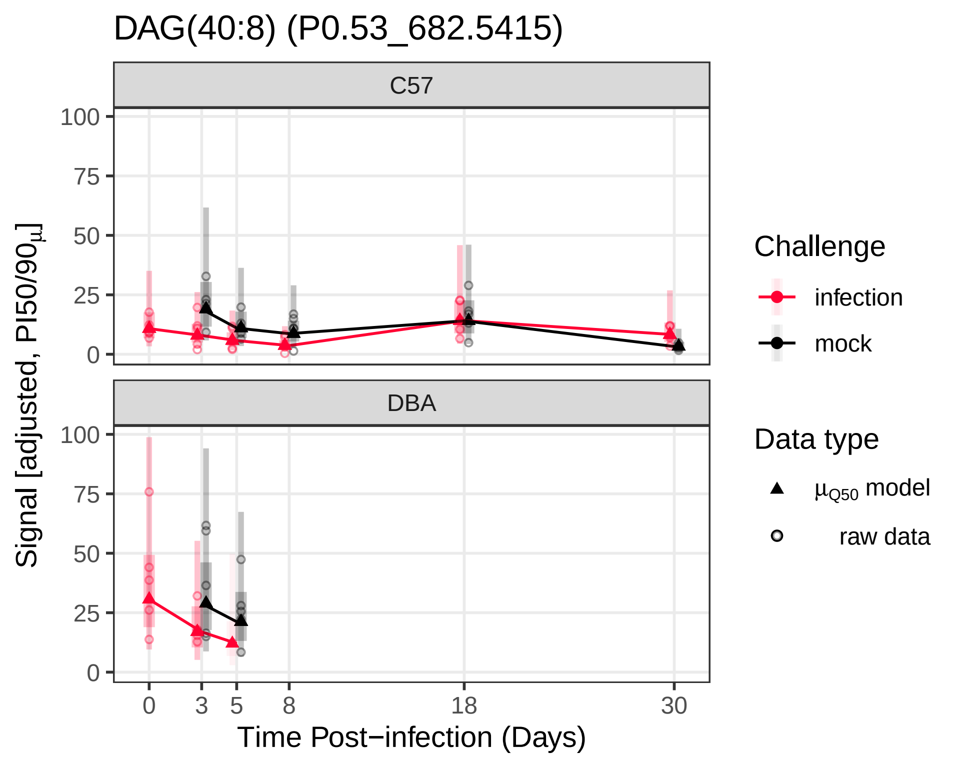
DAG(40:8) (P0.53_682.5415)
C57
DBA
100
75
50
25
0
0
3
5
8
18
30
Time Post−infection (Days)
Signal [adjusted, PI50/90μ]
Challenge
infection
mock
Data type
μQ50 model
raw data

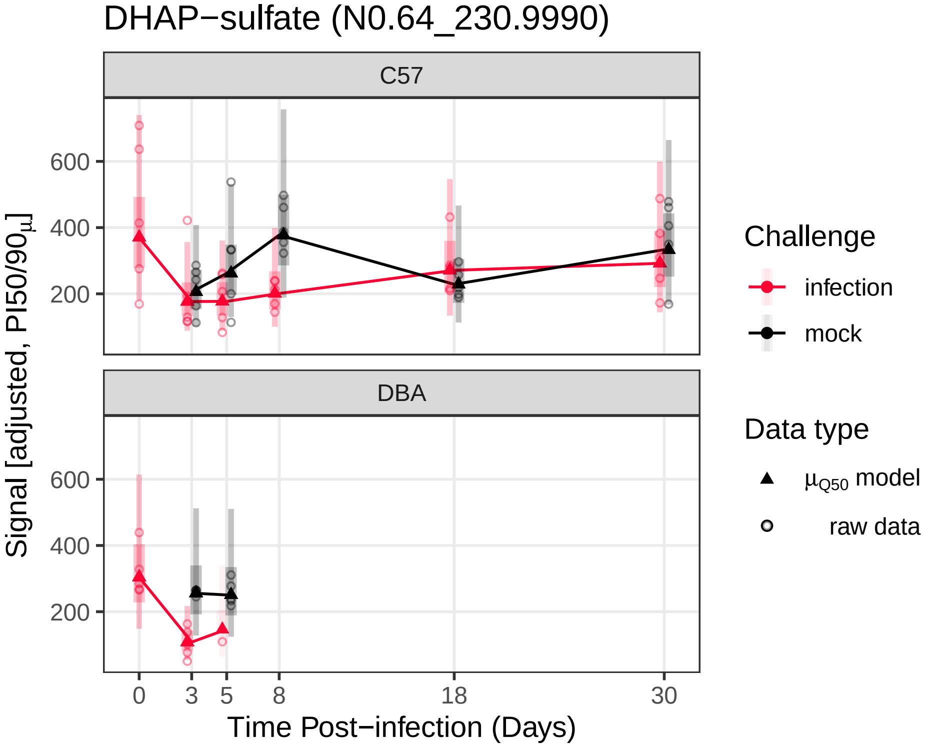
DHAP−sulfate (N0.64_230.9990)
C57
DBA
600
400
200
0
3
5
8
18
30
Time Post−infection (Days)
Signal [adjusted, PI50/90μ]
Challenge
infection
mock
Data type
μQ50 model
raw data

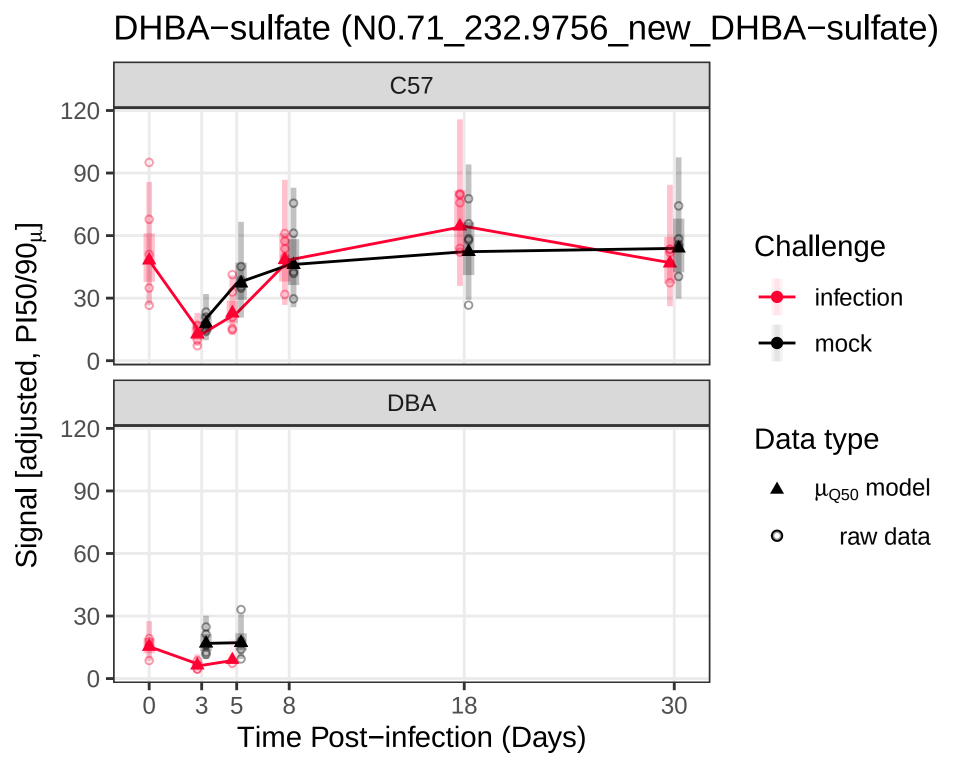

DHBA−sulfate (N0.71_232.9756_new_DHBA−sulfate)
C57
DBA
Signal [adjusted, PI50/90μ]
120
90
60
30
0
0
3
5
8
18
30
Time Post−infection (Days)
Challenge
infection
mock
Data type
μQ50 model
raw data

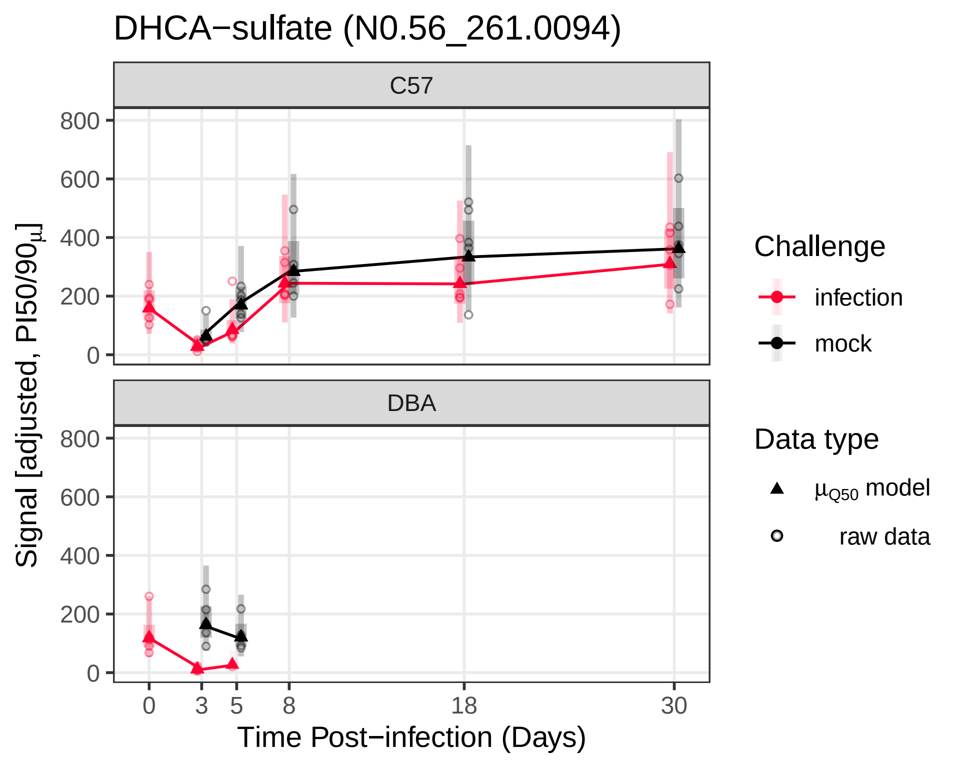
DHCA−sulfate (N0.56_261.0094)
C57
DBA
Signal [adjusted, PI50/90μ]
Time Post−infection (Days)
0
200
400
600
800
0
3
5
8
18
30
Challenge
infection
mock
Data type
μQ50 model
raw data

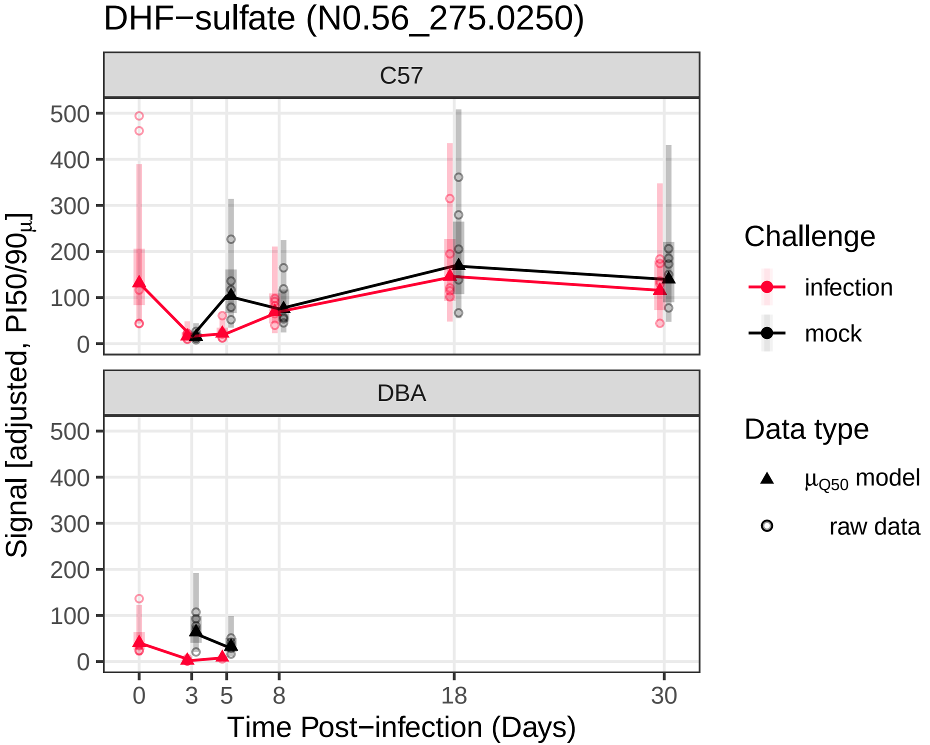

DHF−sulfate (N0.56_275.0250)
C57
DBA
500
400
300
200
100
0
Signal [adjusted, PI50/90μ]
0
3
5
8
18
30
Time Post−infection (Days)
Challenge
infection
mock
Data type
μQ50 model
raw data

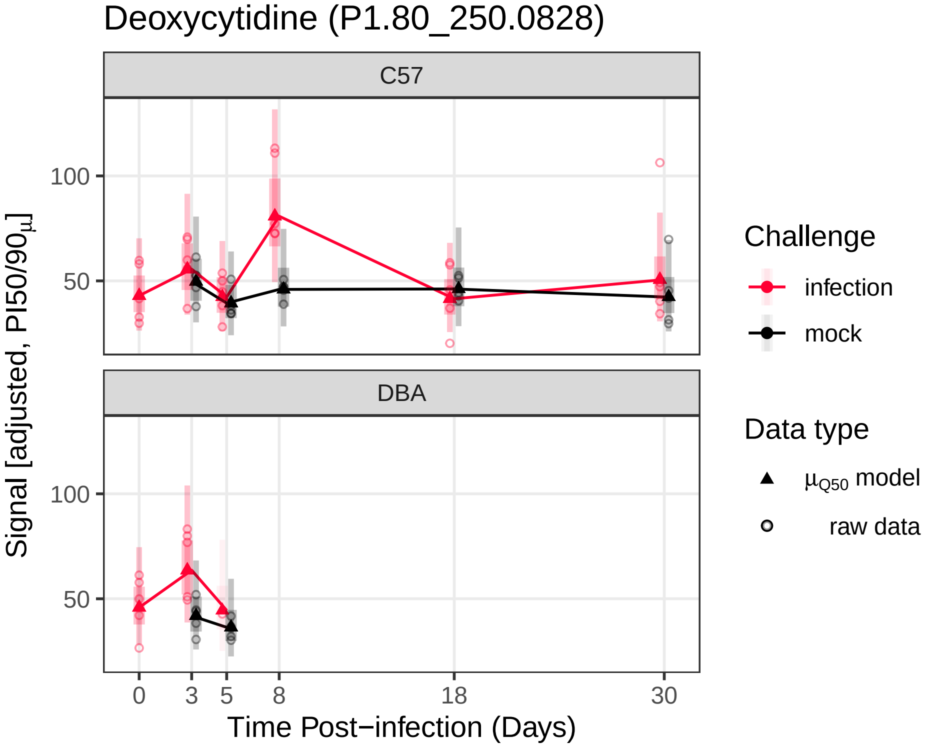
Deoxycytidine (P1.80_250.0828)
C57
DBA
100
50
Signal [adjusted, PI50/90μ]
0
3
5
8
18
30
Time Post−infection (Days)
Challenge
infection
mock
Data type
μQ50 model
raw data

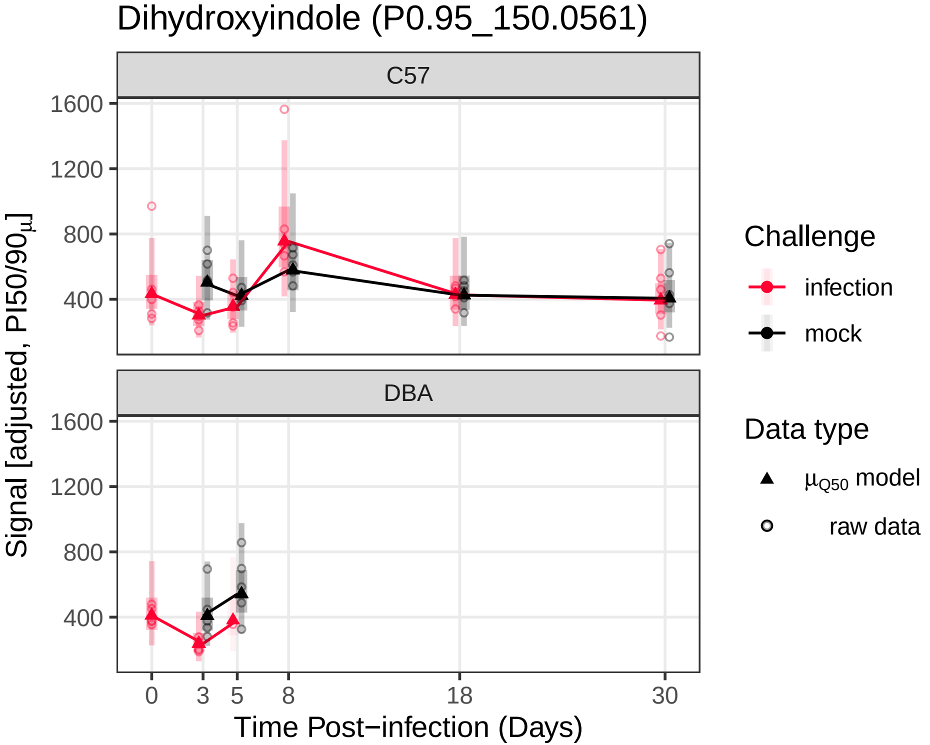

Dihydroxyindole (P0.95_150.0561)
C57
DBA
Signal [adjusted, PI50/90μ]
1600
1200
800
400
0
3
5
8
18
30
Time Post−infection (Days)
Challenge
infection
mock
Data type
μQ50 model
raw data

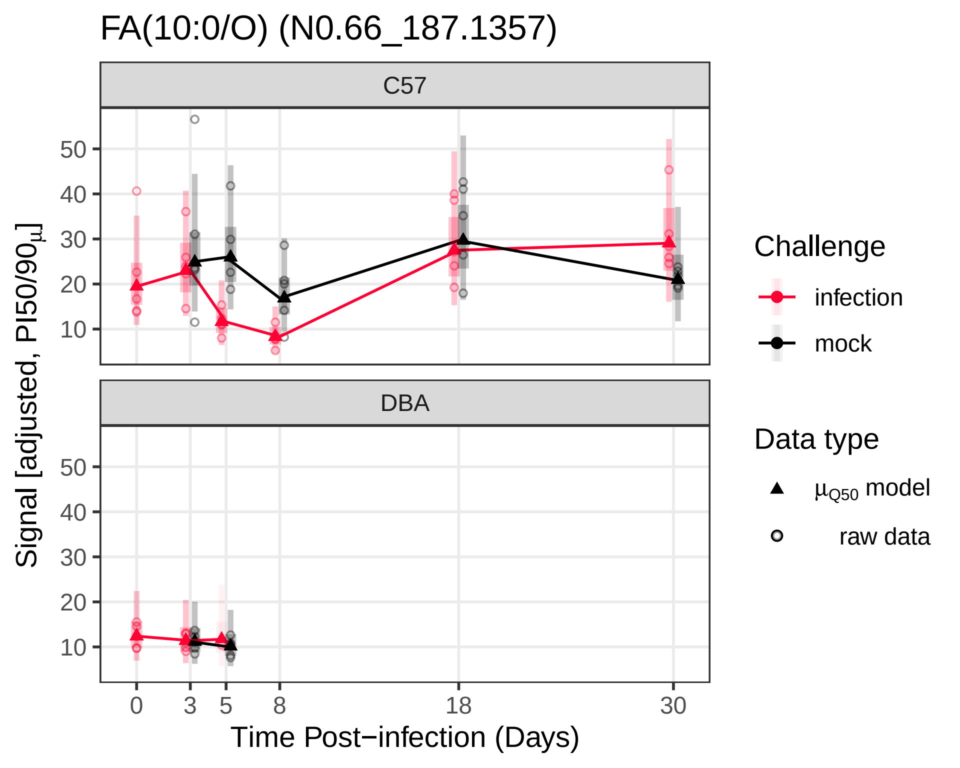
FA(10:0/O) (N0.66_187.1357)
C57
DBA
Signal [adjusted, PI50/90μ]
Time Post−infection (Days)
0
3
5
8
18
30
10
20
30
40
50
Challenge
infection
mock
Data type
μQ50 model
raw data

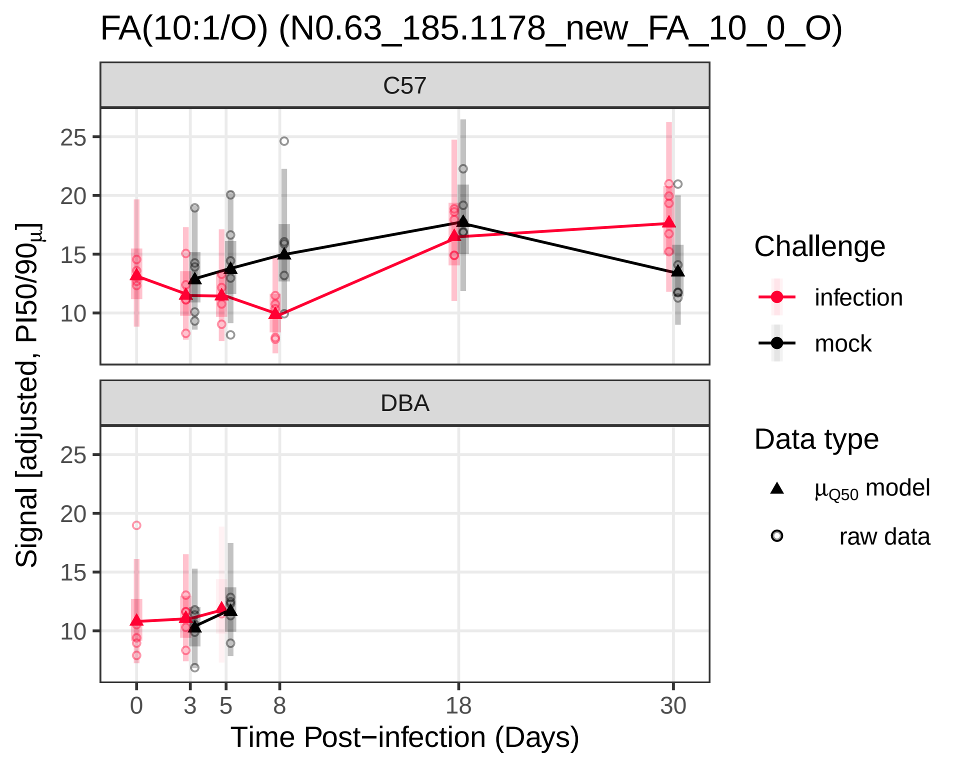
FA(10:1/O) (N0.63_185.1178_new_FA_10_0_O)
C57
DBA
Signal [adjusted, PI50/90μ]
Time Post−infection (Days)
0
3
5
8
18
30
10
15
20
25
Challenge
infection
mock
Data type
μQ50 model
raw data

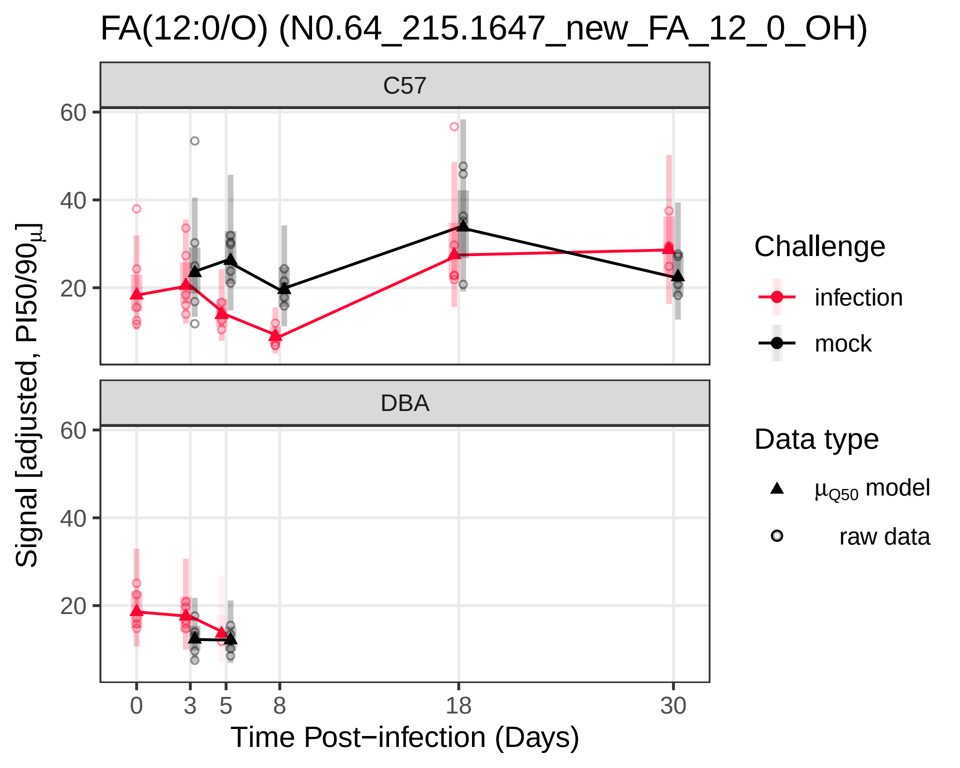

FA(12:0/O) (N0.64_215.1647_new_FA_12_0_OH)
C57
DBA
60
40
20
Signal [adjusted, PI50/90μ]
0
3
5
8
18
30
Time Post−infection (Days)
Challenge
infection
mock
Data type
μQ50 model
raw data

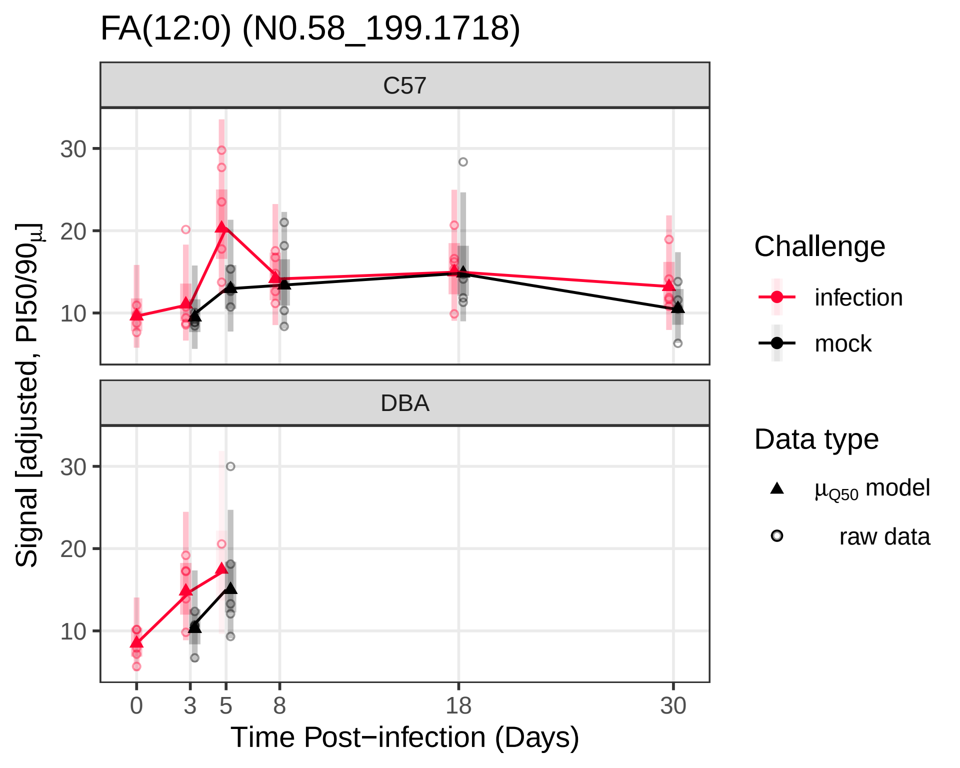
FA(12:0) (N0.58_199.1718)
C57
DBA
30
20
10
0
3
5
8
18
30
Signal [adjusted, PI50/90μ]
Time Post−infection (Days)
Challenge
infection
mock
Data type
μQ50 model
raw data

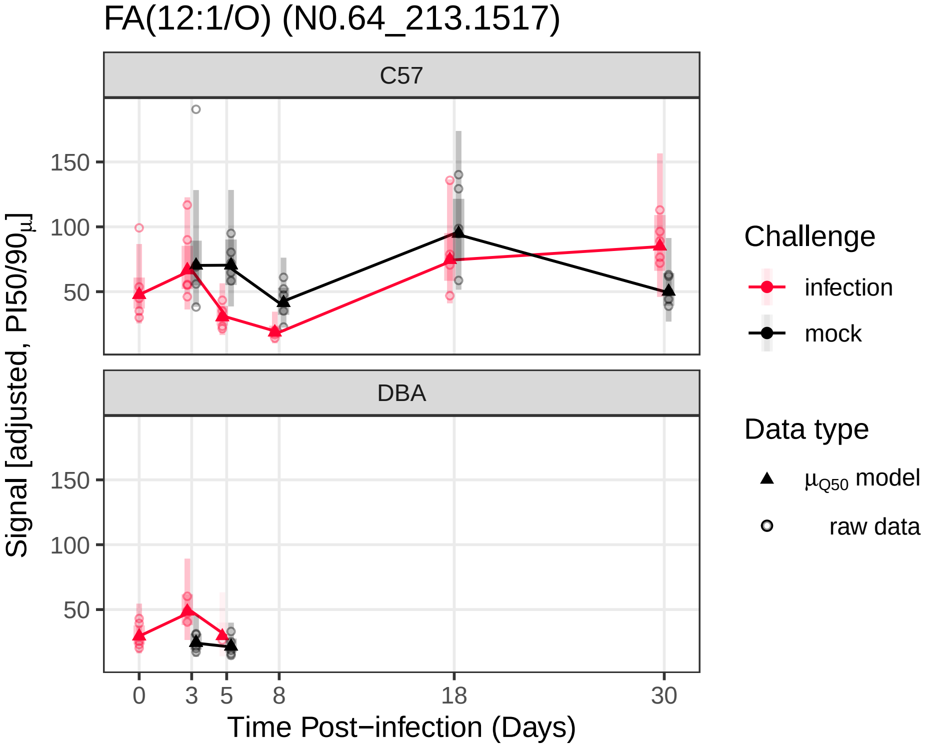
FA(12:1/O) (N0.64_213.1517)
C57
DBA
Signal [adjusted, PI50/90$_{\mu}$]
150
100
50
0
3
5
8
18
30
Time Post−infection (Days)
Challenge
infection
mock
Data type
μ_{Q50} model
raw data

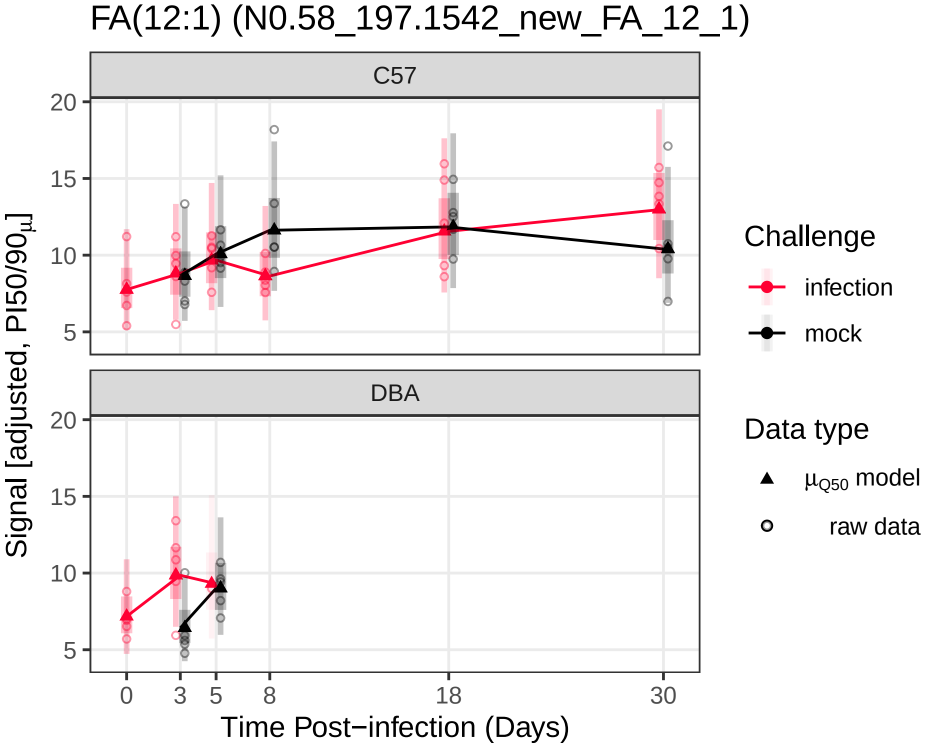
FA(12:1) (N0.58_197.1542_new_FA_12_1)
C57
DBA
Signal [adjusted, PI50/90μ]
20
15
10
5
0
3
5
8
18
30
Time Post−infection (Days)
Challenge
infection
mock
Data type
μQ50 model
raw data

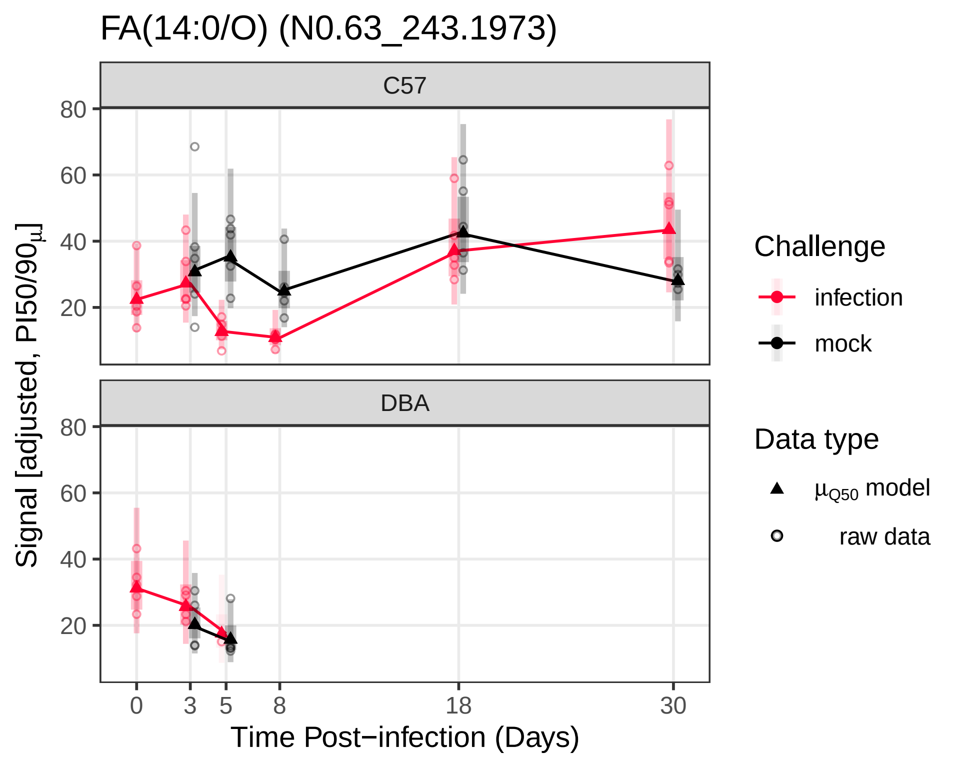
FA(14:0/O) (N0.63_243.1973)
C57
DBA
Signal [adjusted, PI50/90μ]
80
60
40
20
0
3
5
8
18
30
Time Post−infection (Days)
Challenge
infection
mock
Data type
μQ50 model
raw data

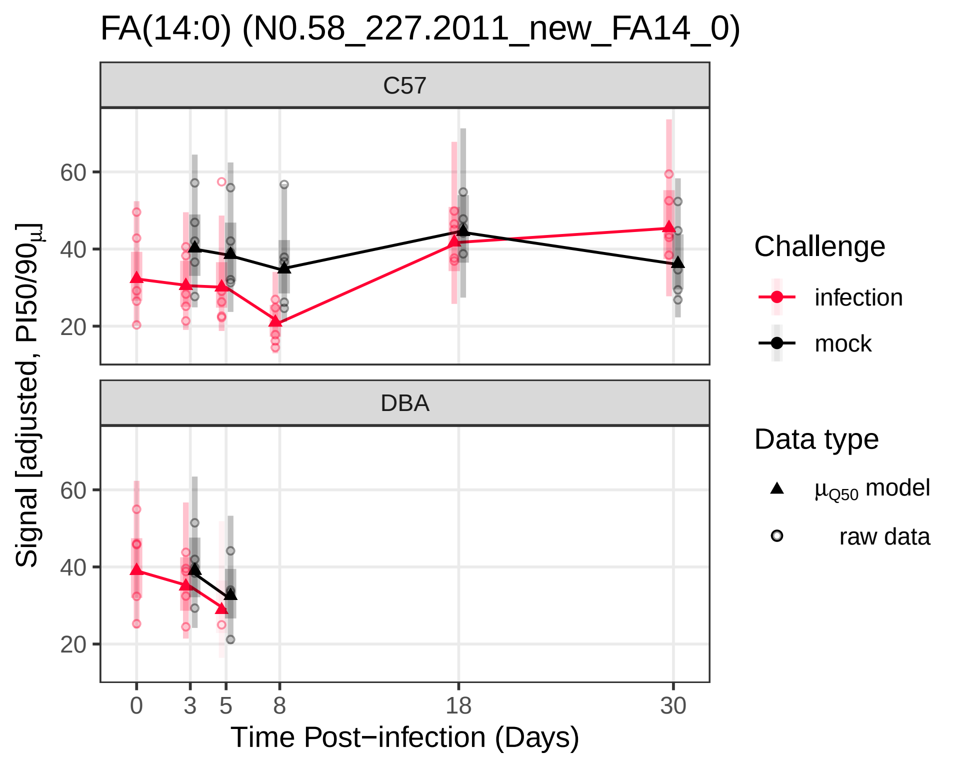
FA(14:0) (N0.58_227.2011_new_FA14_0)
C57
DBA
Signal [adjusted, PI50/90$_{\mu}$]
60
40
20
0
3
5
8
18
30
Time Post−infection (Days)
Challenge
infection
mock
Data type
μ_{Q50} model
raw data

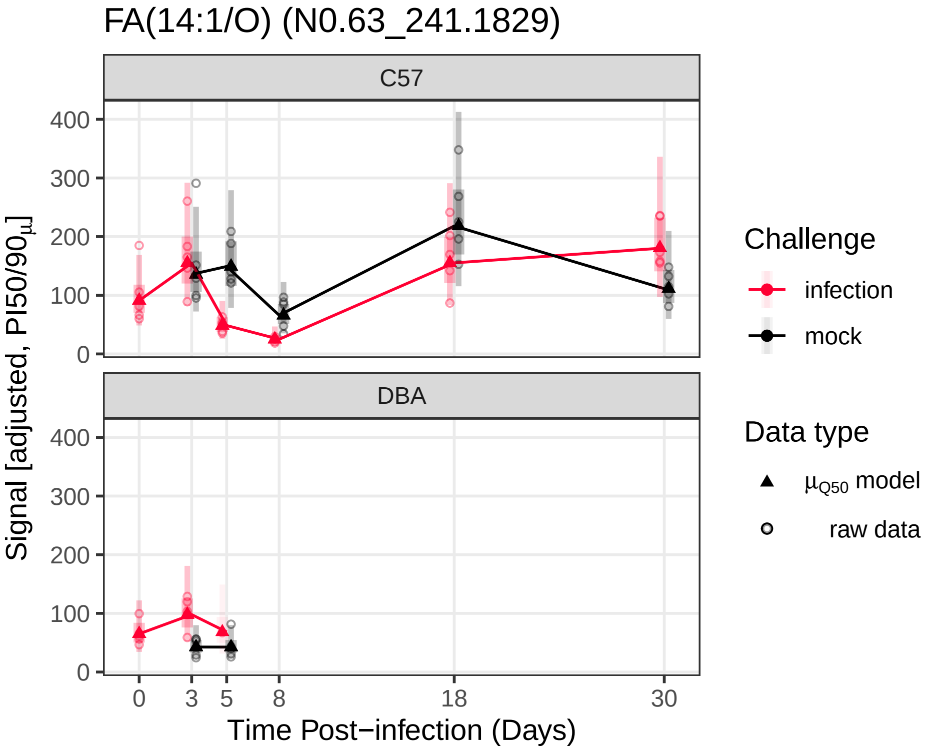
FA(14:1/O) (N0.63_241.1829)
C57
DBA
400
300
200
100
0
Signal [adjusted, PI50/90μ]
0
3
5
8
18
30
Time Post−infection (Days)
Challenge
infection
mock
Data type
μQ50 model
raw data

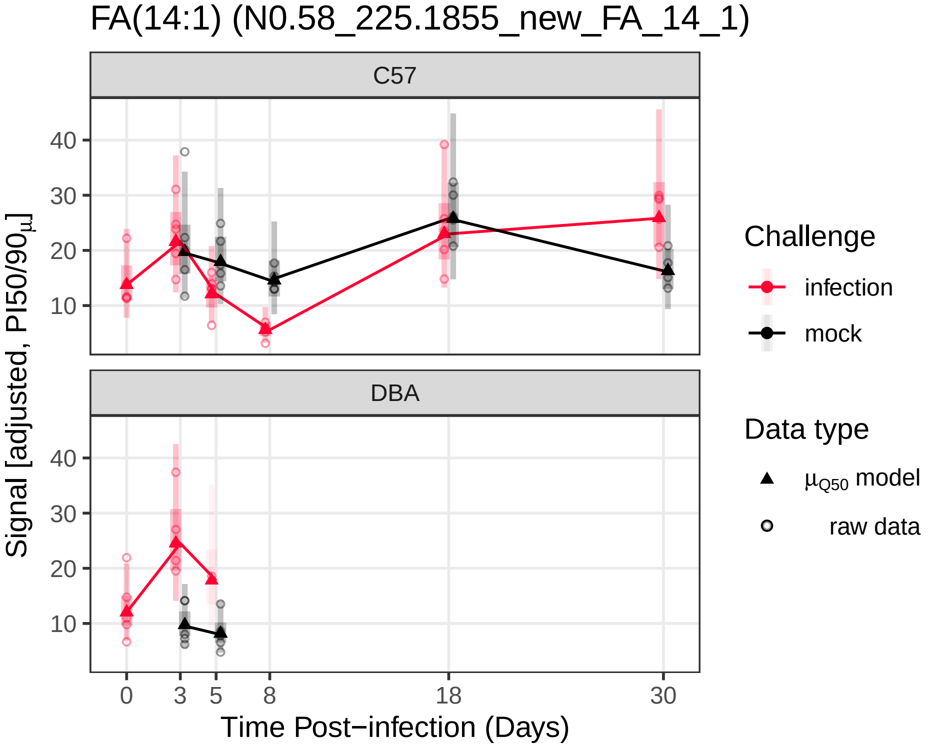
FA(14:1) (N0.58_225.1855_new_FA_14_1)
C57
DBA
Signal [adjusted, PI50/90μ]
40
30
20
10
0
3
5
8
18
30
Time Post−infection (Days)
Challenge
infection
mock
Data type
μQ50 model
raw data

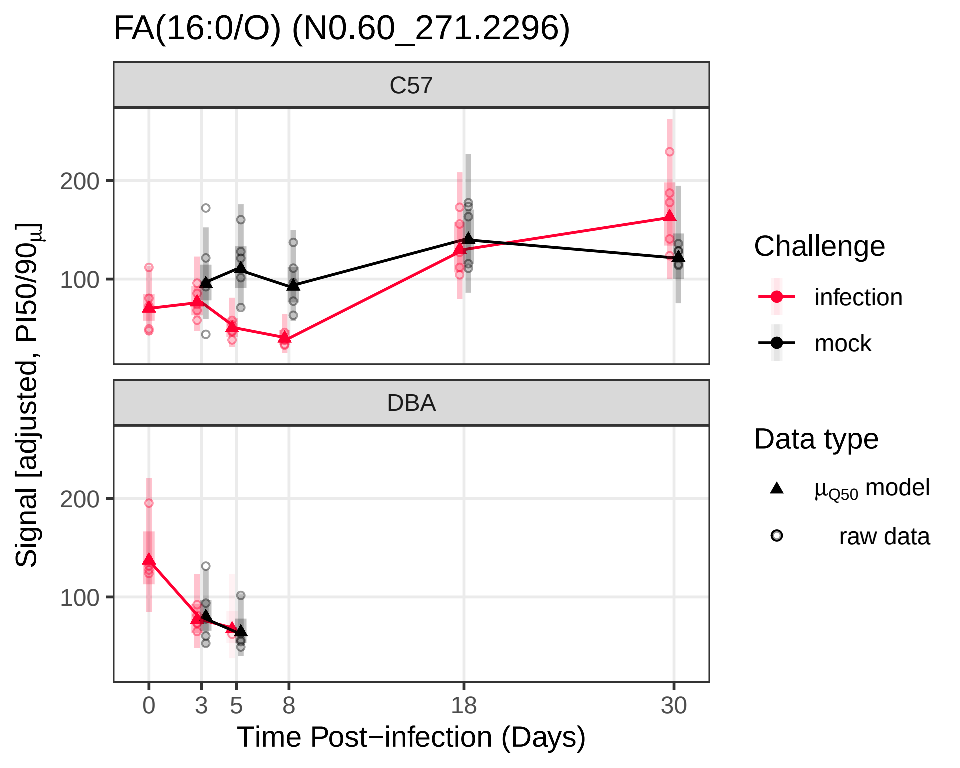
FA(16:0/O) (N0.60_271.2296)
C57
DBA
200
100
200
100
0
3
5
8
18
30
Time Post−infection (Days)
Signal [adjusted, PI50/90μ]
Challenge
infection
mock
Data type
μQ50 model
raw data

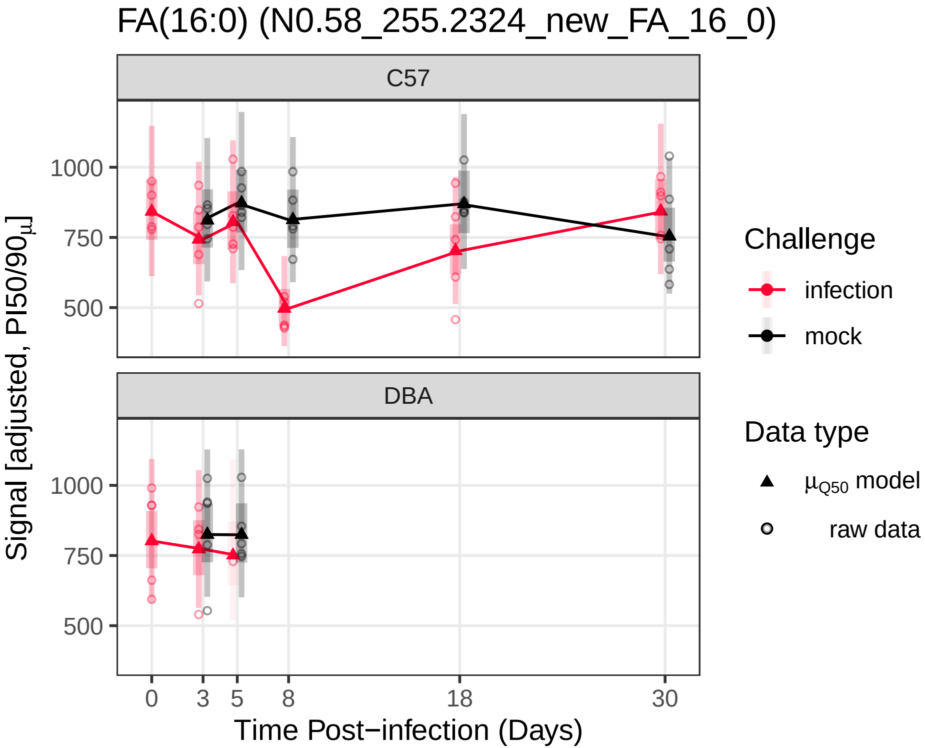

FA(16:0) (N0.58_255.2324_new_FA_16_0)
C57
DBA
Signal [adjusted, PI50/90μ]
1000
750
500
0
3
5
8
18
30
Time Post−infection (Days)
Challenge
infection
mock
Data type
μQ50 model
raw data

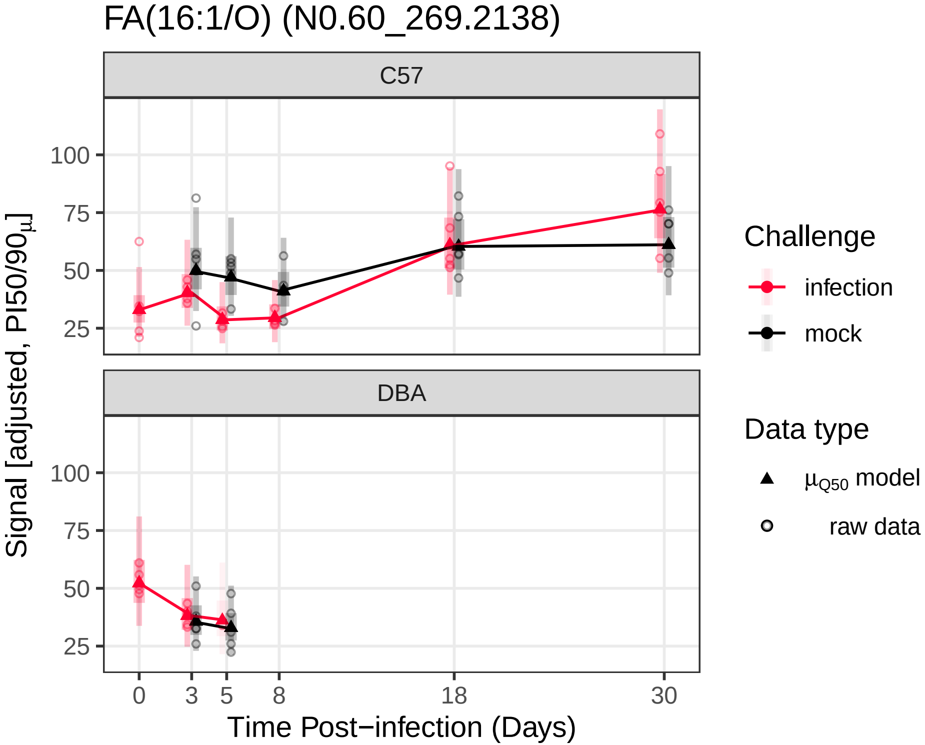
FA(16:1/O) (N0.60_269.2138)
C57
DBA
Signal [adjusted, PI50/90μ]
100
75
50
25
0
3
5
8
18
30
Time Post−infection (Days)
Challenge
infection
mock
Data type
μQ50 model
raw data

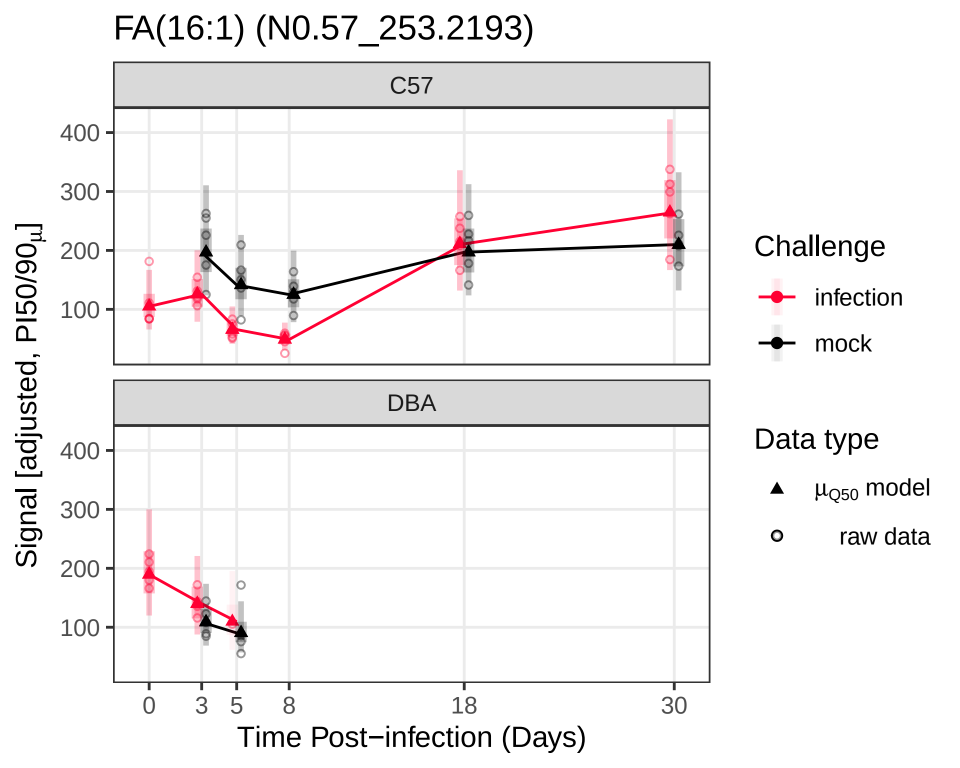

FA(16:1) (N0.57_253.2193)
C57
DBA
400
300
200
100
Signal [adjusted, PI50/90μ]
0
3
5
8
18
30
Time Post−infection (Days)
Challenge
infection
mock
Data type
μQ50 model
raw data

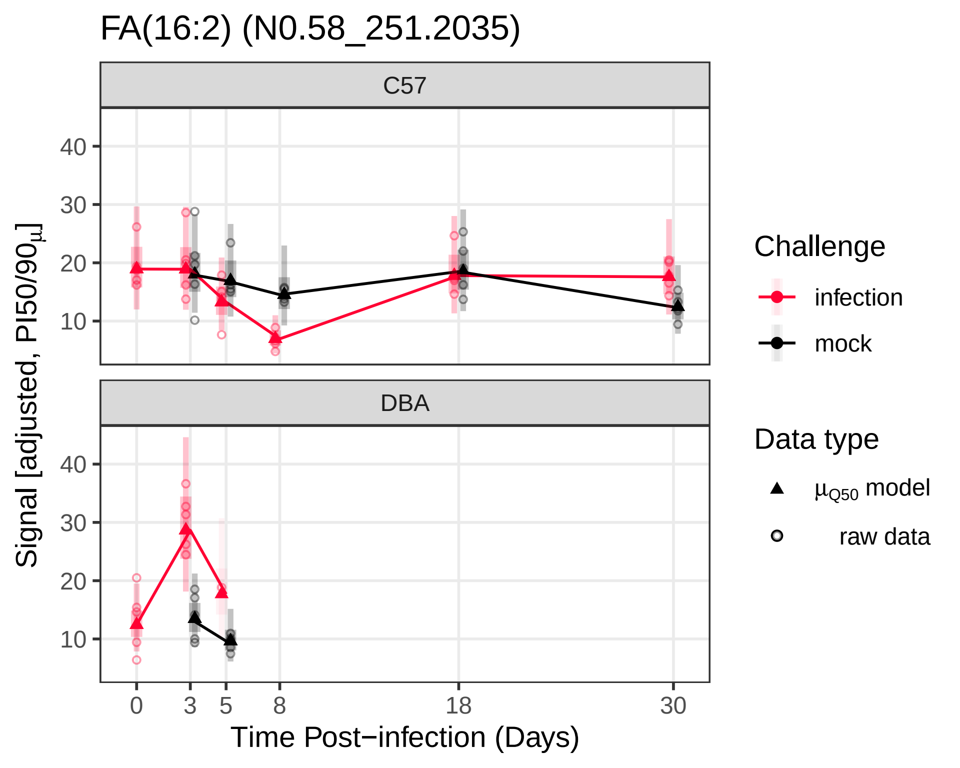

FA(16:2) (N0.58_251.2035)
C57
DBA
Signal [adjusted, PI50/90μ]
40
30
20
10
0
3
5
8
18
30
Time Post−infection (Days)
Challenge
infection
mock
Data type
μQ50 model
raw data

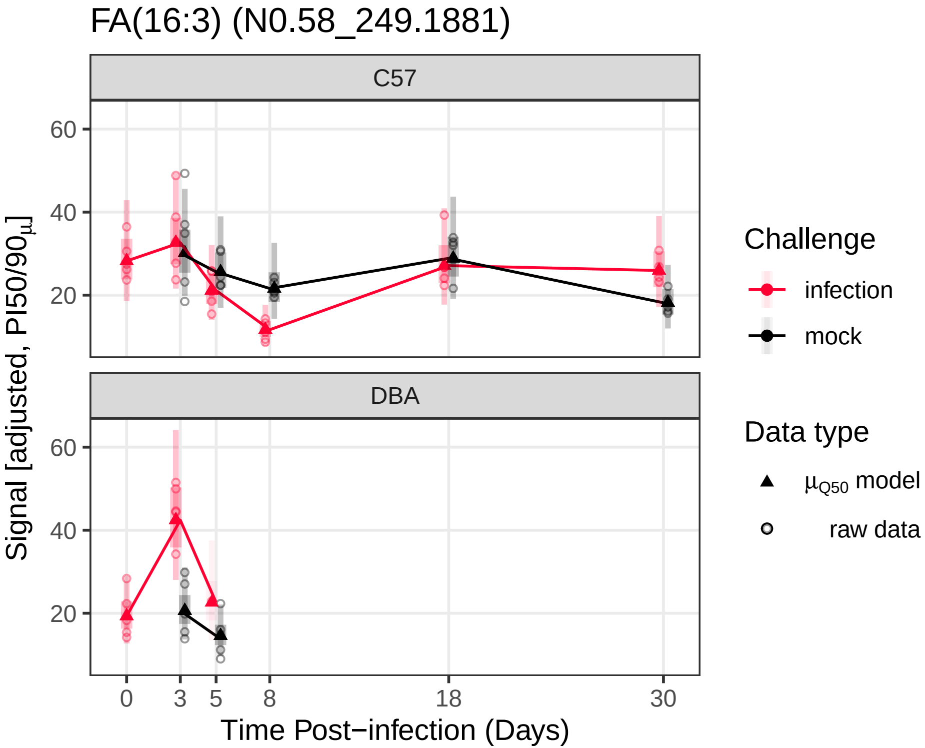
FA(16:3) (N0.58_249.1881)
C57
DBA
60
40
20
0
3
5
8
18
30
Signal [adjusted, PI50/90μ]
Time Post−infection (Days)
Challenge
infection
mock
Data type
μQ50 model
raw data

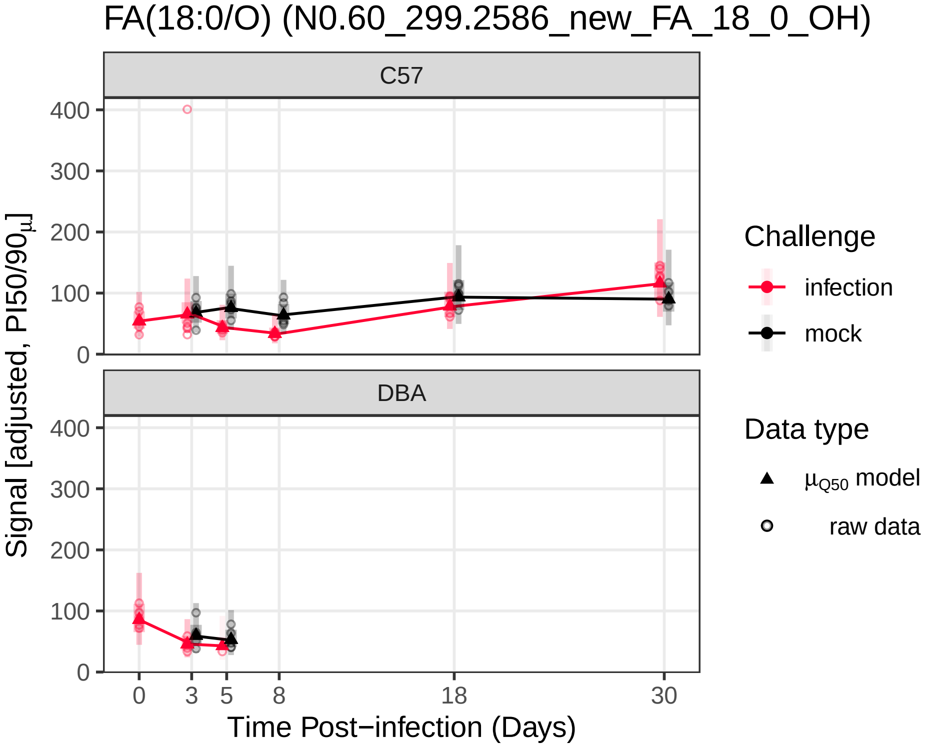
FA(18:0/O) (N0.60_299.2586_new_FA_18_0_OH)
C57
DBA
400
300
200
100
0
0
3
5
8
18
30
Time Post−infection (Days)
Signal [adjusted, PI50/90μ]
Challenge
infection
mock
Data type
μQ50 model
raw data

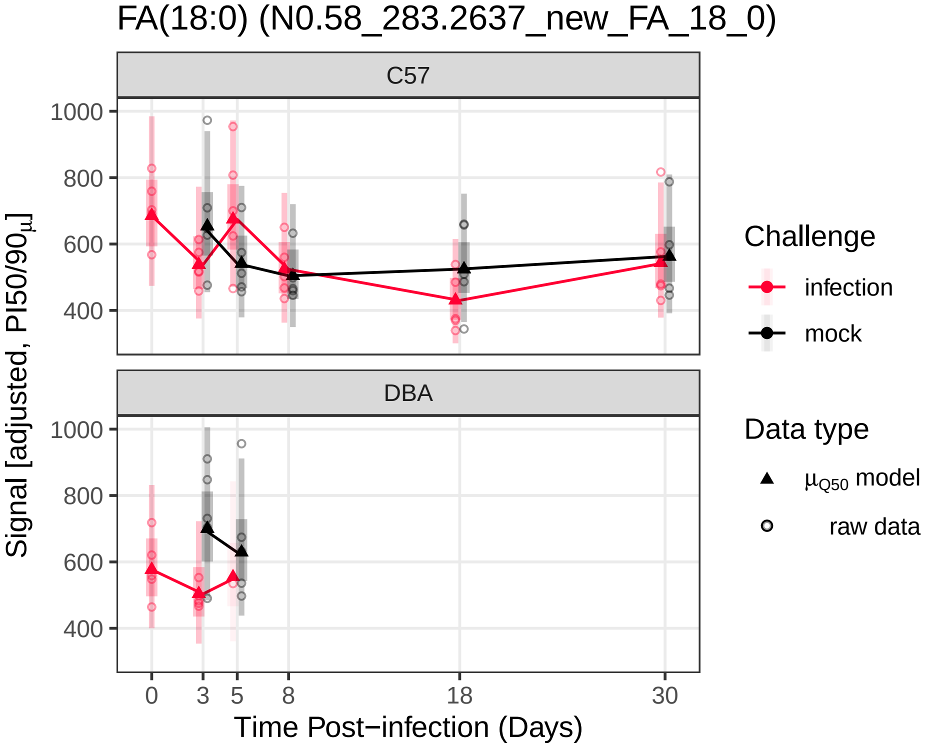

FA(18:0) (N0.58_283.2637_new_FA_18_0)
C57
DBA
1000
800
600
400
Signal [adjusted, PI50/90μ]
0
3
5
8
18
30
Time Post−infection (Days)
Challenge
infection
mock
Data type
μQ50 model
raw data

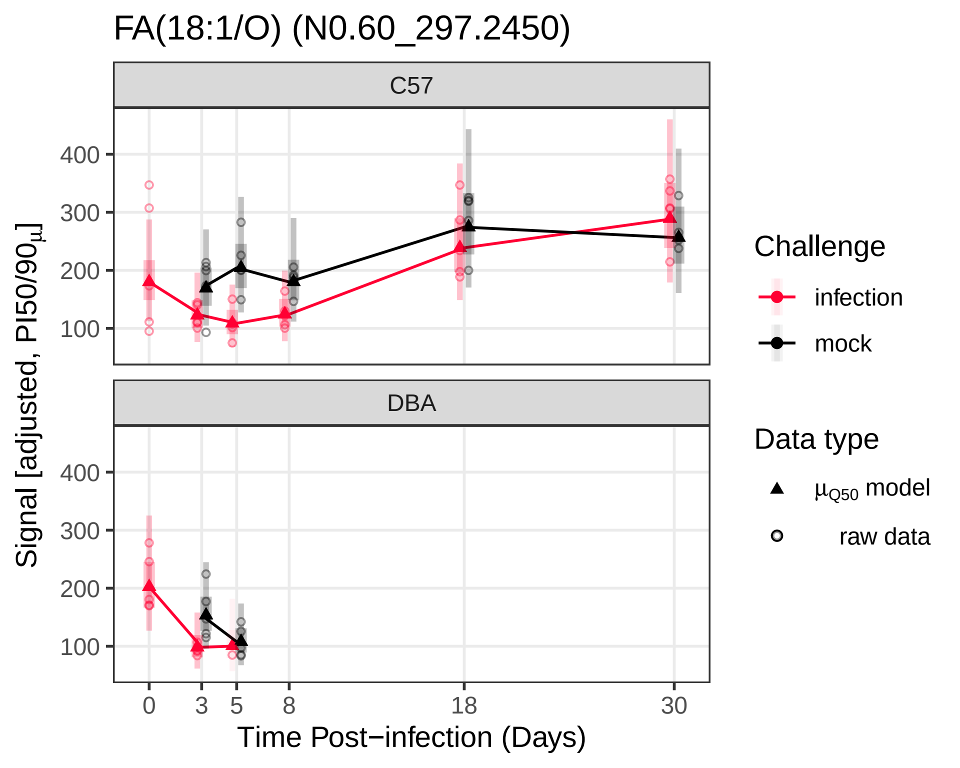

FA(18:1/O) (N0.60_297.2450)
C57
DBA
400
300
200
100
0
3
5
8
18
30
Time Post−infection (Days)
Signal [adjusted, PI50/90μ]
Challenge
infection
mock
Data type
μQ50 model
raw data

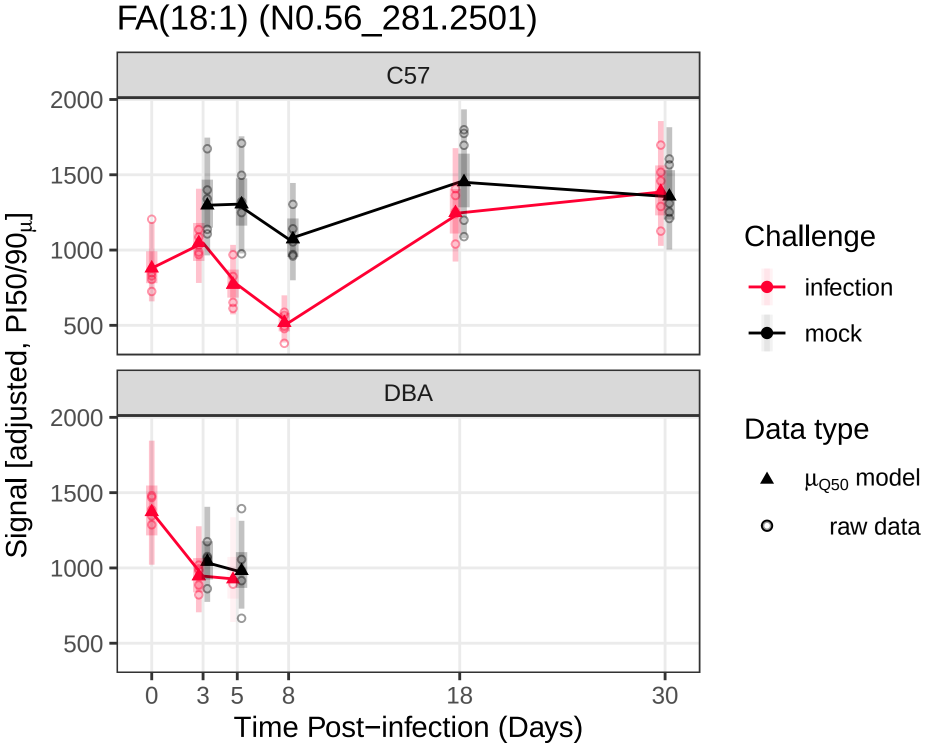
FA(18:1) (N0.56_281.2501)
C57
DBA
2000
1500
1000
500
0
3
5
8
18
30
Signal [adjusted, PI50/90μ]
Time Post−infection (Days)
Challenge
infection
mock
Data type
μQ50 model
raw data

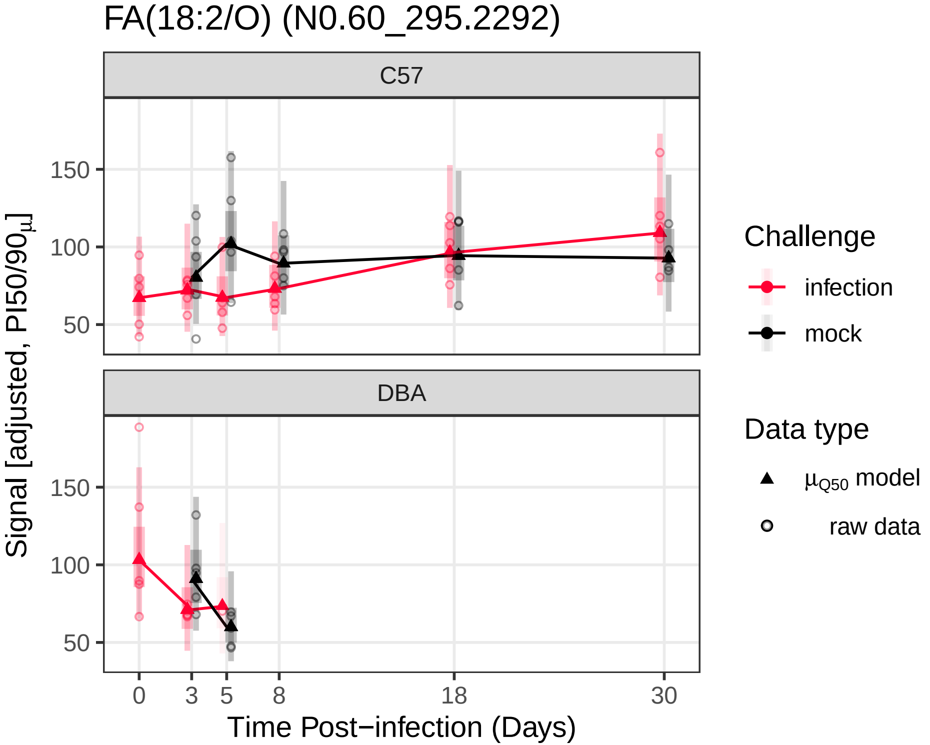
FA(18:2/O) (N0.60_295.2292)
C57
DBA
150
100
50
150
100
50
0
3
5
8
18
30
Time Post−infection (Days)
Signal [adjusted, PI50/90μ]
Challenge
infection
mock
Data type
μQ50 model
raw data

FA(18:2) (N0.57_279.2347)

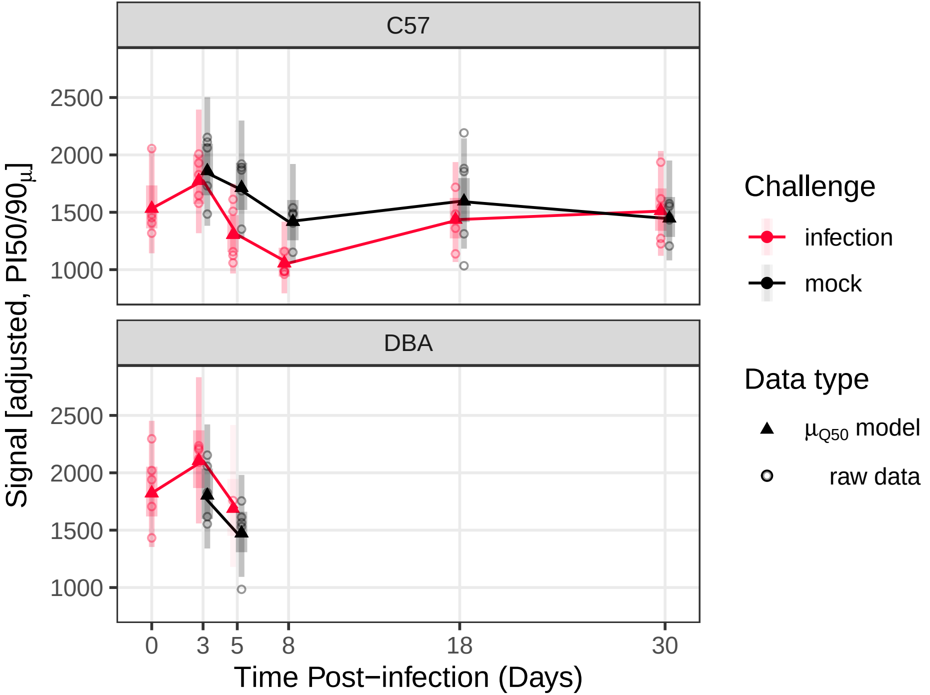

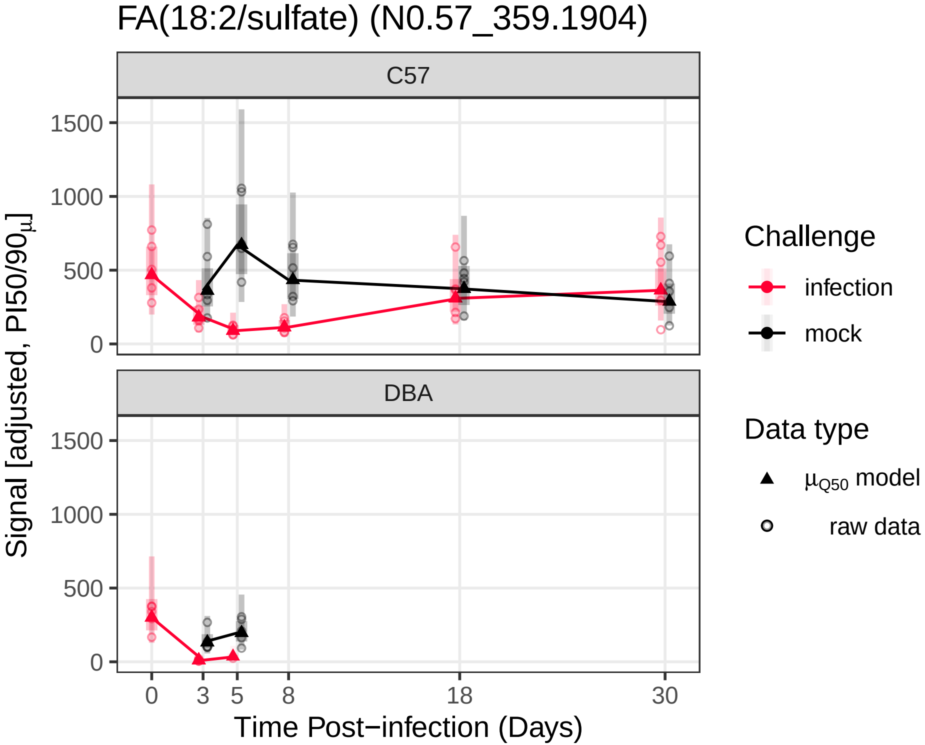
FA(18:2/sulfate) (N0.57_359.1904)
C57
DBA
1500
1000
500
0
0
3
5
8
18
30
Signal [adjusted, PI50/90μ]
Time Post−infection (Days)
Challenge
infection
mock
Data type
μQ50 model
raw data

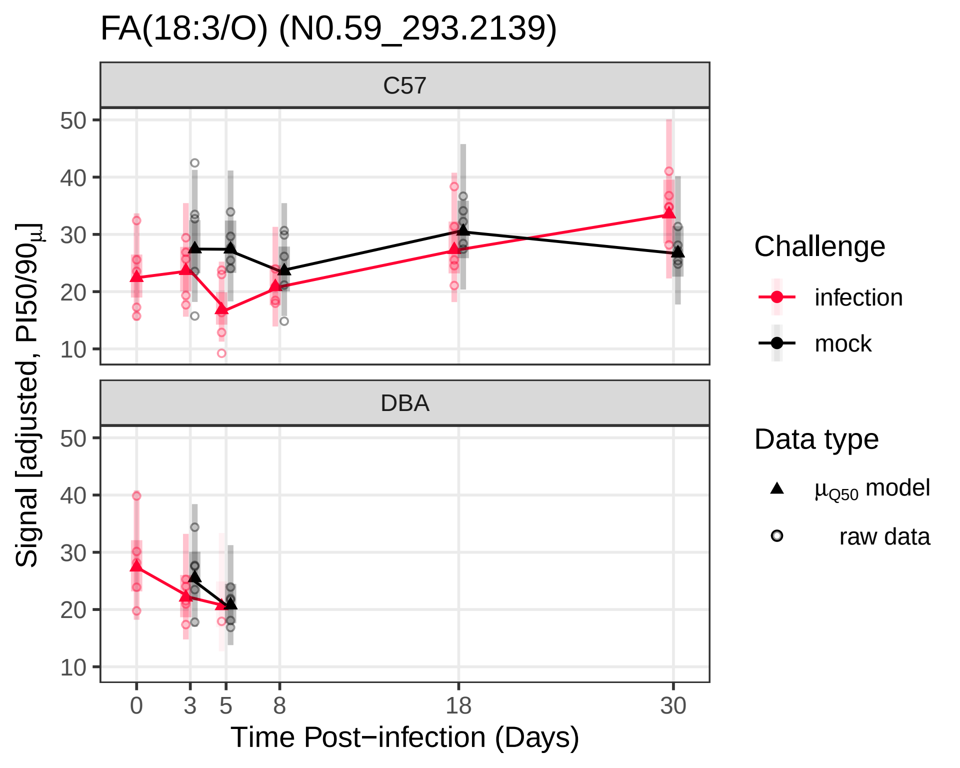

FA(18:3/O) (N0.59_293.2139)
C57
DBA
Signal [adjusted, PI50/90μ]
50
40
30
20
10
0
3
5
8
18
30
Time Post−infection (Days)
Challenge
infection
mock
Data type
μQ50 model
raw data

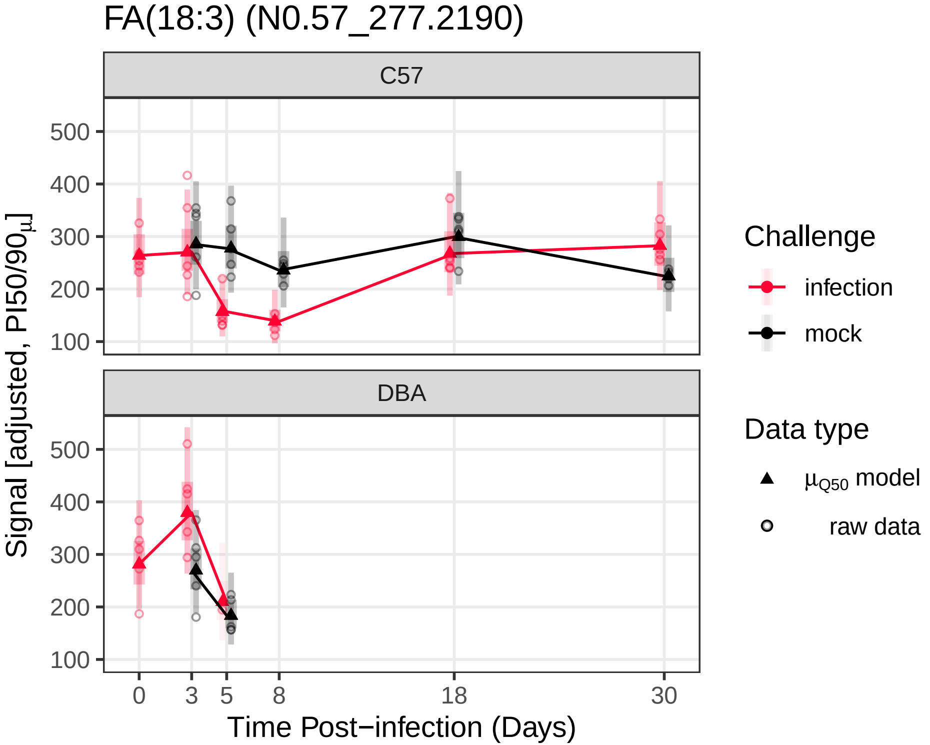
FA(18:3) (N0.57_277.2190)
C57
DBA
Signal [adjusted, PI50/90μ]
500
400
300
200
100
0
3
5
8
18
30
Time Post−infection (Days)
Challenge
infection
mock
Data type
μQ50 model
raw data

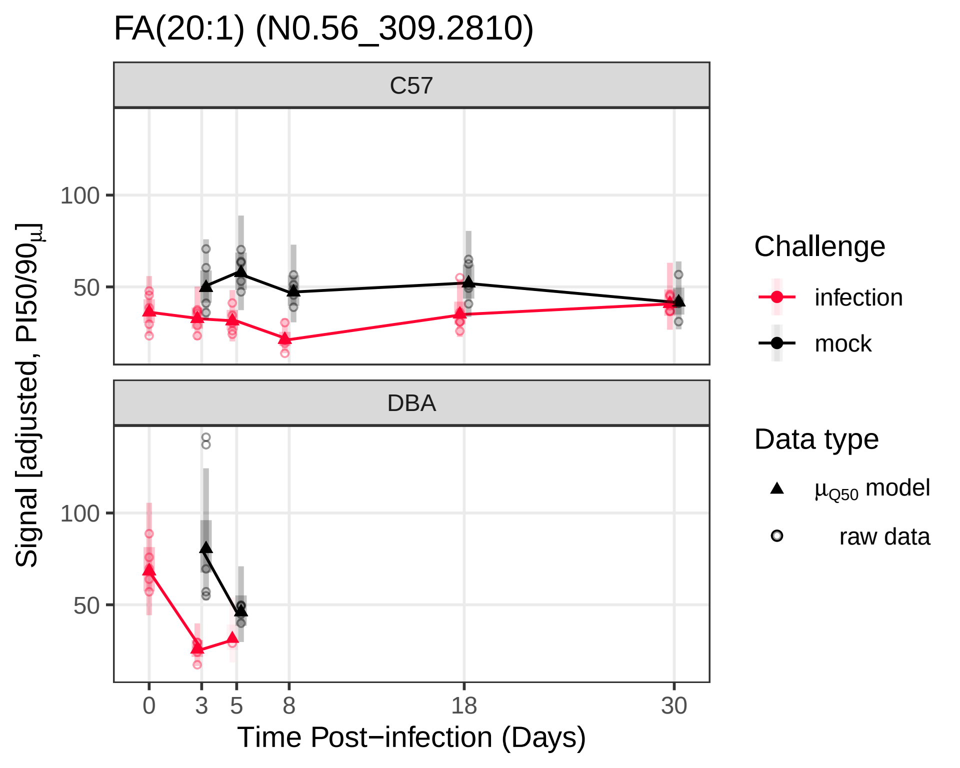

FA(20:1) (N0.56_309.2810)
C57
DBA
100
50
100
50
Signal [adjusted, PI50/90$_\mu$]
0
3
5
8
18
30
Time Post−infection (Days)
Challenge
infection
mock
Data type
μ_{Q50} model
raw data

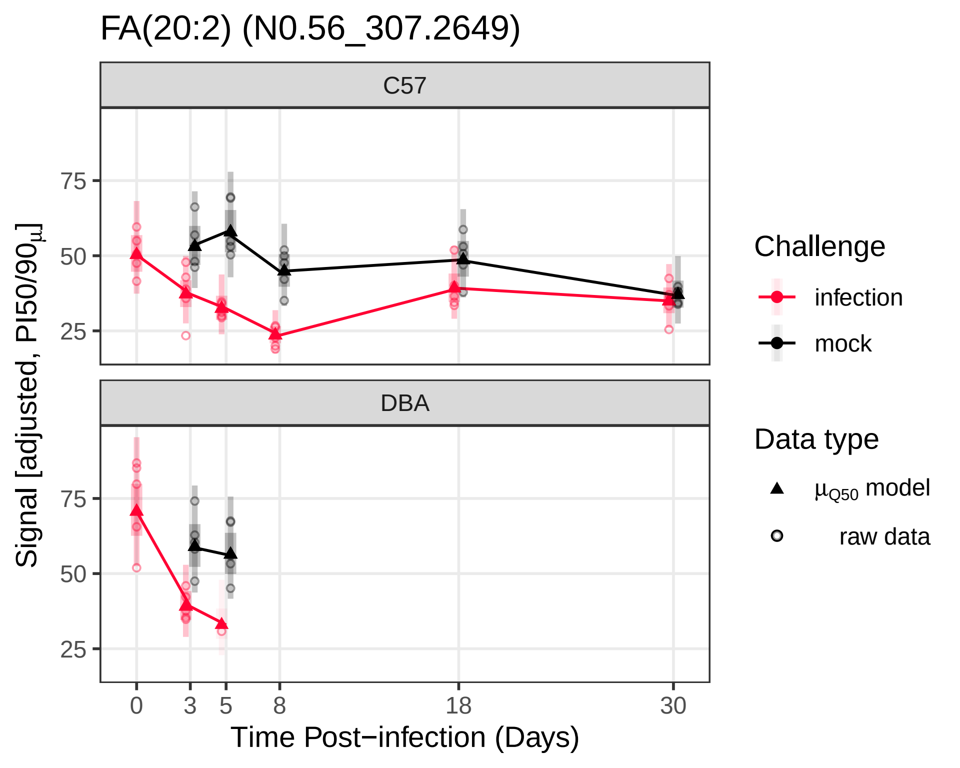
FA(20:2) (N0.56_307.2649)
C57
DBA
Signal [adjusted, PI50/90$_{\mu}$]
Time Post−infection (Days)
0
3
5
8
18
30
25
50
75
Challenge
infection
mock
Data type
μ_{Q50} model
raw data

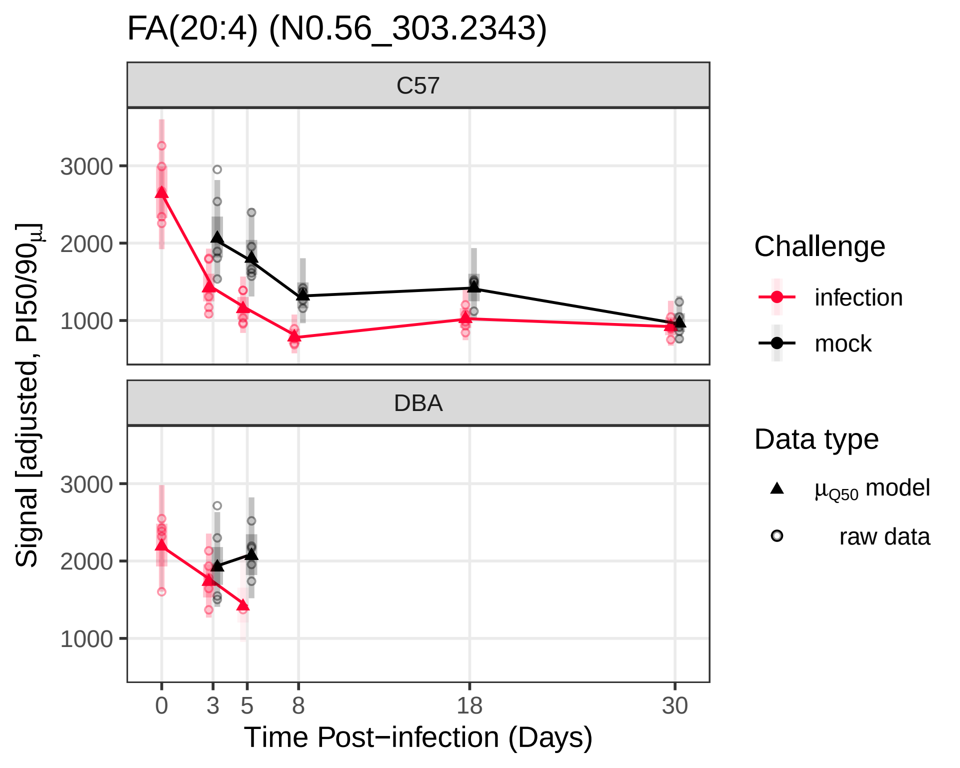
FA(20:4) (N0.56_303.2343)
C57
DBA
Signal [adjusted, PI50/90μ]
3000
2000
1000
0
3
5
8
18
30
Time Post−infection (Days)
Challenge
infection
mock
Data type
μQ50 model
raw data

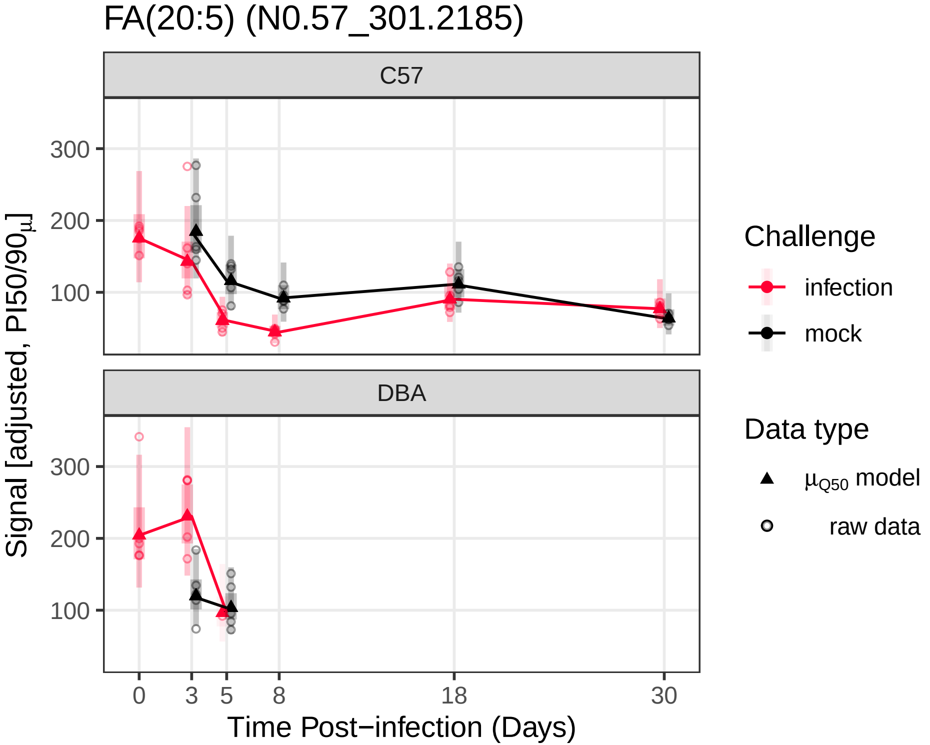

FA(20:5) (N0.57_301.2185)
C57
DBA
Signal [adjusted, PI50/90μ]
300
200
100
0
3
5
8
18
30
Time Post−infection (Days)
Challenge
infection
mock
Data type
μQ50 model
raw data

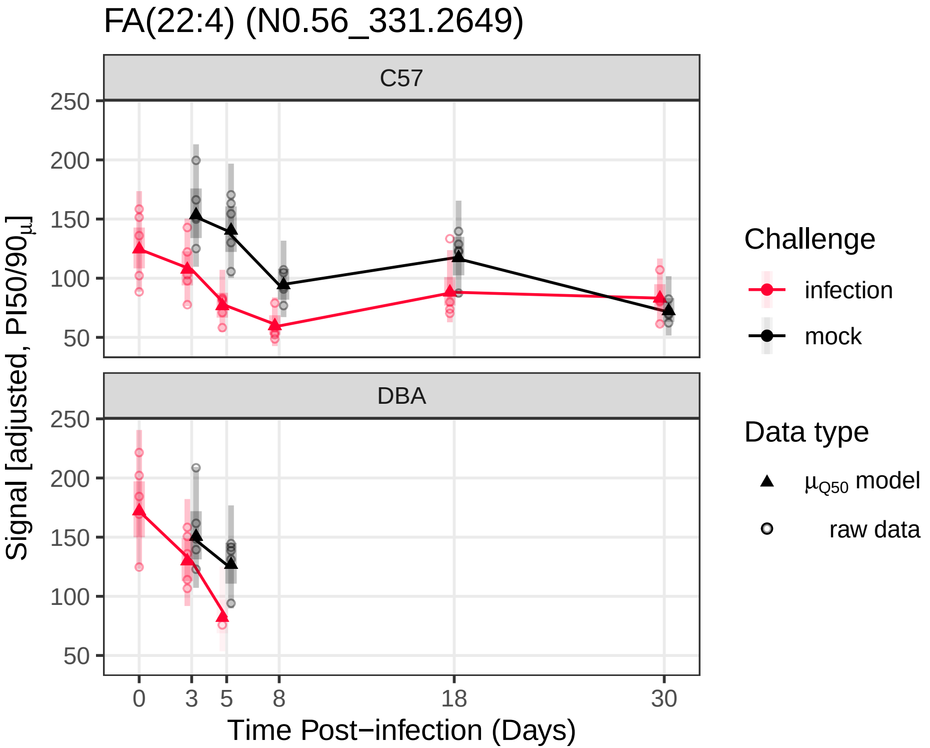
FA(22:4) (N0.56_331.2649)
C57
DBA
250
200
150
100
50
Signal [adjusted, PI50/90$_{\mu}$]
0
3
5
8
18
30
Time Post−infection (Days)
Challenge
infection
mock
Data type
μ_{Q50} model
raw data

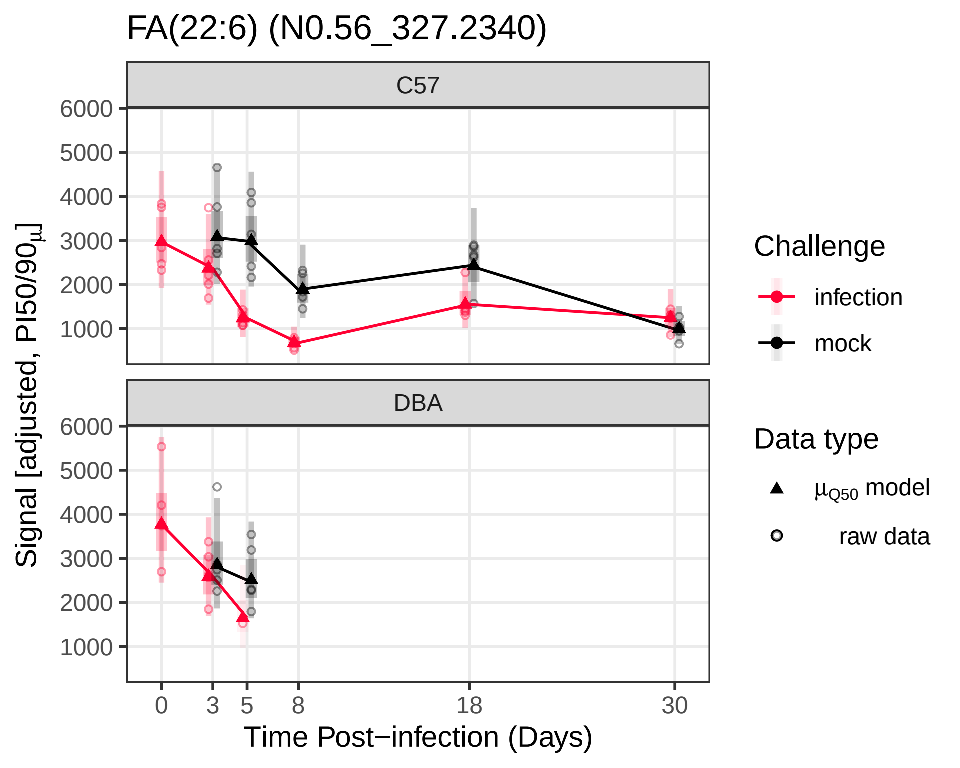

FA(22:6) (N0.56_327.2340)
C57
DBA
6000
5000
4000
3000
2000
1000
Signal [adjusted, PI50/90$_\mu$]
0
3
5
8
18
30
Time Post−infection (Days)
Challenge
infection
mock
Data type
μ_{Q50} model
raw data

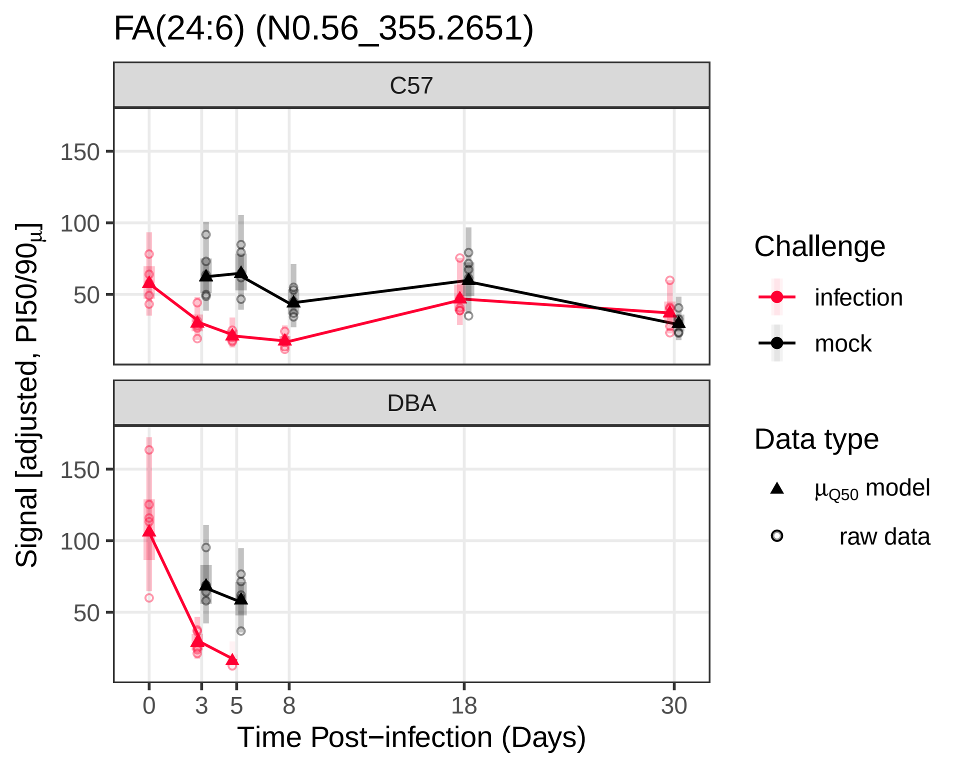
FA(24:6) (N0.56_355.2651)
C57
DBA
150
100
50
0
3
5
8
18
30
Signal [adjusted, PI50/90$_\mu$]
Time Post−infection (Days)
Challenge
infection
mock
Data type
μ_{Q50} model
raw data

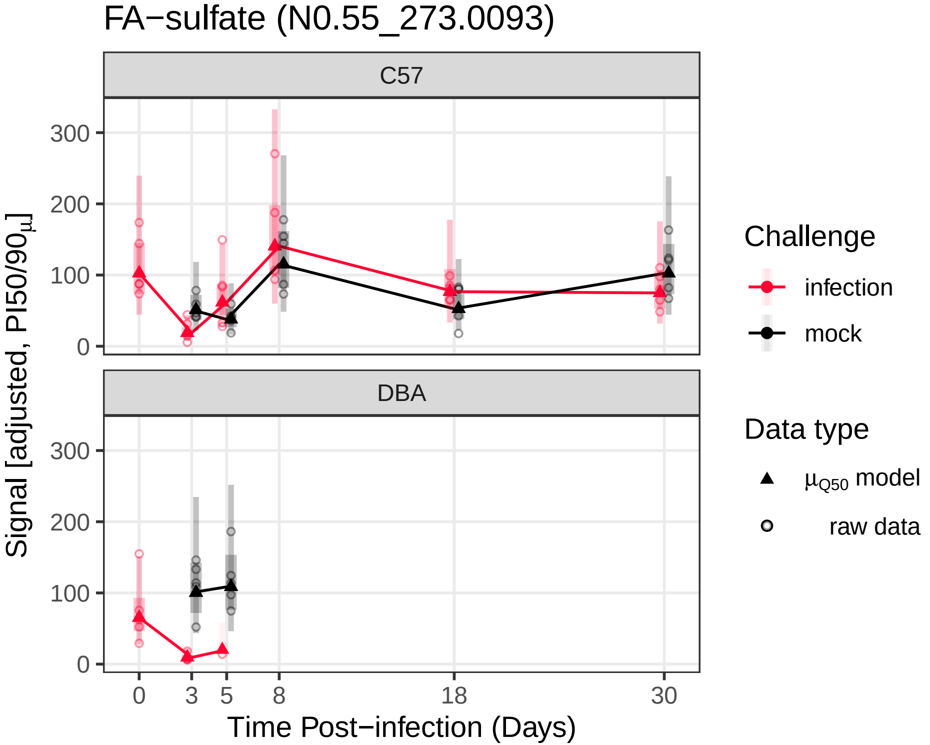
FA−sulfate (N0.55_273.0093)
C57
DBA
300
200
100
0
0
3
5
8
18
30
Time Post−infection (Days)
Signal [adjusted, PI50/90μ]
Challenge
infection
mock
Data type
μQ50 model
raw data

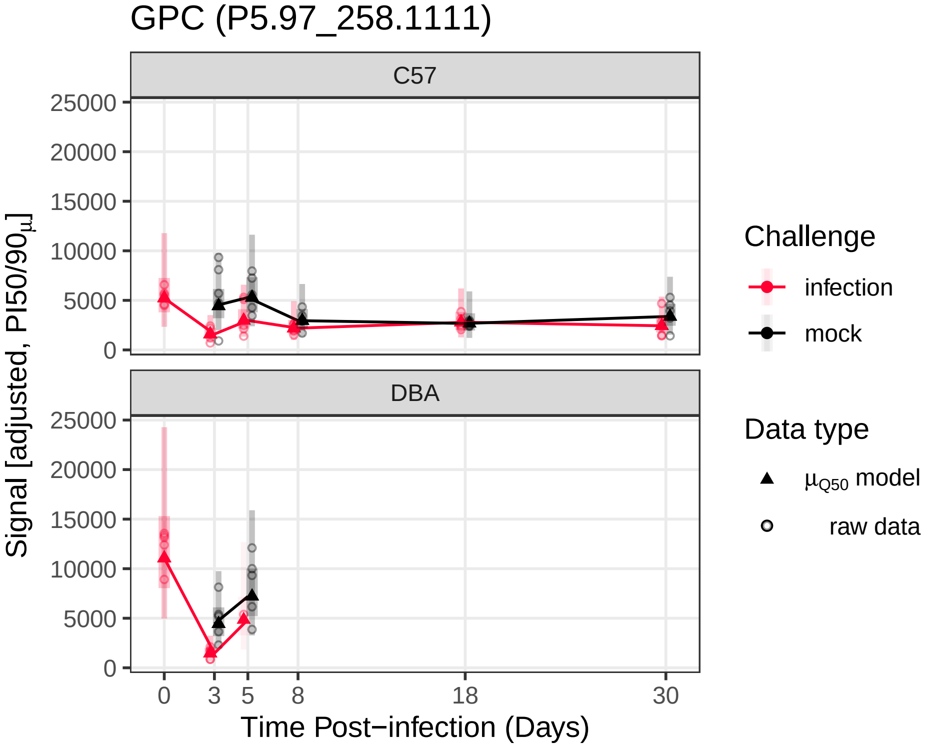
GPC (P5.97_258.1111)
C57
DBA
Signal [adjusted, PI50/90$_{\mu}$]
25000
20000
15000
10000
5000
0
0
3
5
8
18
30
Time Post−infection (Days)
Challenge
infection
mock
Data type
μ_{Q50} model
raw data

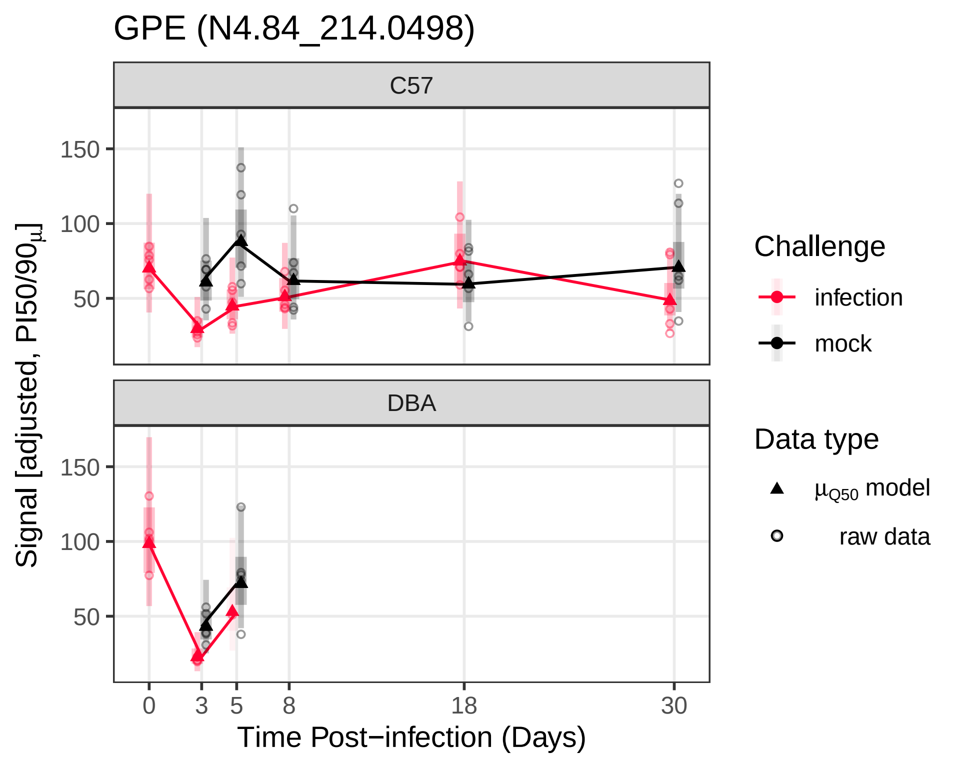
GPE (N4.84_214.0498)
C57
DBA
Signal [adjusted, PI50/90$_{\mu}$]
150
100
50
0
3
5
8
18
30
Time Post−infection (Days)
Challenge
infection
mock
Data type
μ_{Q50} model
raw data

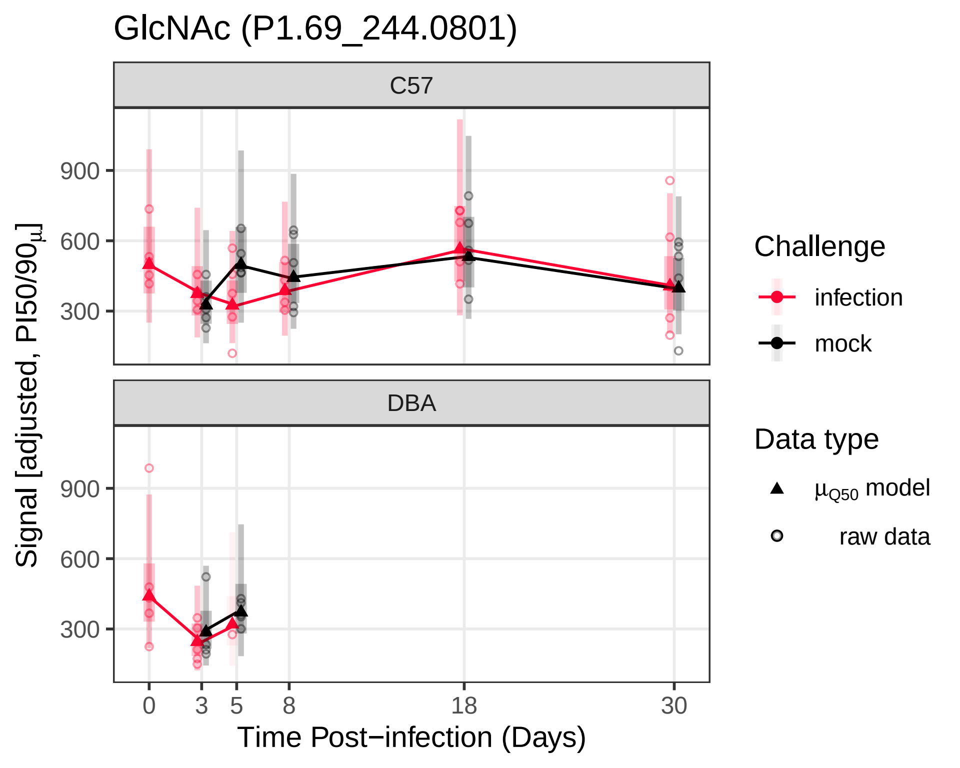

GlcNAc (P1.69_244.0801)
C57
DBA
900
600
300
0
3
5
8
18
30
Signal [adjusted, PI50/90μ]
Time Post−infection (Days)
Challenge
infection
mock
Data type
μQ50 model
raw data

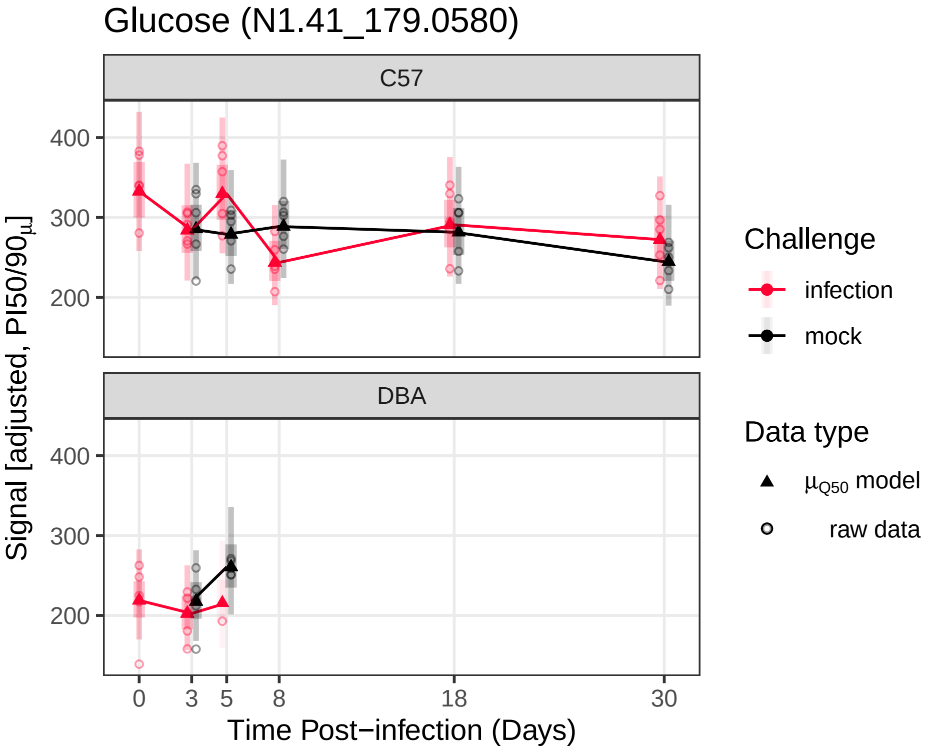

Glucose (N1.41_179.0580)
C57
DBA
400
300
200
Signal [adjusted, PI50/90$_{\mu}$]
0
3
5
8
18
30
Time Post−infection (Days)
Challenge
infection
mock
Data type
μ_{Q50} model
raw data

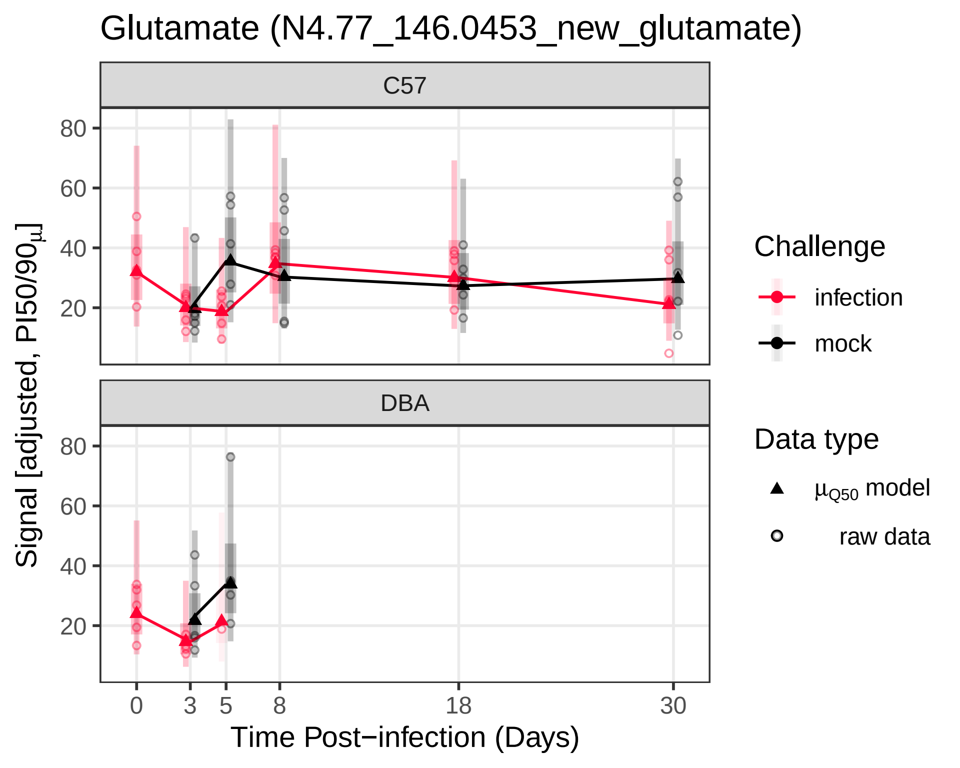

Glutamate (N4.77_146.0453_new_glutamate)
C57
DBA
Signal [adjusted, PI50/90μ]
Time Post−infection (Days)
0
3
5
8
18
30
20
40
60
80
Challenge
infection
mock
Data type
μQ50 model
raw data

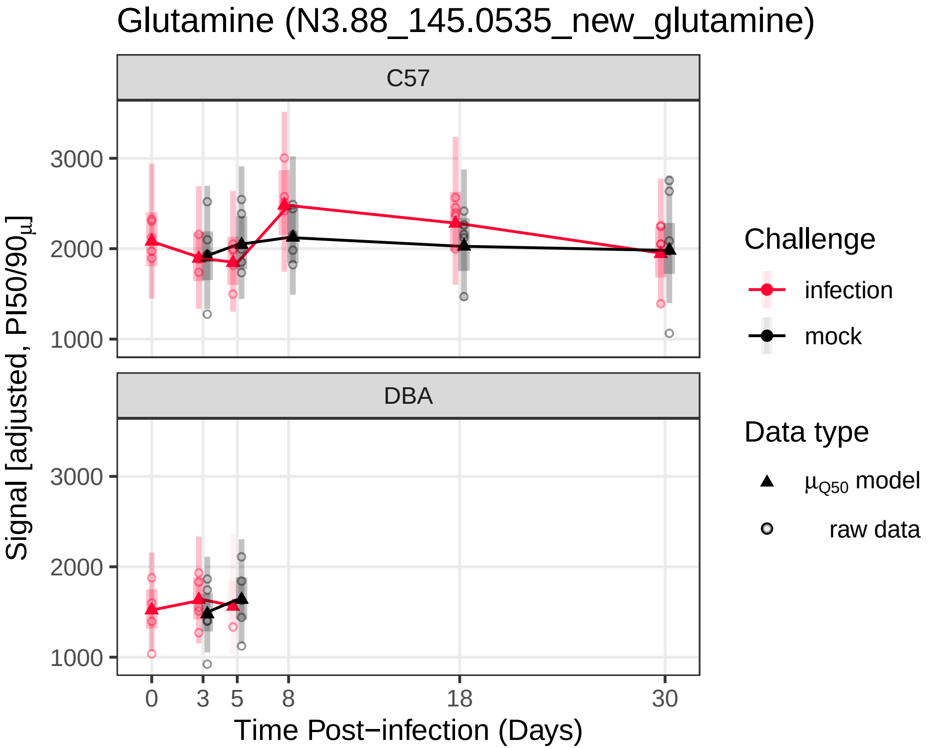

Glutamine (N3.88_145.0535_new_glutamine)
C57
DBA
3000
2000
1000
Signal [adjusted, PI50/90$_\mu$]
0
3
5
8
18
30
Time Post−infection (Days)
Challenge
infection
mock
Data type
μ_{Q50} model
raw data

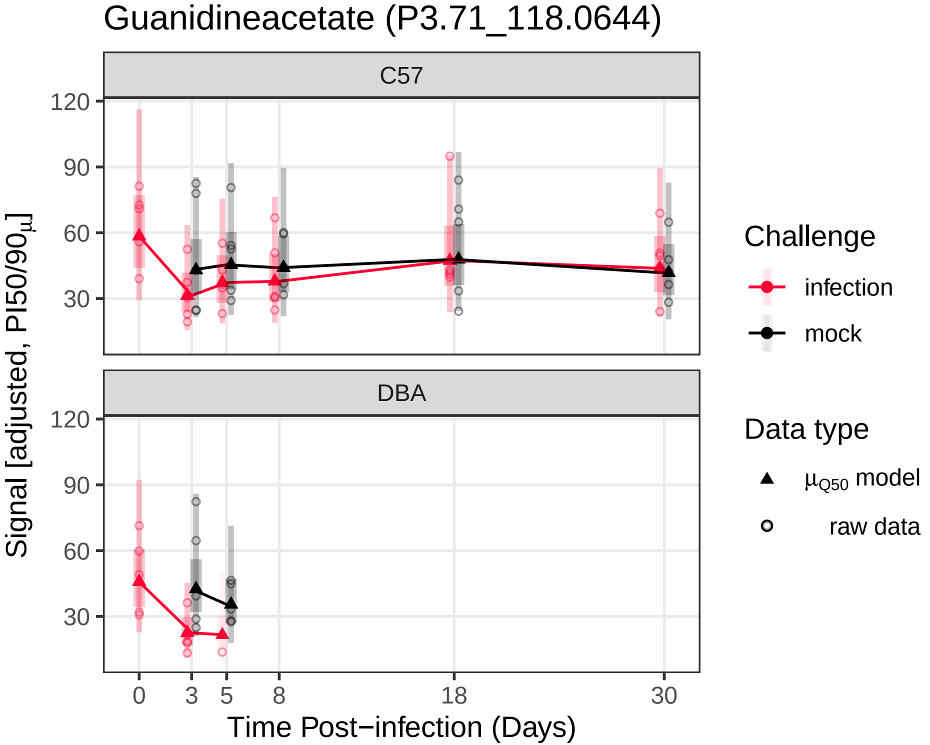
Guanidineacetate (P3.71_118.0644)
C57
DBA
120
90
60
30
0
3
5
8
18
30
Signal [adjusted, PI50/90μ]
Time Post−infection (Days)
Challenge
infection
mock
Data type
μQ50 model
raw data

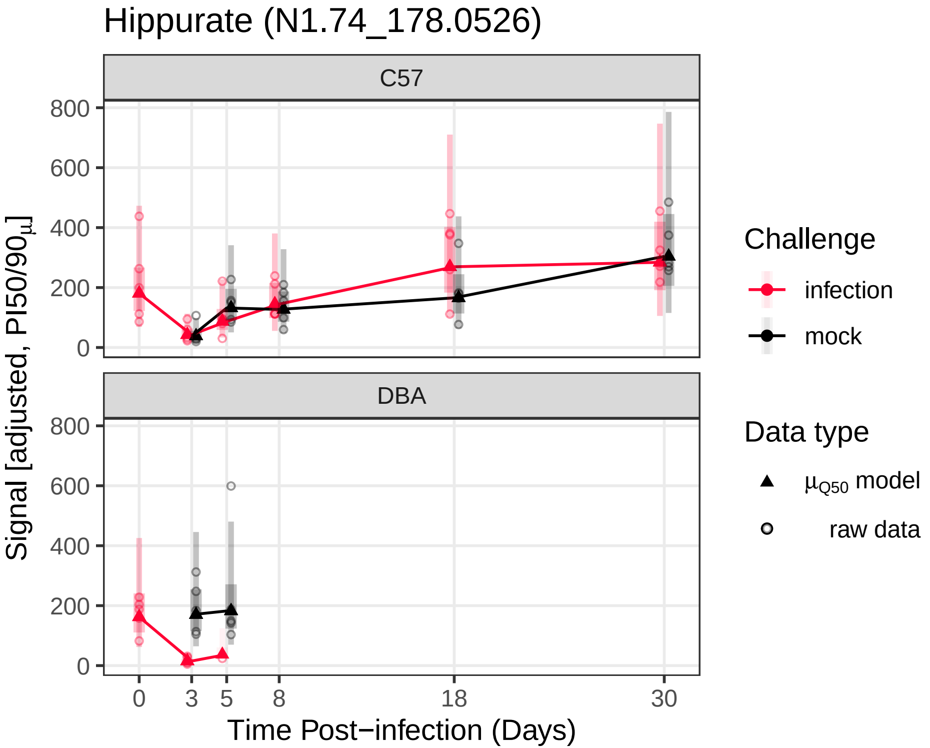
Hippurate (N1.74_178.0526)
C57
DBA
800
600
400
200
0
Signal [adjusted, PI50/90$_{\mu}$]
0
3
5
8
18
30
Time Post−infection (Days)
Challenge
infection
mock
Data type
μ_{Q50} model
raw data

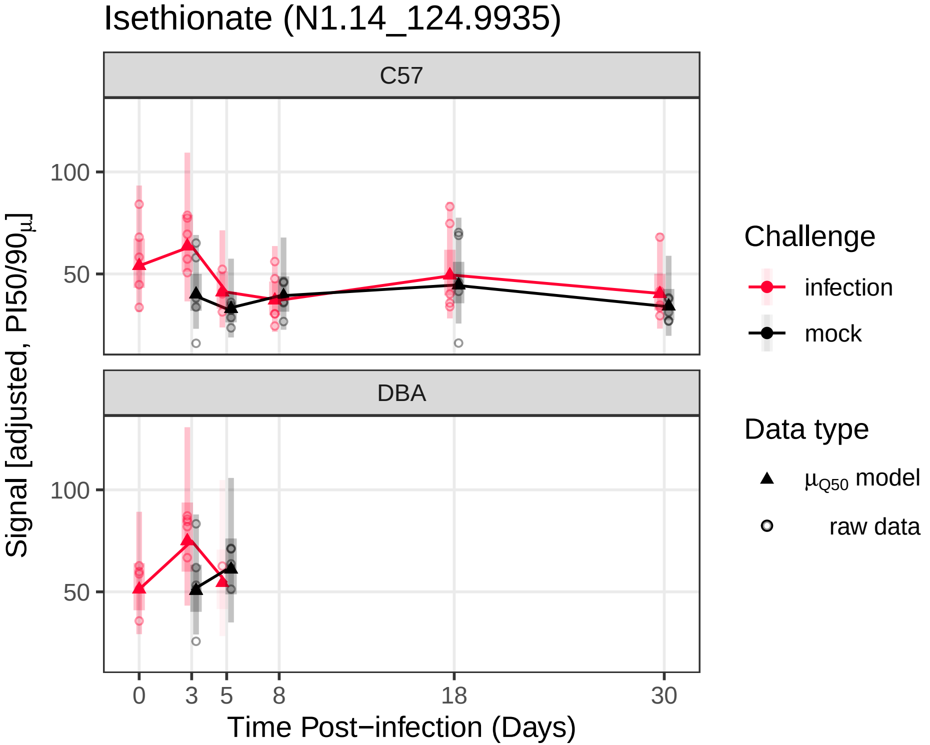
Isethionate (N1.14_124.9935)
C57
DBA
100
50
100
50
0
3
5
8
18
30
Time Post−infection (Days)
Signal [adjusted, PI50/90μ]
Challenge
infection
mock
Data type
μQ50 model
raw data

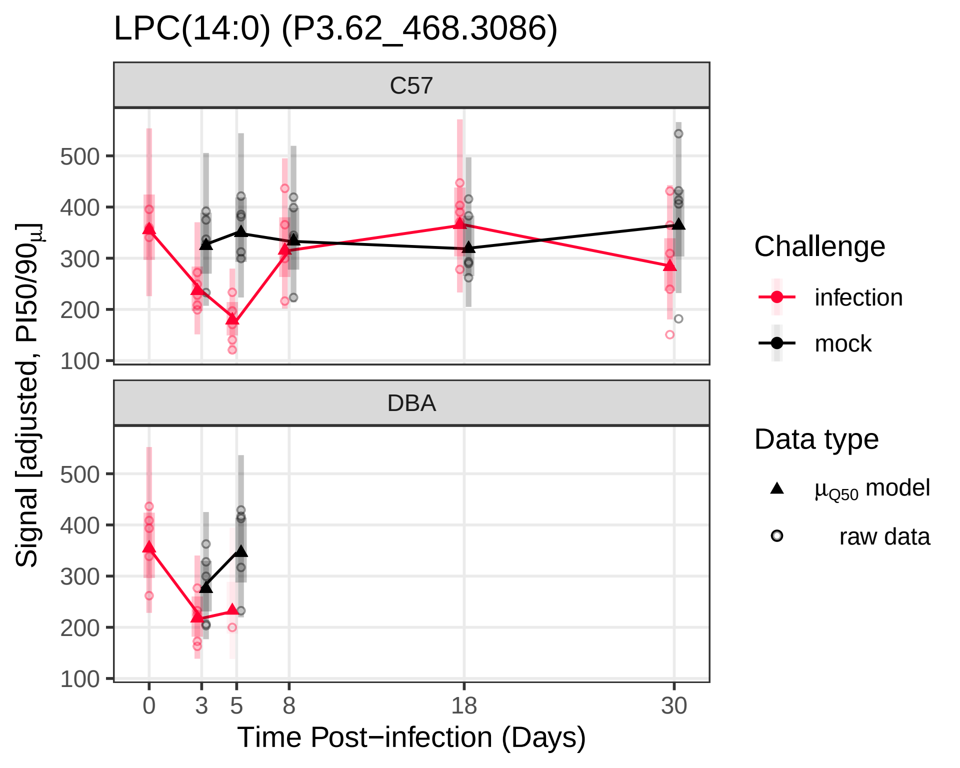

LPC(14:0) (P3.62_468.3086)
C57
DBA
Signal [adjusted, PI50/90μ]
500
400
300
200
100
0
3
5
8
18
30
Time Post−infection (Days)
Challenge
infection
mock
Data type
μQ50 model
raw data

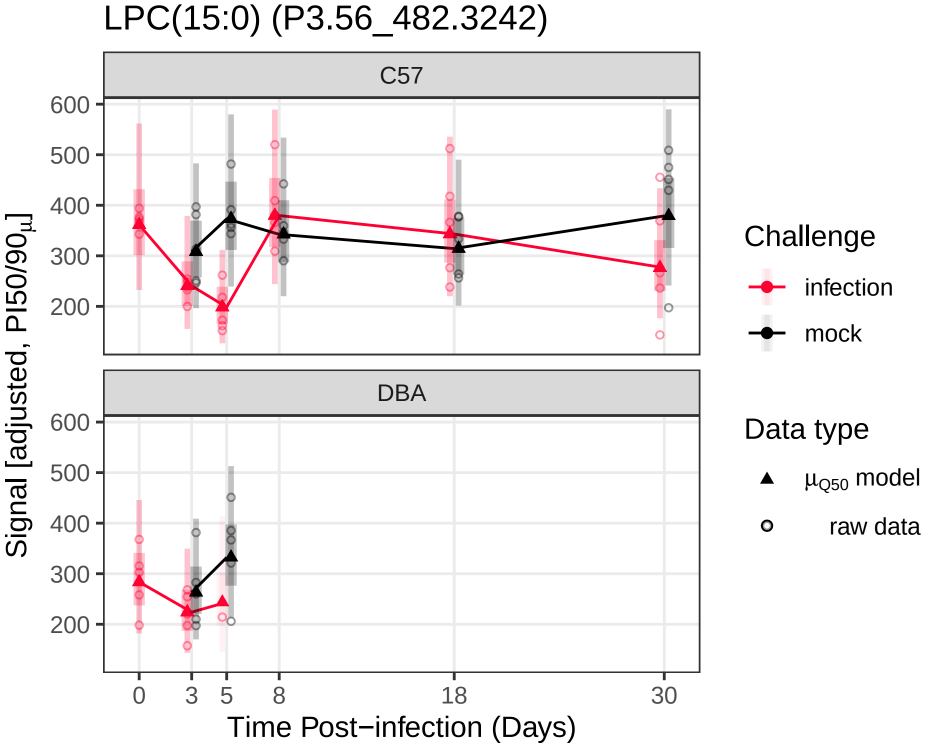

LPC(15:0) (P3.56_482.3242)
C57
DBA
Signal [adjusted, PI50/90μ]
600
500
400
300
200
Time Post−infection (Days)
0
3
5
8
18
30
Challenge
infection
mock
Data type
μQ50 model
raw data

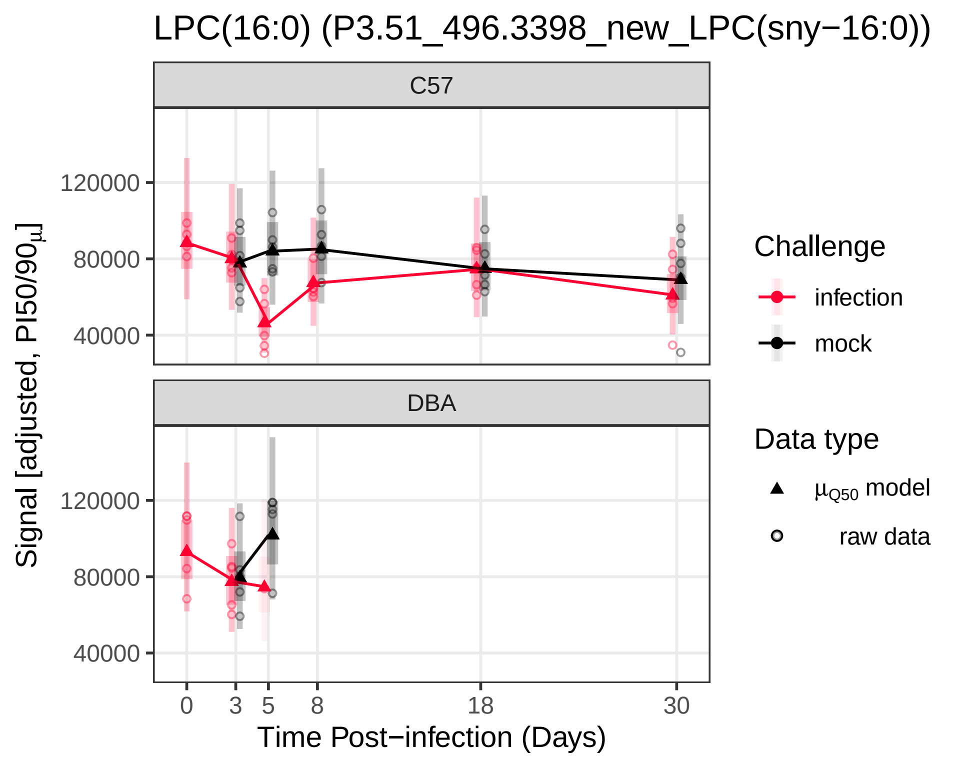

LPC(16:0) (P3.51_496.3398_new_LPC(sny−16:0))
C57
DBA
120000
80000
40000
Signal [adjusted, PI50/90μ]
0
3
5
8
18
30
Time Post−infection (Days)
Challenge
infection
mock
Data type
μQ50 model
raw data

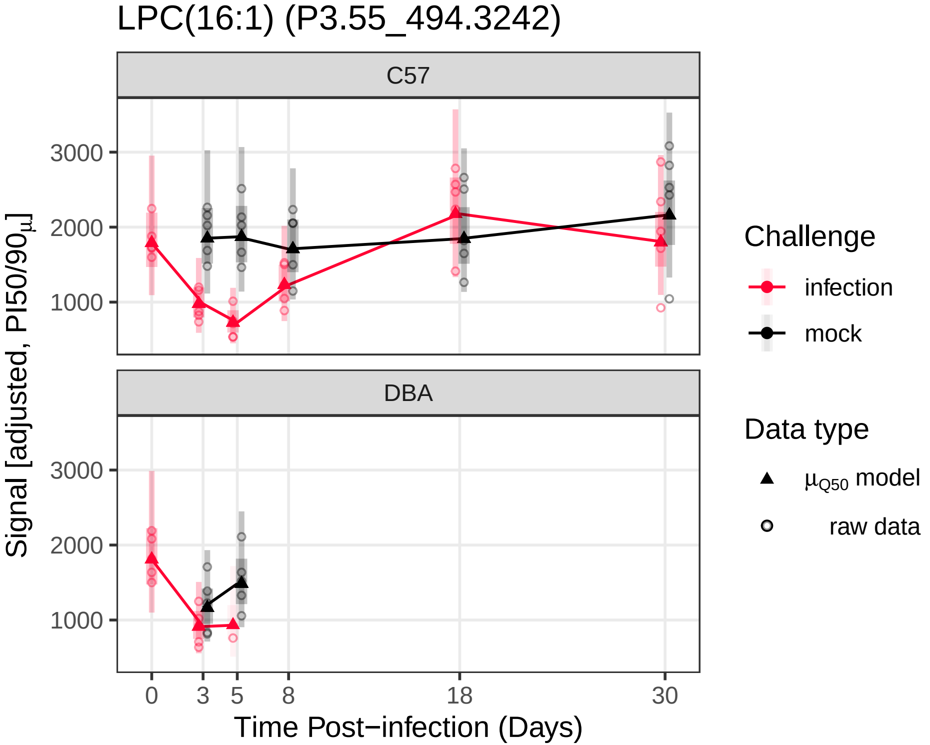
LPC(16:1) (P3.55_494.3242)
C57
DBA
Signal [adjusted, PI50/90$_\mu$]
3000
2000
1000
0
3
5
8
18
30
Time Post−infection (Days)
Challenge
infection
mock
Data type
μ_{Q50} model
raw data

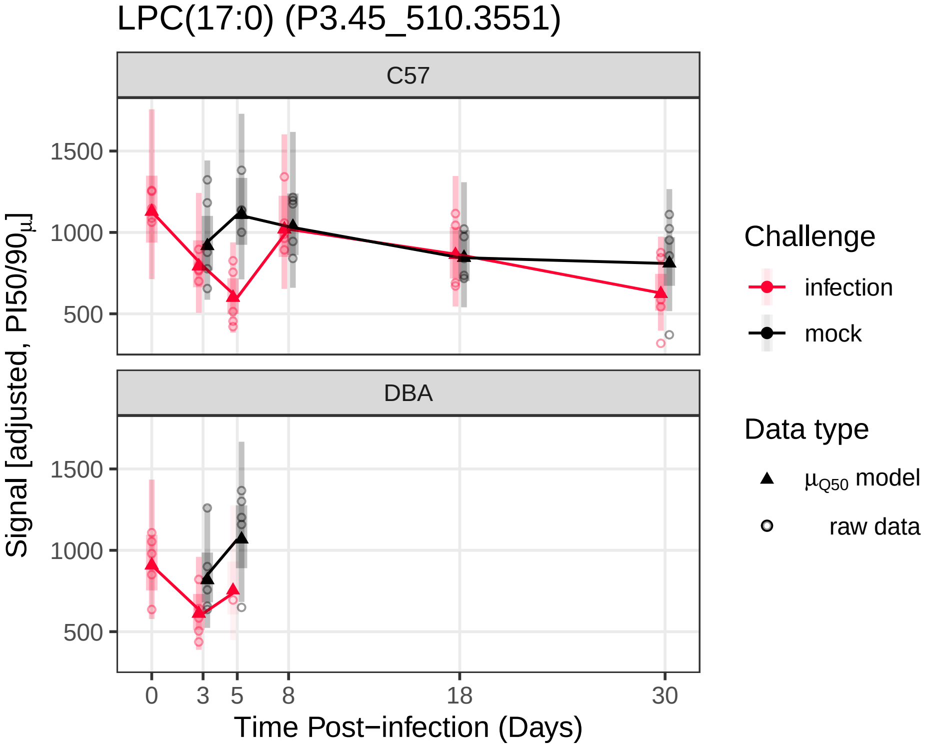

LPC(17:0) (P3.45_510.3551)
C57
DBA
1500
1000
500
Signal [adjusted, PI50/90μ]
0
3
5
8
18
30
Time Post−infection (Days)
Challenge
infection
mock
Data type
μQ50 model
raw data

LPC(17:1) (P3.49_508.3393)

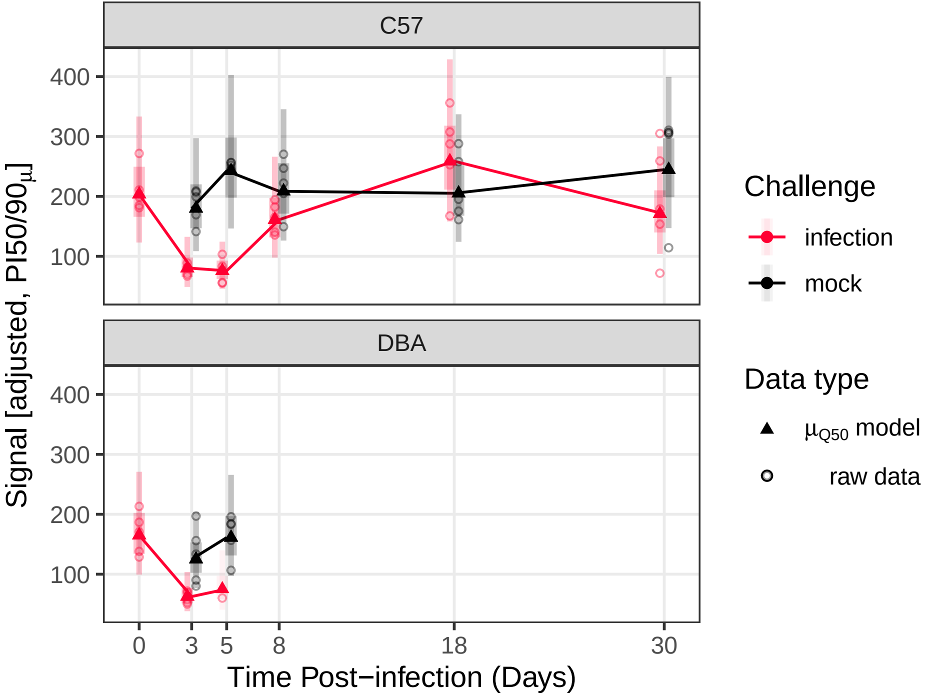

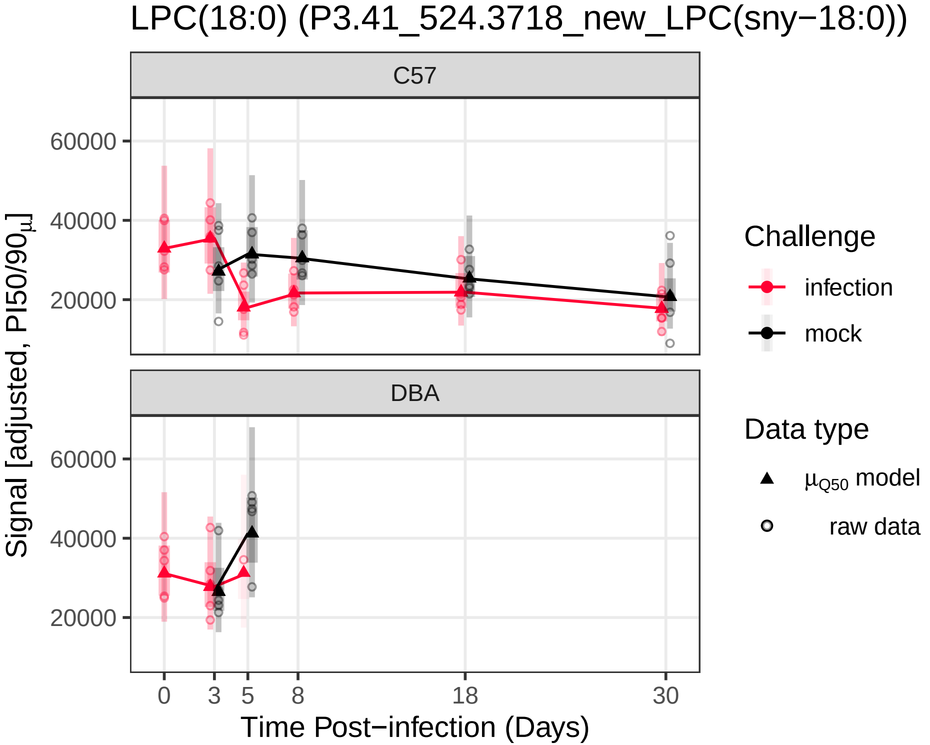
LPC(18:0) (P3.41_524.3718_new_LPC(sny−18:0))
C57
DBA
60000
40000
20000
60000
40000
20000
Signal [adjusted, PI50/90μ]
0
3
5
8
18
30
Time Post−infection (Days)
Challenge
infection
mock
Data type
μQ50 model
raw data

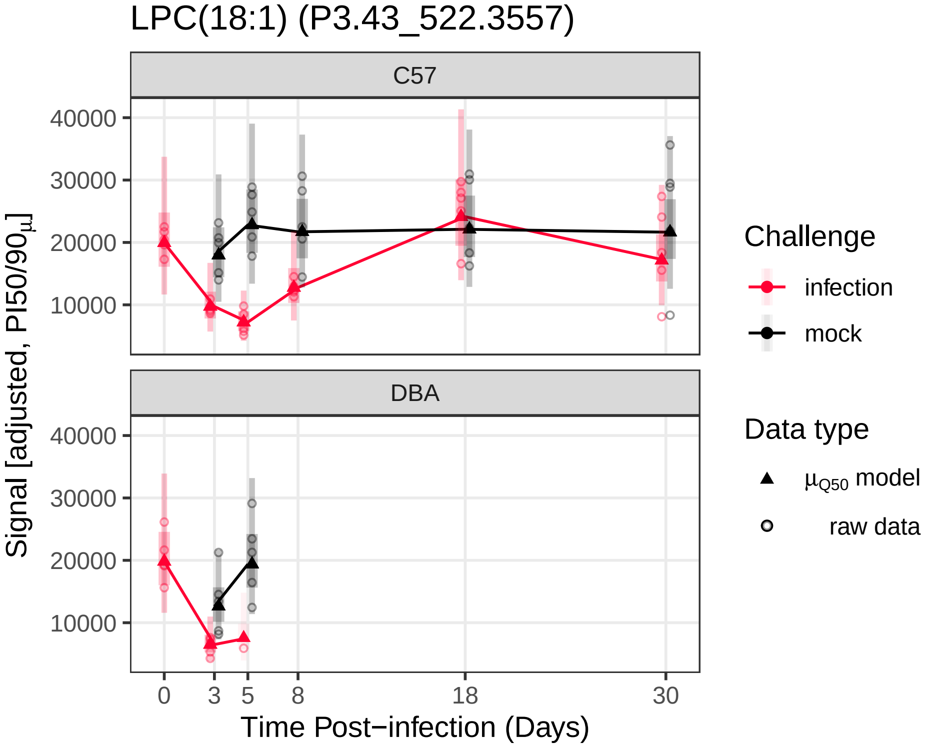

LPC(18:1) (P3.43_522.3557)
C57
DBA
40000
30000
20000
10000
Signal [adjusted, PI50/90μ]
0
3
5
8
18
30
Time Post−infection (Days)
Challenge
infection
mock
Data type
μQ50 model
raw data

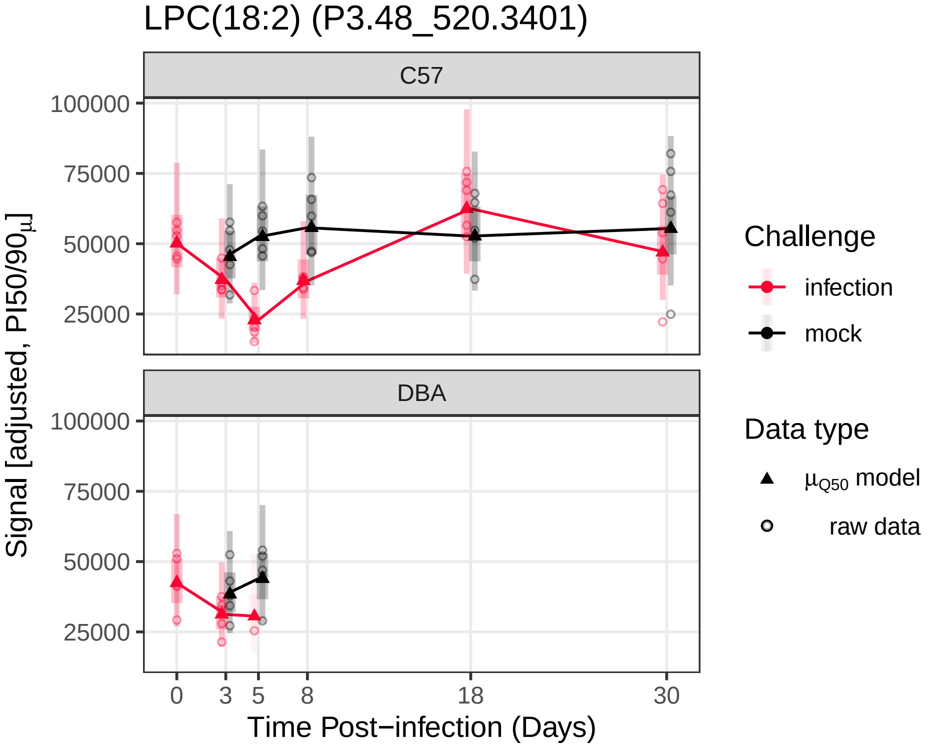
LPC(18:2) (P3.48_520.3401)
C57
DBA
Signal [adjusted, PI50/90μ]
100000
75000
50000
25000
0
3
5
8
18
30
Time Post−infection (Days)
Challenge
infection
mock
Data type
μQ50 model
raw data

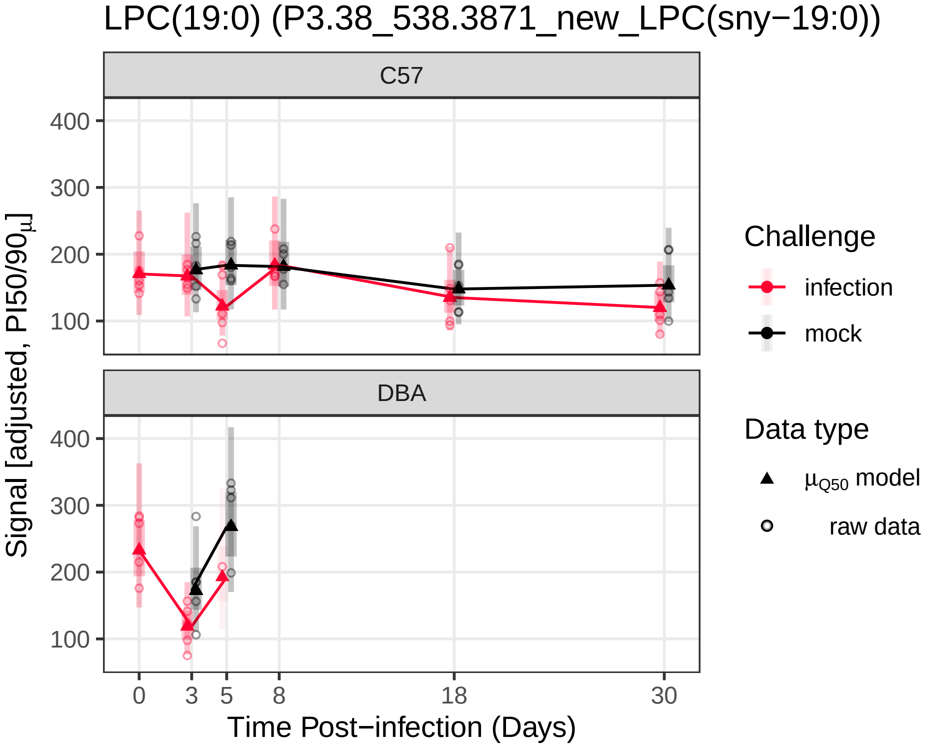
LPC(19:0) (P3.38_538.3871_new_LPC(sny−19:0))
C57
DBA
Signal [adjusted, PI50/90µ]
Time Post−infection (Days)
Challenge
infection
mock
Data type
µQ50 model
raw data

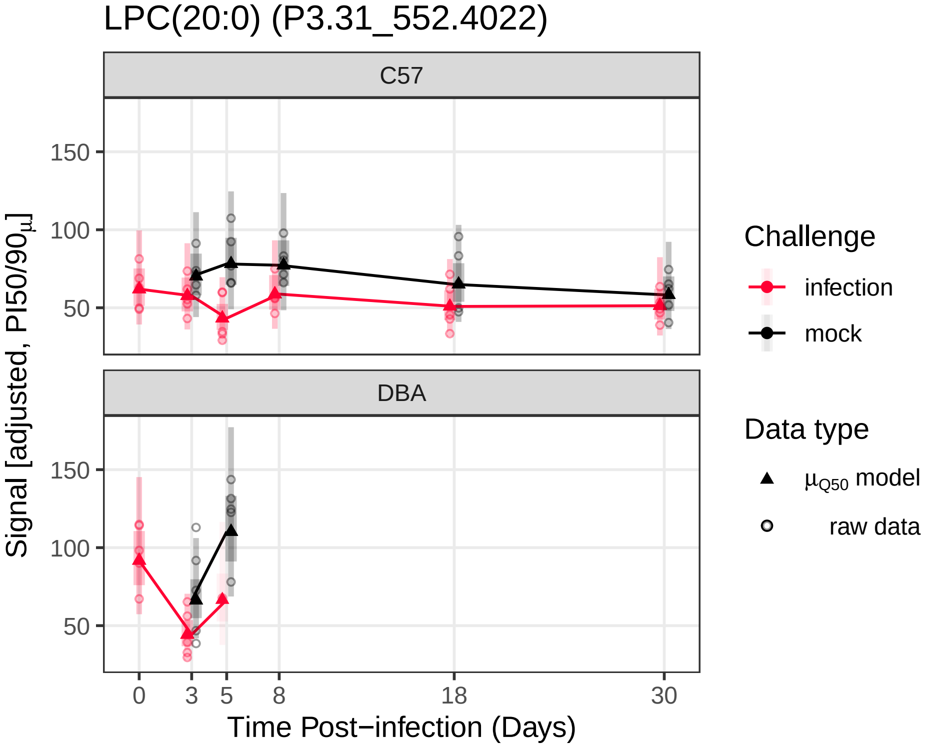

LPC(20:0) (P3.31_552.4022)
C57
DBA
Signal [adjusted, PI50/90μ]
150
100
50
0
3
5
8
18
30
Time Post−infection (Days)
Challenge
infection
mock
Data type
μQ50 model
raw data

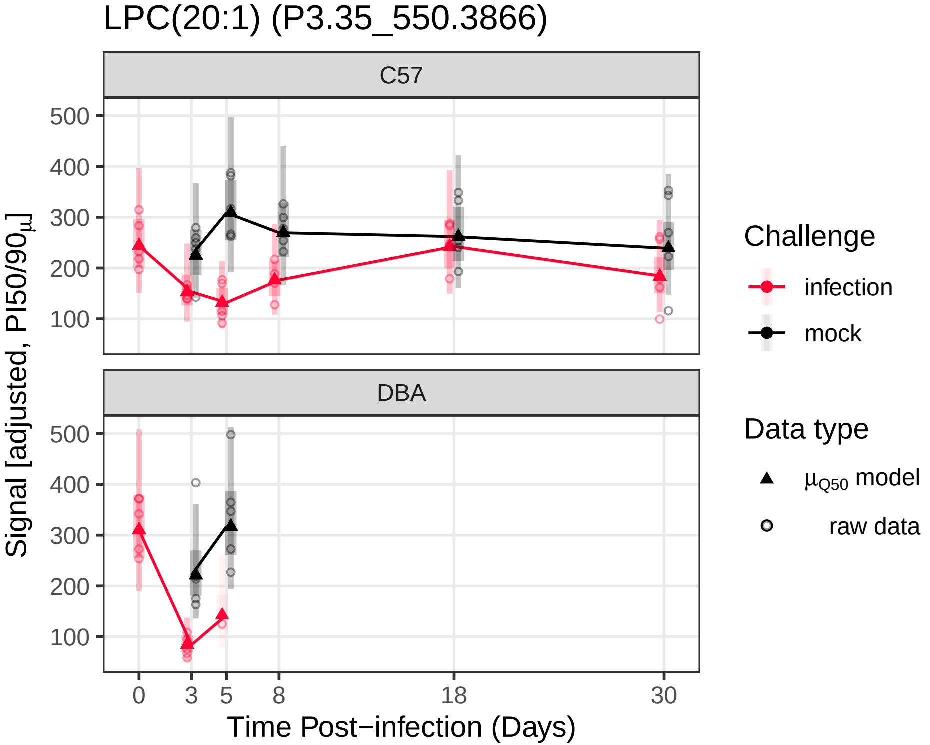

LPC(20:1) (P3.35_550.3866)
C57
DBA
500
400
300
200
100
Signal [adjusted, PI50/90$_{\mu}$]
0
3
5
8
18
30
Time Post−infection (Days)
Challenge
infection
mock
Data type
μ_{Q50} model
raw data

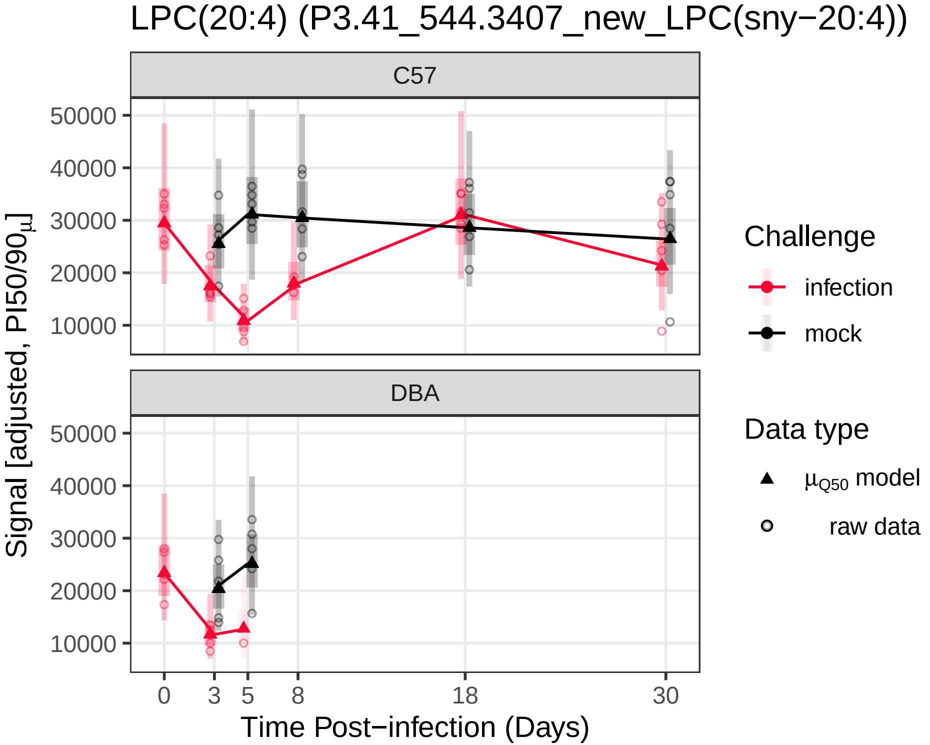

LPC(20:4) (P3.41_544.3407_new_LPC(sny−20:4))
C57
DBA
50000
40000
30000
20000
10000
Signal [adjusted, PI50/90μ]
0
3
5
8
18
30
Time Post−infection (Days)
Challenge
infection
mock
Data type
μQ50 model
raw data

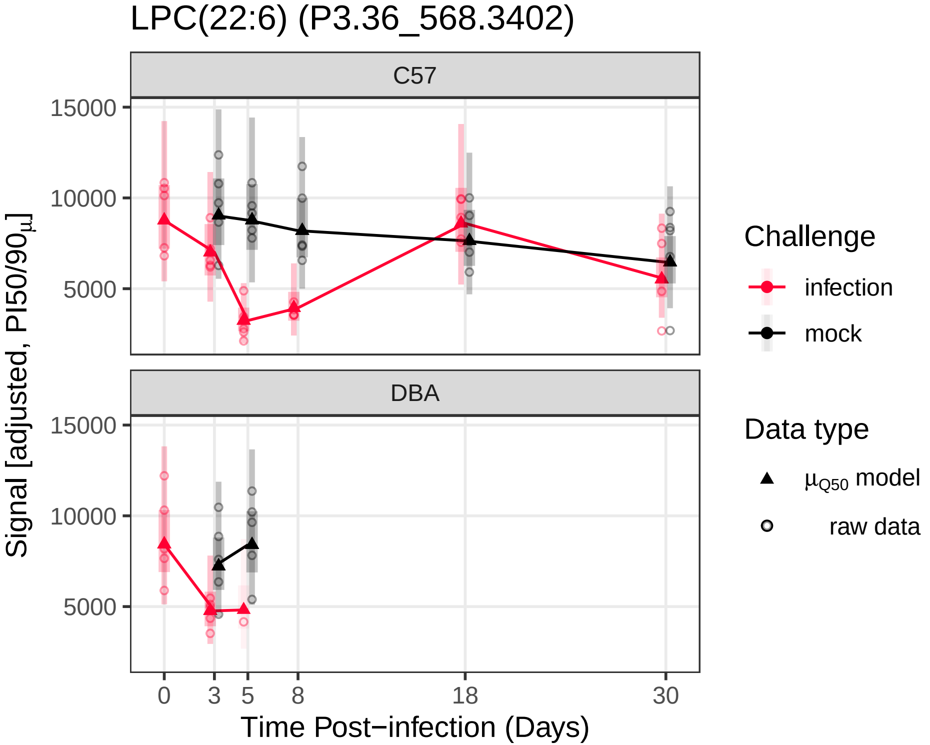
LPC(22:6) (P3.36_568.3402)
C57
DBA
15000
10000
5000
Signal [adjusted, PI50/90μ]
0
3
5
8
18
30
Time Post−infection (Days)
Challenge
infection
mock
Data type
μQ50 model
raw data

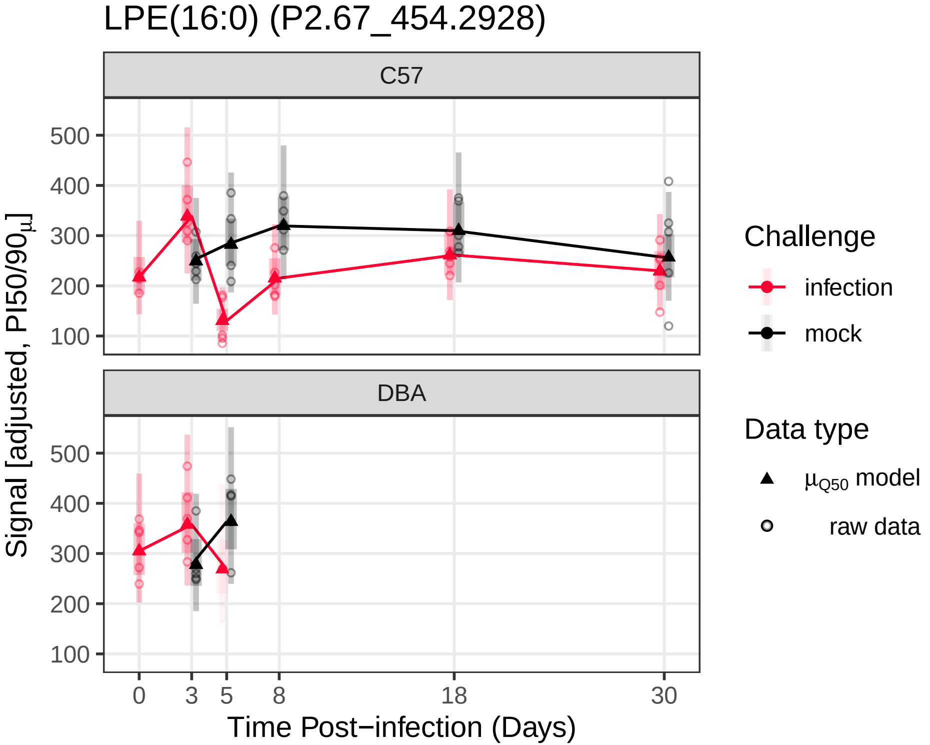

LPE(16:0) (P2.67_454.2928)
C57
DBA
Signal [adjusted, PI50/90μ]
500
400
300
200
100
0
3
5
8
18
30
Time Post−infection (Days)
Challenge
infection
mock
Data type
μQ50 model
raw data

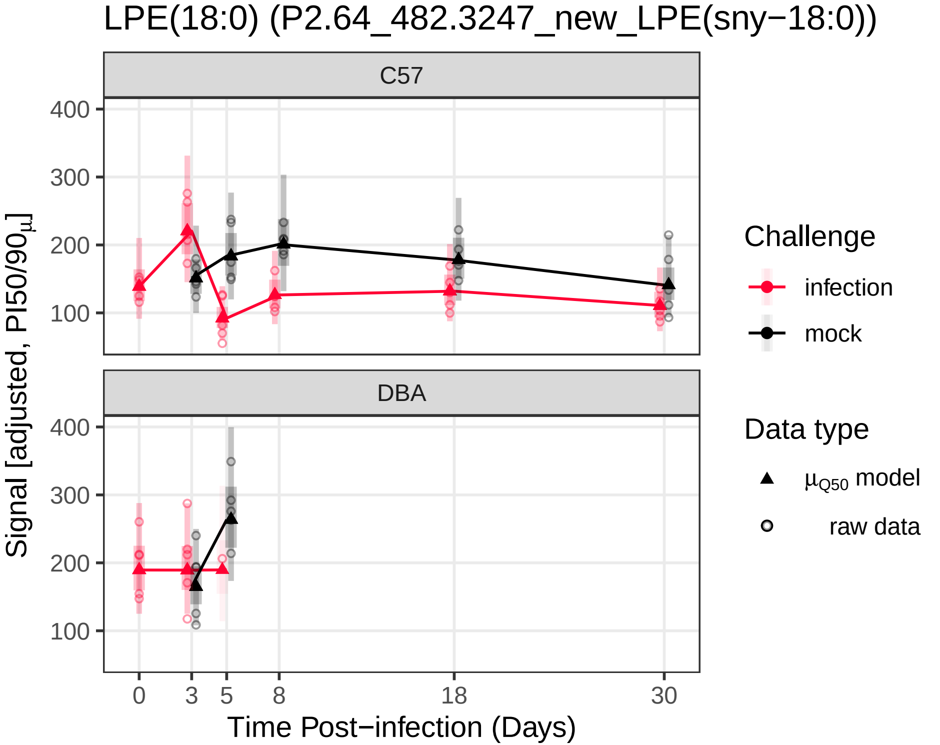

LPE(18:0) (P2.64_482.3247_new_LPE(sny−18:0))
C57
DBA
400
300
200
100
Signal [adjusted, PI50/90μ]
0
3
5
8
18
30
Time Post−infection (Days)
Challenge
infection
mock
Data type
μQ50 model
raw data

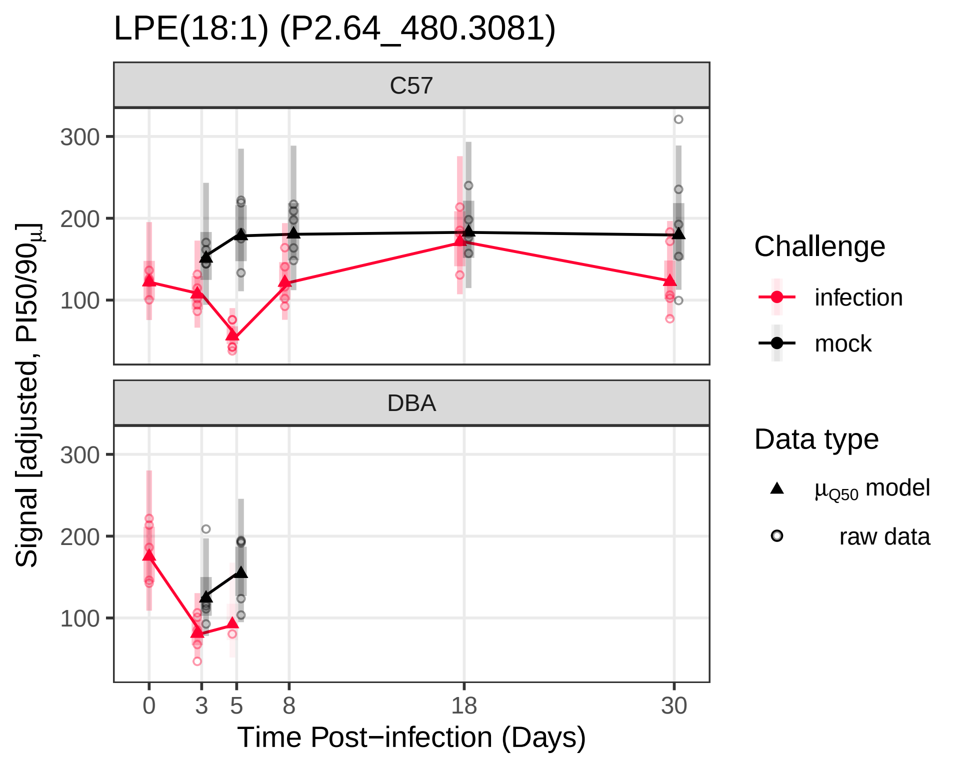

LPE(18:1) (P2.64_480.3081)
C57
DBA
300
200
100
0
3
5
8
18
30
Signal [adjusted, PI50/90$_{\mu}$]
Time Post−infection (Days)
Challenge
infection
mock
Data type
μ_{Q50} model
raw data

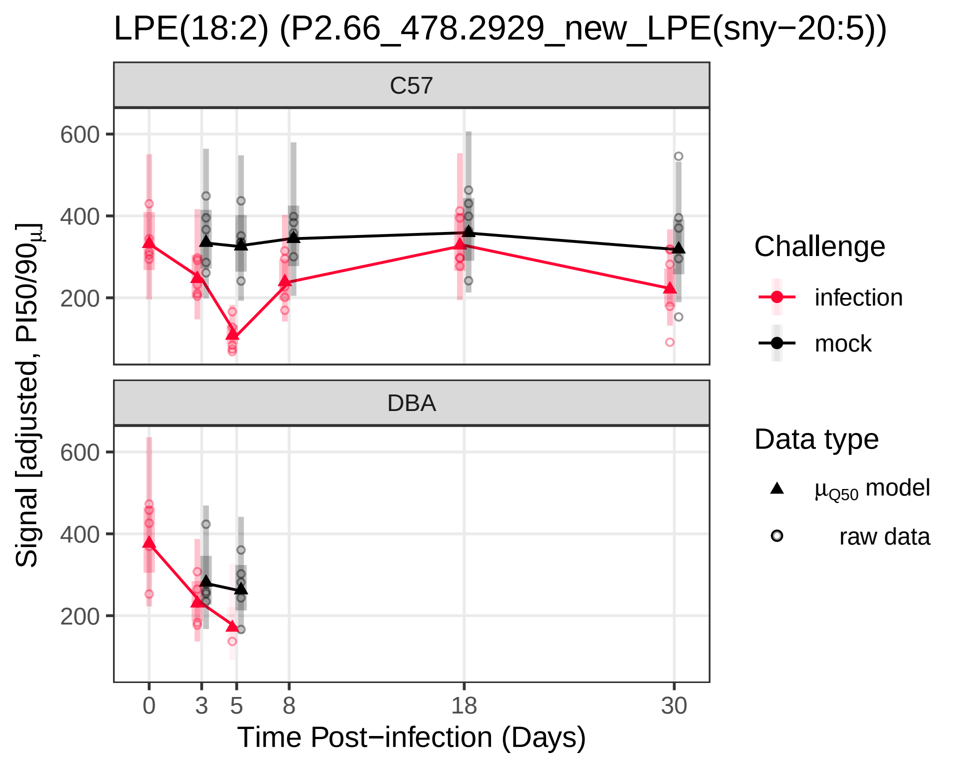

LPE(18:2) (P2.66_478.2929_new_LPE(sny−20:5))
C57
DBA
600
400
200
0
3
5
8
18
30
Signal [adjusted, PI50/90μ]
Time Post−infection (Days)
Challenge
infection
mock
Data type
μQ50 model
raw data

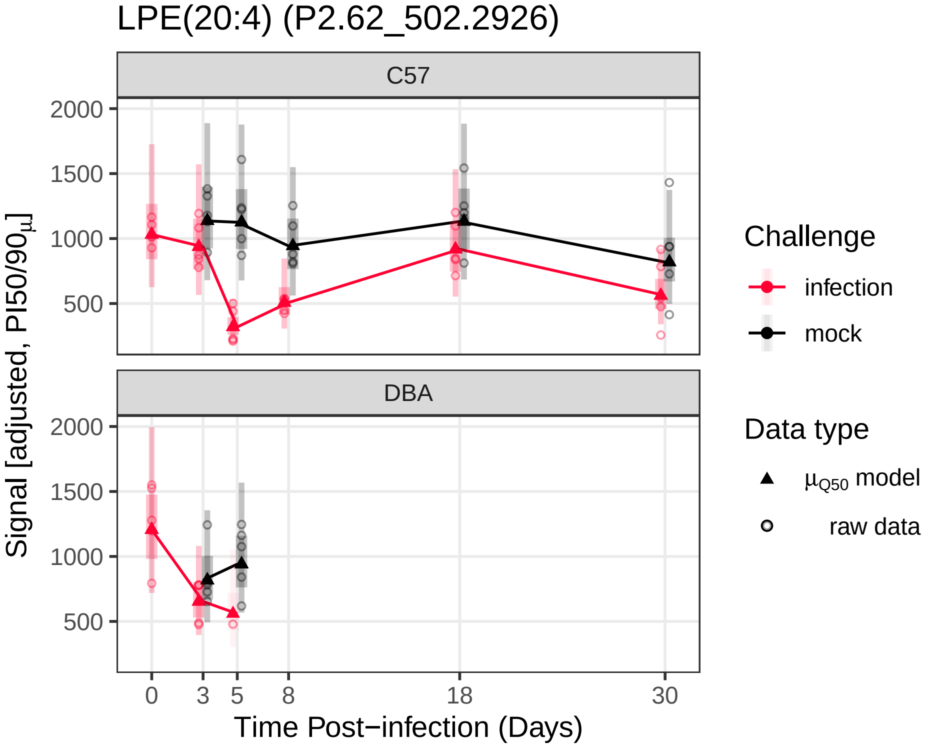
LPE(20:4) (P2.62_502.2926)
C57
DBA
2000
1500
1000
500
Signal [adjusted, PI50/90μ]
0
3
5
8
18
30
Time Post−infection (Days)
Challenge
infection
mock
Data type
μQ50 model
raw data

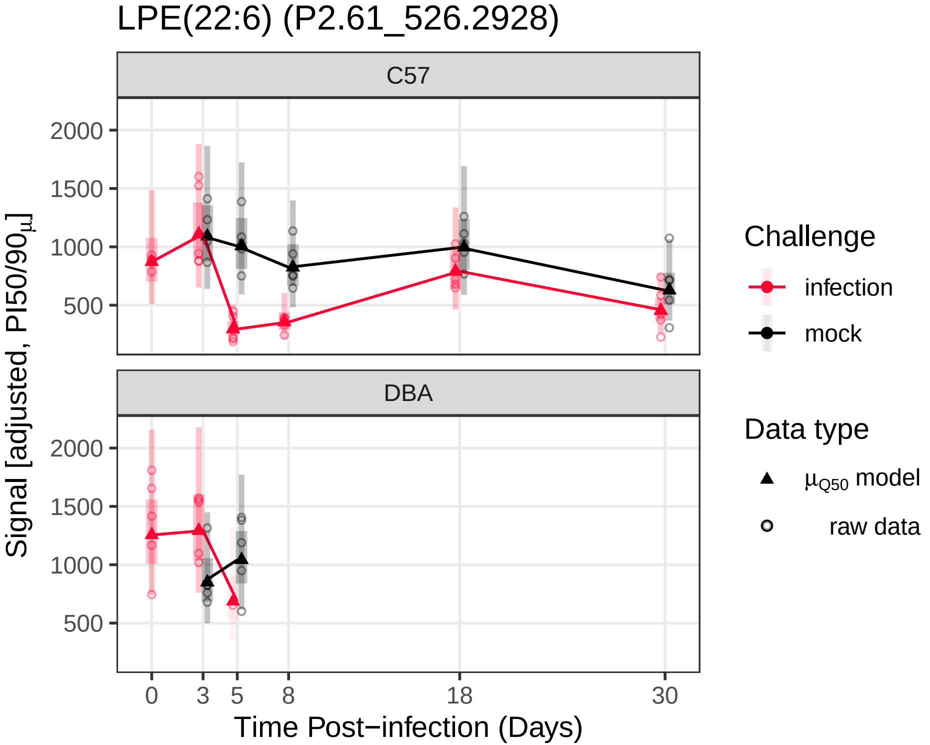

LPE(22:6) (P2.61_526.2928)
C57
DBA
2000
1500
1000
500
0
3
5
8
18
30
Signal [adjusted, PI50/90$_{\mu}$]
Time Post−infection (Days)
Challenge
infection
mock
Data type
μ_{Q50} model
raw data

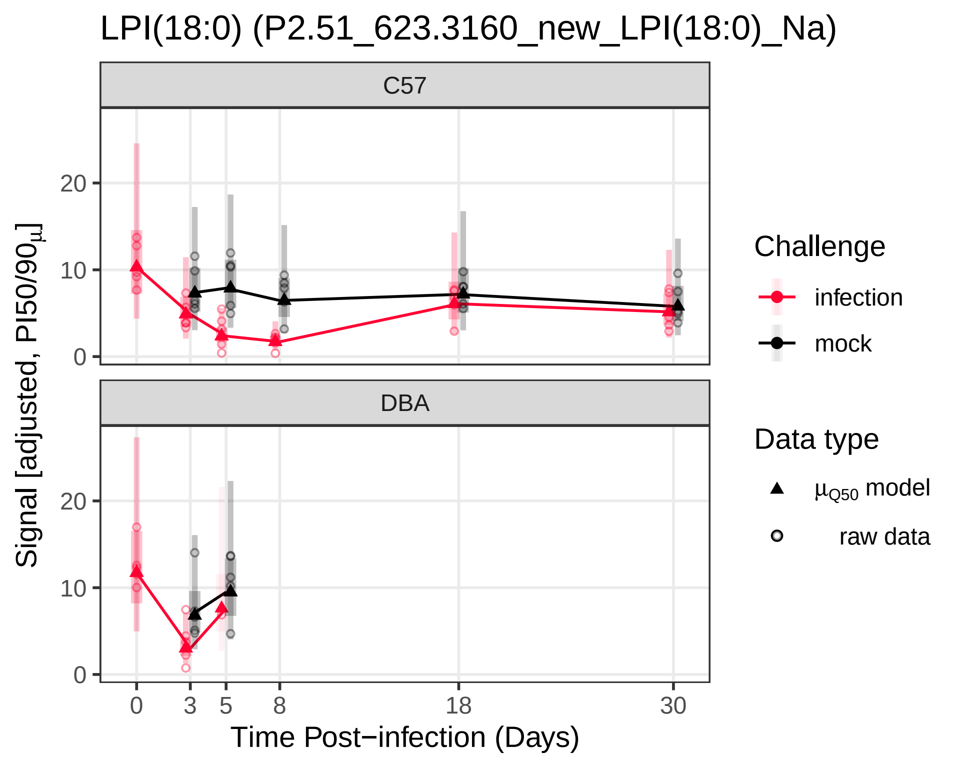
LPI(18:0) (P2.51_623.3160_new_LPI(18:0)_Na)
C57
DBA
Signal [adjusted, PI50/90μ]
0
10
20
0
10
20
0
3
5
8
18
30
Time Post−infection (Days)
Challenge
infection
mock
Data type
μQ50 model
raw data

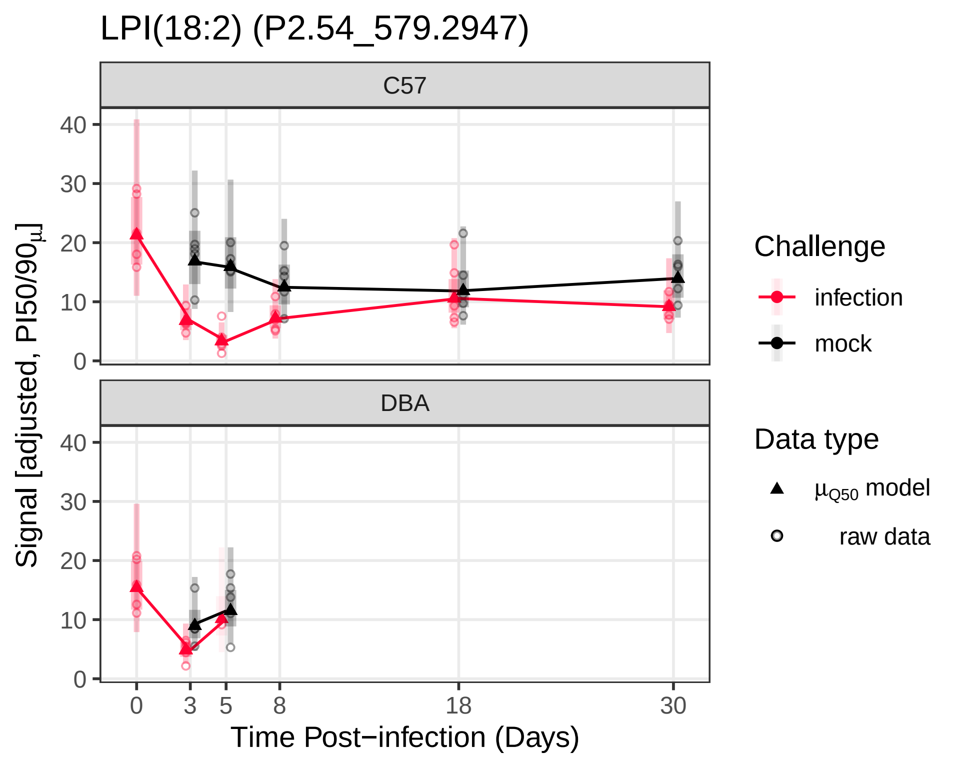

LPI(18:2) (P2.54_579.2947)
C57
DBA
Signal [adjusted, PI50/90μ]
Time Post−infection (Days)
0
10
20
30
40
0
3
5
8
18
30
Challenge
infection
mock
Data type
μQ50 model
raw data

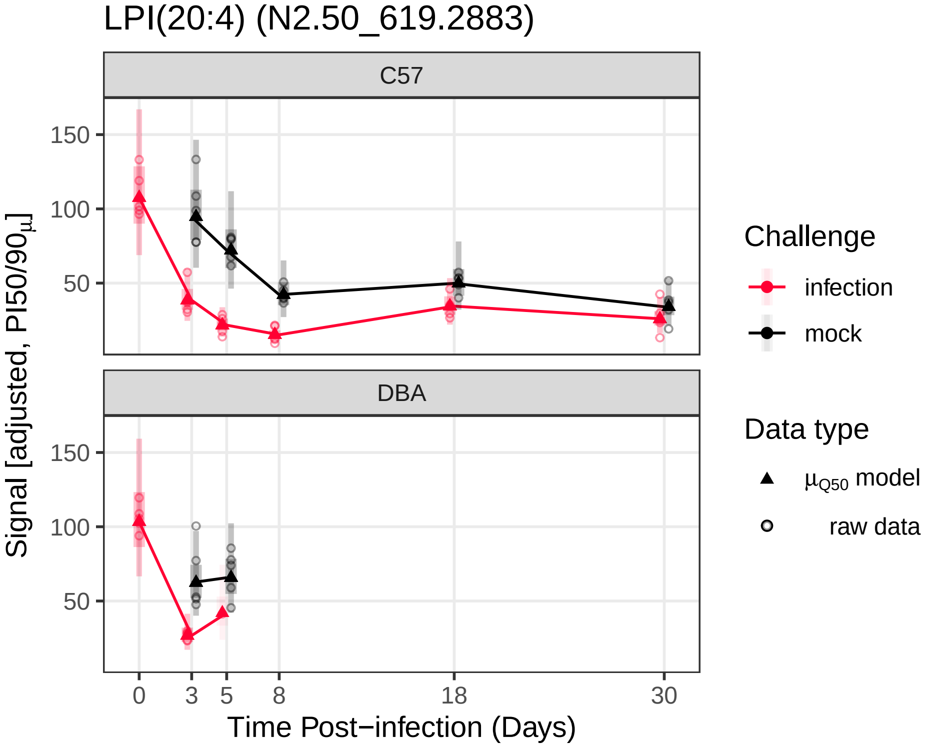

LPI(20:4) (N2.50_619.2883)
C57
DBA
150
100
50
0
3
5
8
18
30
Signal [adjusted, PI50/90μ]
Time Post−infection (Days)
Challenge
infection
mock
Data type
μQ50 model
raw data

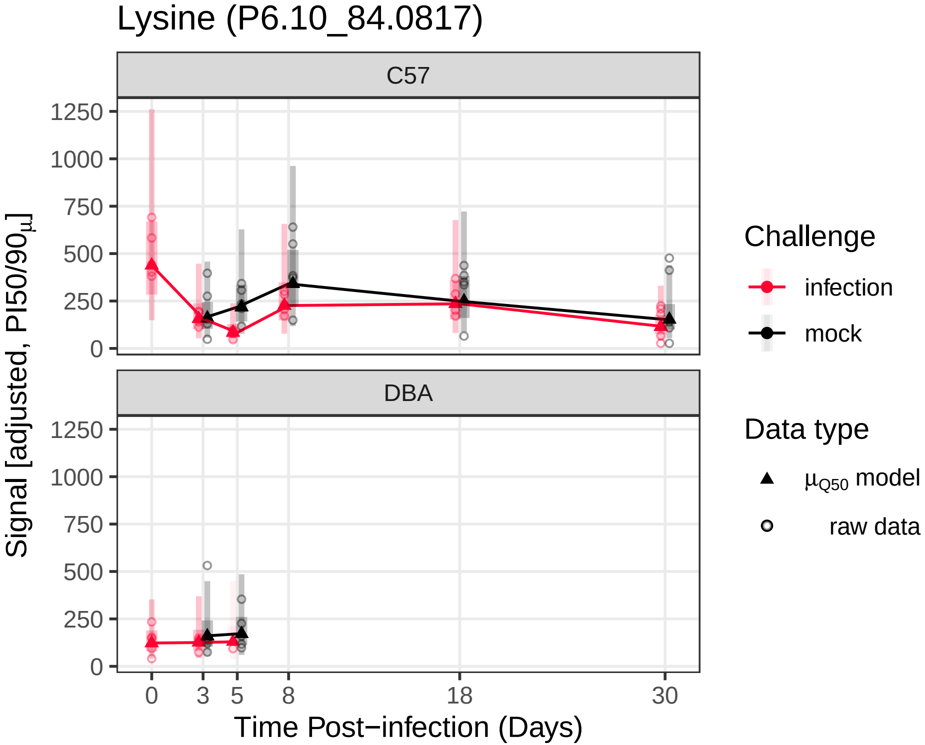
Lysine (P6.10_84.0817)
C57
DBA
1250
1000
750
500
250
0
Signal [adjusted, PI50/90μ]
0
3
5
8
18
30
Time Post−infection (Days)
Challenge
infection
mock
Data type
μQ50 model
raw data

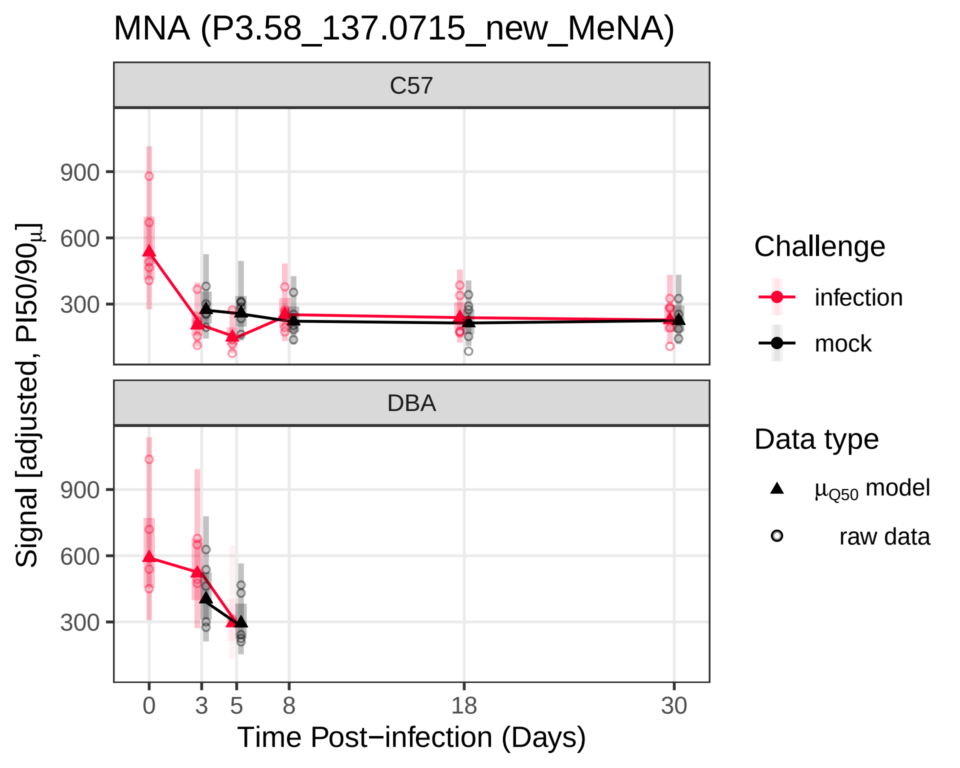

MNA (P3.58_137.0715_new_MeNA)
C57
DBA
900
600
300
Signal [adjusted, PI50/90μ]
0
3
5
8
18
30
Time Post−infection (Days)
Challenge
infection
mock
Data type
μQ50 model
raw data

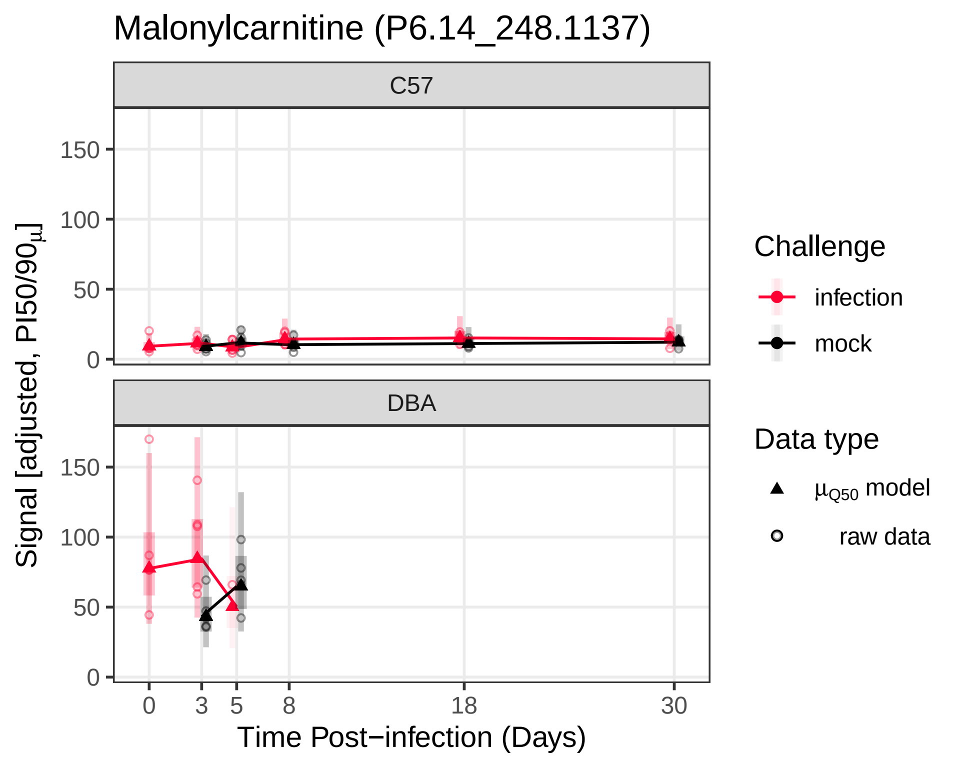

Malonylcarnitine (P6.14_248.1137)
C57
DBA
Signal [adjusted, PI50/90μ]
0
50
100
150
0
3
5
8
18
30
Time Post−infection (Days)
Challenge
infection
mock
Data type
μQ50 model
raw data

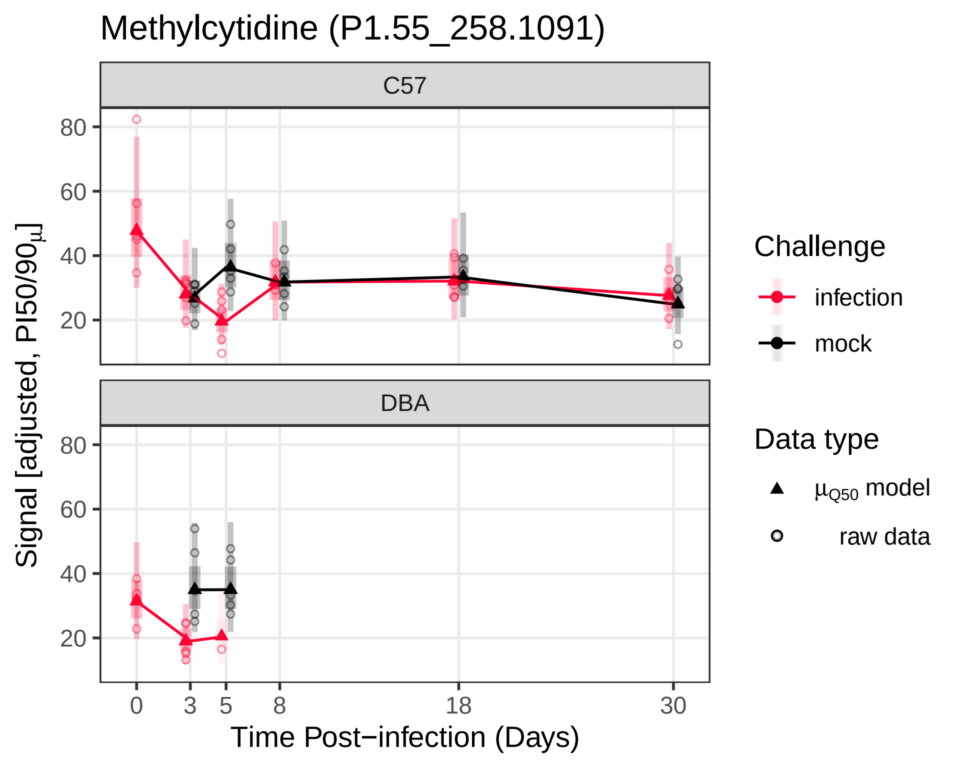
Methylcytidine (P1.55_258.1091)
C57
DBA
80
60
40
20
Signal [adjusted, PI50/90μ]
0
3
5
8
18
30
Time Post−infection (Days)
Challenge
infection
mock
Data type
μQ50 model
raw data

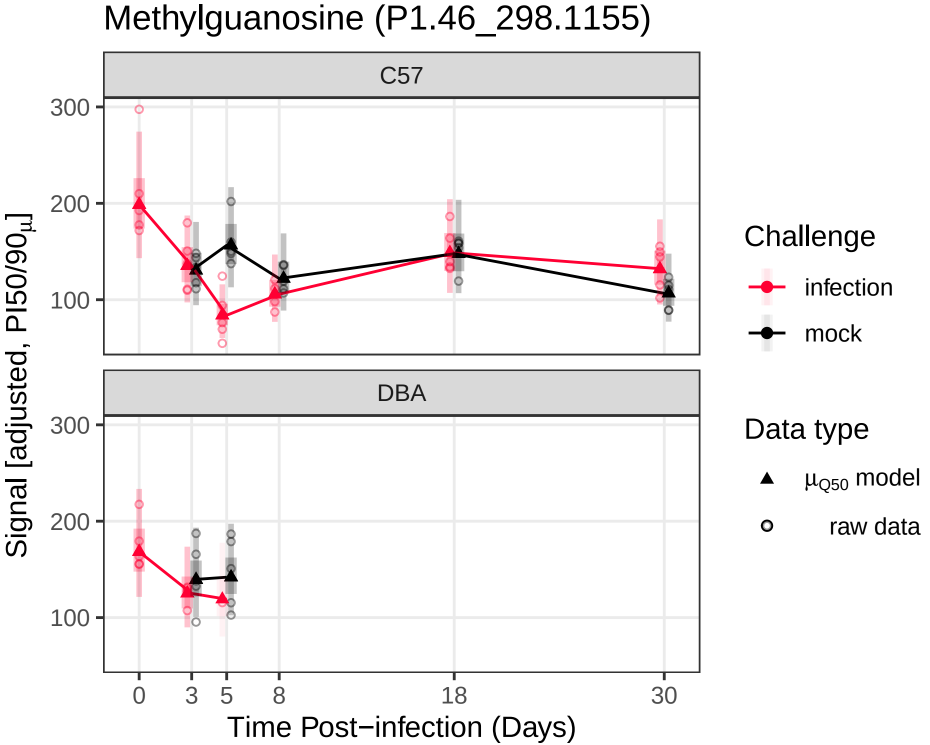

Methylguanosine (P1.46_298.1155)
C57
DBA
300
200
100
0
3
5
8
18
30
Signal [adjusted, PI50/90μ]
Time Post−infection (Days)
Challenge
infection
mock
Data type
μQ50 model
raw data

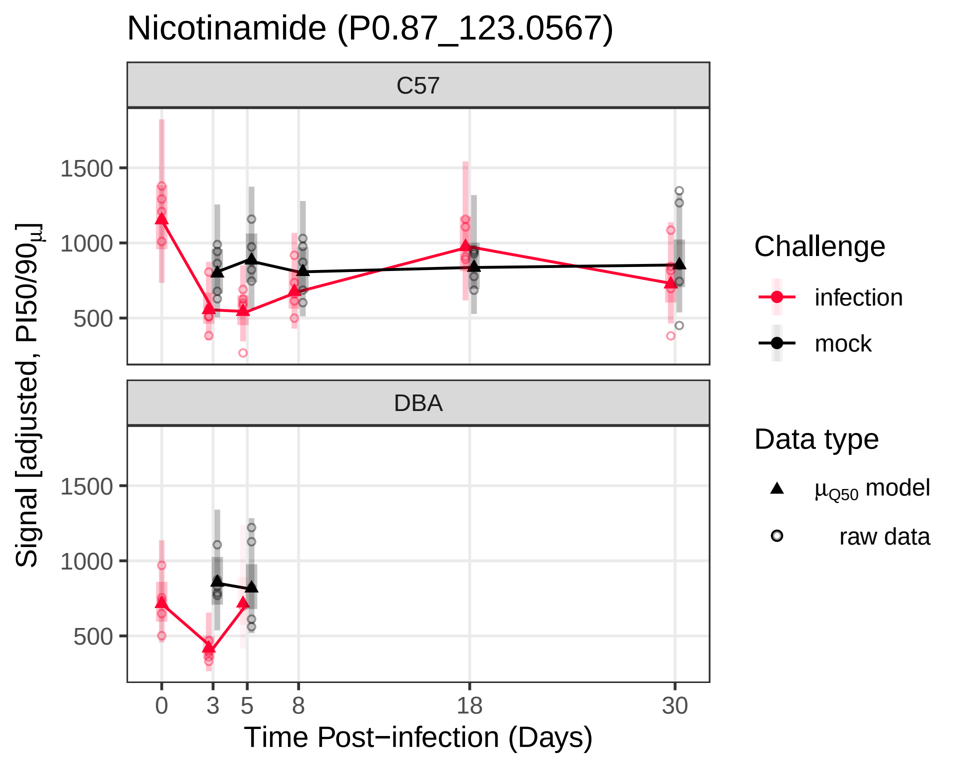

Nicotinamide (P0.87_123.0567)
C57
DBA
1500
1000
500
Signal [adjusted, PI50/90$_{\mu}$]
0
3
5
8
18
30
Time Post−infection (Days)
Challenge
infection
mock
Data type
μ_{Q50} model
raw data

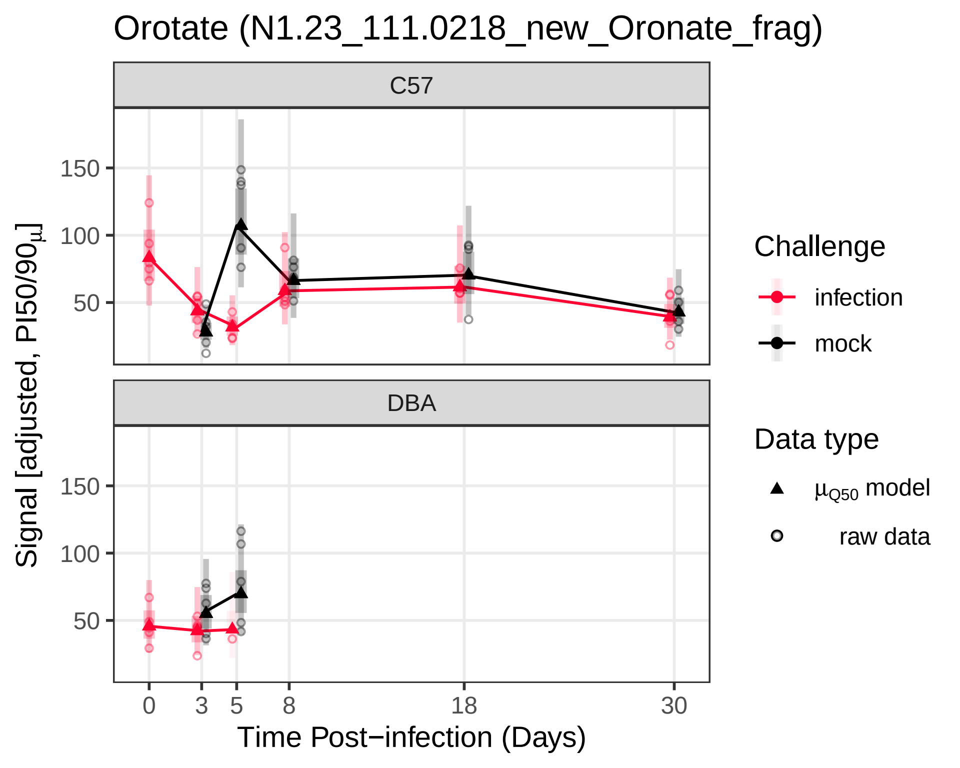

Orotate (N1.23_111.0218_new_Oronate_frag)
C57
DBA
150
100
50
0
3
5
8
18
30
Time Post−infection (Days)
Signal [adjusted, PI50/90μ]
Challenge
infection
mock
Data type
μQ50 model
raw data

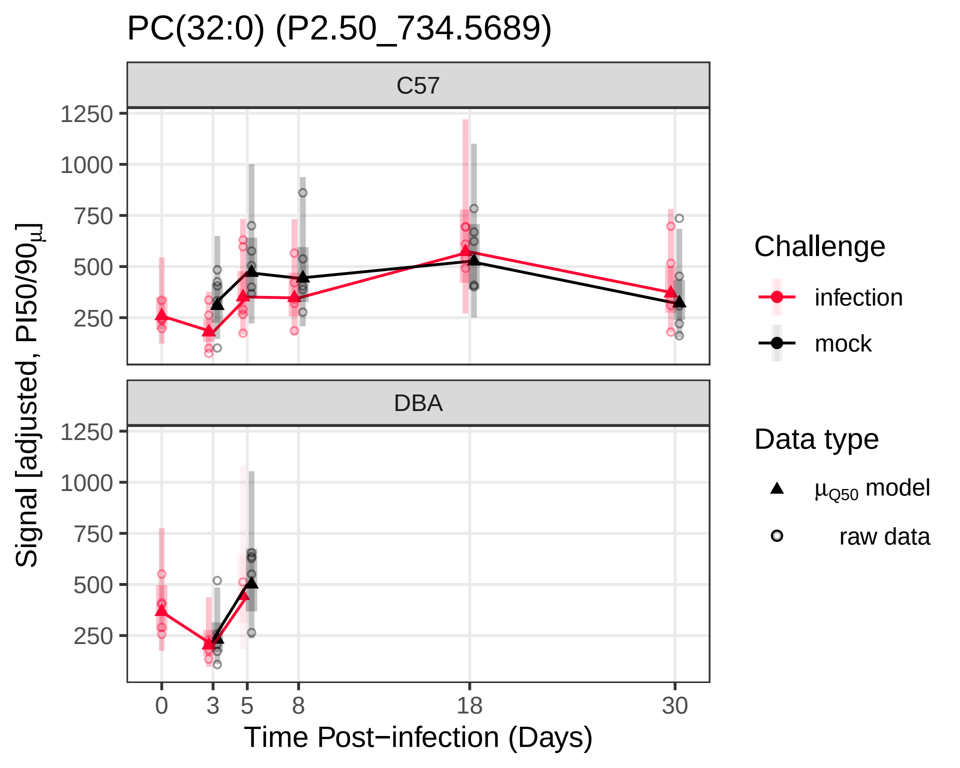
PC(32:0) (P2.50_734.5689)
C57
DBA
1250
1000
750
500
250
0
3
5
8
18
30
Signal [adjusted, PI50/90μ]
Time Post−infection (Days)
Challenge
infection
mock
Data type
μQ50 model
raw data

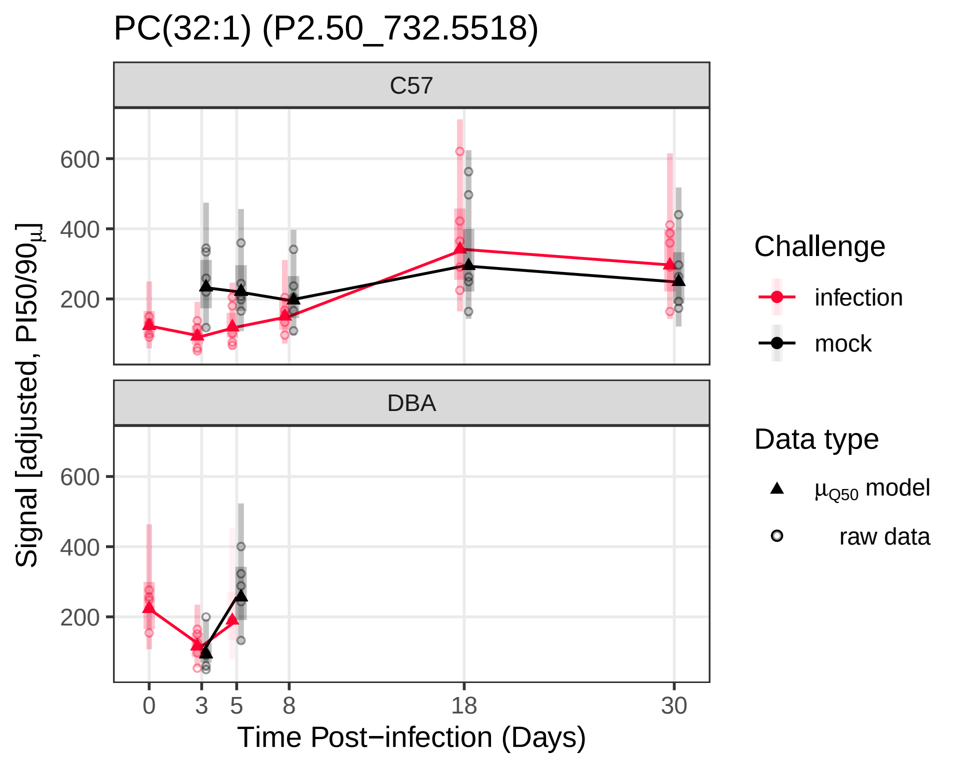
PC(32:1) (P2.50_732.5518)
C57
DBA
Signal [adjusted, PI50/90$_{\mu}$]
600
400
200
0
3
5
8
18
30
Time Post−infection (Days)
Challenge
infection
mock
Data type
μ_{Q50} model
raw data

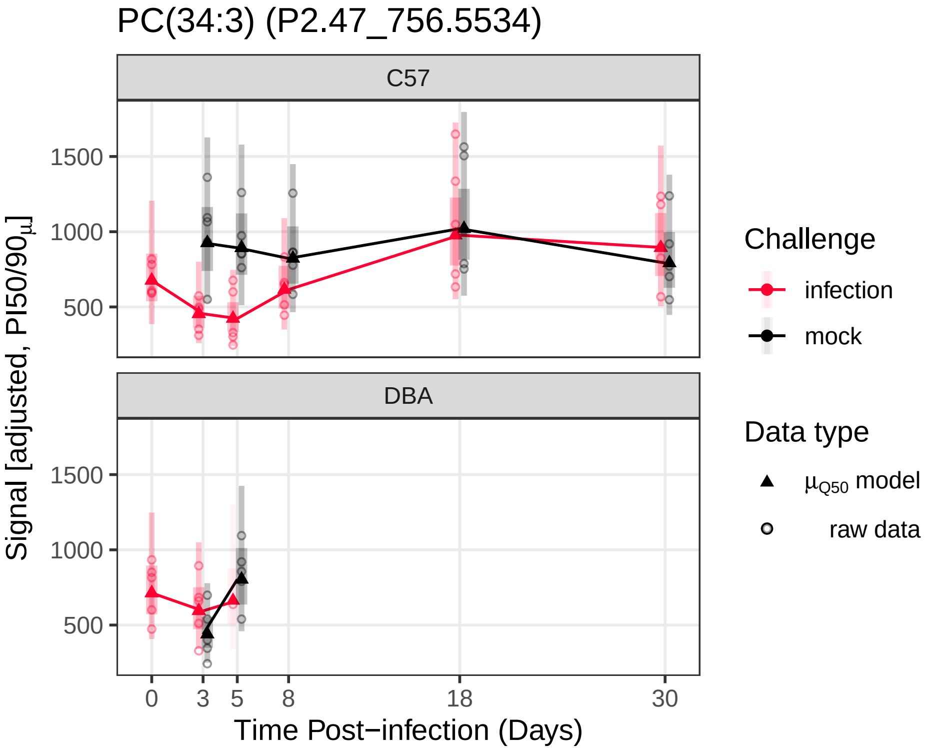
PC(34:3) (P2.47_756.5534)
C57
DBA
Signal [adjusted, PI50/90μ]
1500
1000
500
0
3
5
8
18
30
Time Post−infection (Days)
Challenge
infection
mock
Data type
μQ50 model
raw data

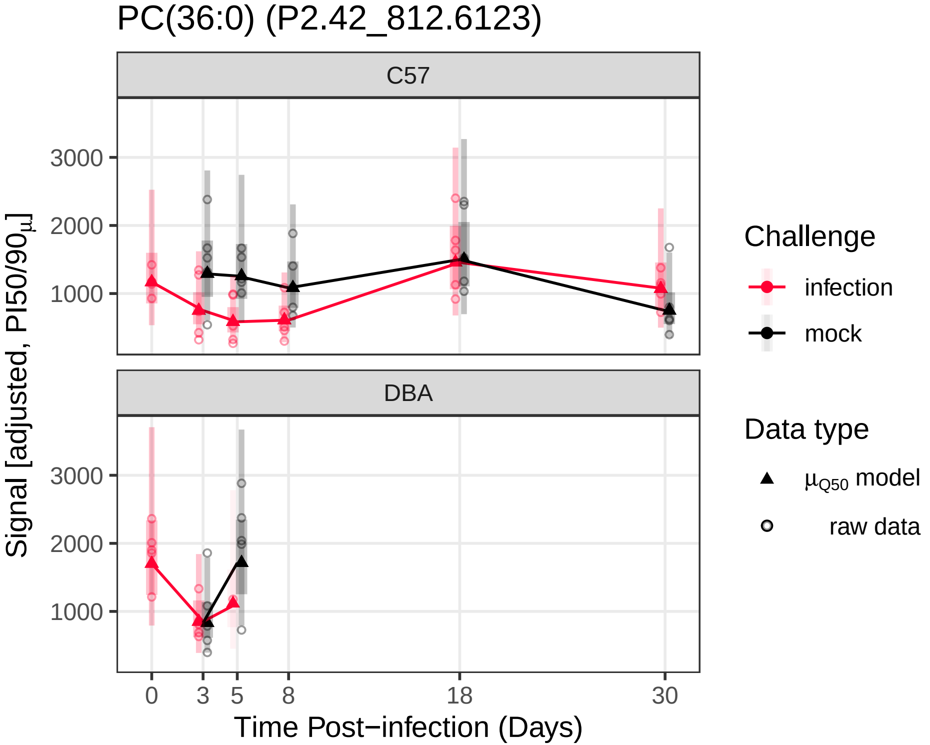
PC(36:0) (P2.42_812.6123)
C57
DBA
3000
2000
1000
Signal [adjusted, PI50/90$_{\mu}$]
0
3
5
8
18
30
Time Post−infection (Days)
Challenge
infection
mock
Data type
μ_{Q50} model
raw data

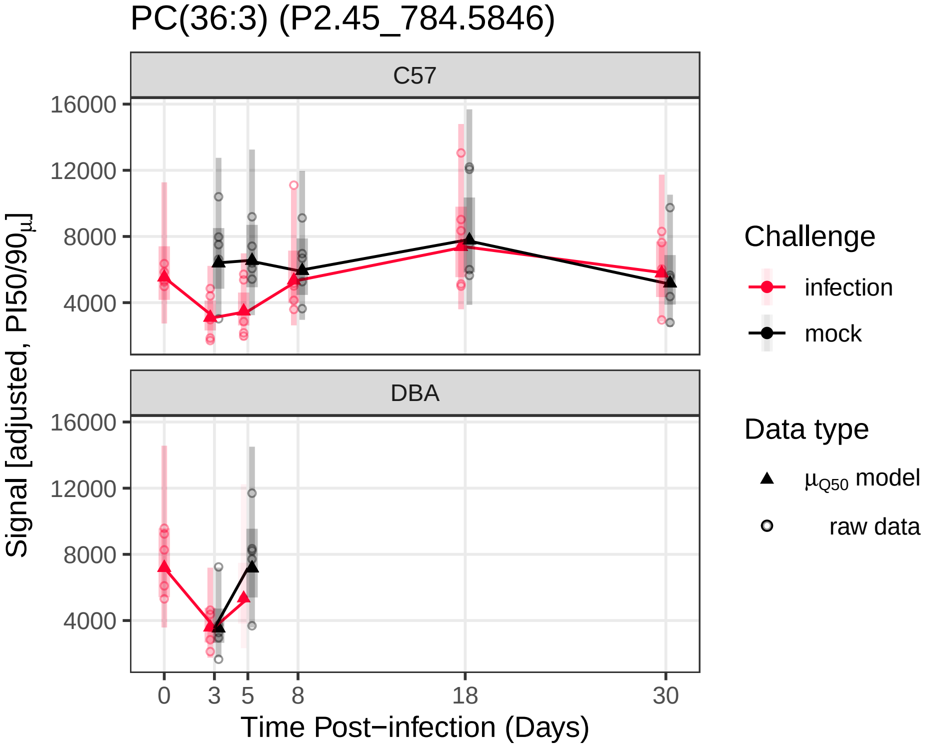
PC(36:3) (P2.45_784.5846)
C57
DBA
16000
12000
8000
4000
0
3
5
8
18
30
Time Post−infection (Days)
Signal [adjusted, PI50/90μ]
Challenge
infection
mock
Data type
μQ50 model
raw data

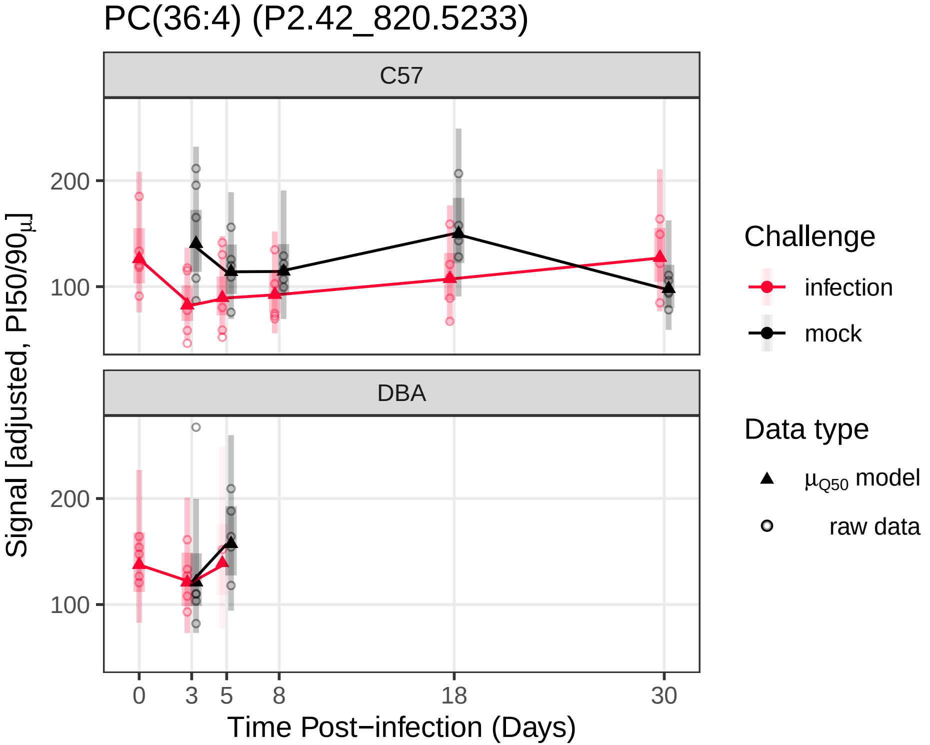
PC(36:4) (P2.42_820.5233)
C57
DBA
200
100
200
100
Signal [adjusted, PI50/90μ]
0
3
5
8
18
30
Time Post−infection (Days)
Challenge
infection
mock
Data type
μQ50 model
raw data

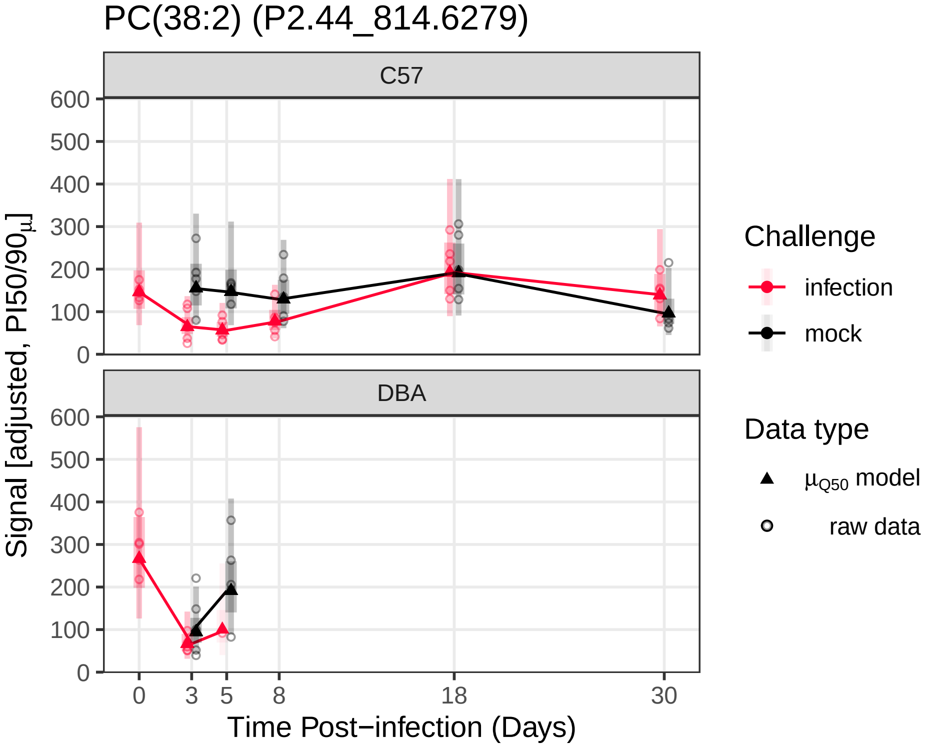

PC(38:2) (P2.44_814.6279)
C57
DBA
600
500
400
300
200
100
0
0
3
5
8
18
30
Time Post−infection (Days)
Signal [adjusted, PI50/90μ]
Challenge
infection
mock
Data type
μQ50 model
raw data

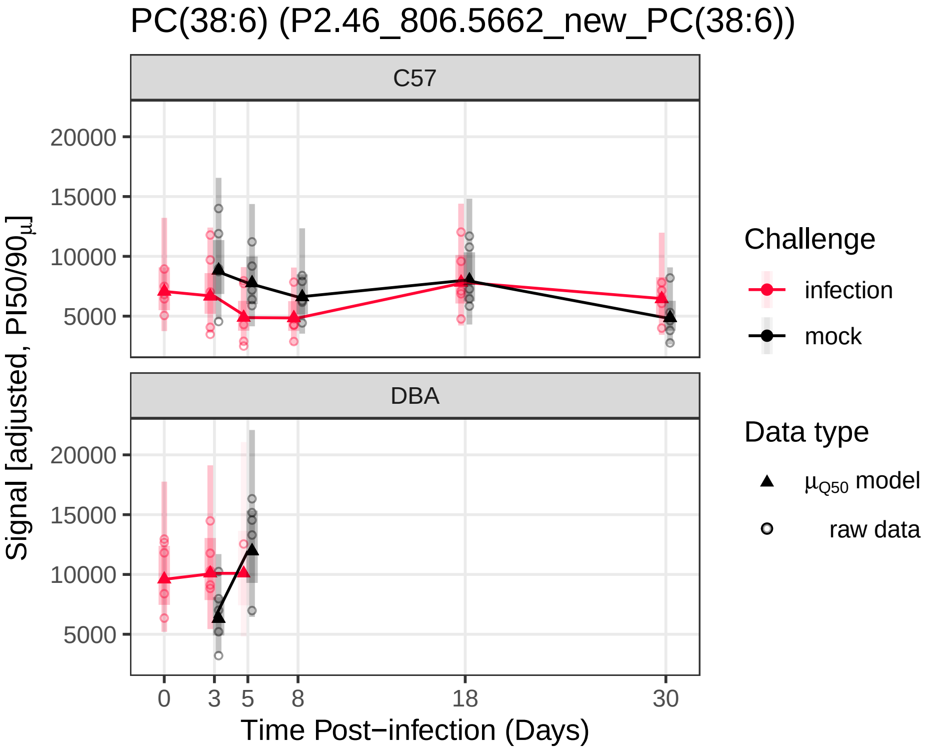

PC(38:6) (P2.46_806.5662_new_PC(38:6))
C57
DBA
20000
15000
10000
5000
0
3
5
8
18
30
Signal [adjusted, PI50/90μ]
Time Post−infection (Days)
Challenge
infection
mock
Data type
μQ50 model
raw data

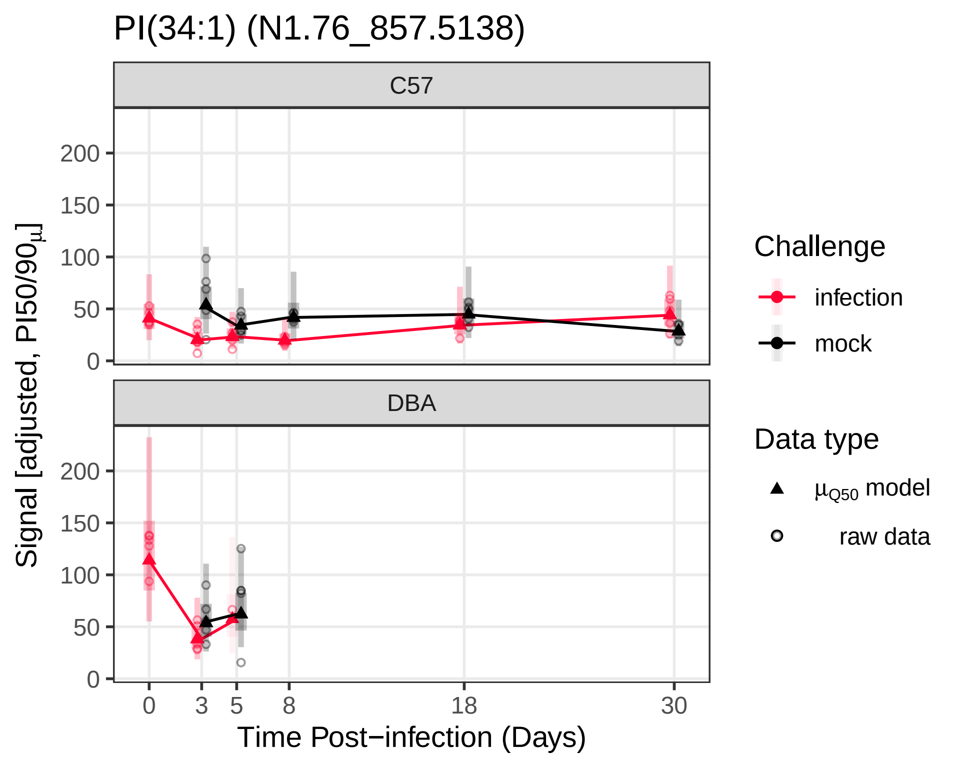
PI(34:1) (N1.76_857.5138)
C57
DBA
Signal [adjusted, PI50/90μ]
200
150
100
50
0
0
3
5
8
18
30
Time Post−infection (Days)
Challenge
infection
mock
Data type
μQ50 model
raw data

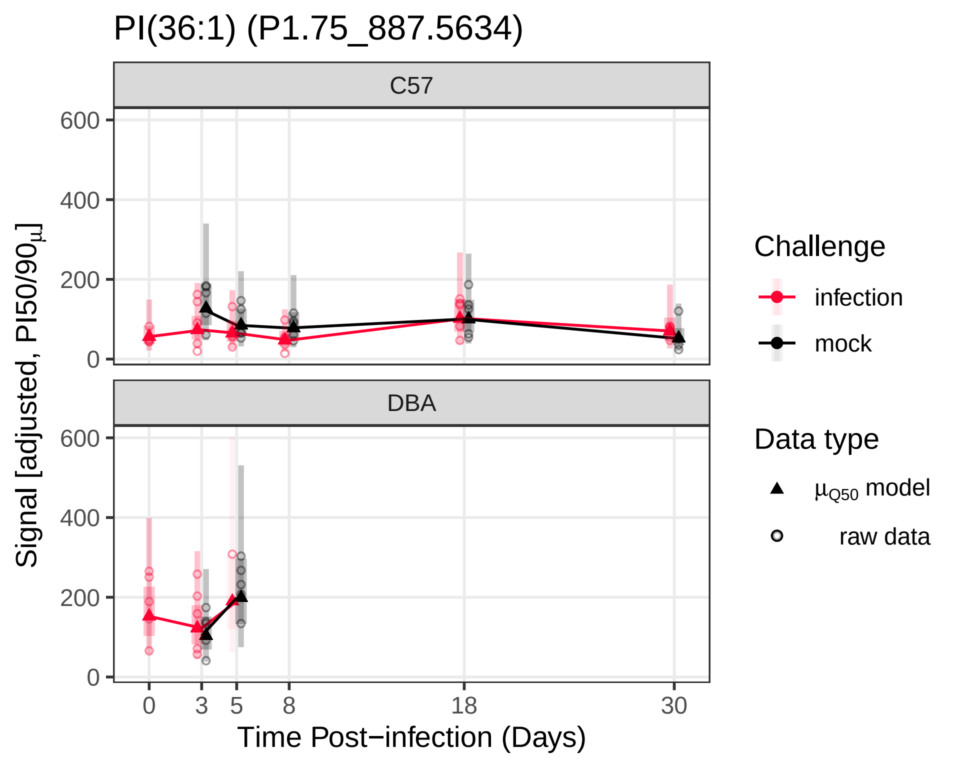
PI(36:1) (P1.75_887.5634)
C57
DBA
600
400
200
0
Signal [adjusted, PI50/90$_{\mu}$]
0
3
5
8
18
30
Time Post−infection (Days)
Challenge
infection
mock
Data type
μ_{Q50} model
raw data

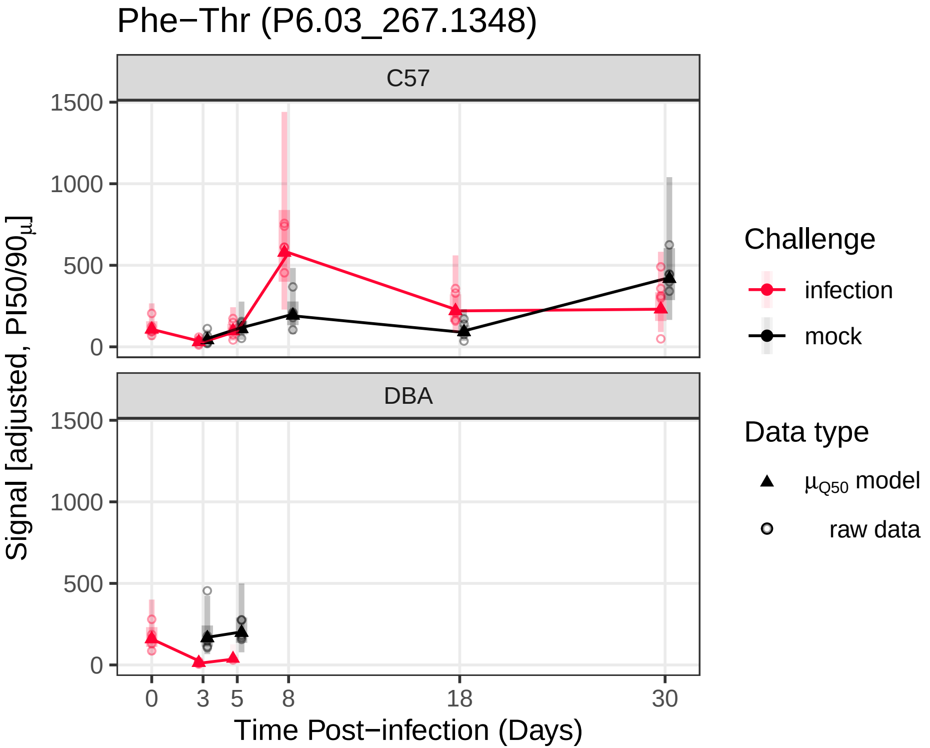

Phe−Thr (P6.03_267.1348)
C57
DBA
1500
1000
500
0
0
3
5
8
18
30
Time Post−infection (Days)
Signal [adjusted, PI50/90μ]
Challenge
infection
mock
Data type
μQ50 model
raw data

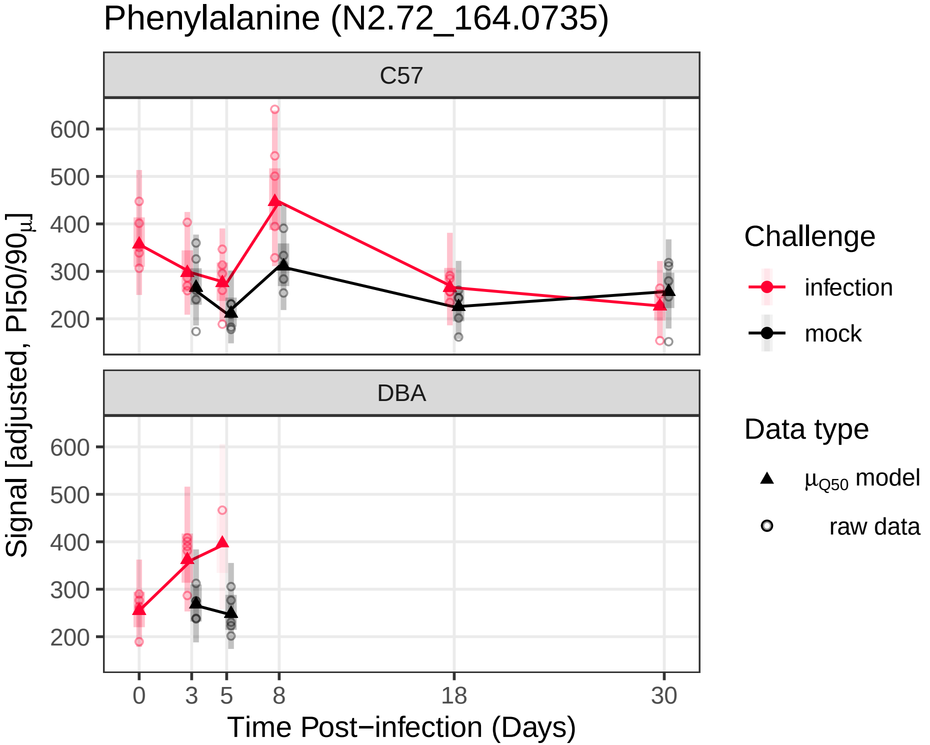

Phenylalanine (N2.72_164.0735)
C57
DBA
600
500
400
300
200
0
3
5
8
18
30
Signal [adjusted, PI50/90μ]
Time Post−infection (Days)
Challenge
infection
mock
Data type
μQ50 model
raw data

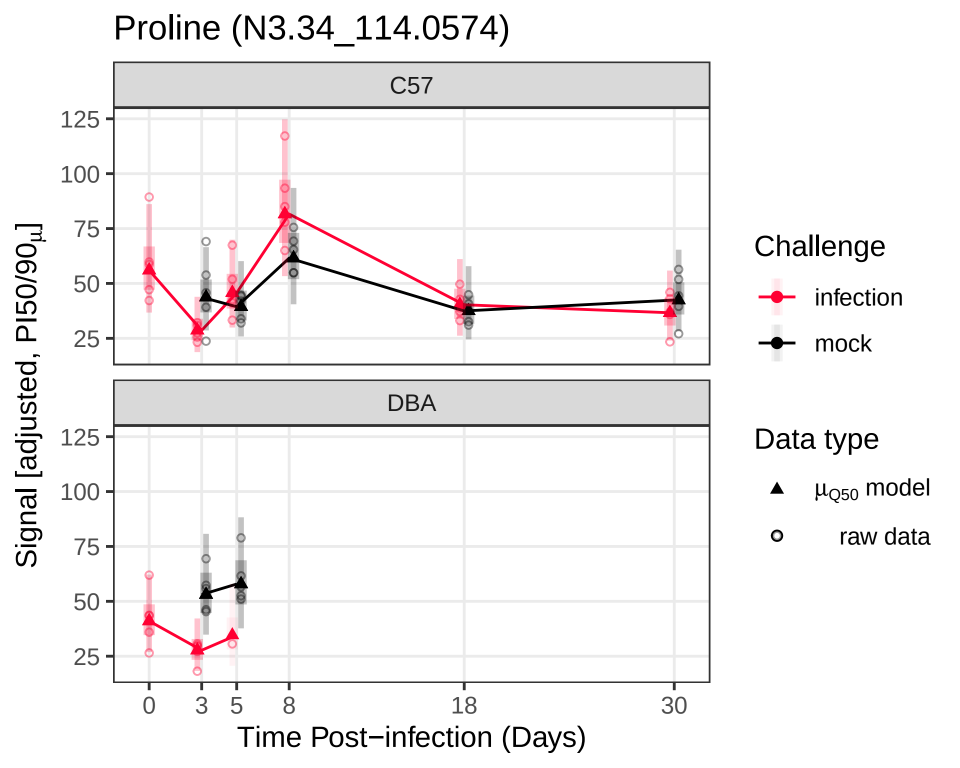
Proline (N3.34_114.0574)
C57
DBA
125
100
75
50
25
0
3
5
8
18
30
Signal [adjusted, PI50/90μ]
Time Post−infection (Days)
Challenge
infection
mock
Data type
μQ50 model
raw data

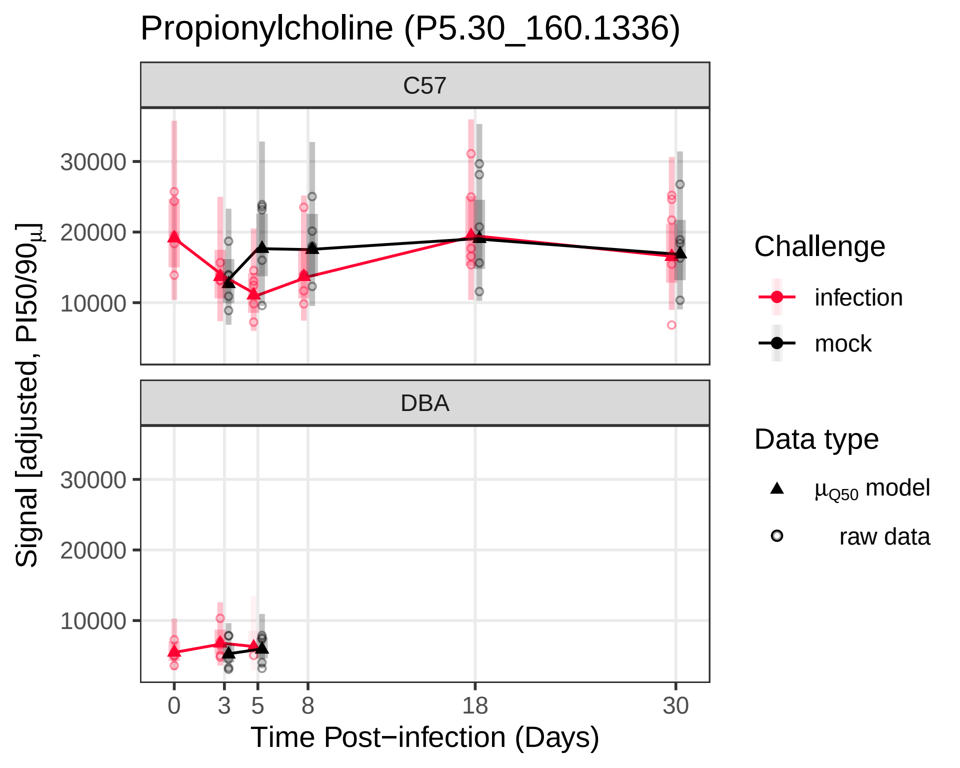

Propionylcholine (P5.30_160.1336)
C57
DBA
30000
20000
10000
Signal [adjusted, PI50/90$_{\mu}$]
0
3
5
8
18
30
Time Post−infection (Days)
Challenge
infection
mock
Data type
μ_{Q50} model
raw data

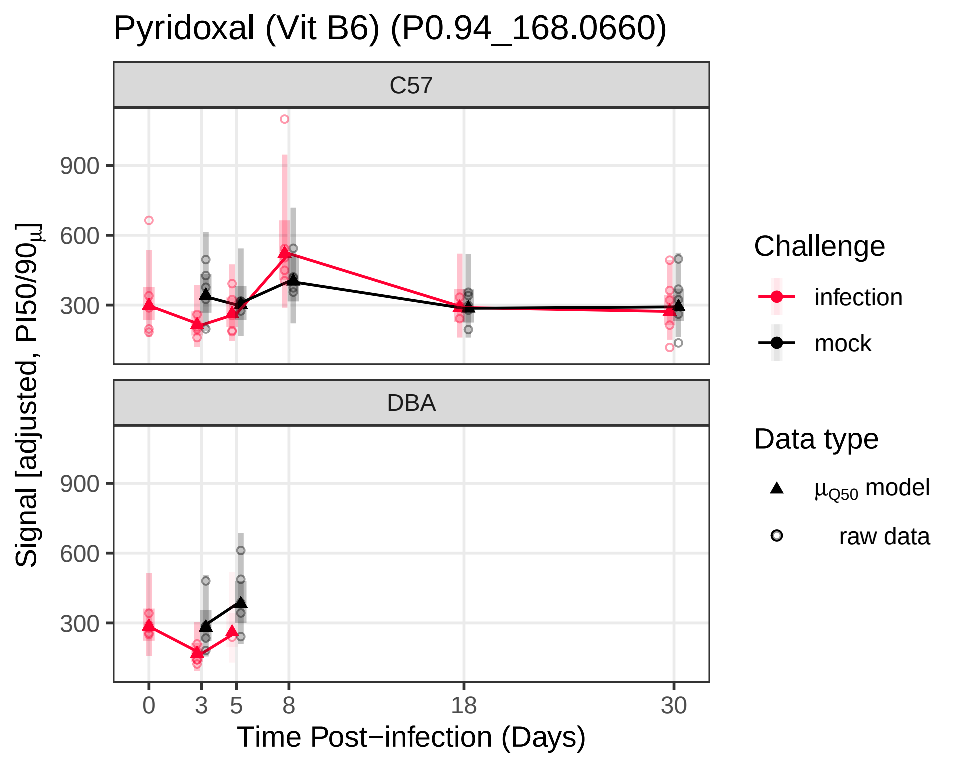

Pyridoxal (Vit B6) (P0.94_168.0660)
C57
DBA
900
600
300
Signal [adjusted, PI50/90$_{\mu}$]
0
3
5
8
18
30
Time Post−infection (Days)
Challenge
infection
mock
Data type
μ_{Q50} model
raw data

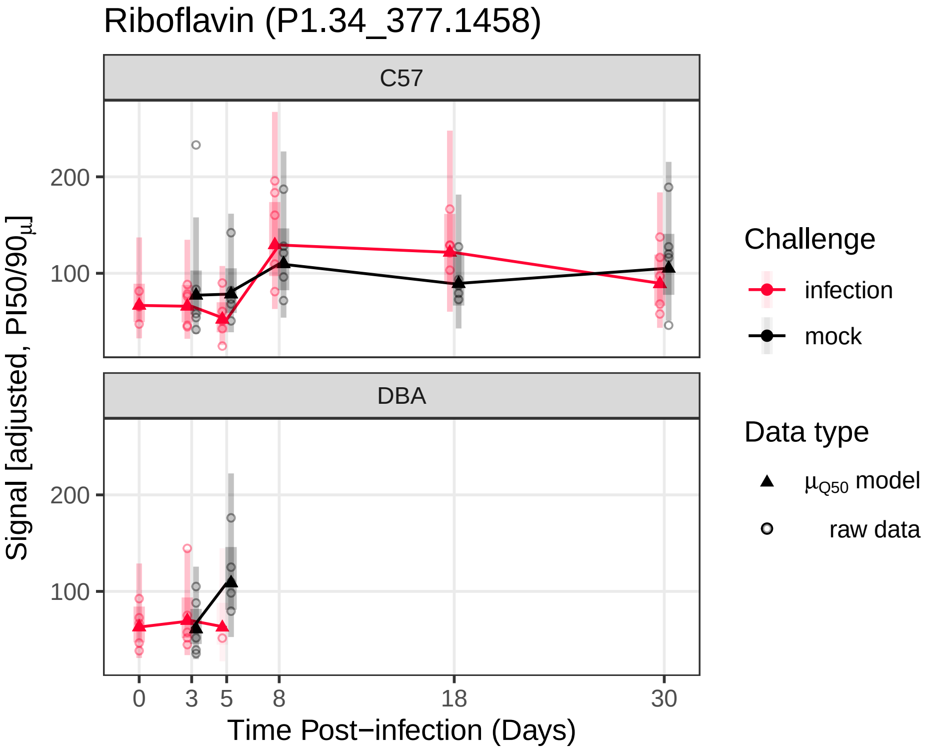
Riboflavin (P1.34_377.1458)
C57
DBA
200
100
Signal [adjusted, PI50/90$_{\mu}$]
0
3
5
8
18
30
Time Post−infection (Days)
Challenge
infection
mock
Data type
μ_{Q50} model
raw data

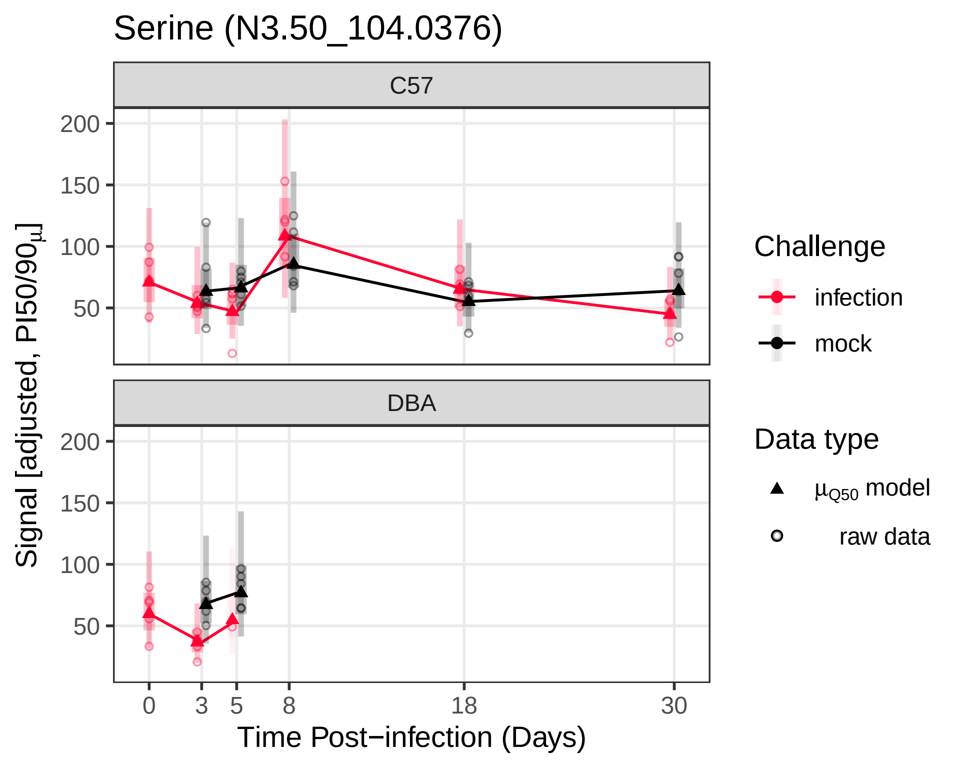
Serine (N3.50_104.0376)
C57
DBA
Signal [adjusted, PI50/90μ]
200
150
100
50
0
3
5
8
18
30
Time Post−infection (Days)
Challenge
infection
mock
Data type
μQ50 model
raw data

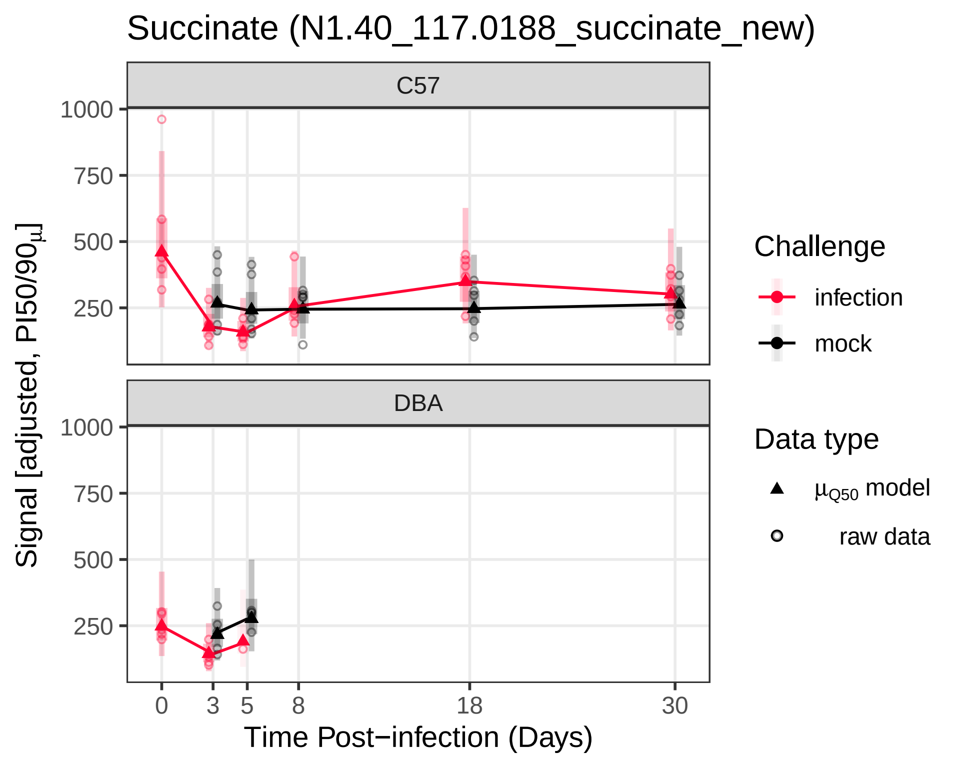
Succinate (N1.40_117.0188_succinate_new)
C57
DBA
1000
750
500
250
0
3
5
8
18
30
Signal [adjusted, PI50/90μ]
Time Post−infection (Days)
Challenge
infection
mock
Data type
μQ50 model
raw data

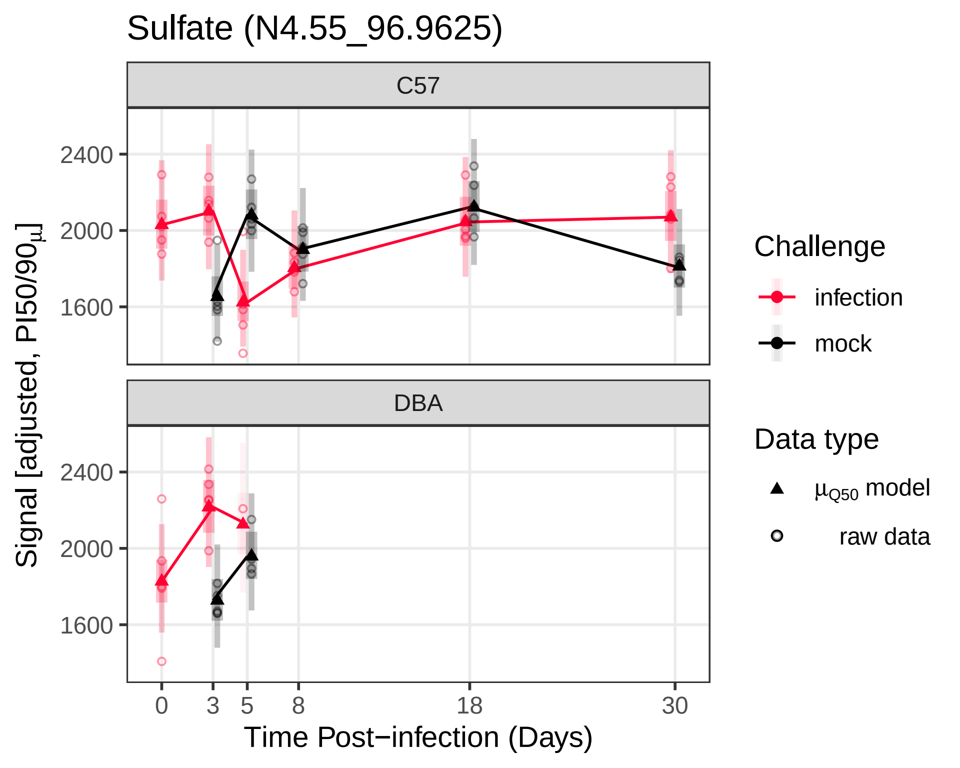
Sulfate (N4.55_96.9625)
C57
DBA
2400
2000
1600
Signal [adjusted, PI50/90μ]
0
3
5
8
18
30
Time Post−infection (Days)
Challenge
infection
mock
Data type
μQ50 model
raw data

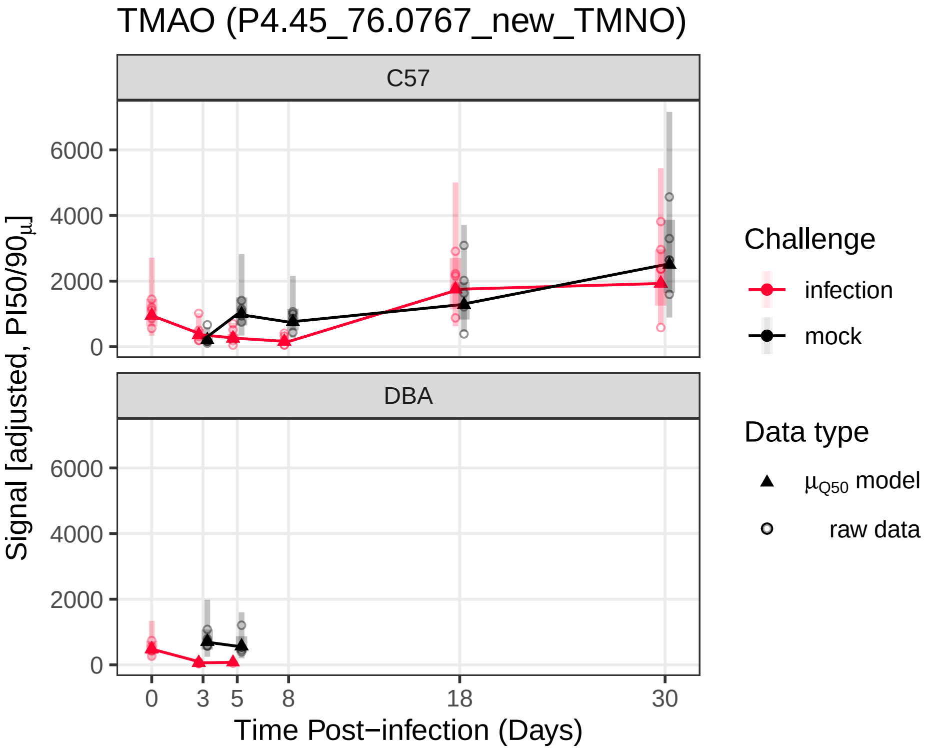
TMAO (P4.45_76.0767_new_TMNO)
C57
DBA
6000
4000
2000
0
Signal [adjusted, PI50/90μ]
0
3
5
8
18
30
Time Post−infection (Days)
Challenge
infection
mock
Data type
μQ50 model
raw data

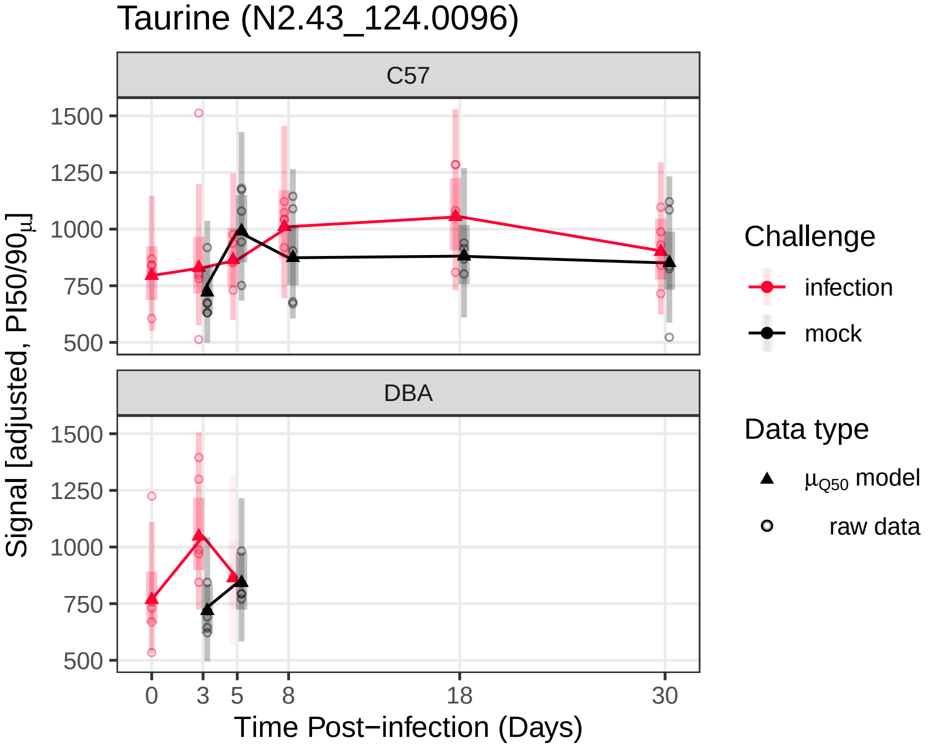

Taurine (N2.43_124.0096)
C57
DBA
1500
1250
1000
750
500
0
3
5
8
18
30
Signal [adjusted, PI50/90μ]
Time Post−infection (Days)
Challenge
infection
mock
Data type
μQ50 model
raw data

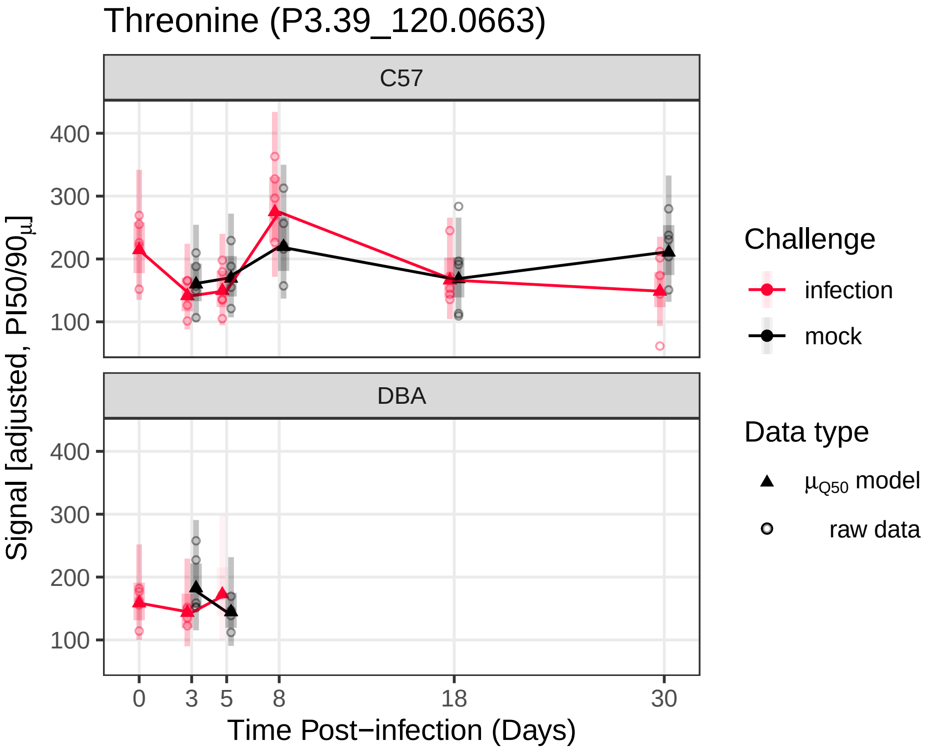

Threonine (P3.39_120.0663)
C57
DBA
400
300
200
100
0
3
5
8
18
30
Signal [adjusted, PI50/90$_{\mu}$]
Time Post−infection (Days)
Challenge
infection
mock
Data type
μ_{Q50} model
raw data

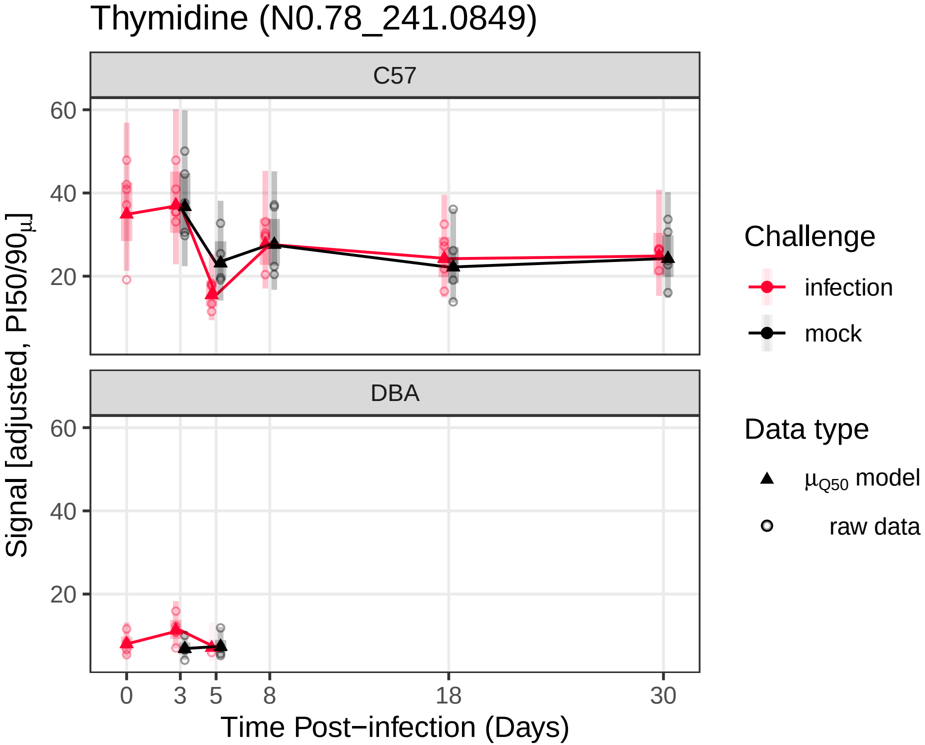
Thymidine (N0.78_241.0849)
C57
DBA
Signal [adjusted, PI50/90$_\mu$]
60
40
20
0
3
5
8
18
30
Time Post−infection (Days)
Challenge
infection
mock
Data type
μ_{Q50} model
raw data

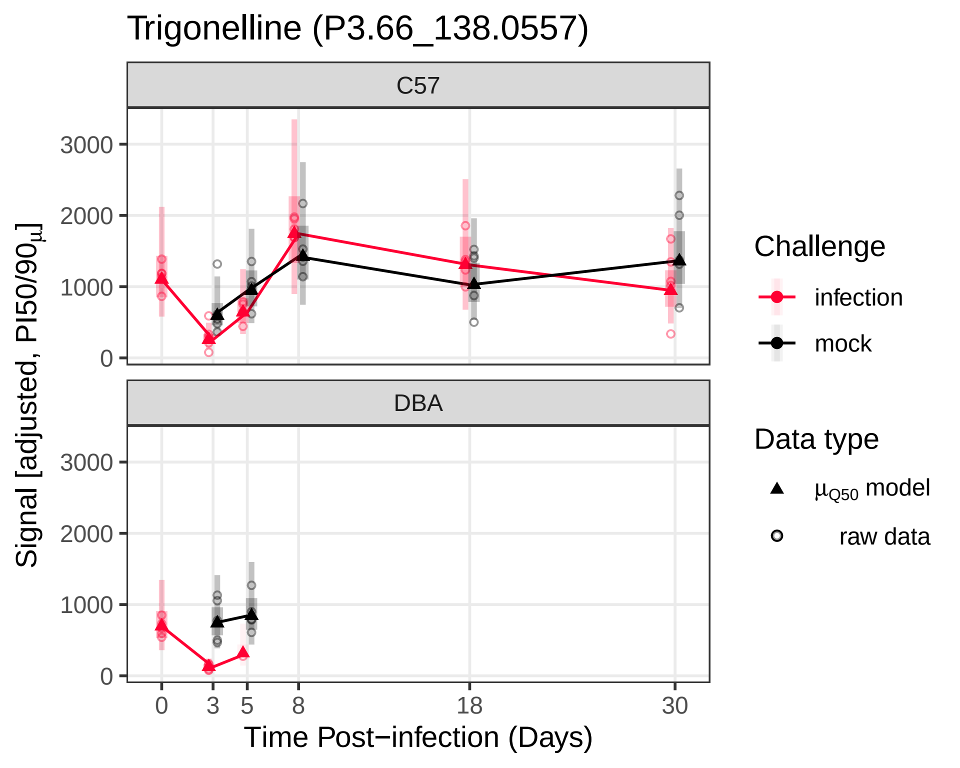

Trigonelline (P3.66_138.0557)
C57
DBA
3000
2000
1000
0
0
3
5
8
18
30
Signal [adjusted, PI50/90μ]
Time Post−infection (Days)
Challenge
infection
mock
Data type
μQ50 model
raw data

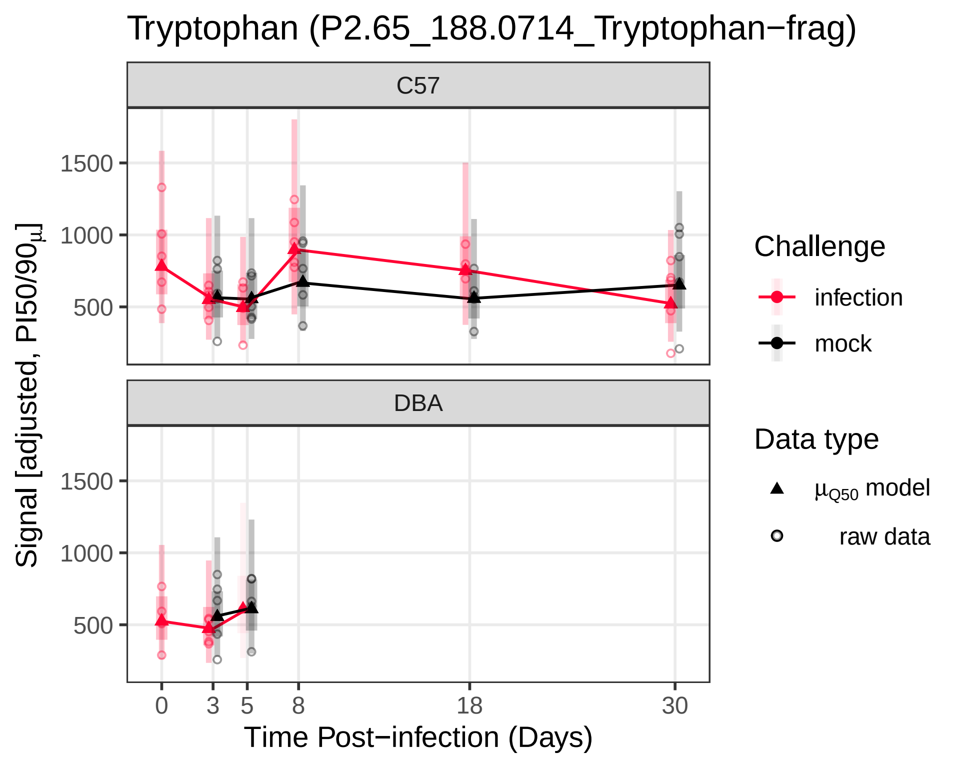

Tryptophan (P2.65_188.0714_Tryptophan−frag)
C57
DBA
Signal [adjusted, PI50/90$_\mu$]
1500
1000
500
0
3
5
8
18
30
Time Post−infection (Days)
Challenge
infection
mock
Data type
μ_{Q50} model
raw data

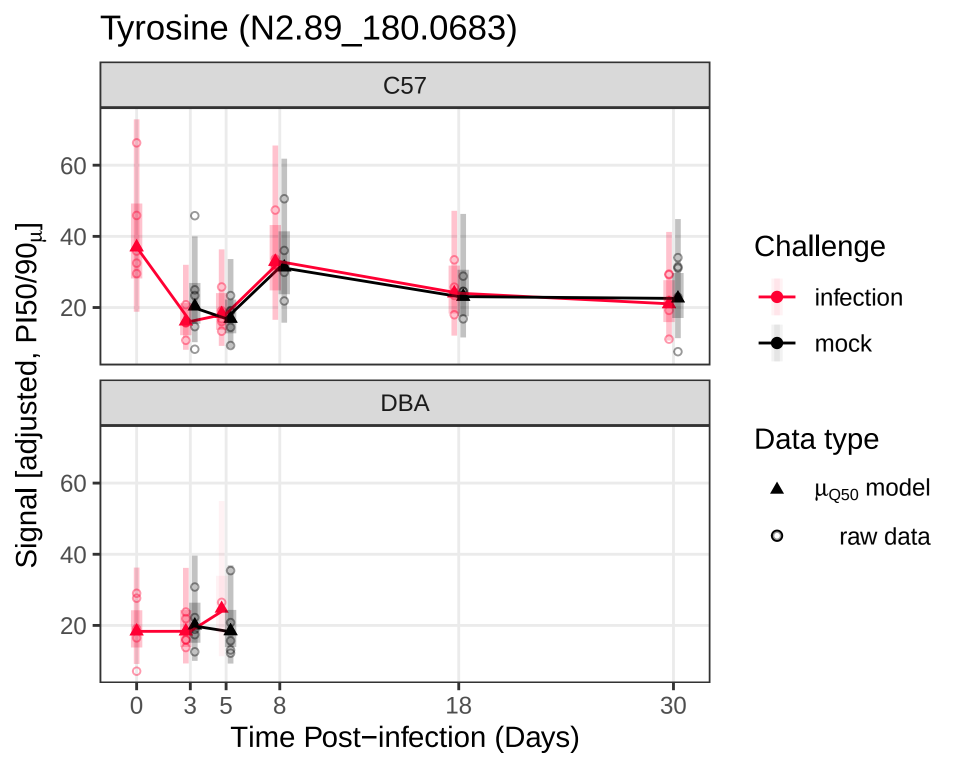

Tyrosine (N2.89_180.0683)
C57
DBA
60
40
20
0
3
5
8
18
30
Time Post−infection (Days)
Signal [adjusted, PI50/90μ]
Challenge
infection
mock
Data type
μQ50 model
raw data

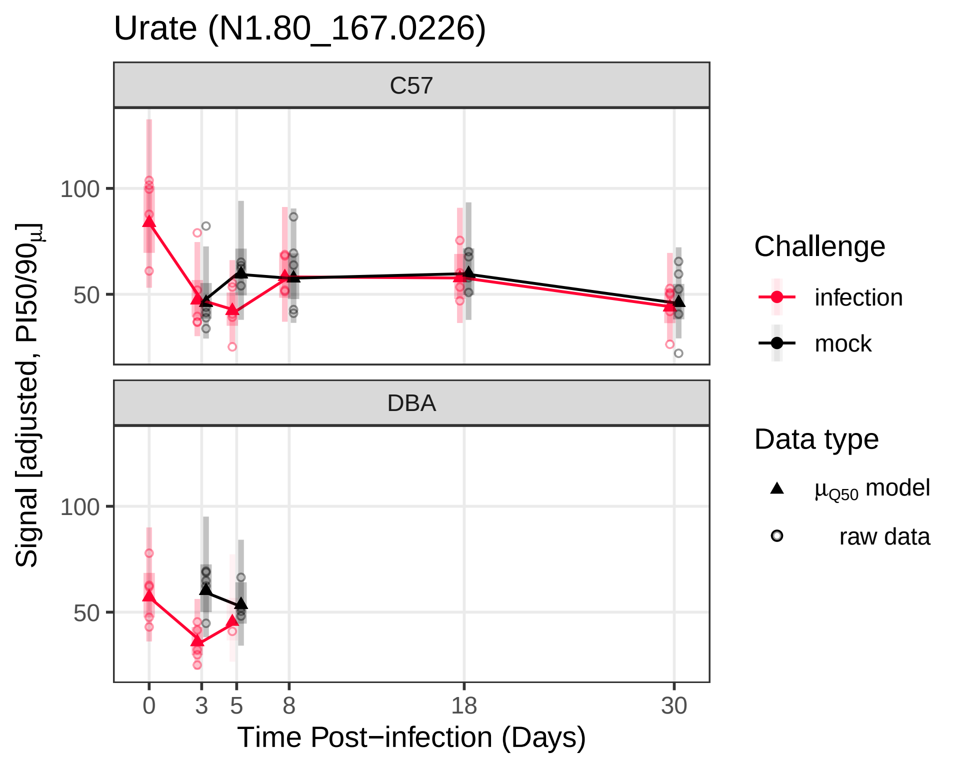

Urate (N1.80_167.0226)
C57
DBA
Signal [adjusted, PI50/90$_{\mu}$]
100
50
100
50
0
3
5
8
18
30
Time Post−infection (Days)
Challenge
infection
mock
Data type
μ_{Q50} model
raw data

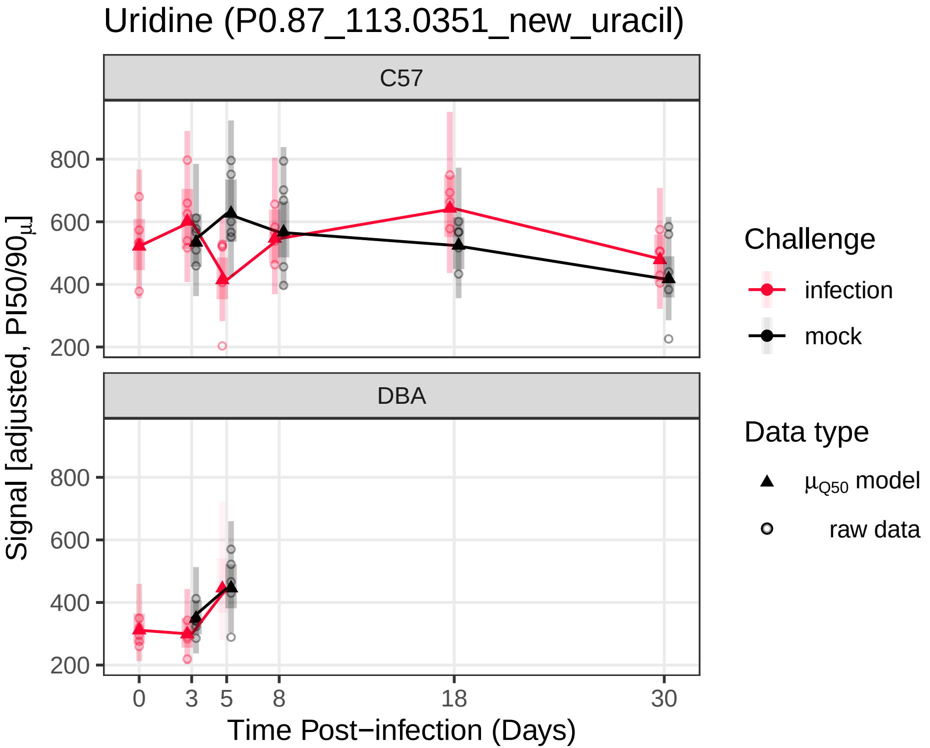

Uridine (P0.87_113.0351_new_uracil)
C57
DBA
800
600
400
200
Signal [adjusted, PI50/90$_{\mu}$]
0
3
5
8
18
30
Time Post−infection (Days)
Challenge
infection
mock
Data type
μ_{Q50} model
raw data

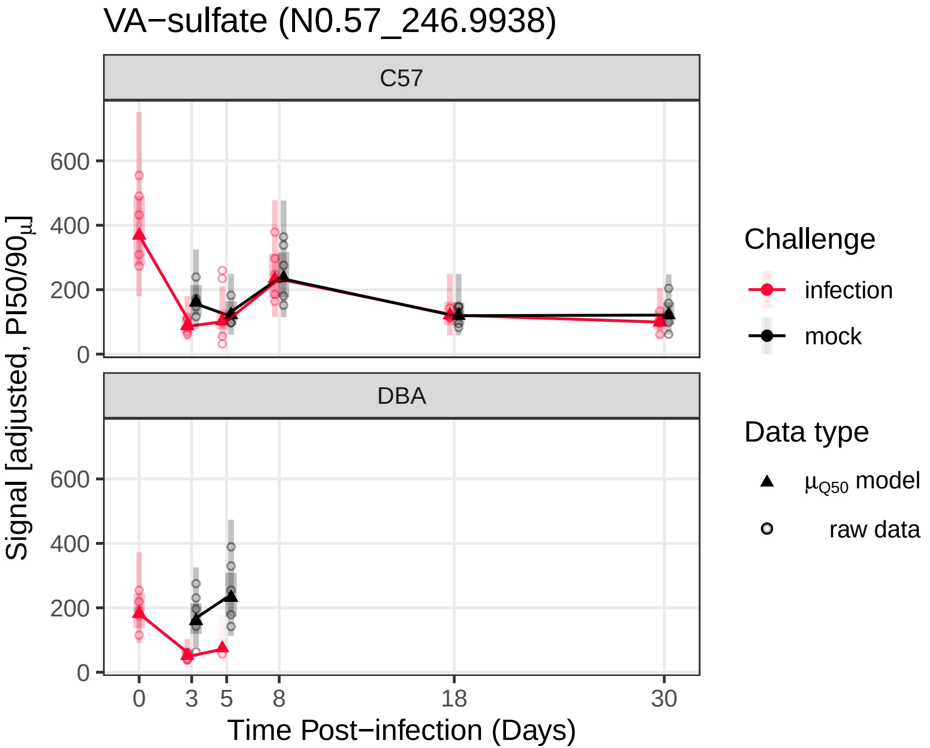
VA−sulfate (N0.57_246.9938)
C57
DBA
Signal [adjusted, PI50/90μ]
0
200
400
600
0
3
5
8
18
30
Time Post−infection (Days)
Challenge
infection
mock
Data type
μQ50 model
raw data

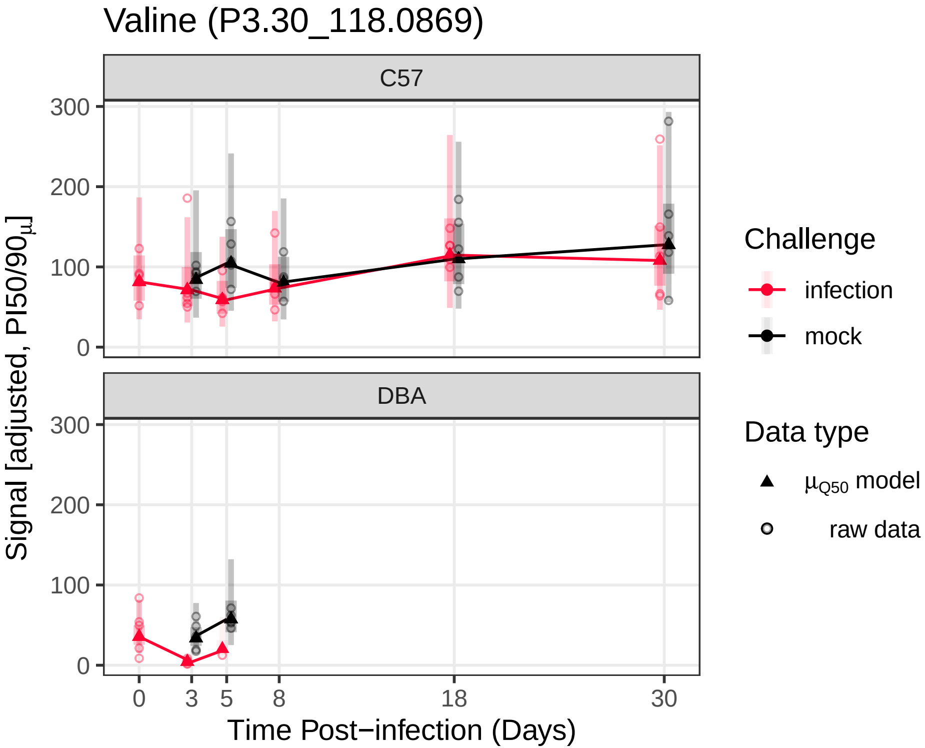

Valine (P3.30_118.0869)
C57
DBA
300
200
100
0
Signal [adjusted, PI50/90μ]
0
3
5
8
18
30
Time Post−infection (Days)
Challenge
infection
mock
Data type
μQ50 model
raw data

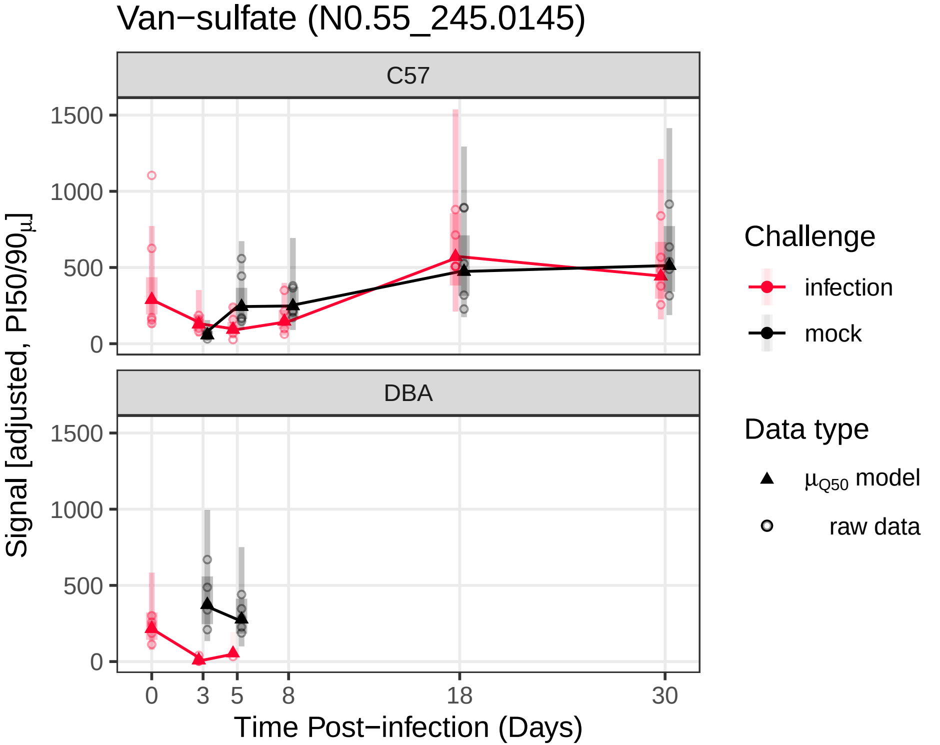

Van−sulfate (N0.55_245.0145)
C57
DBA
1500
1000
500
0
Signal [adjusted, PI50/90μ]
0
3
5
8
18
30
Time Post−infection (Days)
Challenge
infection
mock
Data type
μQ50 model
raw data

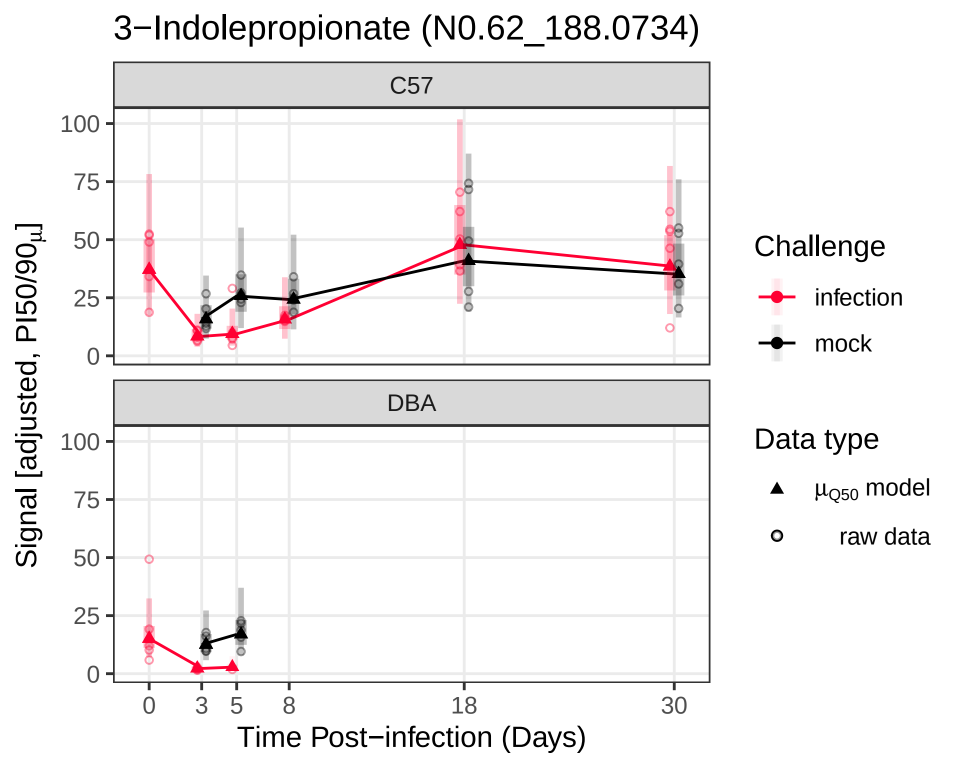
3−Indolepropionate (N0.62_188.0734)
C57
DBA
Signal [adjusted, PI50/90$_{\mu}$]
100
75
50
25
0
0
3
5
8
18
30
Time Post−infection (Days)
Challenge
infection
mock
Data type
μ_{Q50} model
raw data

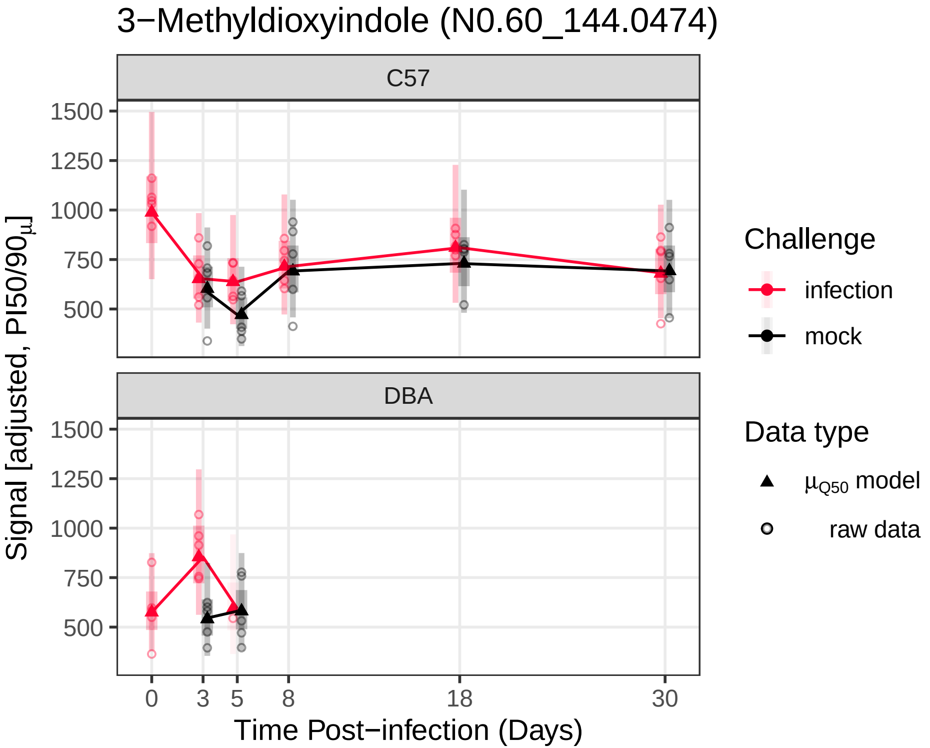

3−Methyldioxyindole (N0.60_144.0474)
C57
DBA
Signal [adjusted, PI50/90μ]
1500
1250
1000
750
500
0
3
5
8
18
30
Time Post−infection (Days)
Challenge
infection
mock
Data type
μQ50 model
raw data

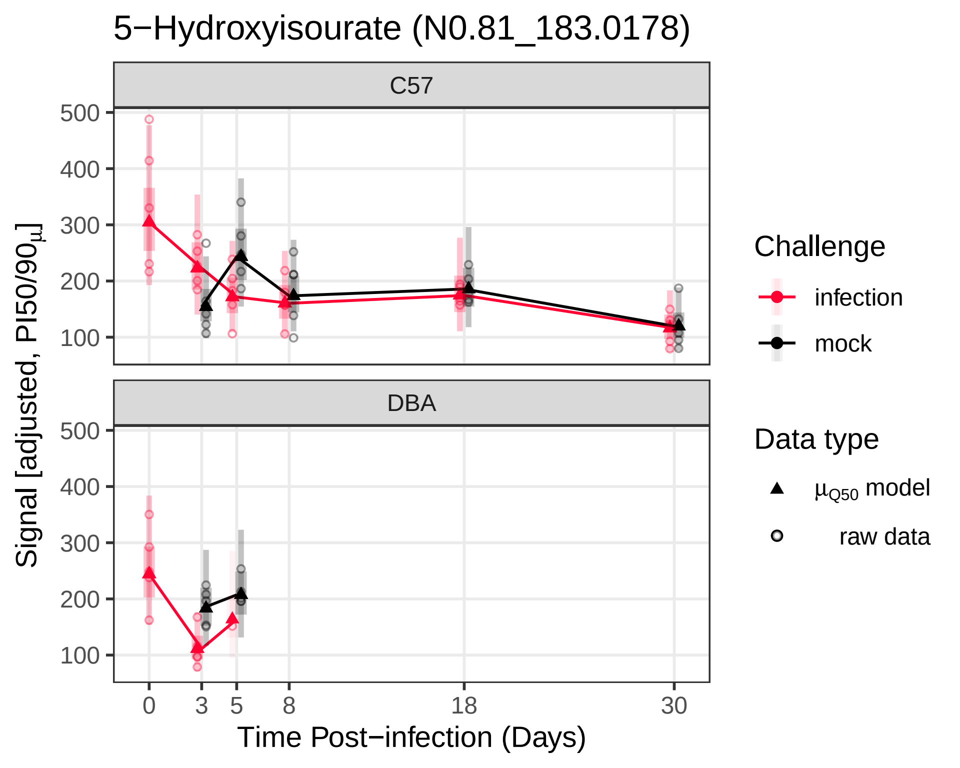

5−Hydroxyisourate (N0.81_183.0178)
C57
DBA
Signal [adjusted, PI50/90μ]
500
400
300
200
100
0
3
5
8
18
30
Time Post−infection (Days)
Challenge
infection
mock
Data type
μQ50 model
raw data

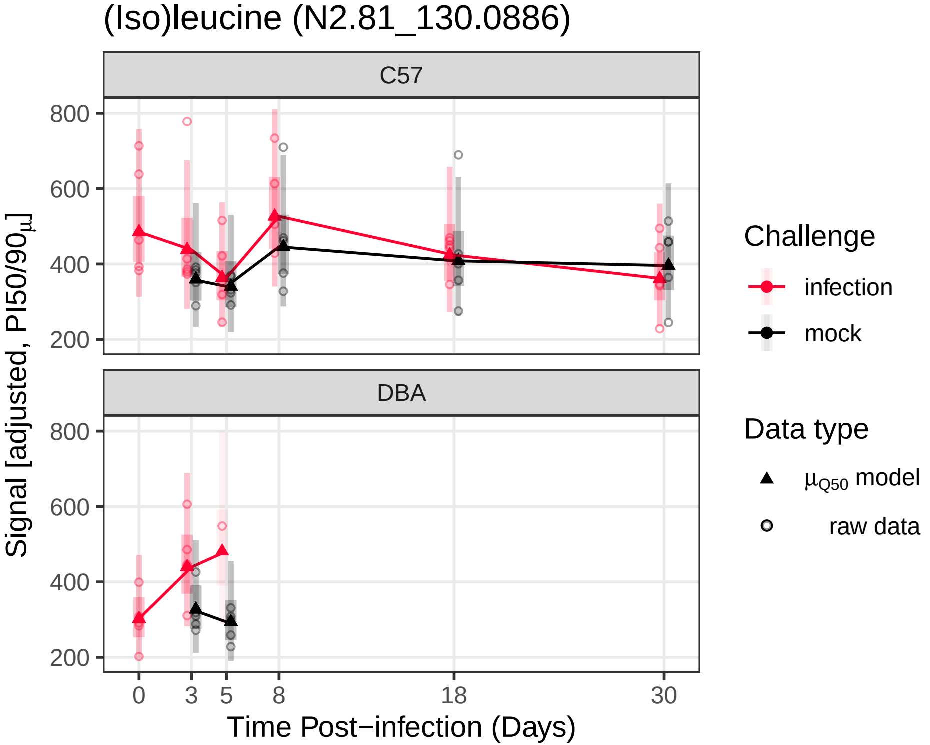
(Iso)leucine (N2.81_130.0886)
C57
DBA
800
600
400
200
Signal [adjusted, PI50/90μ]
0
3
5
8
18
30
Time Post−infection (Days)
Challenge
infection
mock
Data type
μQ50 model
raw data

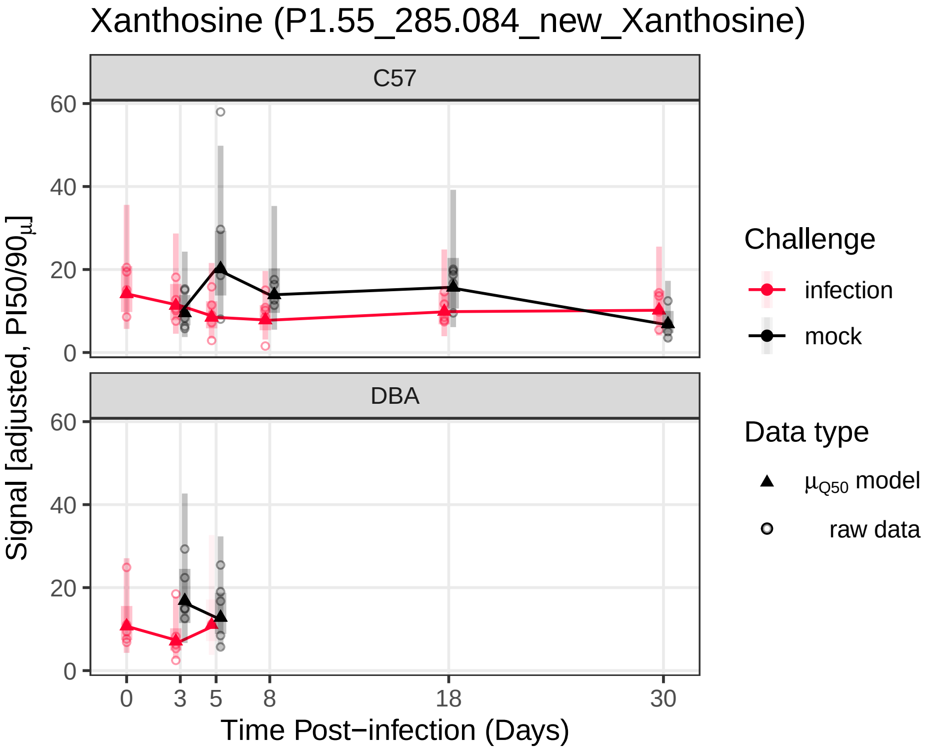
Xanthosine (P1.55_285.084_new_Xanthosine)
C57
DBA
Signal [adjusted, PI50/90$_{\mu}$]
60
40
20
0
Time Post−infection (Days)
0
3
5
8
18
30
Challenge
infection
mock
Data type
μ_{Q50} model
raw data

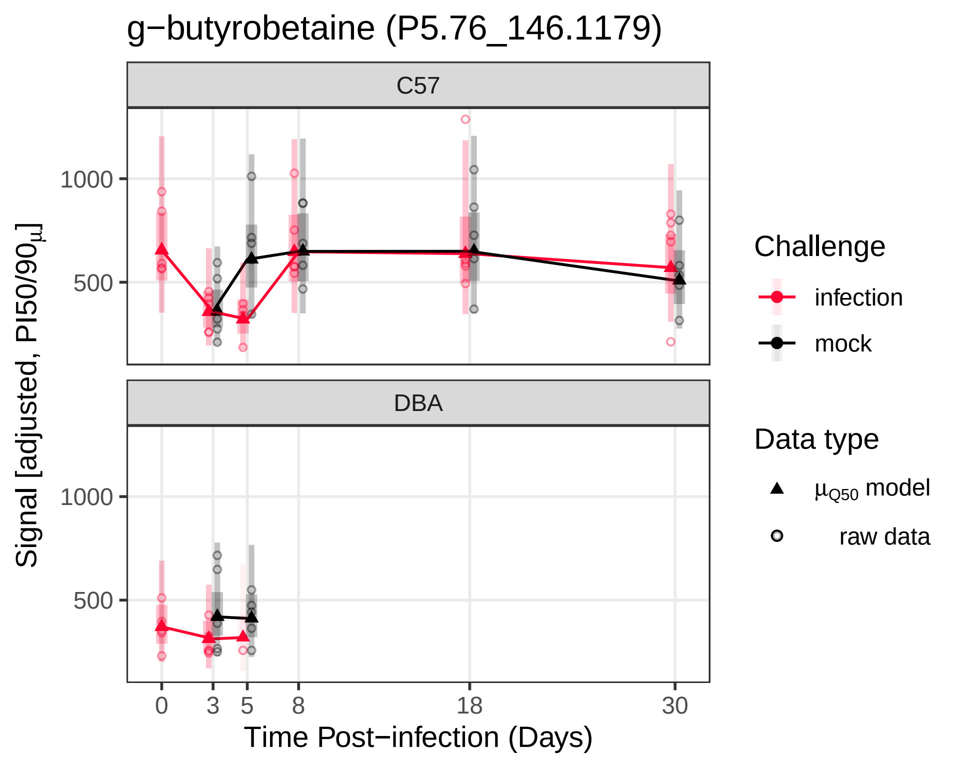
g−butyrobetaine (P5.76_146.1179)
C57
DBA
Signal [adjusted, PI50/90μ]
1000
500
1000
500
0
3
5
8
18
30
Time Post−infection (Days)
Challenge
infection
mock
Data type
μQ50 model
raw data

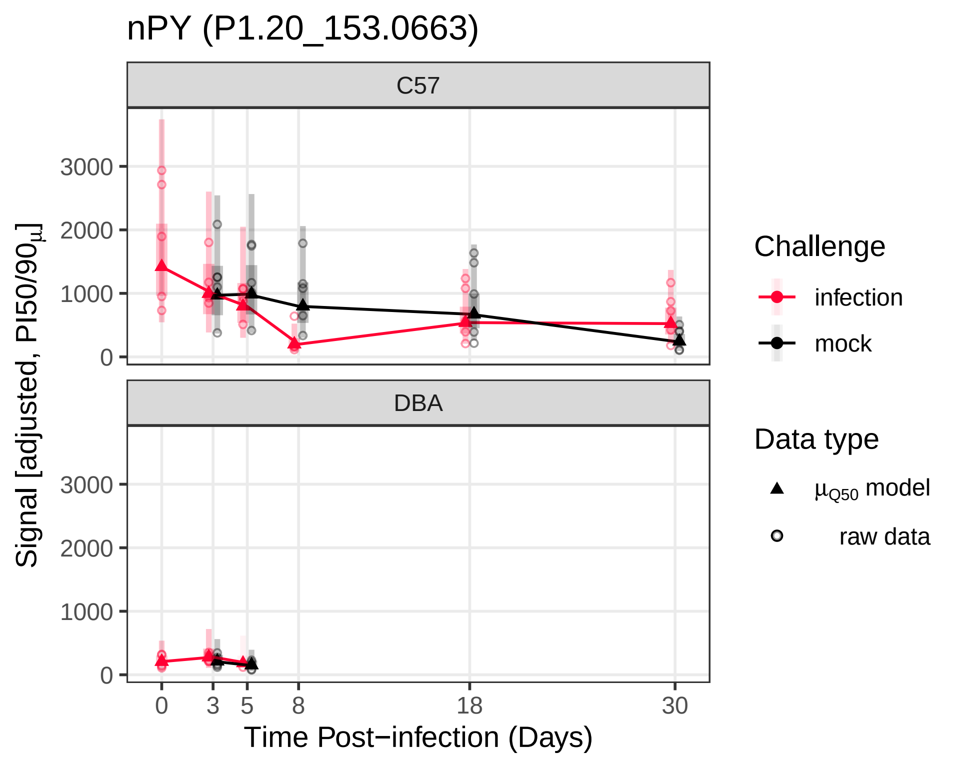
nPY (P1.20_153.0663)
C57
DBA
Signal [adjusted, PI50/90$_{\mu}$]
3000
2000
1000
0
Time Post−infection (Days)
0
3
5
8
18
30
Challenge
infection
mock
Data type
μ_{Q50} model
raw data
