# Supplementary material for: Impaired beta-oxidation increases vulnerability to influenza A infection
Source: J Biol Chem. 2021 Oct 9;297(5):101298. doi: 10.1016/j.jbc.2021.101298 (PMC8564733; doi:10.1016/j.jbc.2021.101298)
Supplement: Figure S3 [file mmc3.pdf]

Impaired beta-oxidation and increased vulnerability to Influenza A infection in a diabetic mouse model.

Sebastiaan van Liempd[1], Diana Cabrera[1], Carolin Pilzner[2], Heike Kollmus[2], Klaus Schughart[2,3,4], Juan M. Falcón-Pérez[1,3]

1. Metabolomics Platform CIC bioGUNE-BRTA, Derio, Spain

2. Department of Infection Genetics, Helmholtz Centre for Infection Research, Braunschweig, Germany.

3. University of Veterinary Medicine Hannover, Hannover, Germany.

4. Department of Microbiology, Immunology and Biochemistry, University of Tennessee Health Science Center, Memphis, TN, USA.

5. IKERBASQUE, Basque Foundation for Science, Bilbao, Spain

Corresponding author:

Sebastiaan van Liempd

CIC bioGUNE, Metabolomics Platform

Parque Tecnológico de Vizcaya Ed. 800

48160, Derio, Bizkaia, Spain

Email: smvanliempd@cicbiogune.es

Figure S3, Heatmap for the effects of all identified metabolites, analogous to Figure 2A and 3.

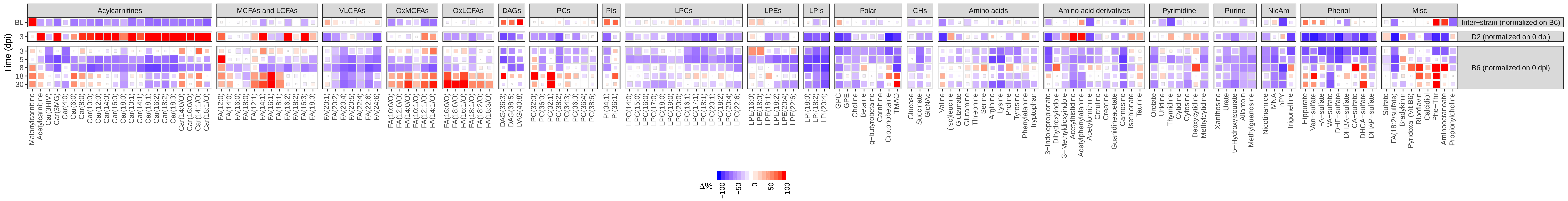
Acylcarnitines
MCFAs and LCFAs
VLCFAs
OxMCFAs
OxLCFAs
DAGs
PCs
PIs
LPCs
LPEs
LPIs
Polar
CHs
Amino acids
Amino acid derivatives
Pyrimidine
Purine
NicAm
Phenol
Misc
Time (dpi)
BL
3
3
5
8
18
30
Inter−strain (normalized on B6)
D2 (normalized on 0 dpi)
B6 (normalized on 0 dpi)
Malonylcarnitine
Acetylcarnitine
Car(3HIV)
Car(3MG)
Car(4:0)
Car(6:0)
Car(8:0)
Car(10:0)
Car(12:0)
Car(14:0)
Car(16:0)
Car(18:0)
Car(10:1)
Car(14:1)
Car(18:1)
Car(16:2)
Car(18:2)
Car(18:3)
Car(14:0|O)
Car(16:0|O)
Car(14:1|O)
Car(18:1|O)
FA(12:0)
FA(14:0)
FA(16:0)
FA(18:0)
FA(12:1)
FA(14:1)
FA(16:1)
FA(18:1)
FA(16:2)
FA(18:2)
FA(16:3)
FA(18:3)
FA(20:1)
FA(20:2)
FA(20:4)
FA(20:5)
FA(22:4)
FA(22:6)
FA(24:6)
FA(10:0|O)
FA(12:0|O)
FA(14:0|O)
FA(10:1|O)
FA(12:1|O)
FA(14:1|O)
FA(16:0|O)
FA(18:0|O)
FA(16:1|O)
FA(18:1|O)
FA(18:2|O)
FA(18:3|O)
DAG(36:3)
DAG(38:5)
DAG(40:8)
PC(32:0)
PC(36:0)
PC(32:1)
PC(38:2)
PC(34:3)
PC(36:3)
PC(36:4)
PC(38:6)
PI(34:1)
PI(36:1)
LPC(14:0)
LPC(15:0)
LPC(16:0)
LPC(17:0)
LPC(18:0)
LPC(19:0)
LPC(20:0)
LPC(16:1)
LPC(17:1)
LPC(18:1)
LPC(20:1)
LPC(18:2)
LPC(20:4)
LPC(22:6)
LPE(16:0)
LPE(18:0)
LPE(18:1)
LPE(18:2)
LPE(20:4)
LPE(22:6)
LPI(18:0)
LPI(18:2)
LPI(20:4)
GPC
GPE
Choline
Betaine
g−butyrobetaine
Carnitine
Crotonobetaine
TMAO
Glucose
Succinate
GlcNAc
Valine
(Iso)leucine
Glutamate
Glutamine
Threonine
Serine
Arginine
Lysine
Proline
Tyrosine
Phenylalanine
Tryptophan
3−Indolepropionate
Dihydroxyindole
3−Methyldioxyindole
Acetylhistidine
Acetylphenylalanine
Acetylornithine
Citruline
Creatine
Guanidineacetate
Carnosine
Isethionate
Taurine
Orotate
Uridine
Thymidine
Cytidine
Cytosine
Deoxycytidine
Methylcytidine
Xanthosine
Urate
5−Hydroxyisourate
Allantoin
Methylguanosine
Nicotinamide
MNA
nPY
Trigonelline
Hippurate
Van−sulfate
FA−sulfate
VA−sulfate
DHF−sulfate
DHBA−sulfate
CA−sulfate
DHCA−sulfate
DHAP−sulfate
Sulfate
FA(18:2/sulfate)
Bradykinin
Pyridoxal (Vit B6)
Riboflavin
Calcidiol
Phe−Thr
Aminooctanoate
Propionylcholine
Δ%
−100
−50
0
50
100
